# Supplementary figures and images for: PEBP1 amplifies mitochondrial dysfunction-induced integrated stress response (part 1 of 3)
Source: eLife. 2025 Jan 29;13:RP102852. doi: 10.7554/eLife.102852 (PMC11778924; doi:10.7554/eLife.102852)

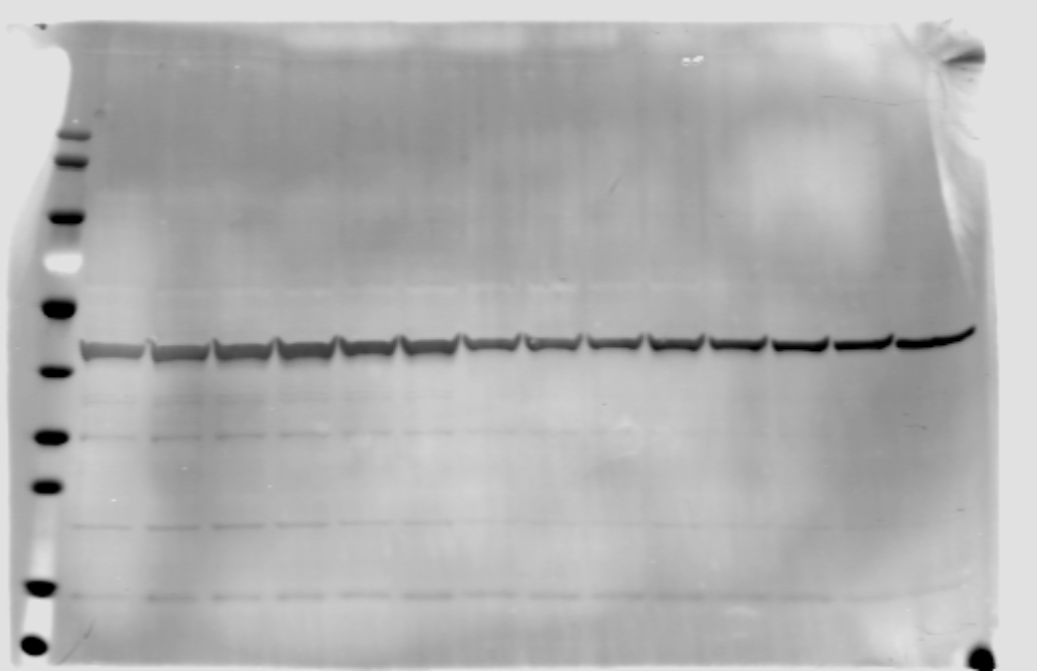

Supplement: Figure 1—source data 1. [file elife-102852-fig1-data1.zip › Figure 1-source data 1/Fig1F_Actin_original.tif]

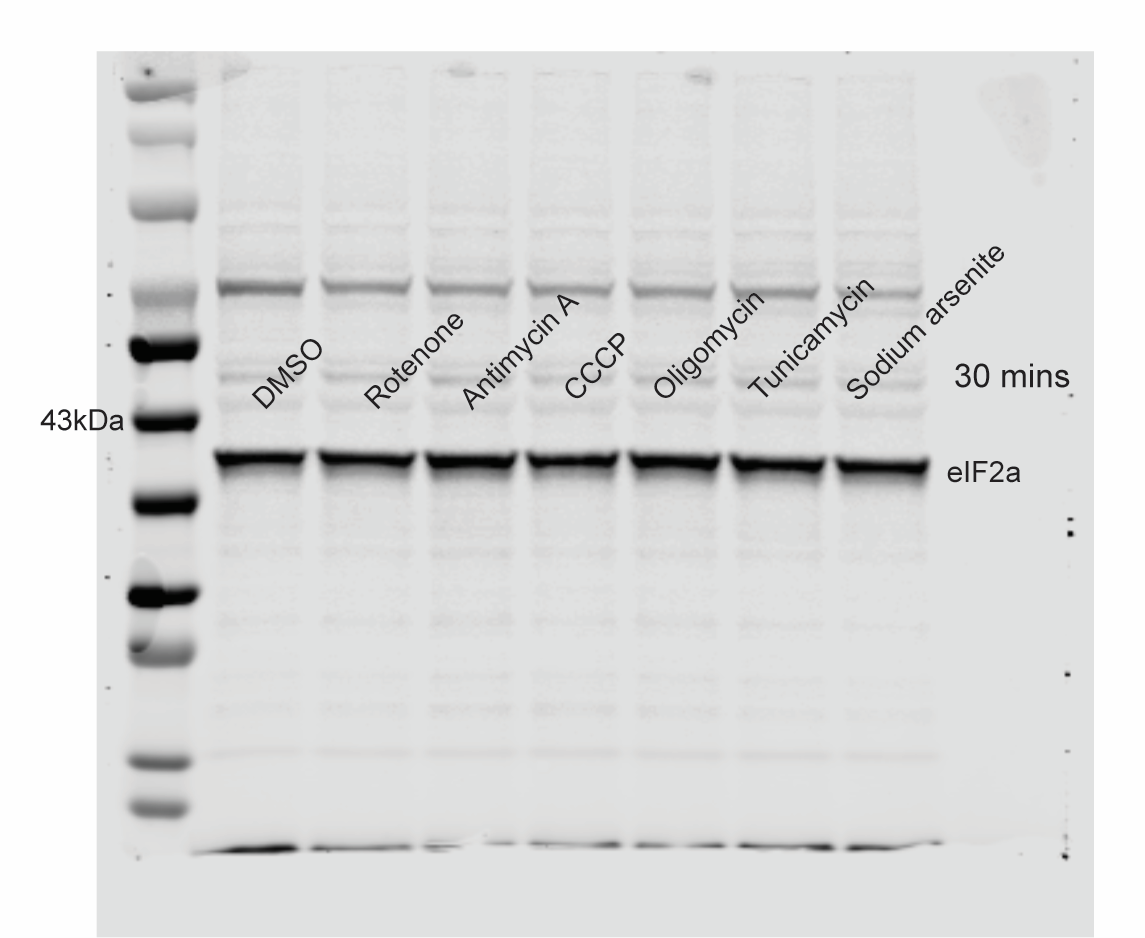

Supplement: Figure 1—source data 1. [file elife-102852-fig1-data1.zip › Figure 1-source data 1/Fig1G_eIF2a_band_indicated.tif]

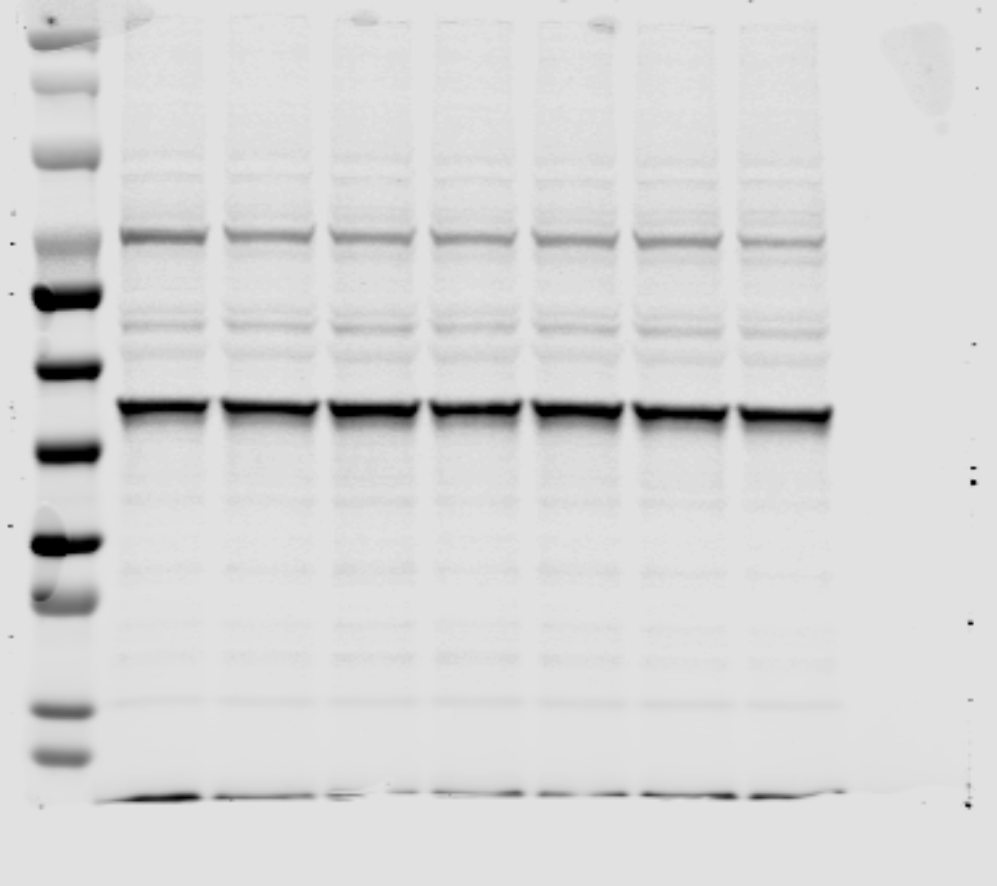

Supplement: Figure 1—source data 1. [file elife-102852-fig1-data1.zip › Figure 1-source data 1/Fig1G_eIF2a_original.tif]

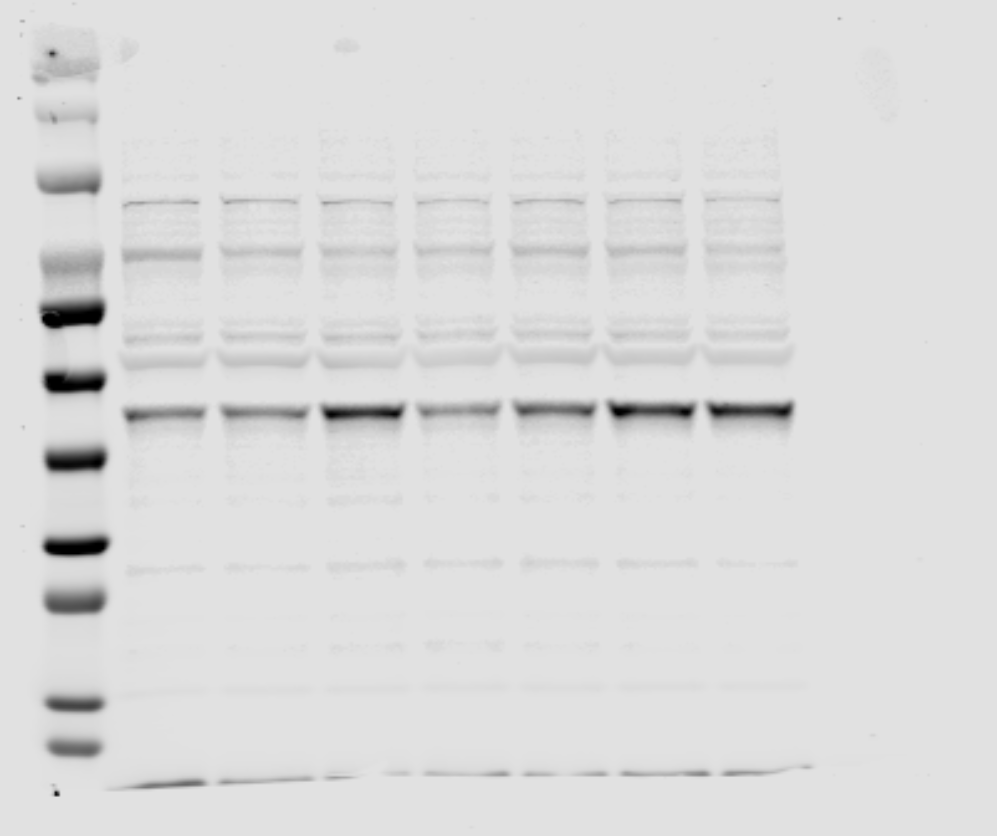

Supplement: Figure 1—source data 1. [file elife-102852-fig1-data1.zip › Figure 1-source data 1/Fig1G_P-eIF2a_original.tif]

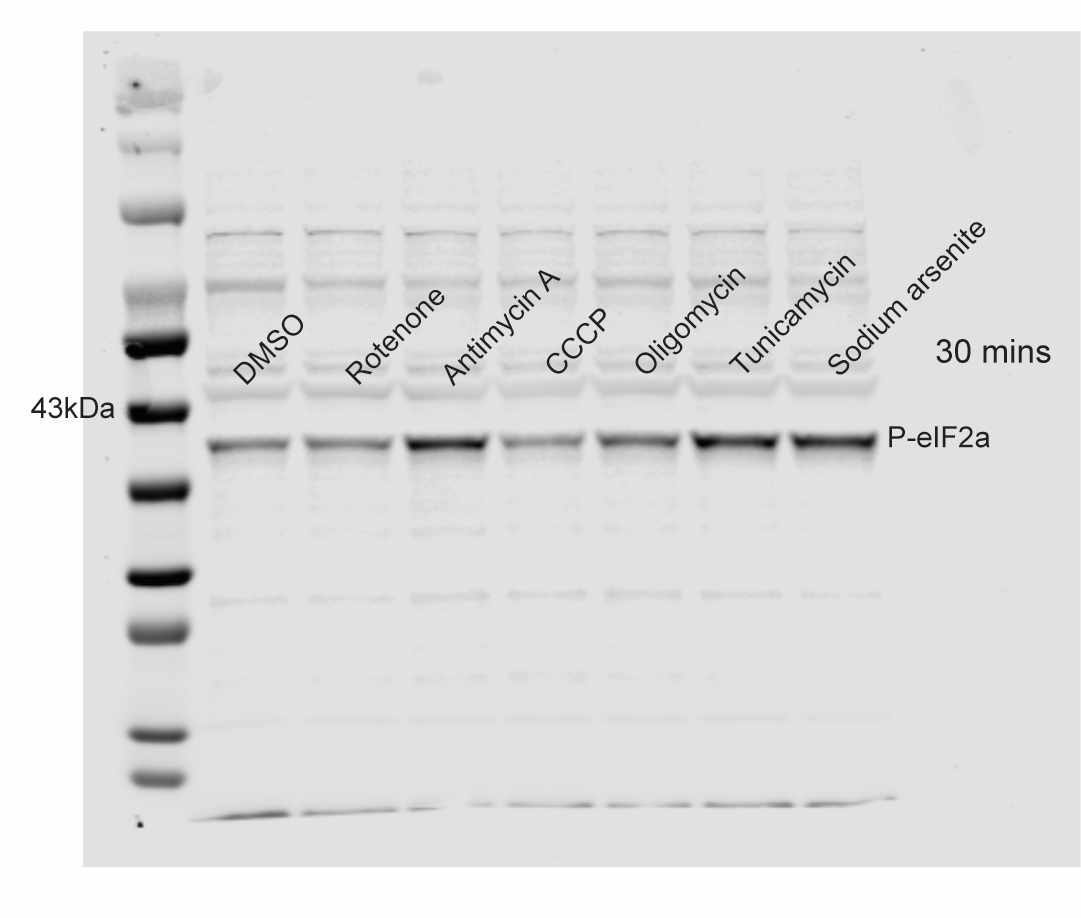

Supplement: Figure 1—source data 1. [file elife-102852-fig1-data1.zip › Figure 1-source data 1/Fig1G_P-eIF2a_band_indicated.tif]

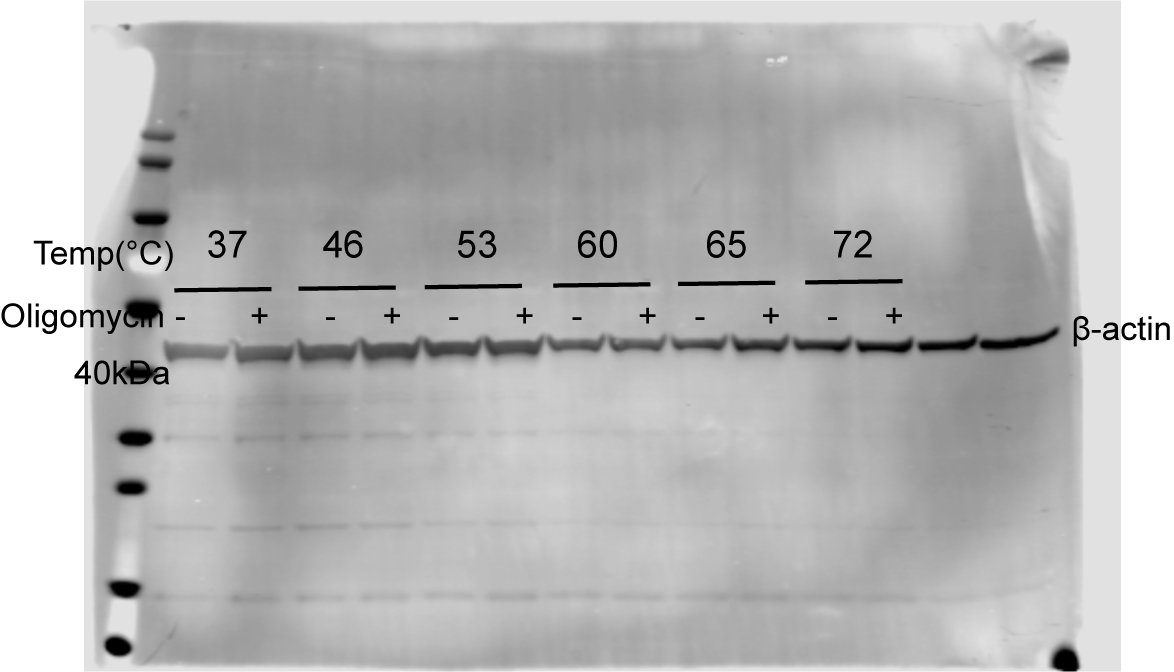

Supplement: Figure 1—source data 1. [file elife-102852-fig1-data1.zip › Figure 1-source data 1/Fig1F_Actin_band_indicated.tif]

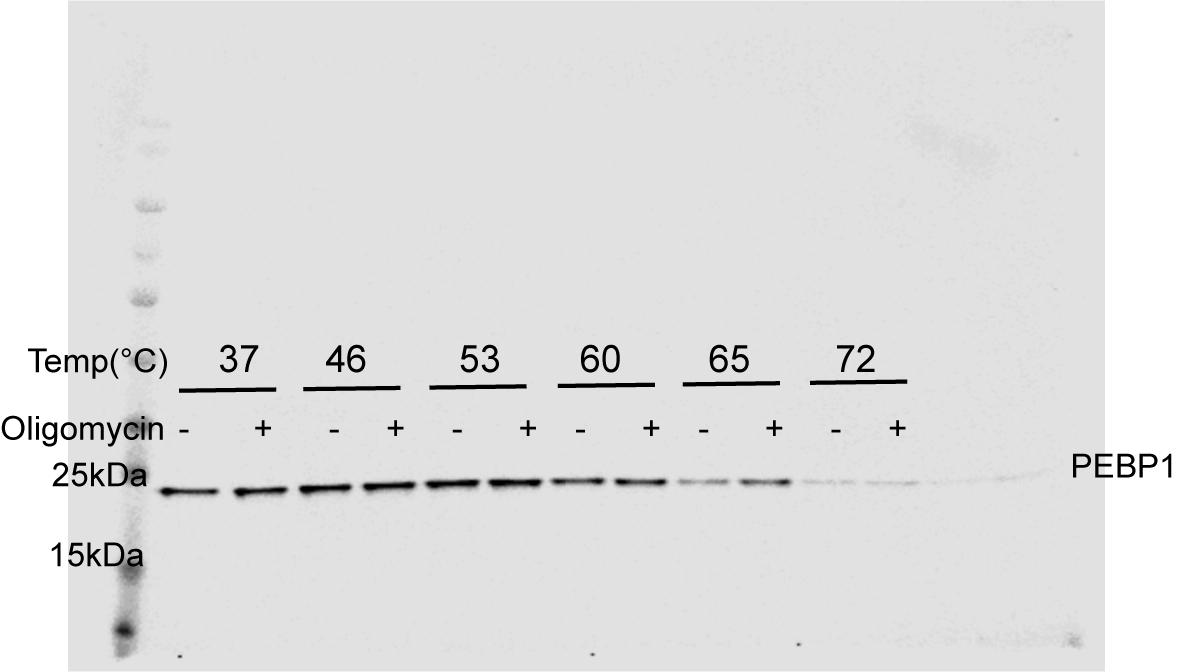

Supplement: Figure 1—source data 1. [file elife-102852-fig1-data1.zip › Figure 1-source data 1/Fig1F_PEBP1_band_indicated.tif]

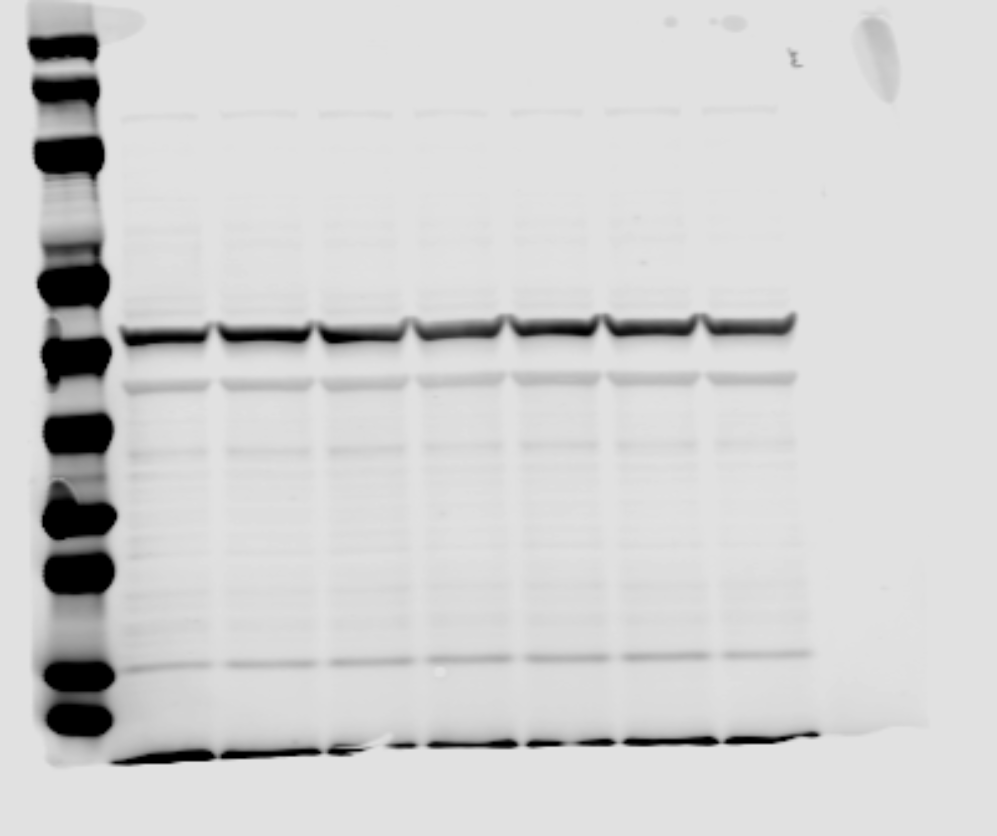

Supplement: Figure 1—source data 1. [file elife-102852-fig1-data1.zip › Figure 1-source data 1/Fig1G_Actin_original.tif]

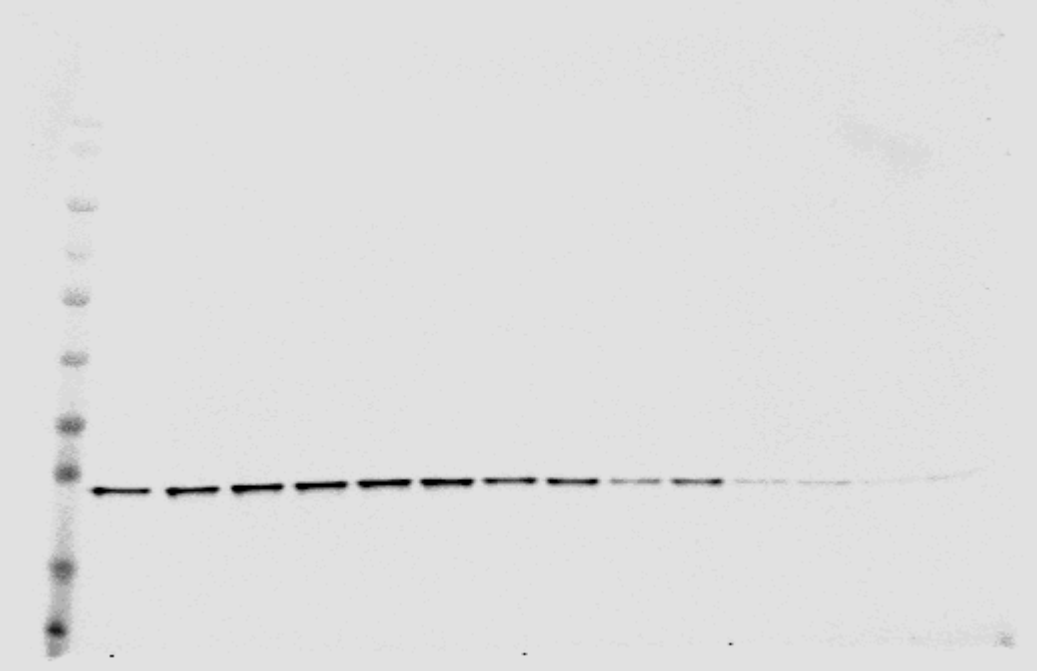

Supplement: Figure 1—source data 1. [file elife-102852-fig1-data1.zip › Figure 1-source data 1/Fig1F_PEBP1_original.tif]

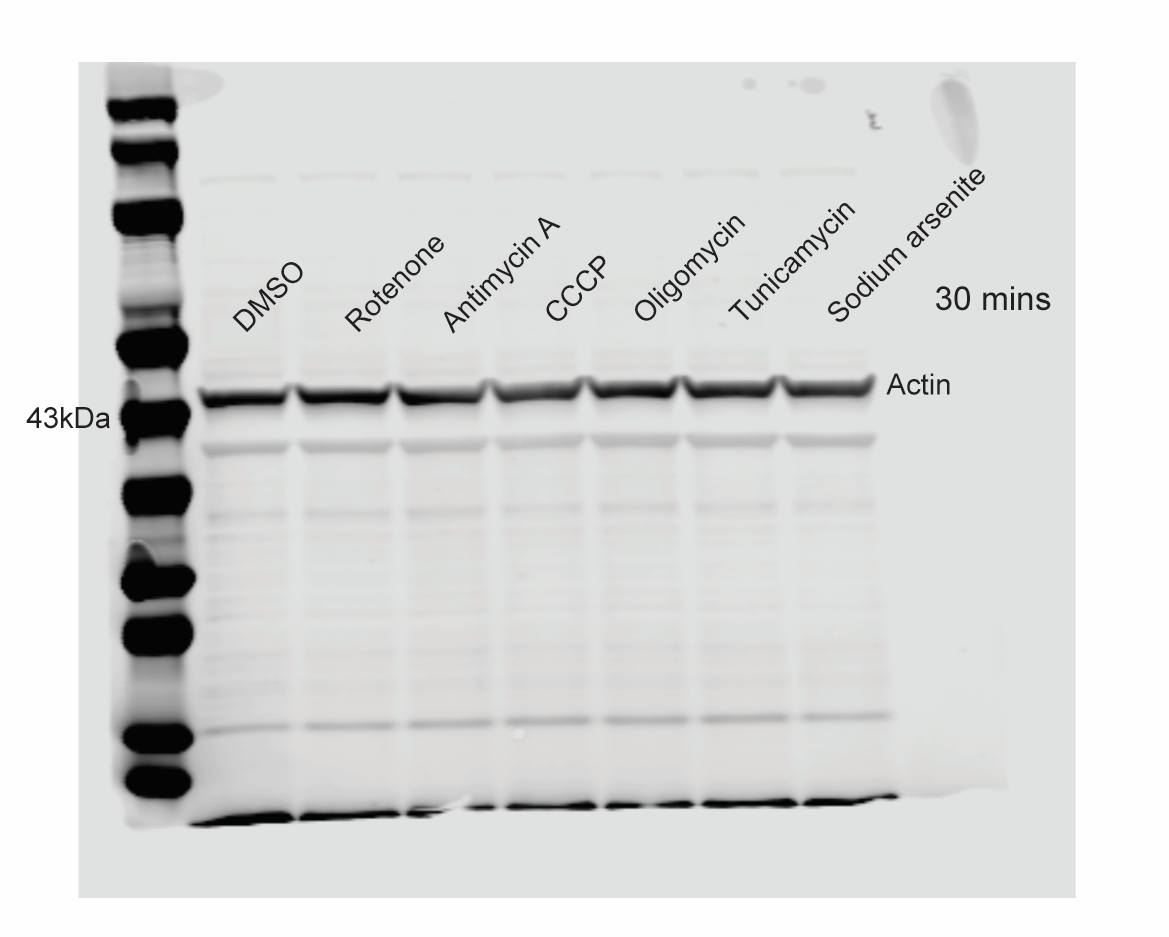

Supplement: Figure 1—source data 1. [file elife-102852-fig1-data1.zip › Figure 1-source data 1/Fig1G_Actin_band_indicated.tif]

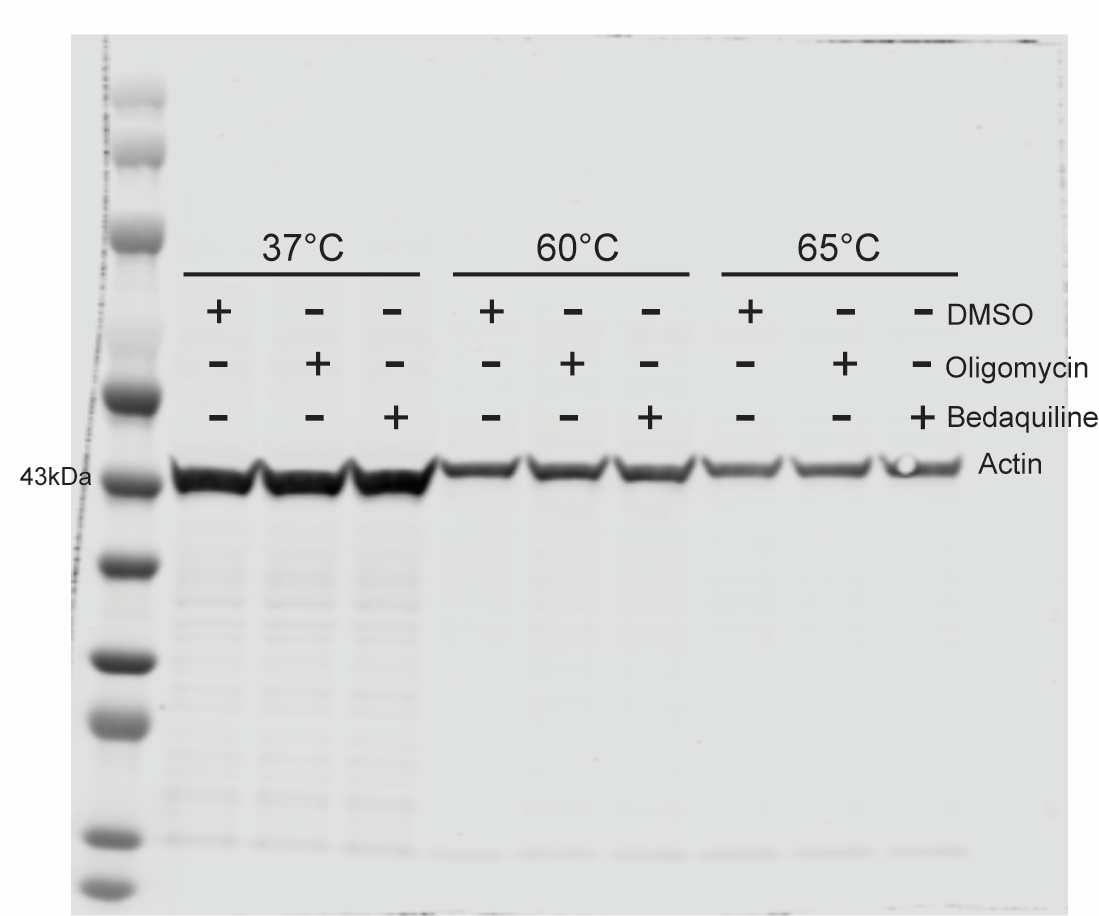

Supplement: Figure 1—figure supplement 1—source data 1. [file elife-102852-fig1-figsupp1-data1.zip › Figure 1-source data 2/Fig1Supplement1C_Actin_band_indicated.tif]

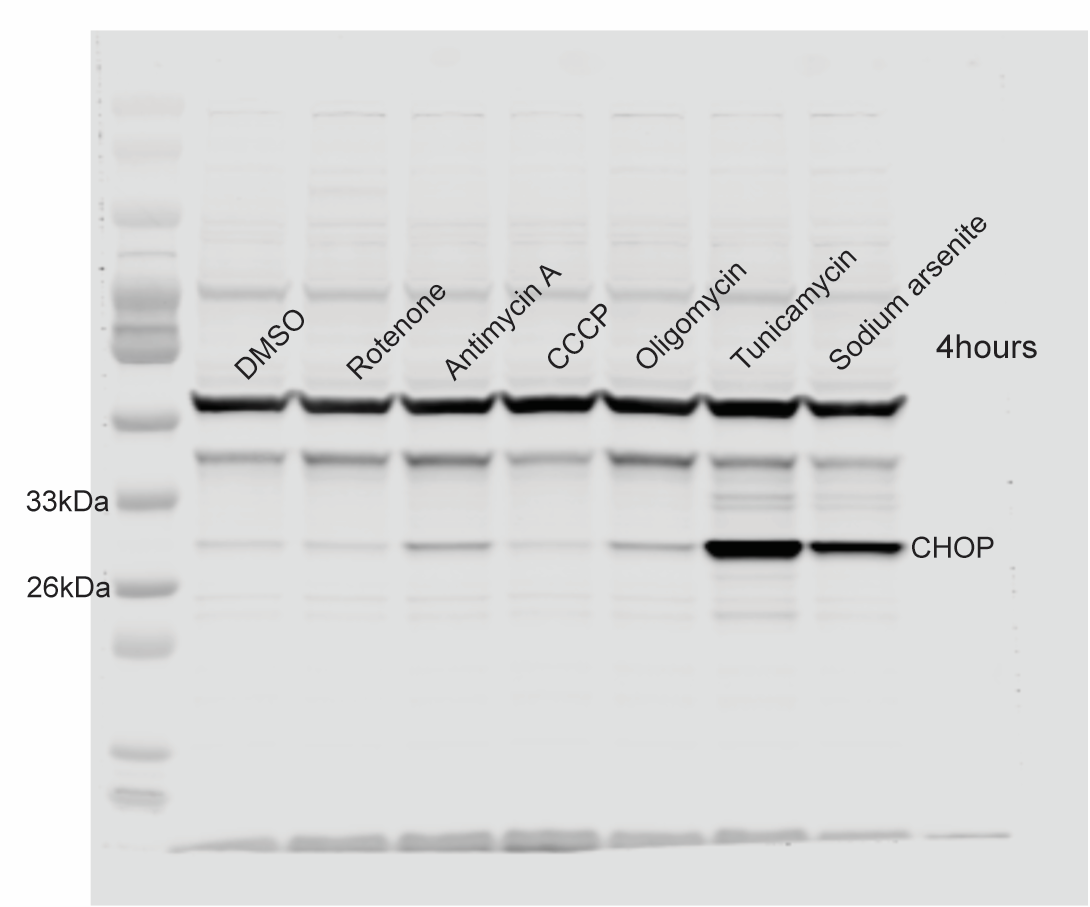

Supplement: Figure 1—figure supplement 1—source data 1. [file elife-102852-fig1-figsupp1-data1.zip › Figure 1-source data 2/Fig1Supplement1F_CHOP_band_indicated.tif]

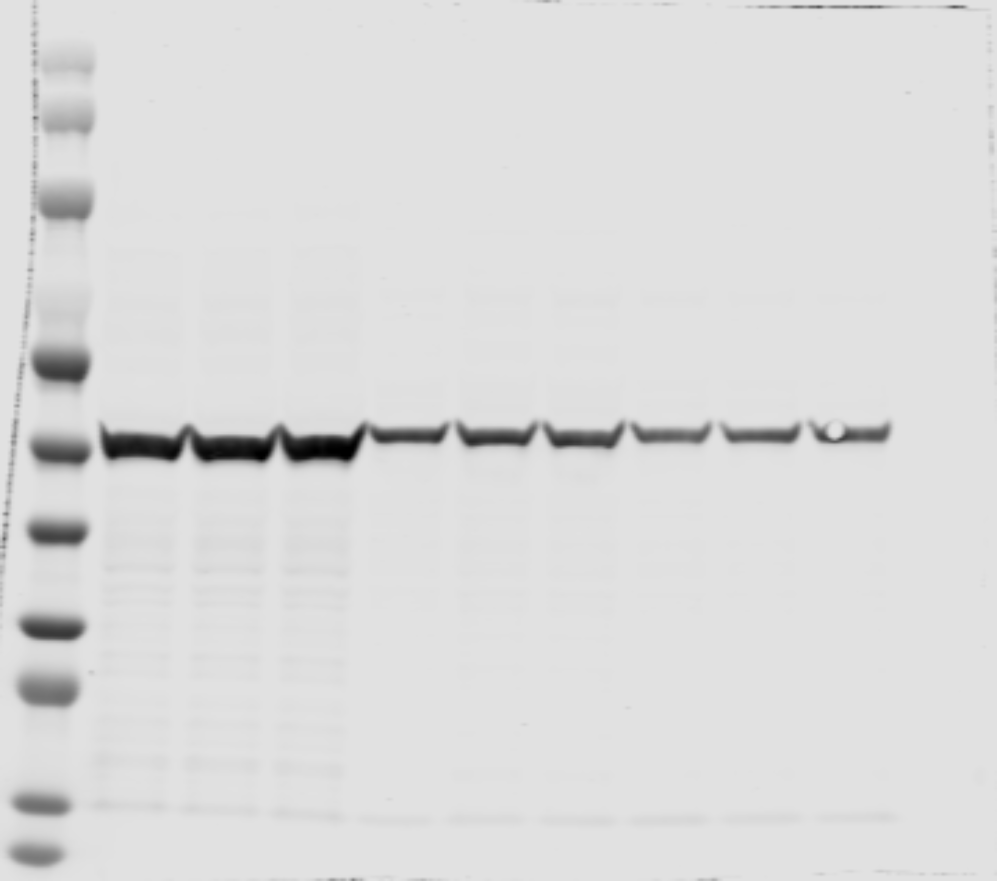

Supplement: Figure 1—figure supplement 1—source data 1. [file elife-102852-fig1-figsupp1-data1.zip › Figure 1-source data 2/Fig1Supplement1C_Actin_original.tif]

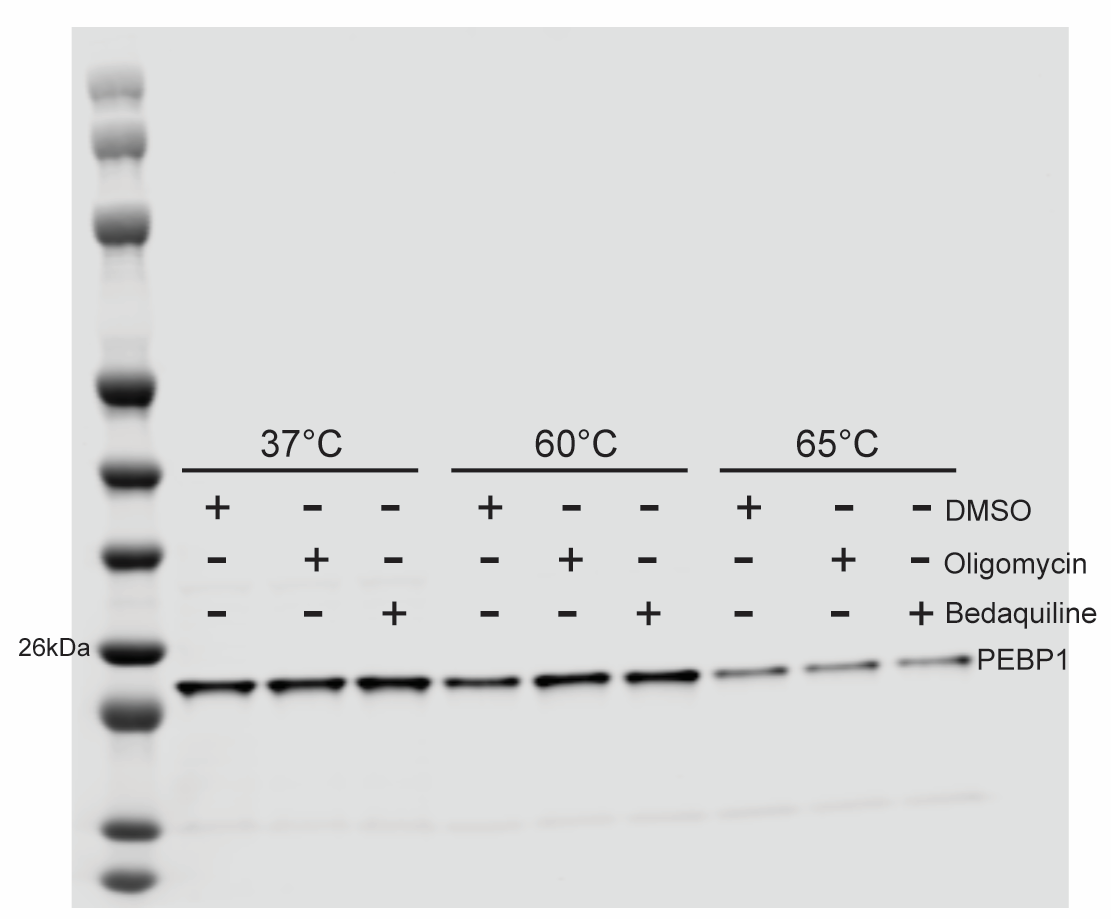

Supplement: Figure 1—figure supplement 1—source data 1. [file elife-102852-fig1-figsupp1-data1.zip › Figure 1-source data 2/Fig1Supplement1C_PEBP1_band_indicated.tif]

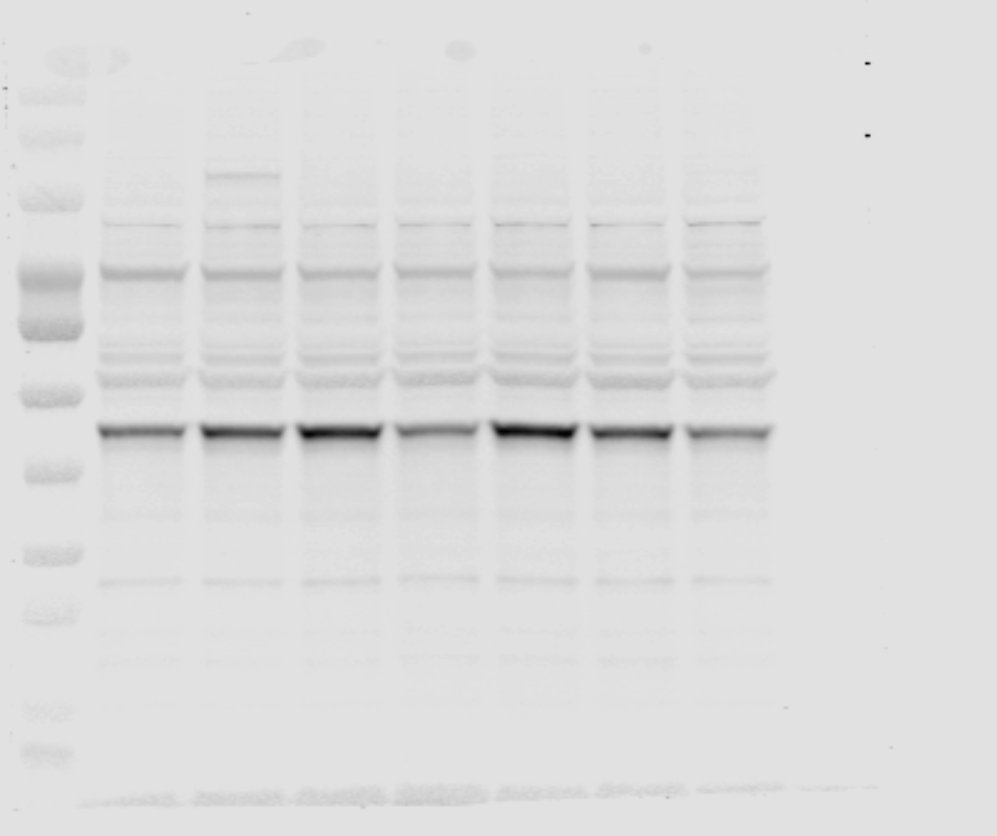

Supplement: Figure 1—figure supplement 1—source data 1. [file elife-102852-fig1-figsupp1-data1.zip › Figure 1-source data 2/Fig1Supplement1F_P-eIF2a_original.tif]

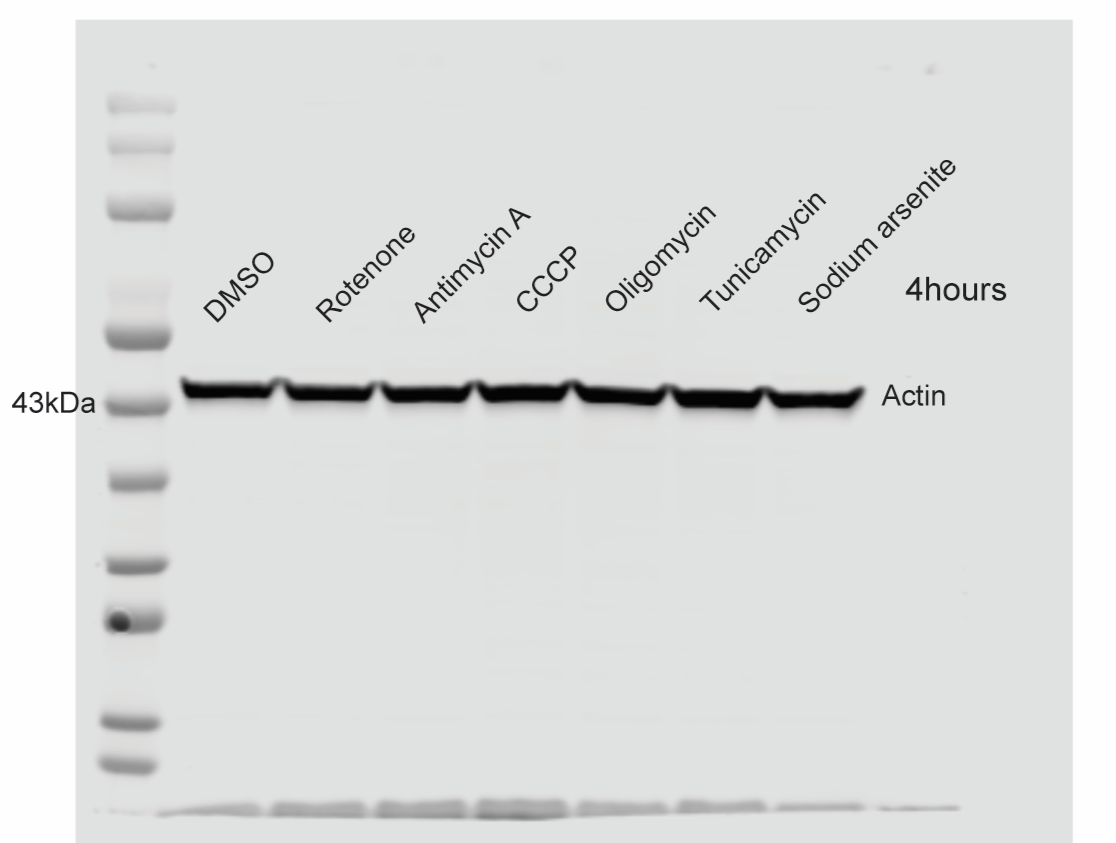

Supplement: Figure 1—figure supplement 1—source data 1. [file elife-102852-fig1-figsupp1-data1.zip › Figure 1-source data 2/Fig1Supplement1F_Actin_band_indicated.tif]

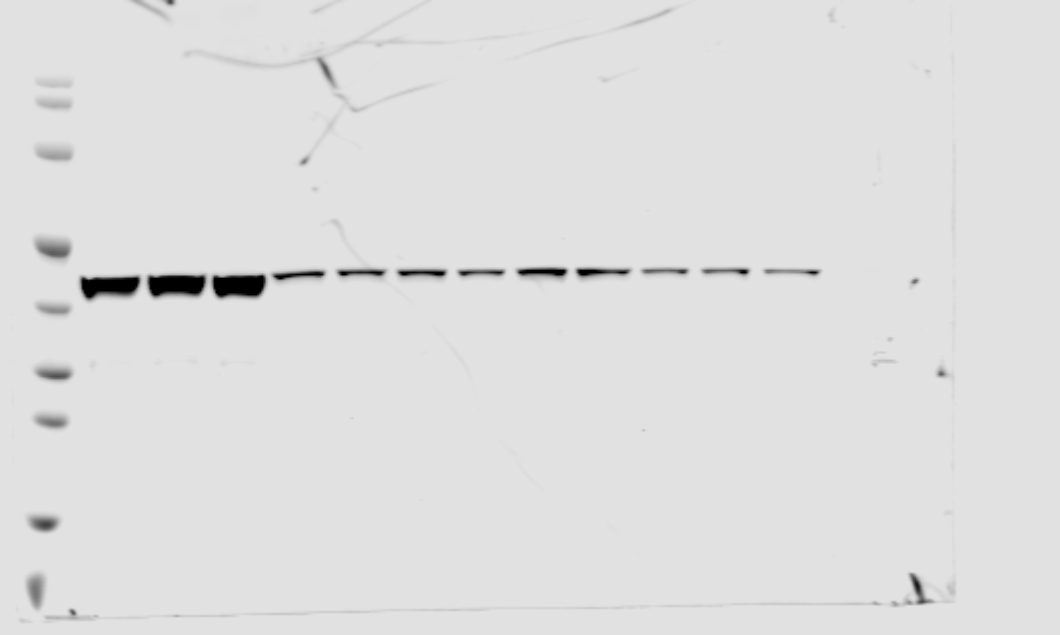

Supplement: Figure 1—figure supplement 1—source data 1. [file elife-102852-fig1-figsupp1-data1.zip › Figure 1-source data 2/Fig1Supplement1E_Actin_original.tif]

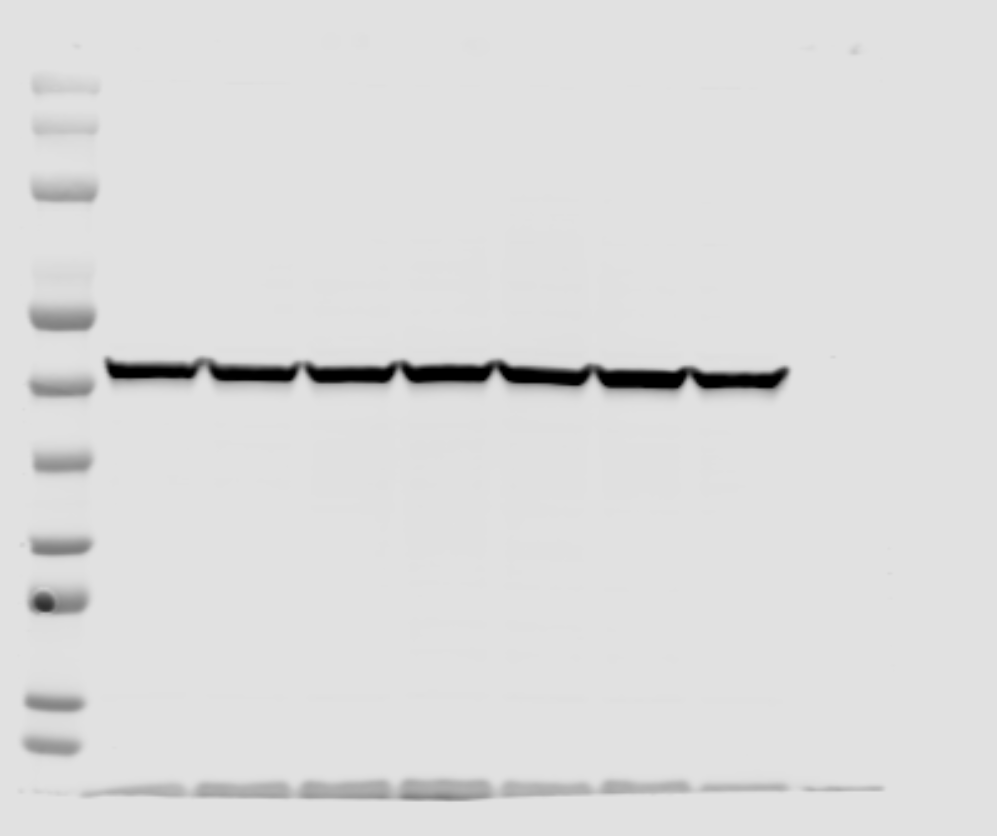

Supplement: Figure 1—figure supplement 1—source data 1. [file elife-102852-fig1-figsupp1-data1.zip › Figure 1-source data 2/Fig1Supplement1F_Actin_original.tif]

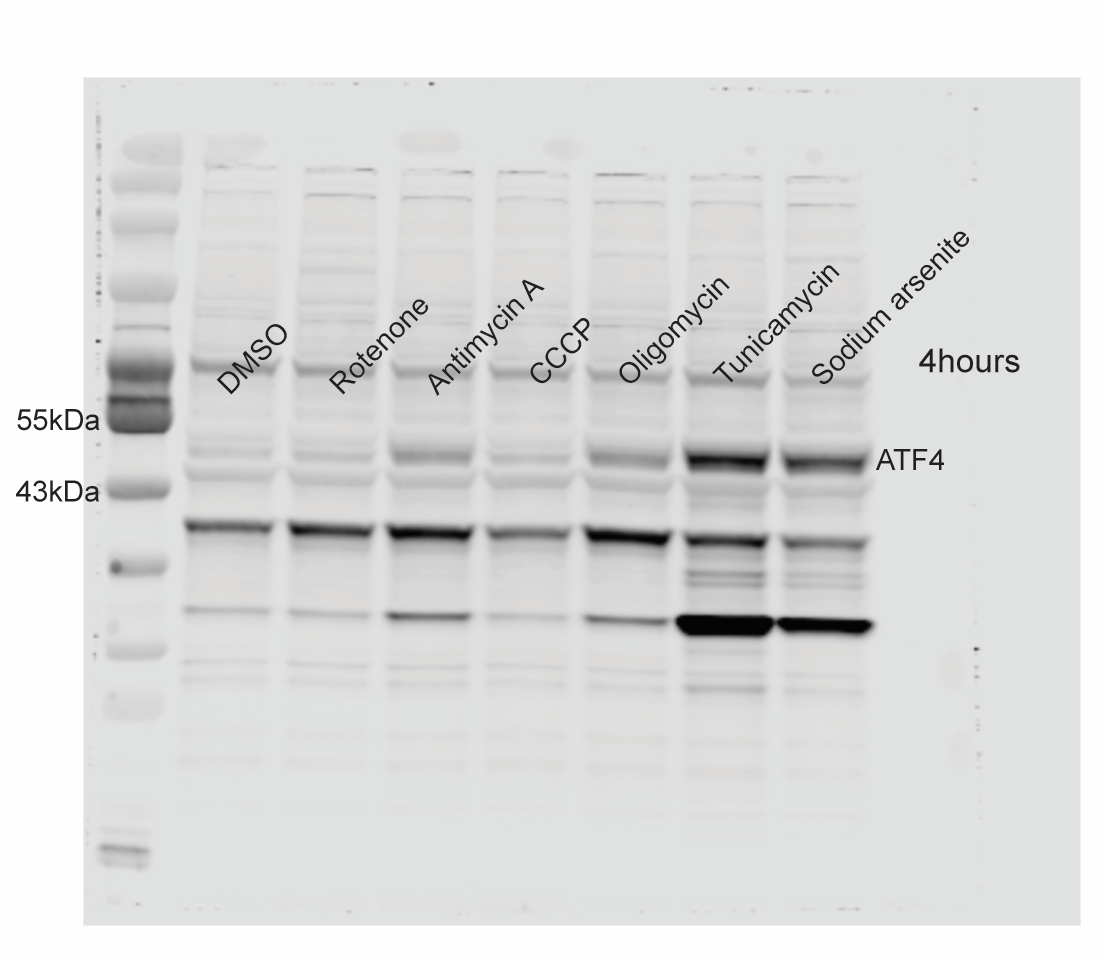

Supplement: Figure 1—figure supplement 1—source data 1. [file elife-102852-fig1-figsupp1-data1.zip › Figure 1-source data 2/Fig1Supplement1F_ATF4_band_indicated.tif]

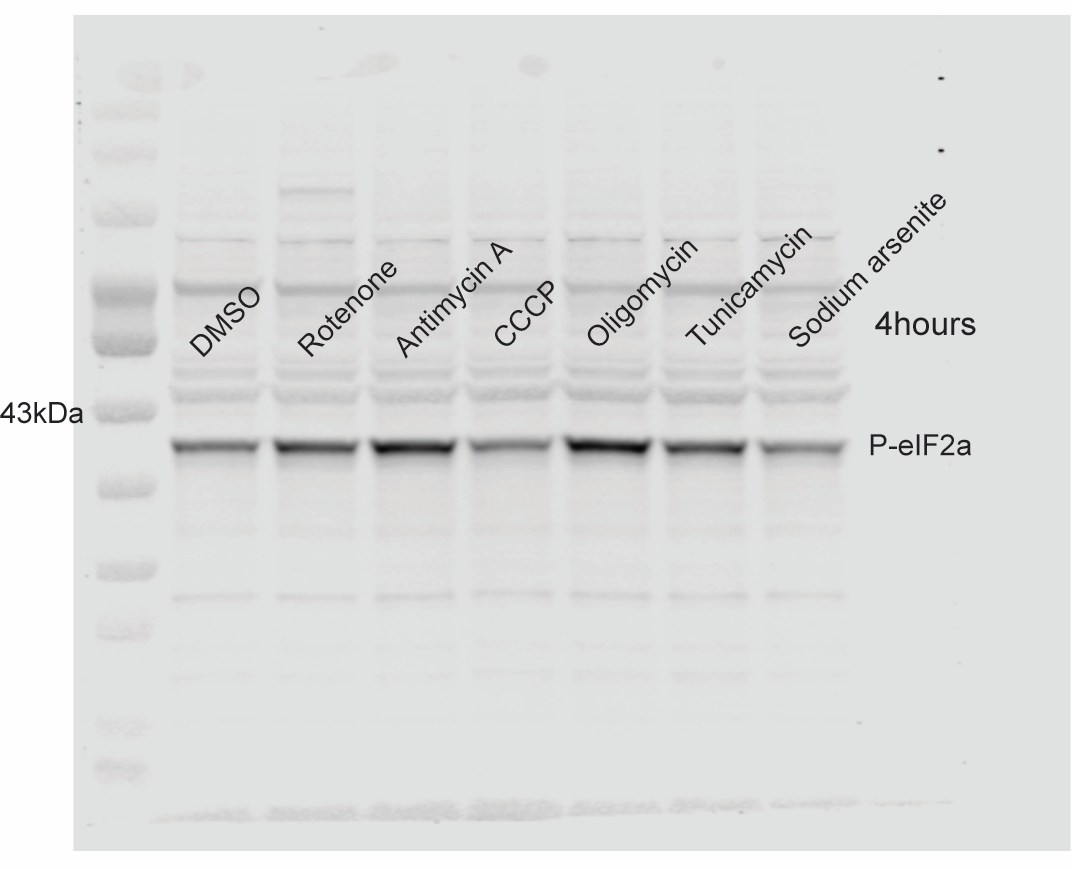

Supplement: Figure 1—figure supplement 1—source data 1. [file elife-102852-fig1-figsupp1-data1.zip › Figure 1-source data 2/Fig1Supplement1F_P-eIF2a_band_indicated.tif]

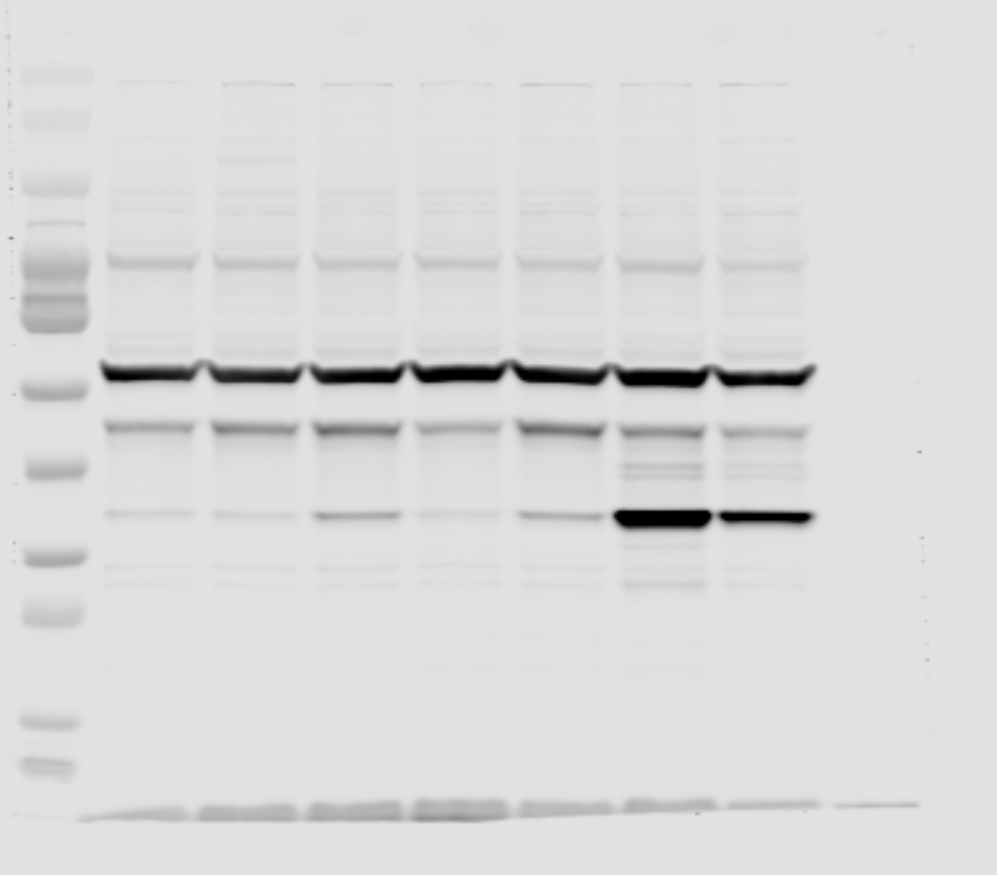

Supplement: Figure 1—figure supplement 1—source data 1. [file elife-102852-fig1-figsupp1-data1.zip › Figure 1-source data 2/Fig1Supplement1F_CHOP_original.tif]

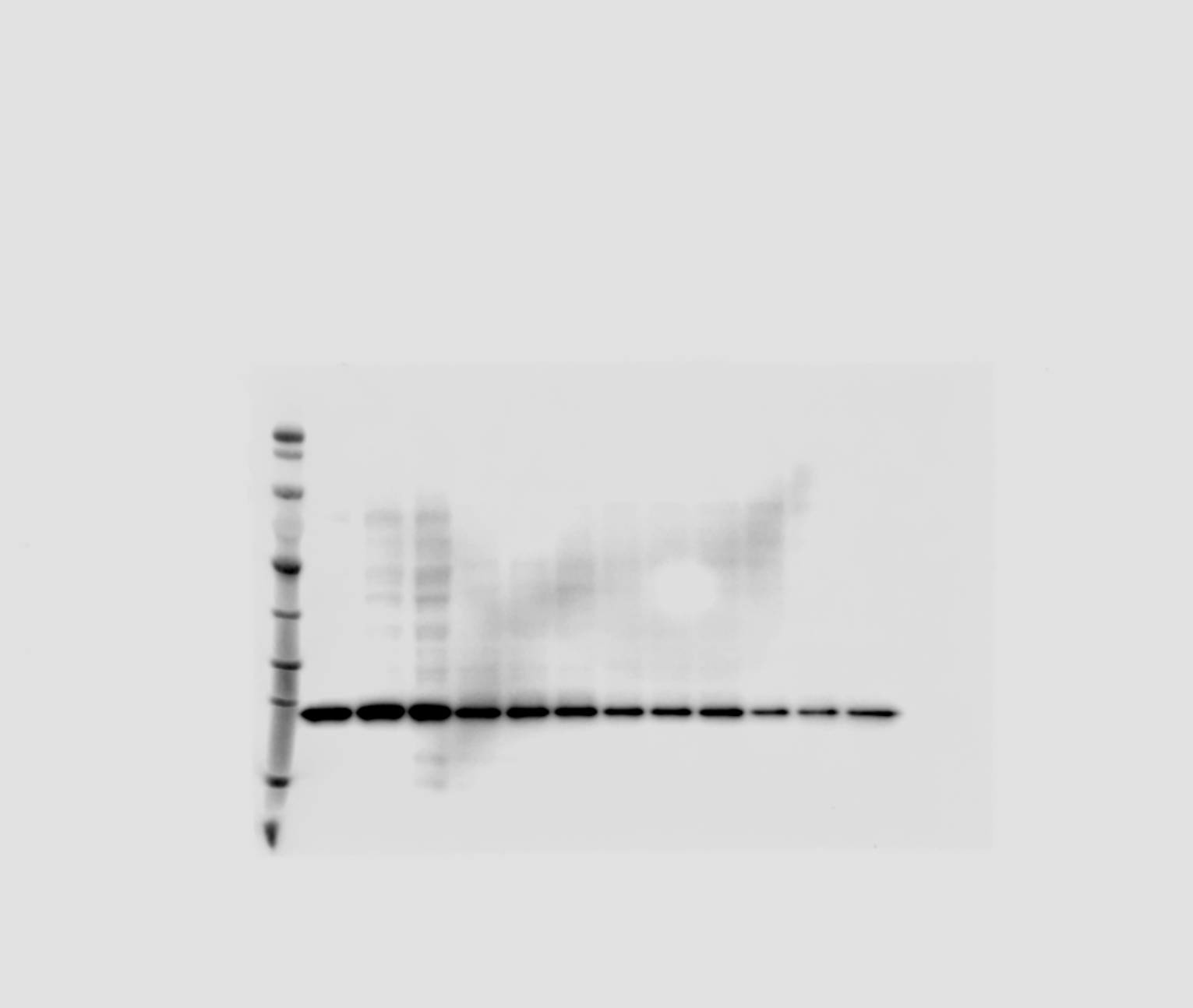

Supplement: Figure 1—figure supplement 1—source data 1. [file elife-102852-fig1-figsupp1-data1.zip › Figure 1-source data 2/Fig1Supplement1E_PEBP1_original.tif]

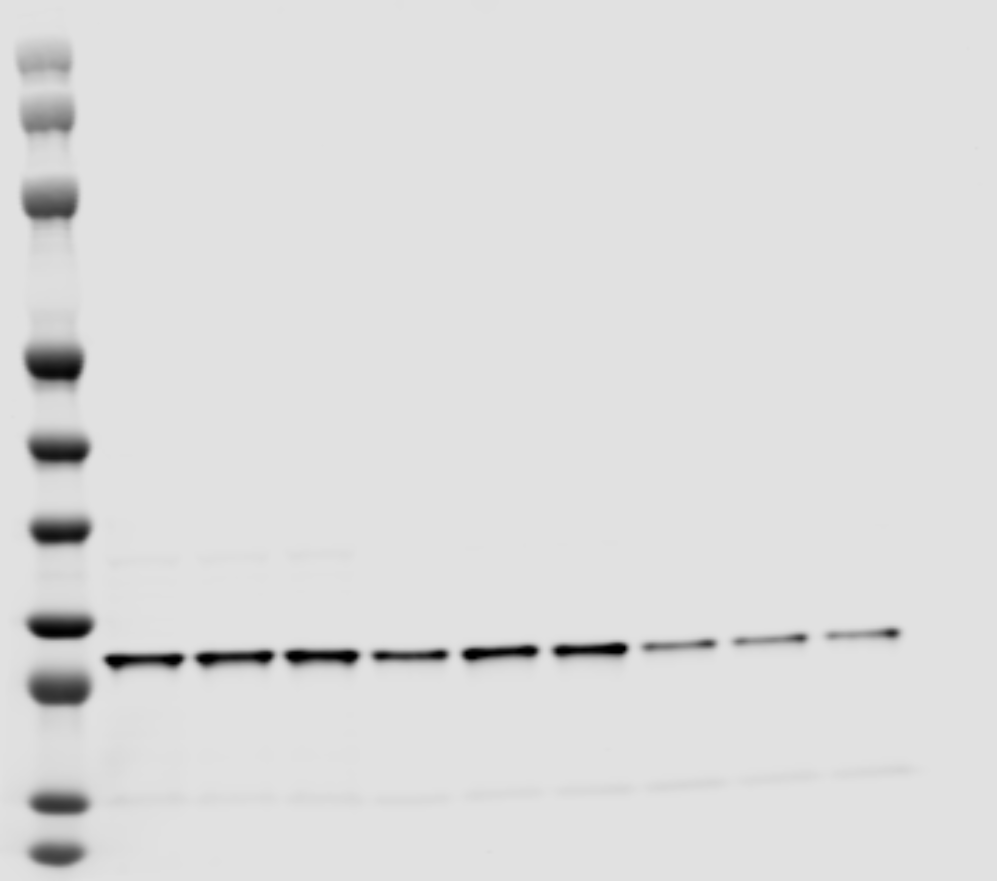

Supplement: Figure 1—figure supplement 1—source data 1. [file elife-102852-fig1-figsupp1-data1.zip › Figure 1-source data 2/Fig1Supplement1C_PEBP1_original.tif]

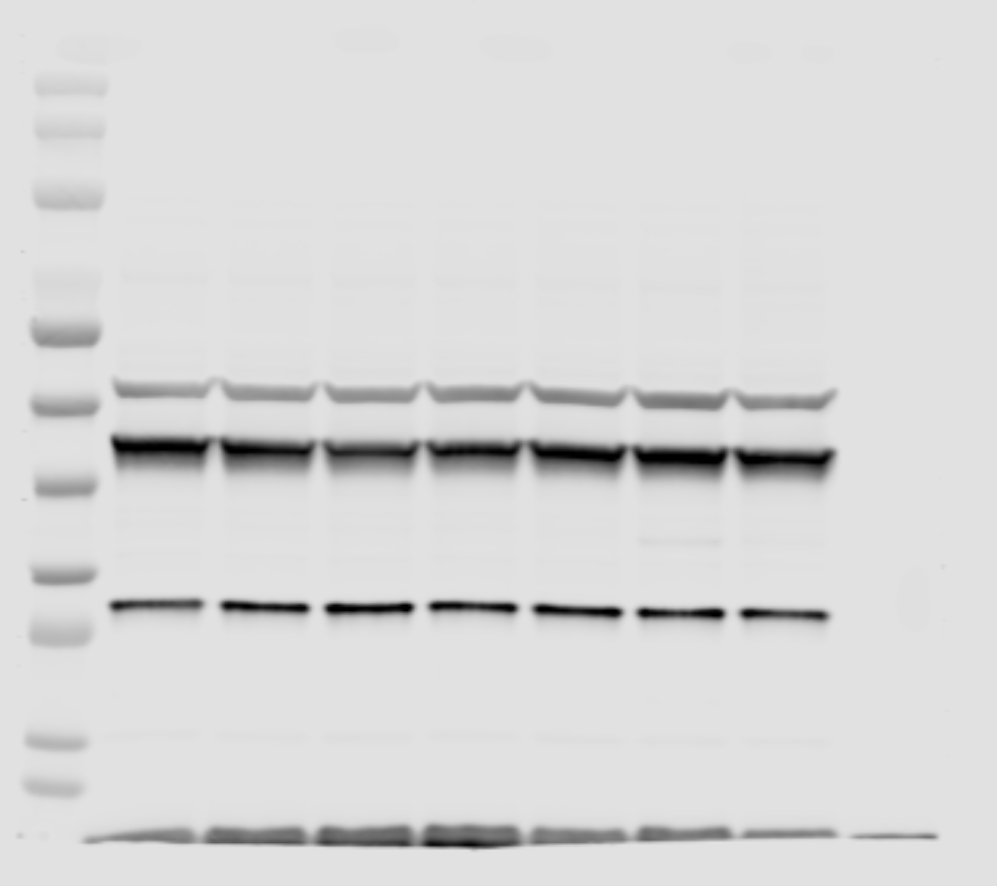

Supplement: Figure 1—figure supplement 1—source data 1. [file elife-102852-fig1-figsupp1-data1.zip › Figure 1-source data 2/Fig1Supplement1F_eIF2a_original.tif]

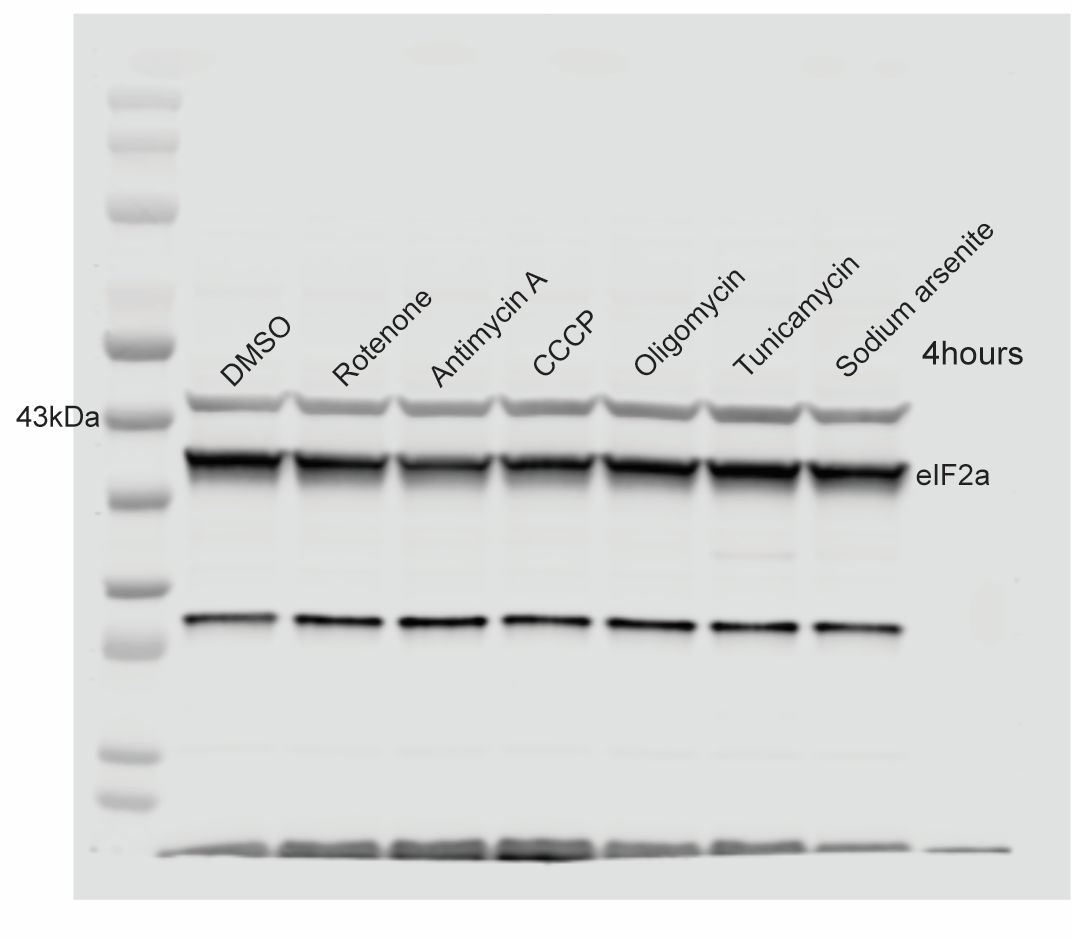

Supplement: Figure 1—figure supplement 1—source data 1. [file elife-102852-fig1-figsupp1-data1.zip › Figure 1-source data 2/Fig1Supplement1F_eIF2a_band_indicated.tif]

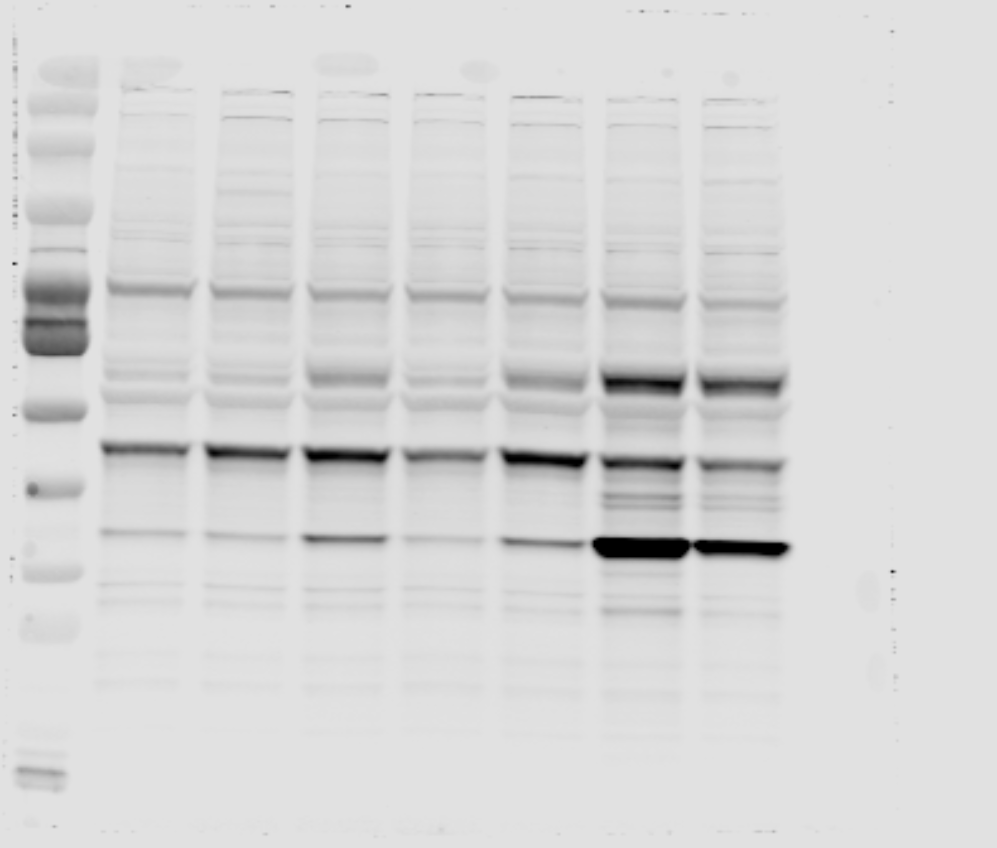

Supplement: Figure 1—figure supplement 1—source data 1. [file elife-102852-fig1-figsupp1-data1.zip › Figure 1-source data 2/Fig1Supplement1F_ATF4_original.tif]

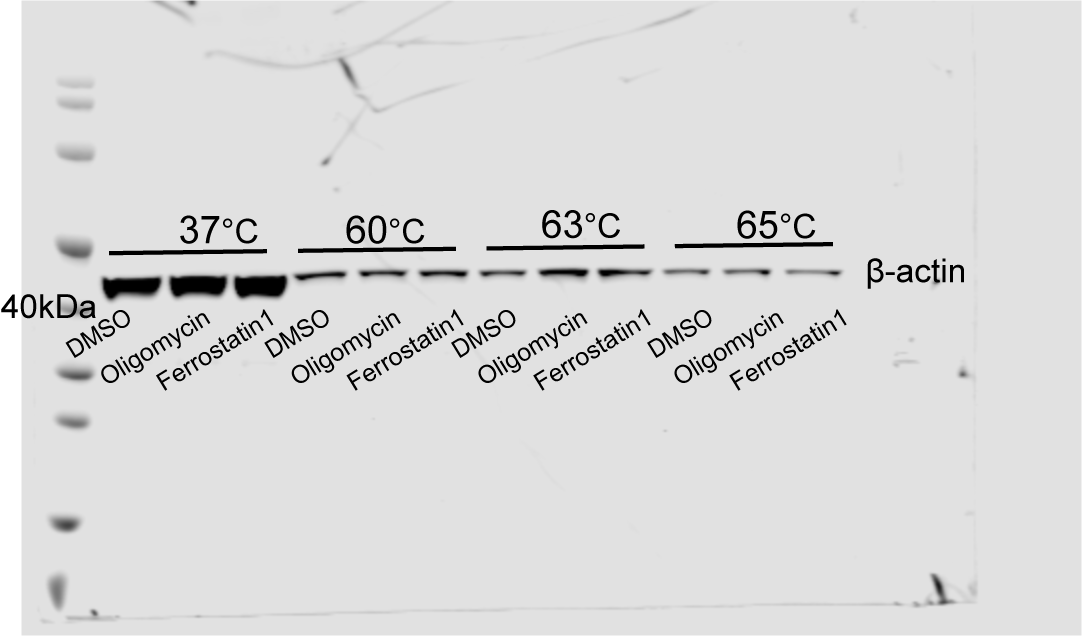

Supplement: Figure 1—figure supplement 1—source data 1. [file elife-102852-fig1-figsupp1-data1.zip › Figure 1-source data 2/Fig1Supplement1E_Actin_band_indicated.tif]

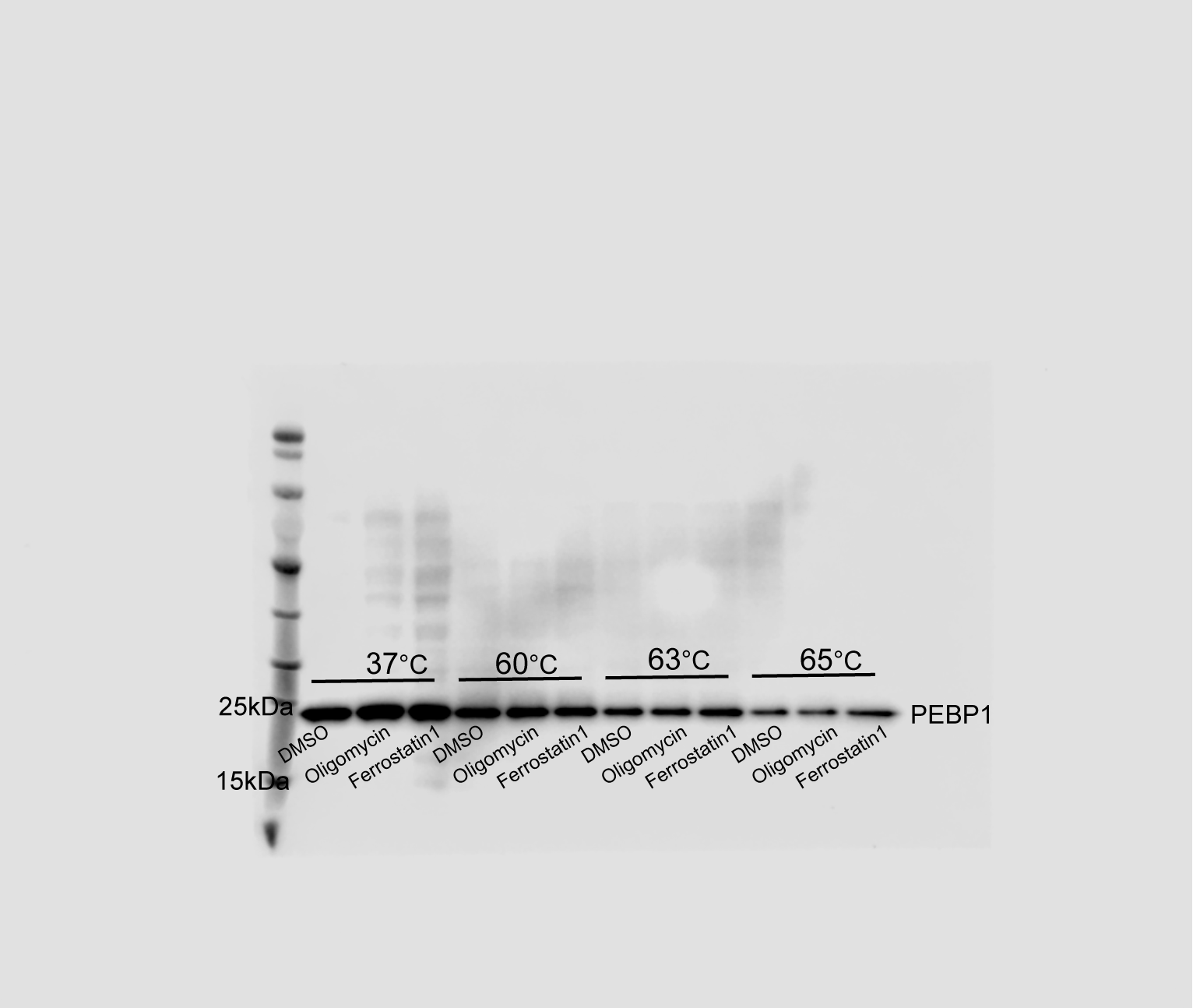

Supplement: Figure 1—figure supplement 1—source data 1. [file elife-102852-fig1-figsupp1-data1.zip › Figure 1-source data 2/Fig1Supplement1E_PEBP1_band_indicated.tif]

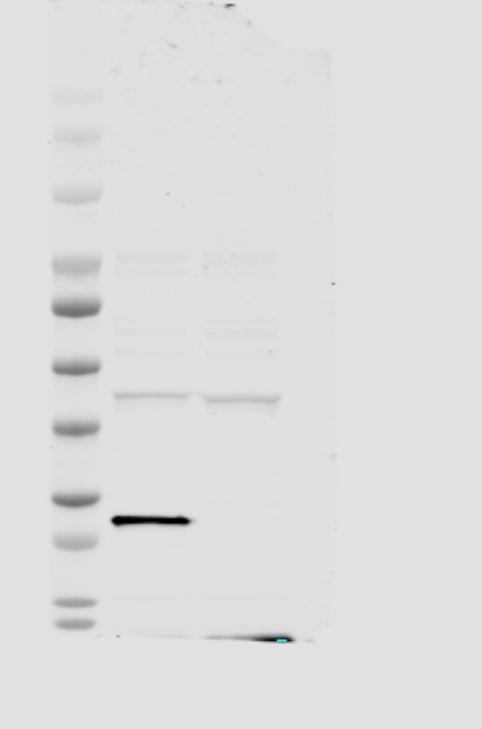

Supplement: Figure 2—source data 1. [file elife-102852-fig2-data1.zip › Figure 2-source data 1/Fig2A_PEBP1_original.tif]

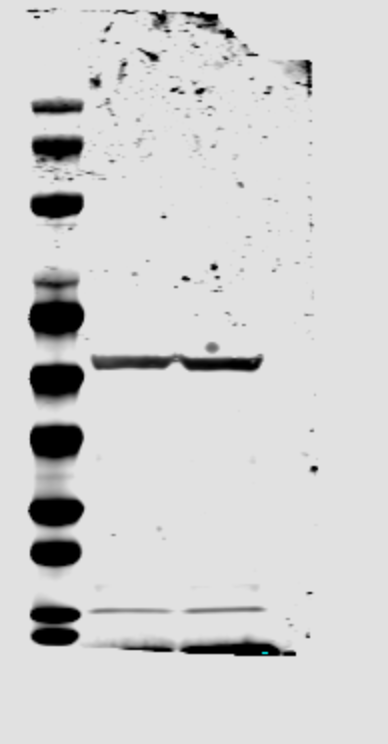

Supplement: Figure 2—source data 1. [file elife-102852-fig2-data1.zip › Figure 2-source data 1/Fig2A_Actin_original.tif]

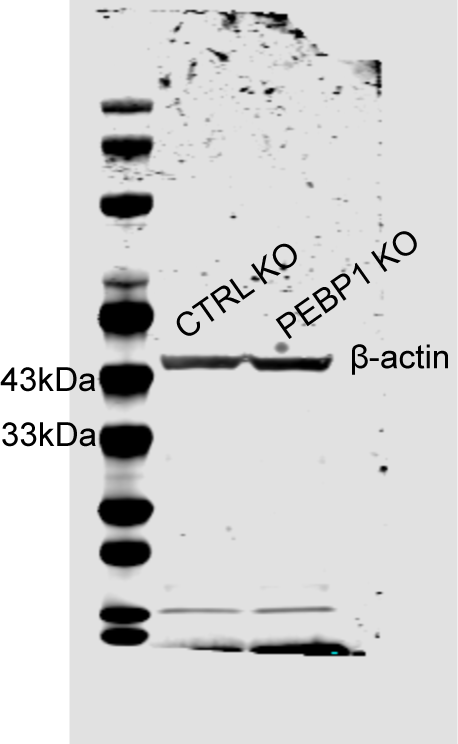

Supplement: Figure 2—source data 1. [file elife-102852-fig2-data1.zip › Figure 2-source data 1/Fig2A_Actin_band_indicated.tif]

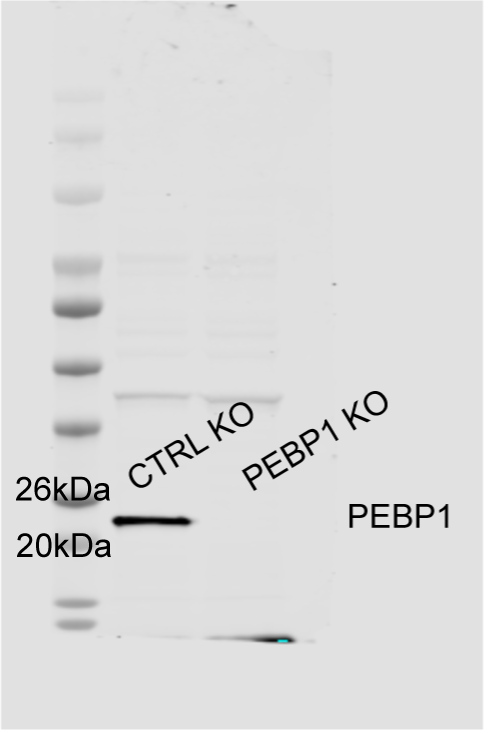

Supplement: Figure 2—source data 1. [file elife-102852-fig2-data1.zip › Figure 2-source data 1/Fig2A_PEBP1_band_indicated.tif]

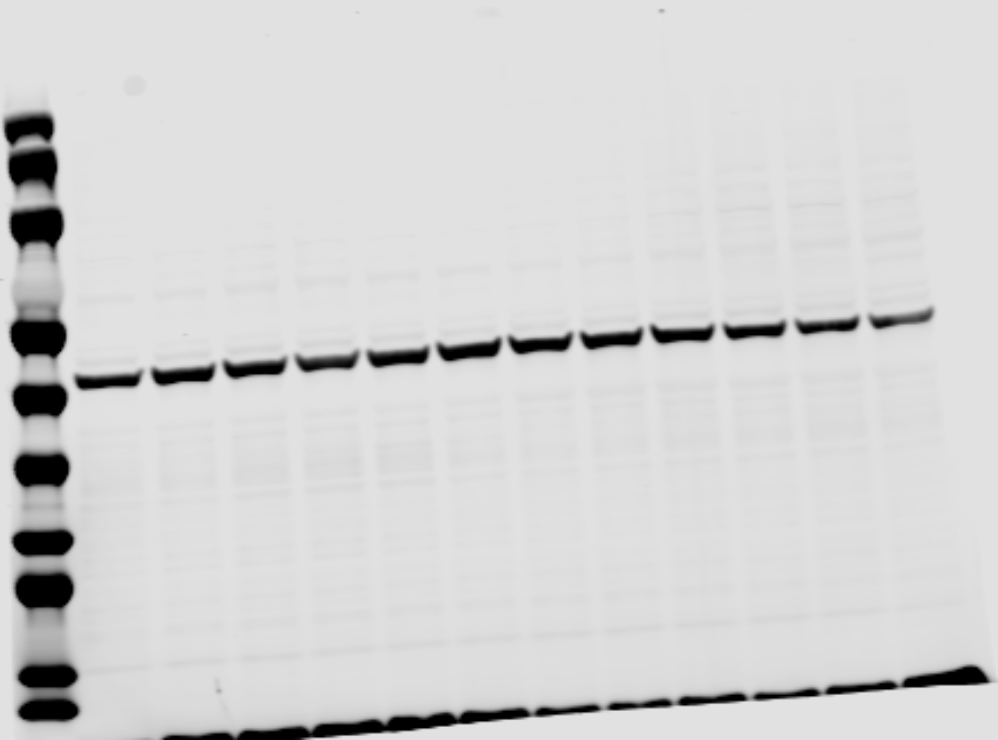

Supplement: Figure 3—source data 1. [file elife-102852-fig3-data1.zip › Figure 3-source data 1/Fig3E_Actin_original.tif]

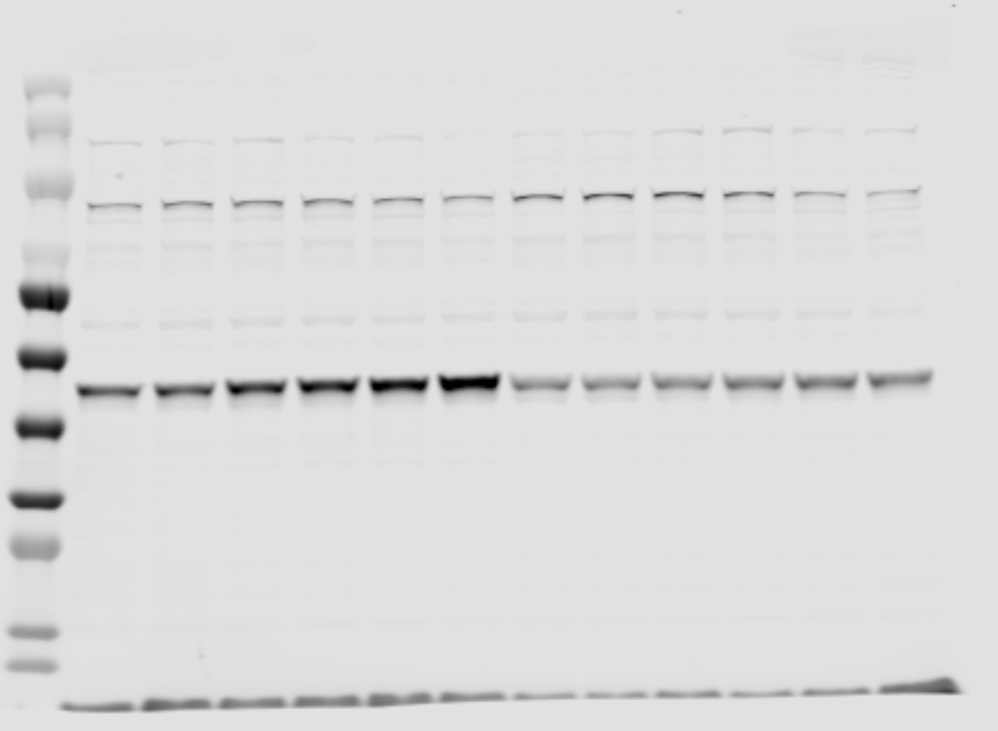

Supplement: Figure 3—source data 1. [file elife-102852-fig3-data1.zip › Figure 3-source data 1/Fig3E_P-eIF2a_original.tif]

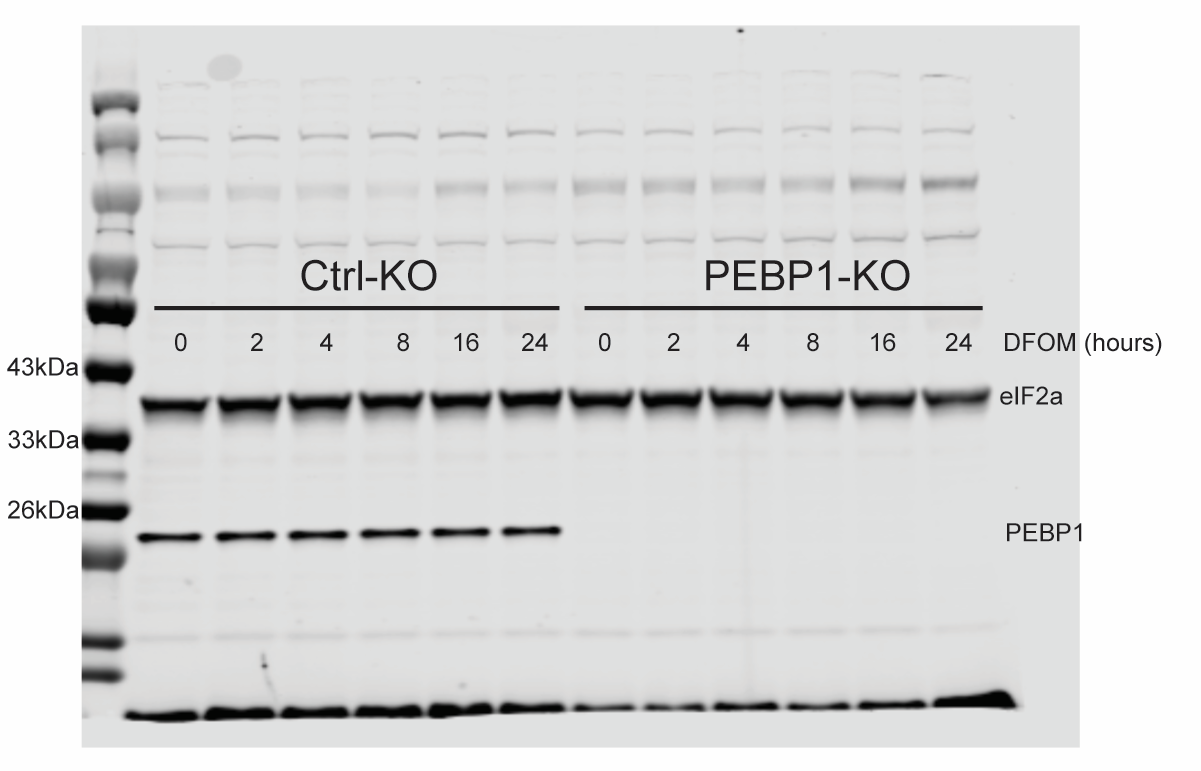

Supplement: Figure 3—source data 1. [file elife-102852-fig3-data1.zip › Figure 3-source data 1/Fig3E_eIF2a_PEBP1_bands_indicated.tif]

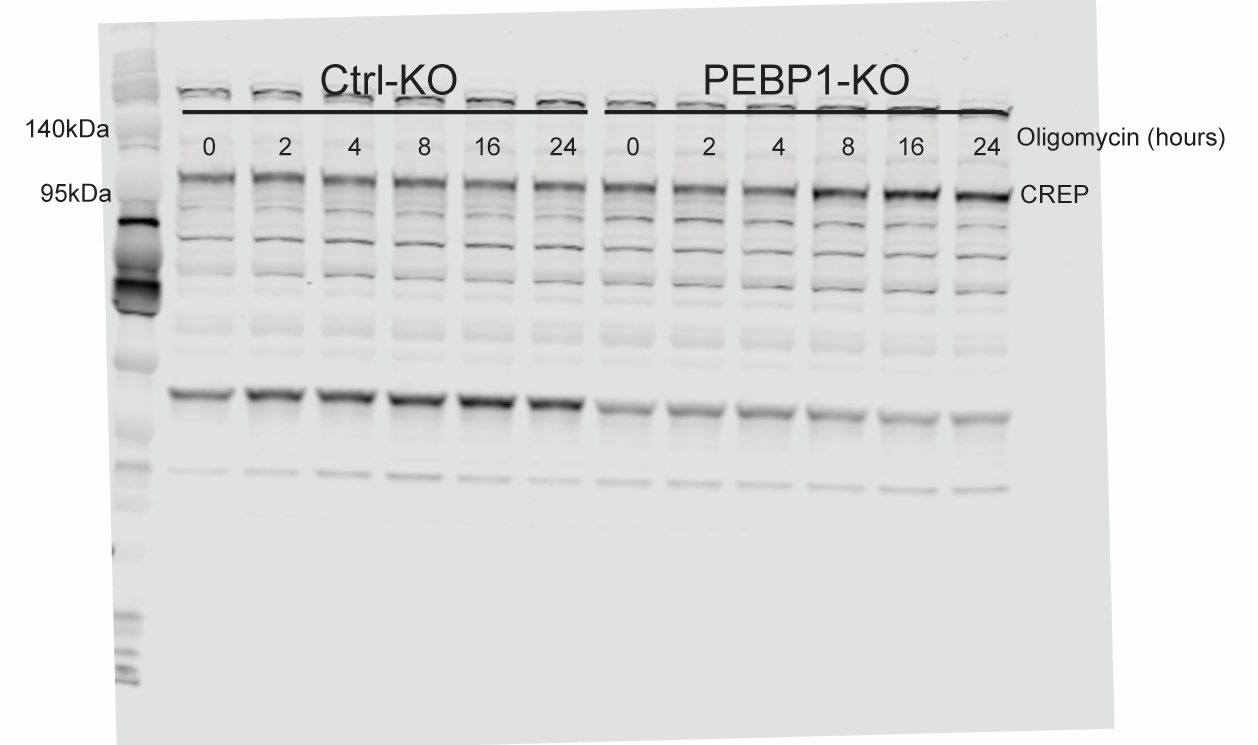

Supplement: Figure 3—source data 1. [file elife-102852-fig3-data1.zip › Figure 3-source data 1/Fig3D_CREP_band_indicated.tif]

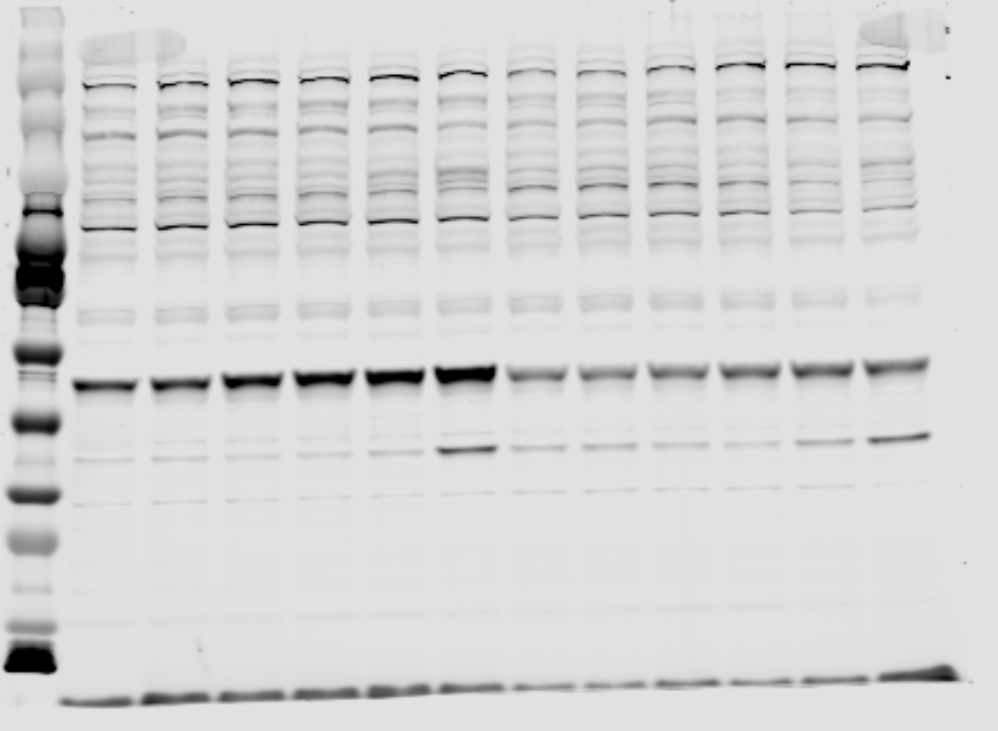

Supplement: Figure 3—source data 1. [file elife-102852-fig3-data1.zip › Figure 3-source data 1/Fig3E_CHOP_original.tif]

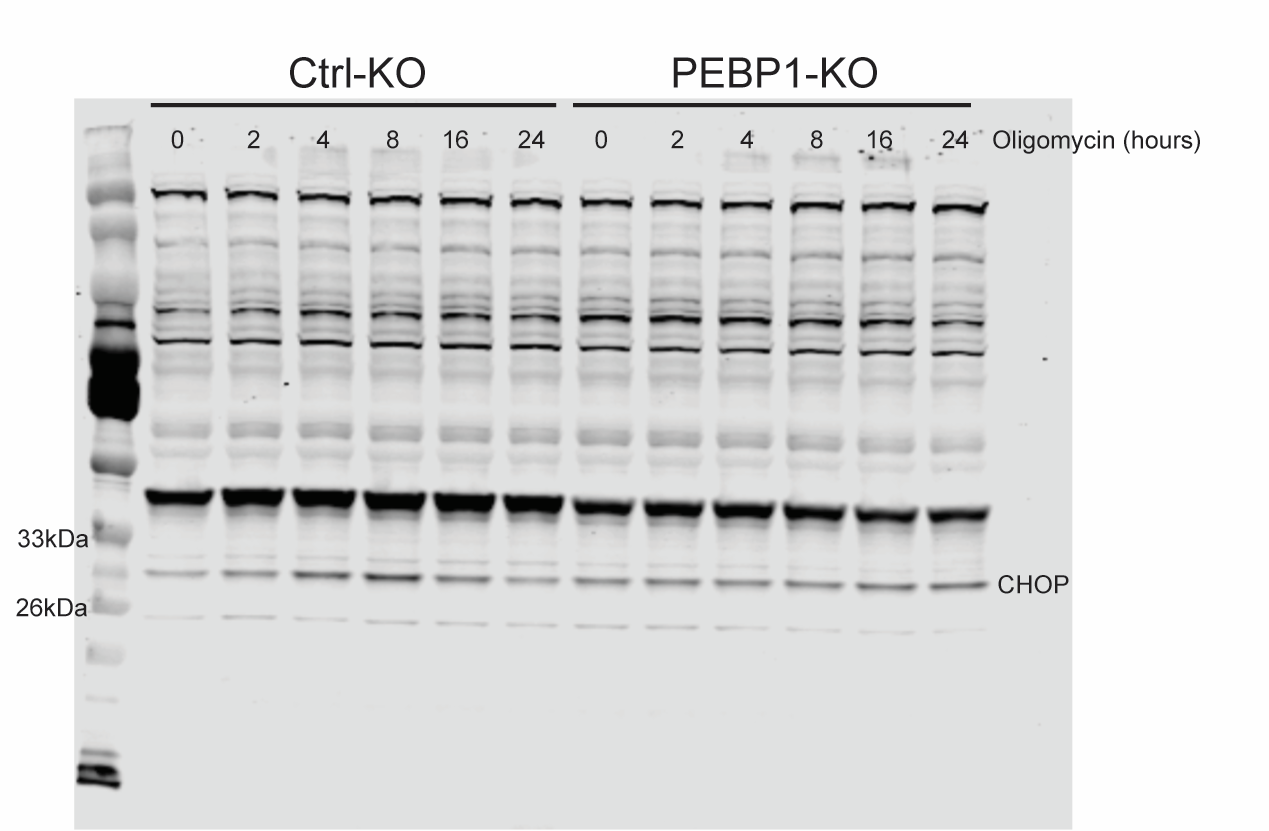

Supplement: Figure 3—source data 1. [file elife-102852-fig3-data1.zip › Figure 3-source data 1/Fig3D_CHOP_band_indicated.tif]

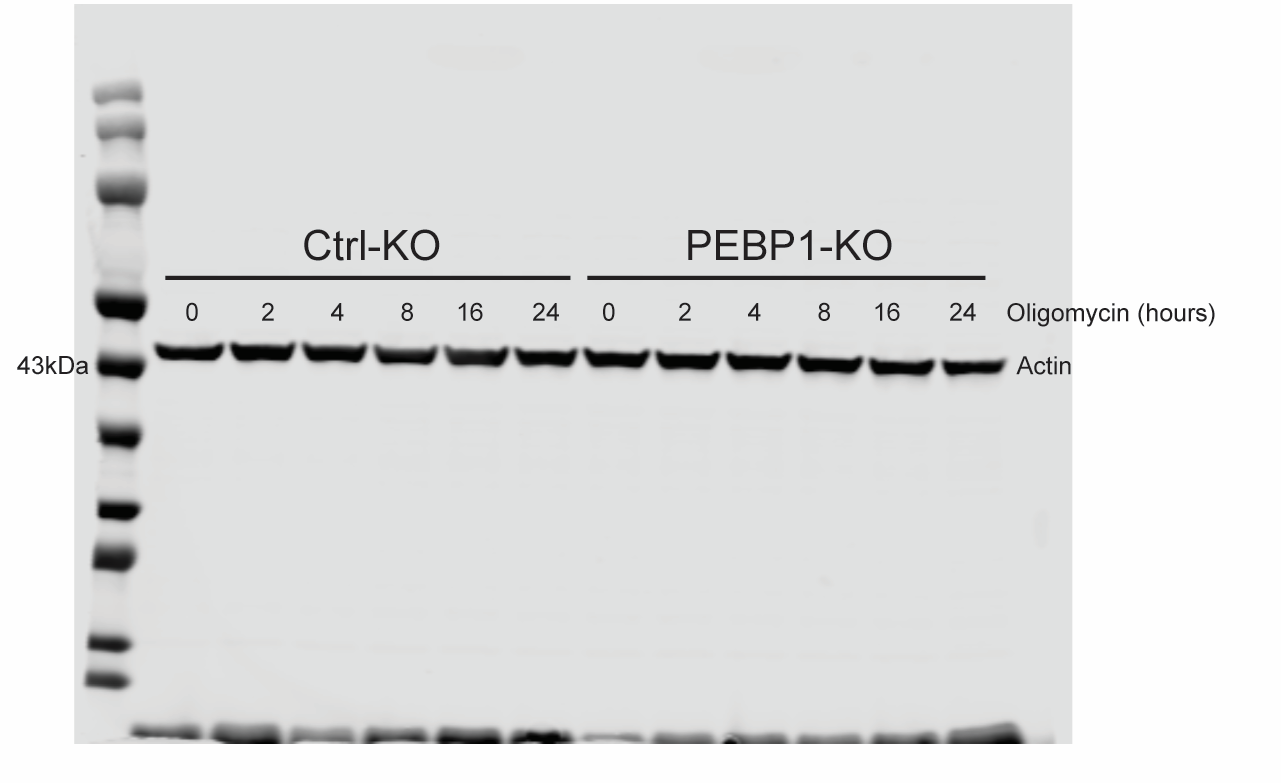

Supplement: Figure 3—source data 1. [file elife-102852-fig3-data1.zip › Figure 3-source data 1/Fig3D_Actin_band_indicated.tif]

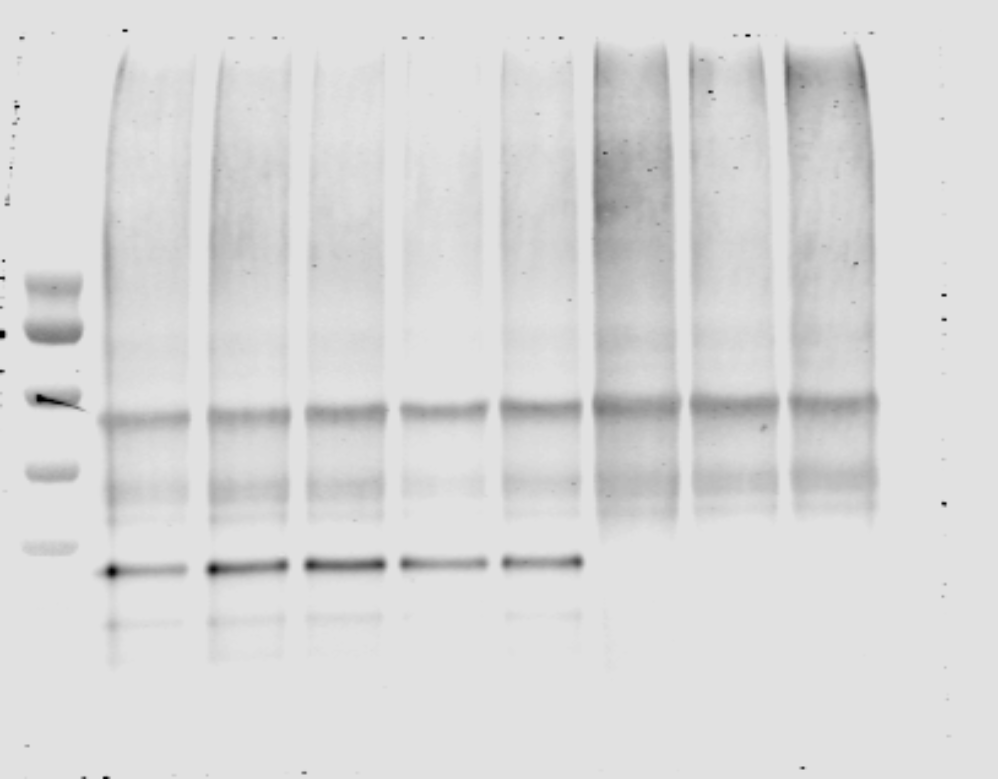

Supplement: Figure 3—source data 1. [file elife-102852-fig3-data1.zip › Figure 3-source data 1/Fig3C_GAPDH_PEBP1_original.tif.tif]

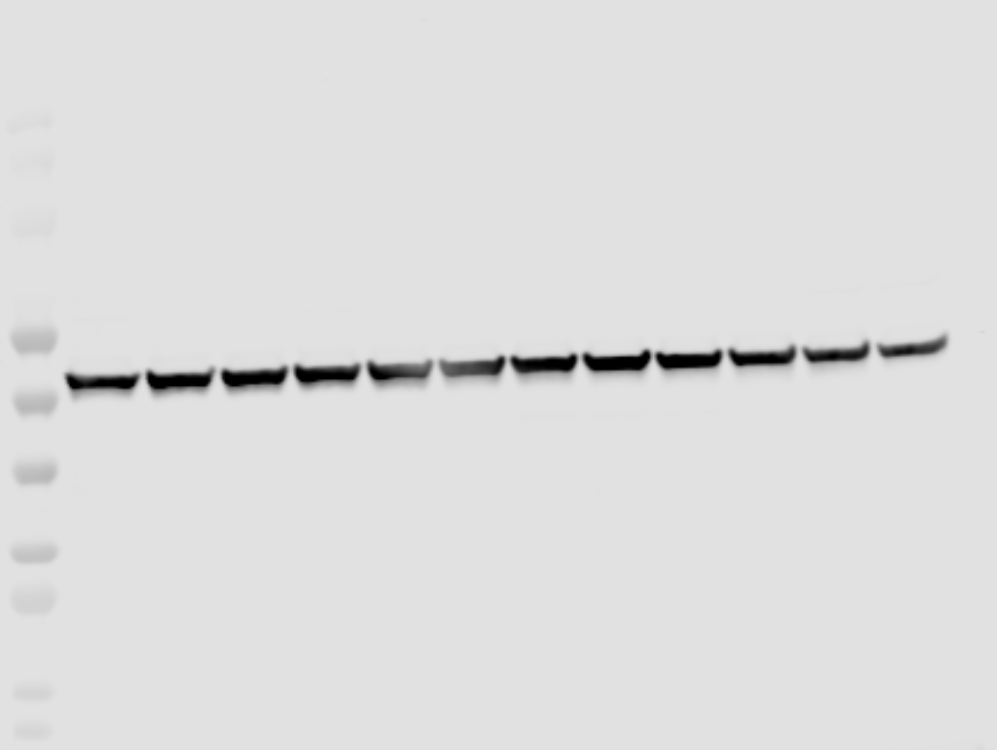

Supplement: Figure 3—source data 1. [file elife-102852-fig3-data1.zip › Figure 3-source data 1/Fig3F_Actin_original.tif]

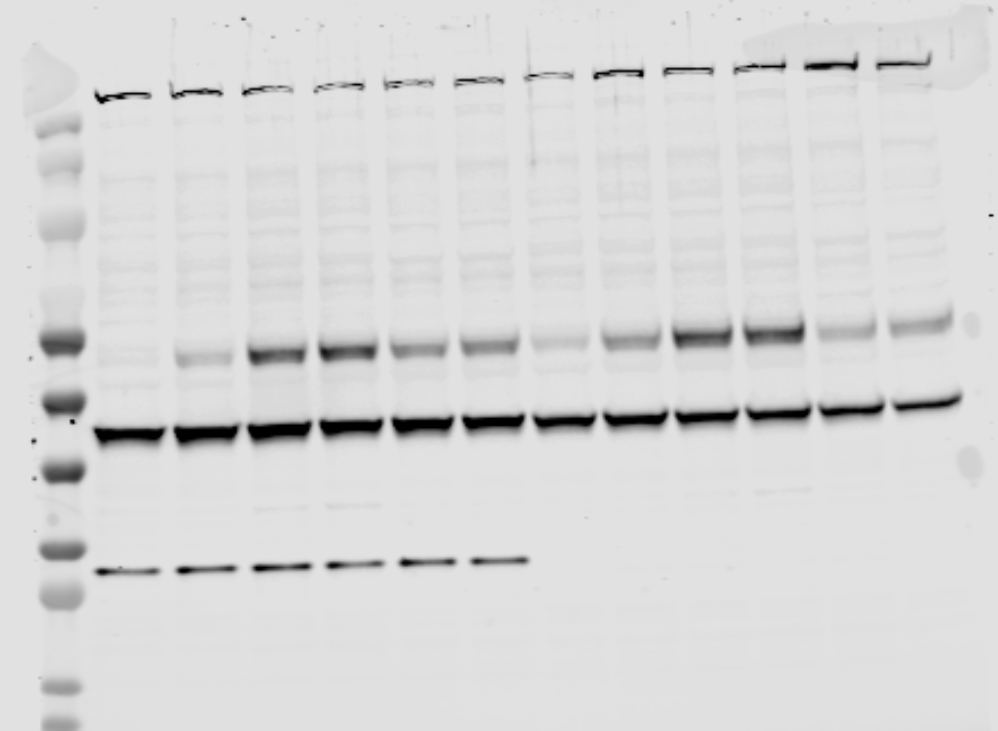

Supplement: Figure 3—source data 1. [file elife-102852-fig3-data1.zip › Figure 3-source data 1/Fig3F_ATF4_original.tif]

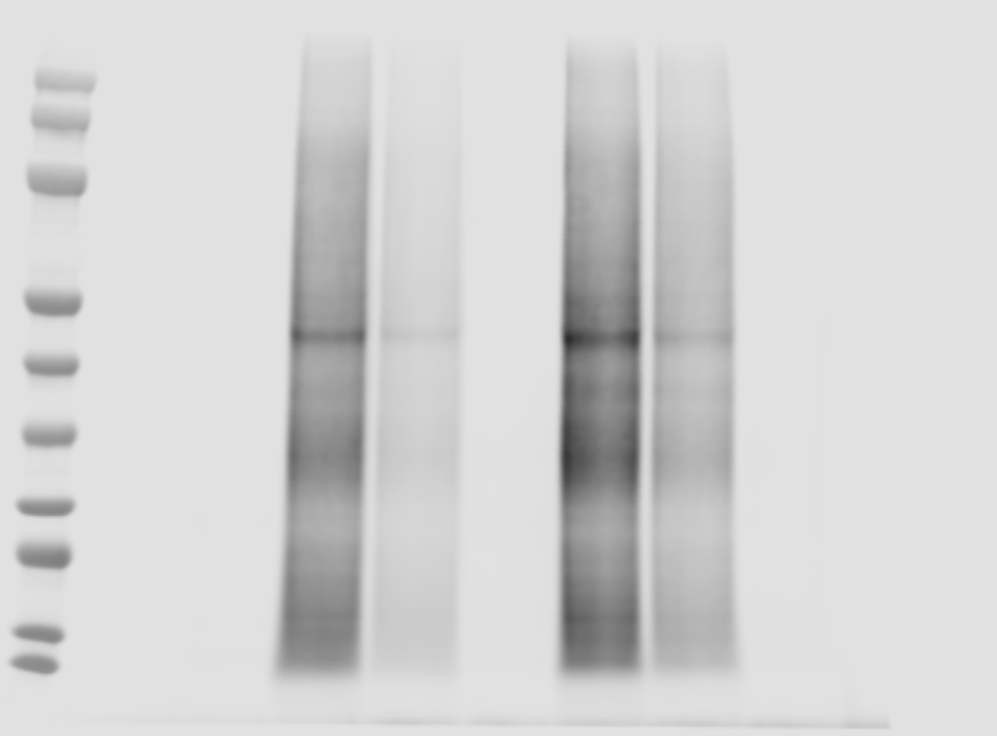

Supplement: Figure 3—source data 1. [file elife-102852-fig3-data1.zip › Figure 3-source data 1/Fig3C_HPG_original.tif]

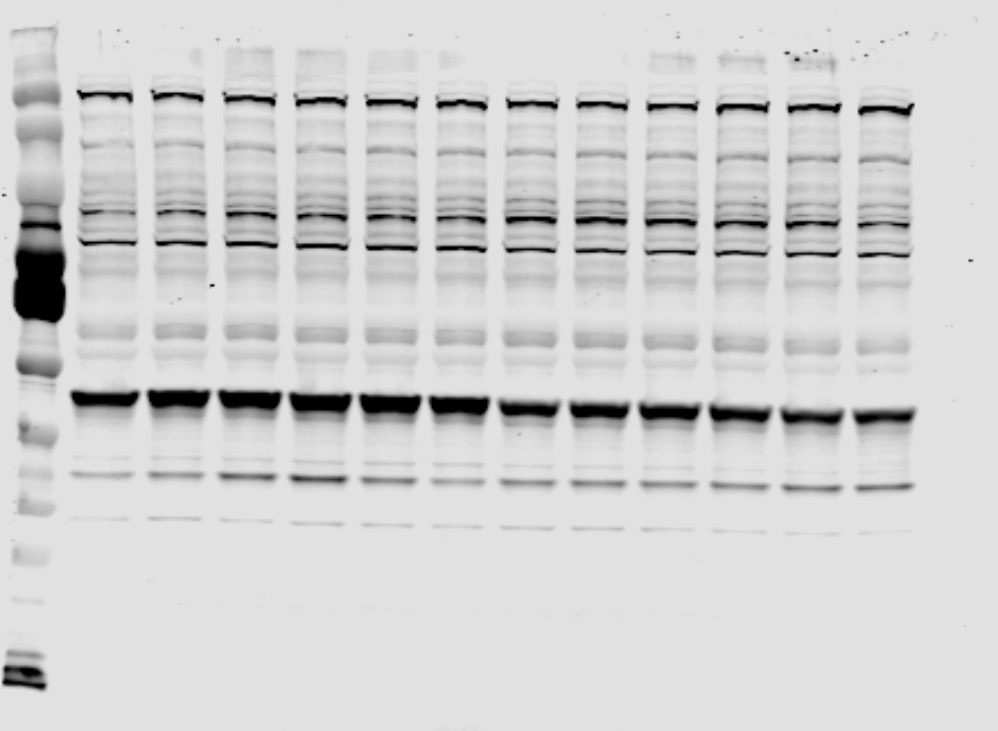

Supplement: Figure 3—source data 1. [file elife-102852-fig3-data1.zip › Figure 3-source data 1/Fig3D_CHOP_original.tif]

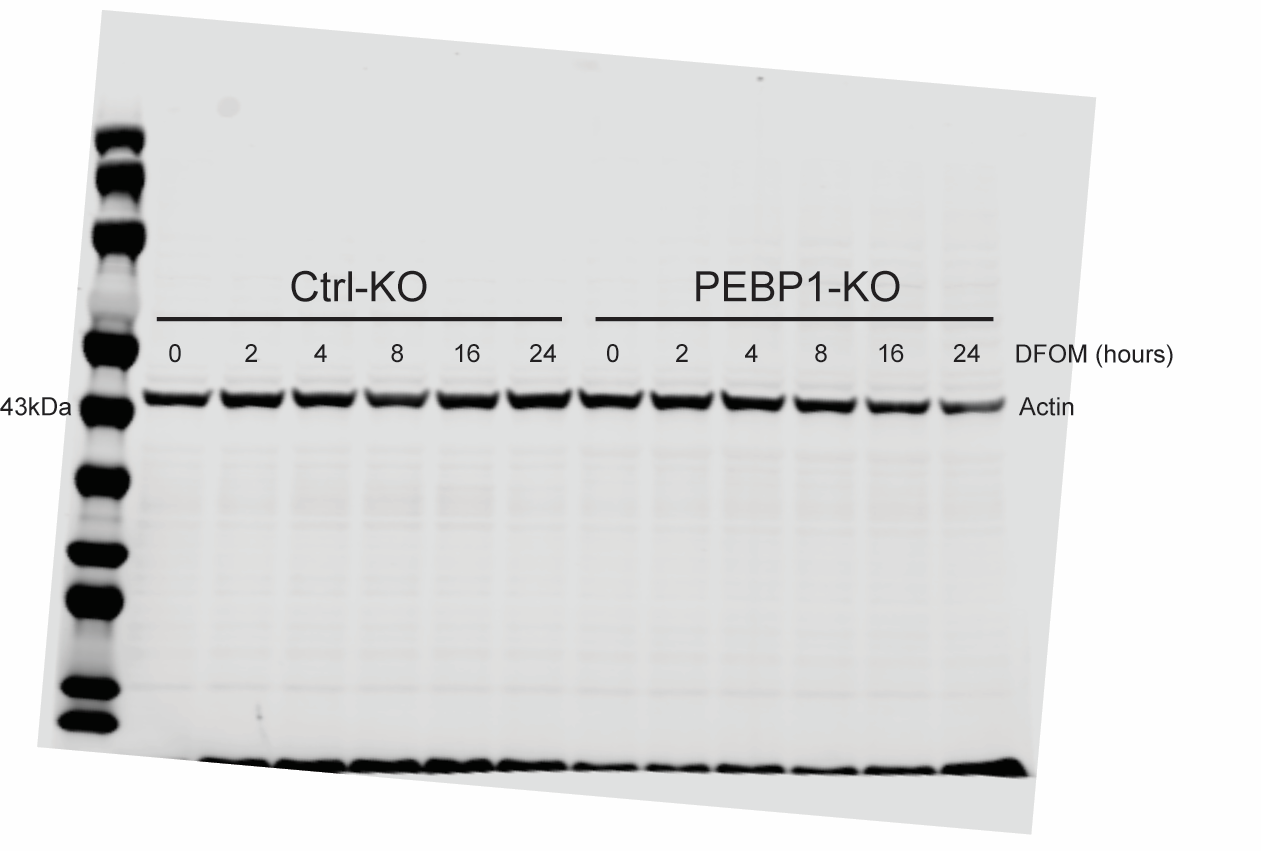

Supplement: Figure 3—source data 1. [file elife-102852-fig3-data1.zip › Figure 3-source data 1/Fig3E_Actin_band_indicated.tif]

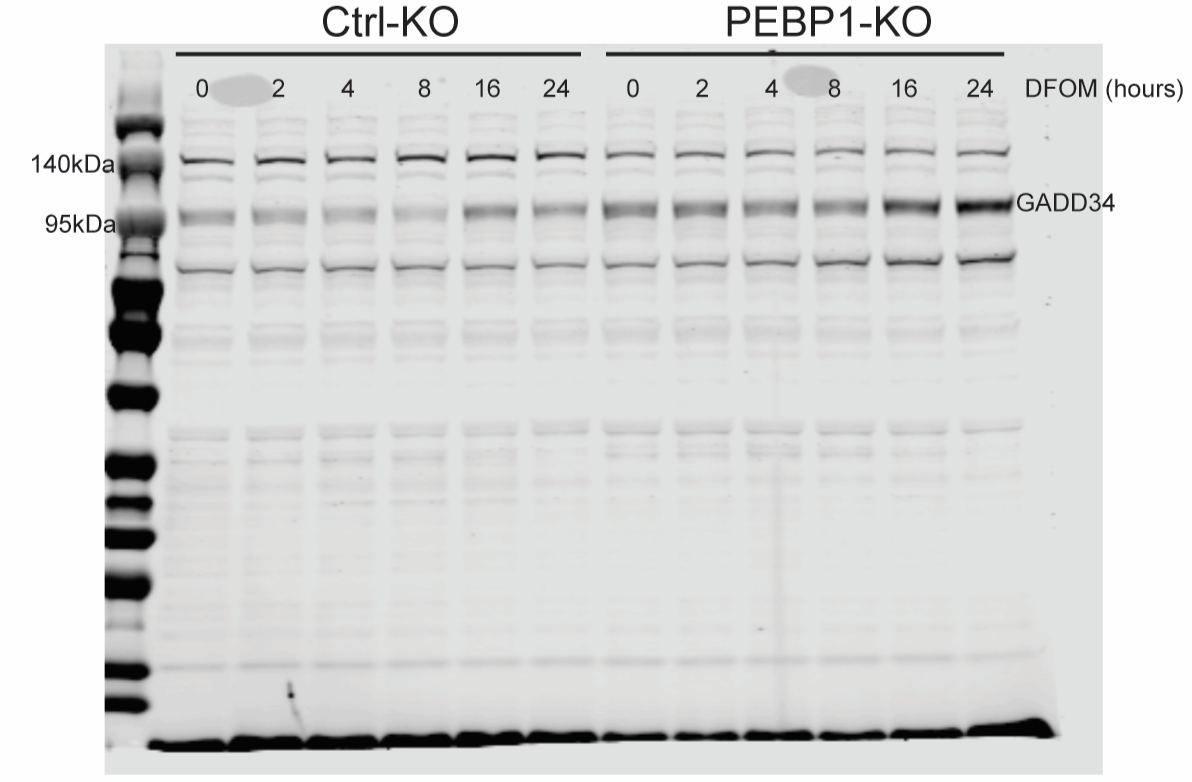

Supplement: Figure 3—source data 1. [file elife-102852-fig3-data1.zip › Figure 3-source data 1/Fig3E_GADD34_band_indicated.tif]

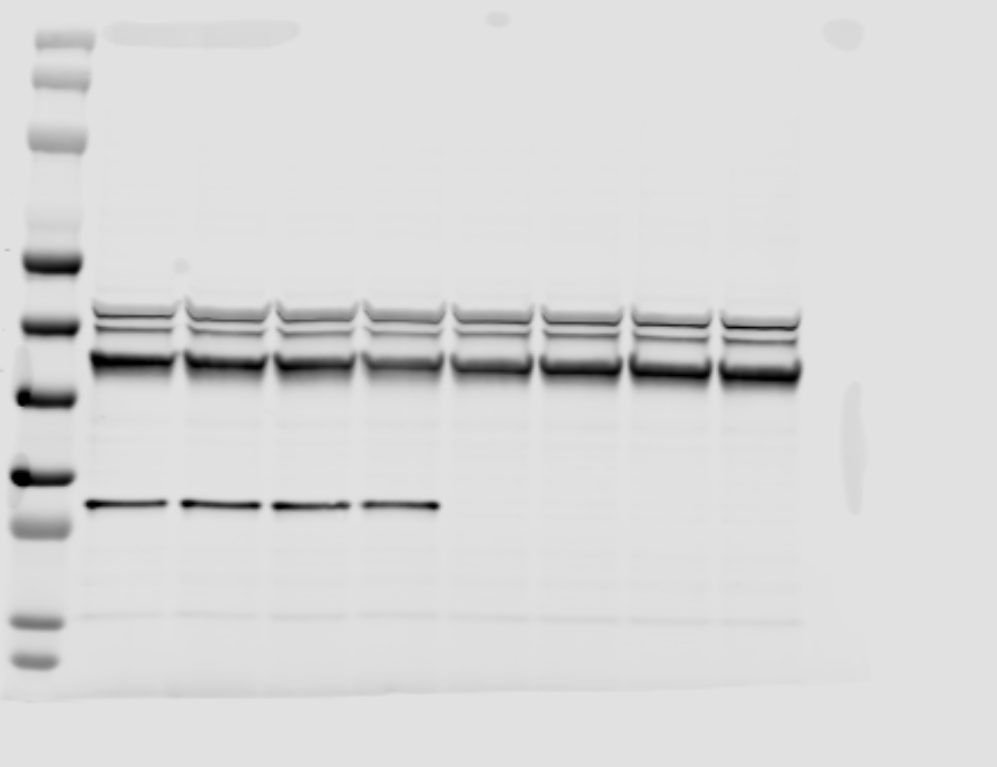

Supplement: Figure 3—source data 1. [file elife-102852-fig3-data1.zip › Figure 3-source data 1/Fig3A_ERK_original.tif]

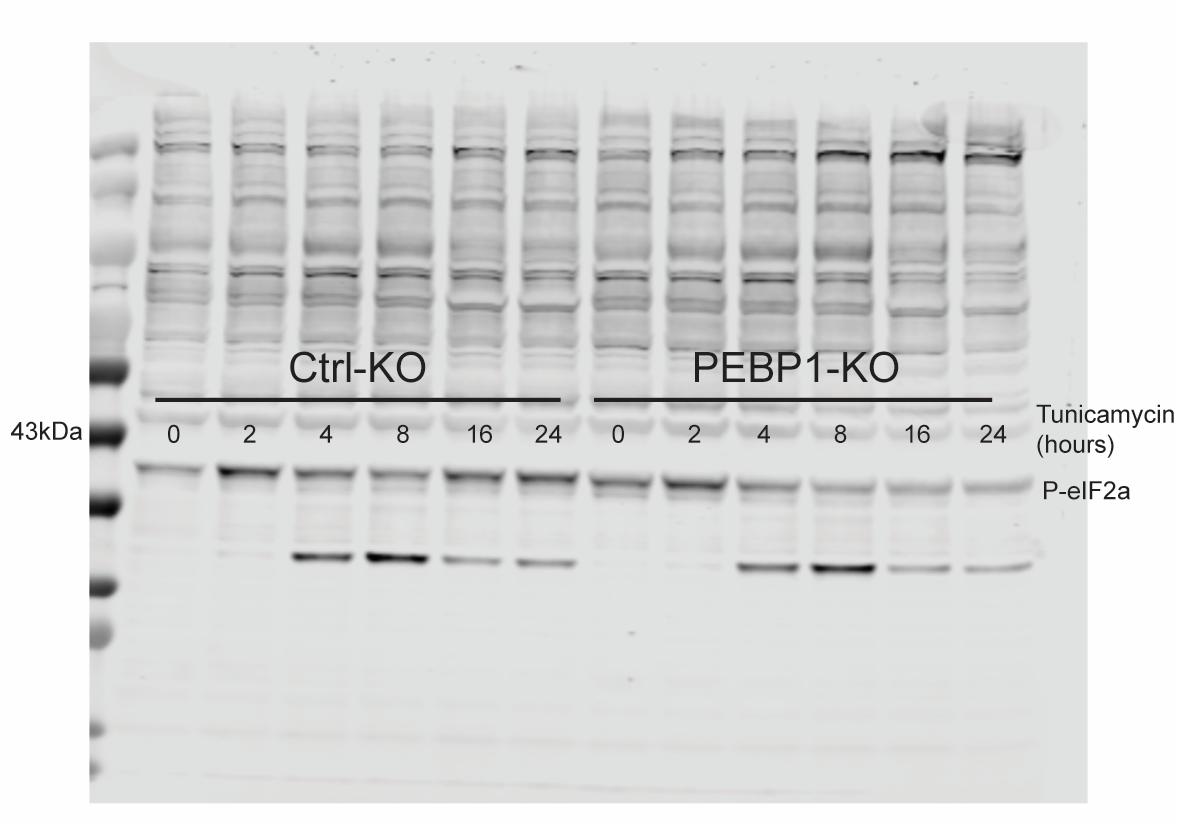

Supplement: Figure 3—source data 1. [file elife-102852-fig3-data1.zip › Figure 3-source data 1/Fig3F_P-eIF2a_band_indicated.tif]

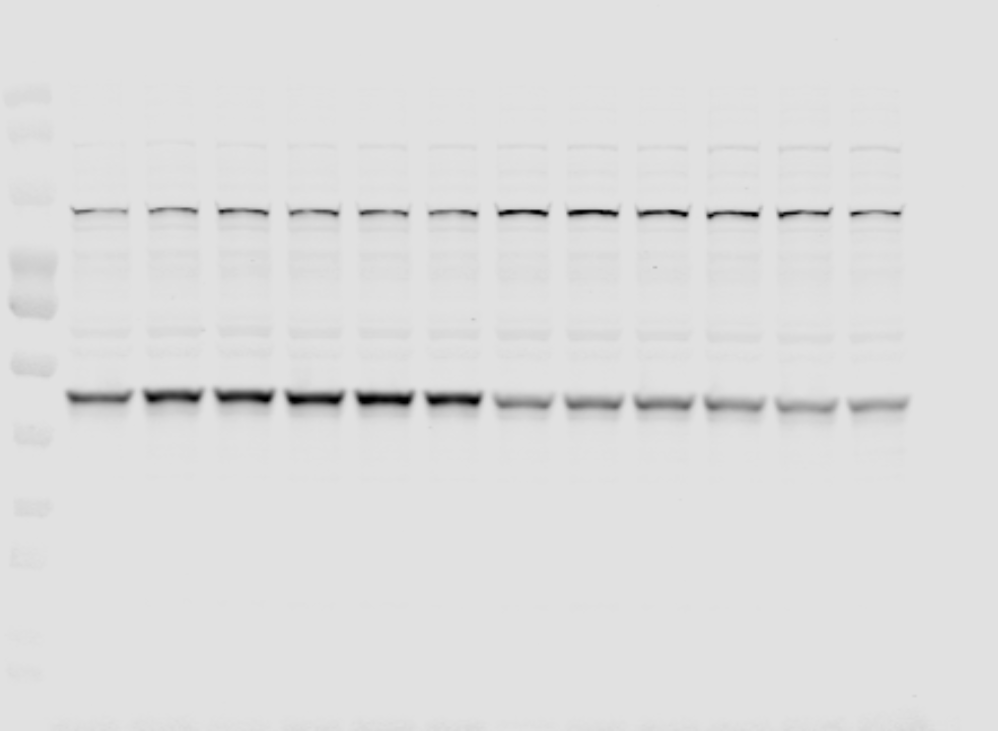

Supplement: Figure 3—source data 1. [file elife-102852-fig3-data1.zip › Figure 3-source data 1/Fig3D_P-eIF2a_original.tif]

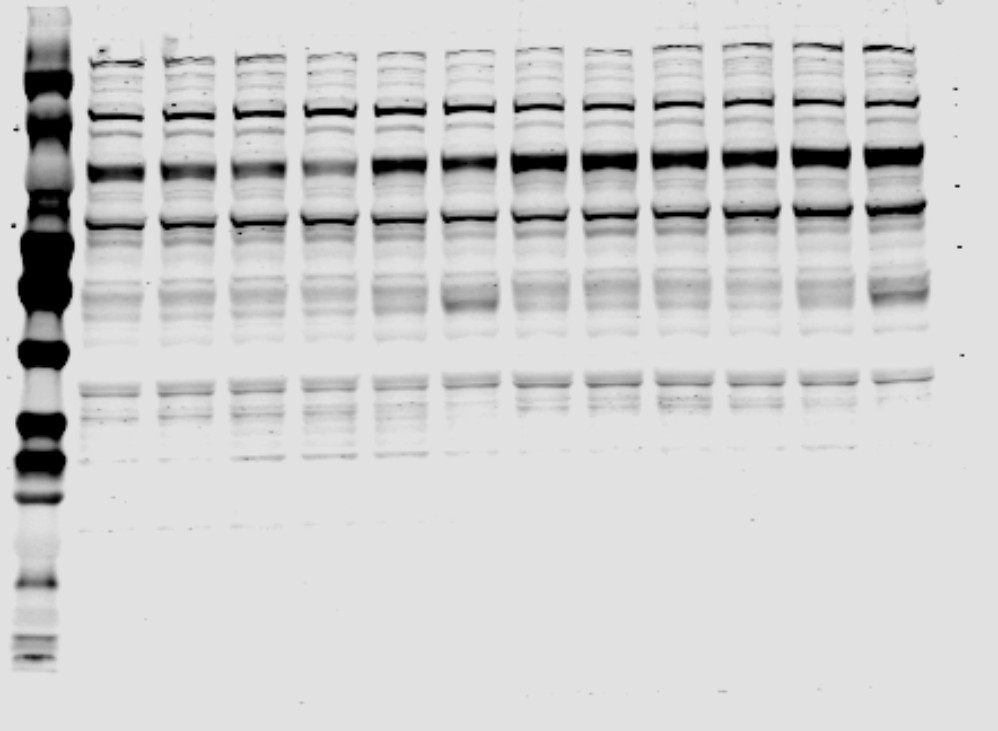

Supplement: Figure 3—source data 1. [file elife-102852-fig3-data1.zip › Figure 3-source data 1/Fig3E_ATF4_original.tif]

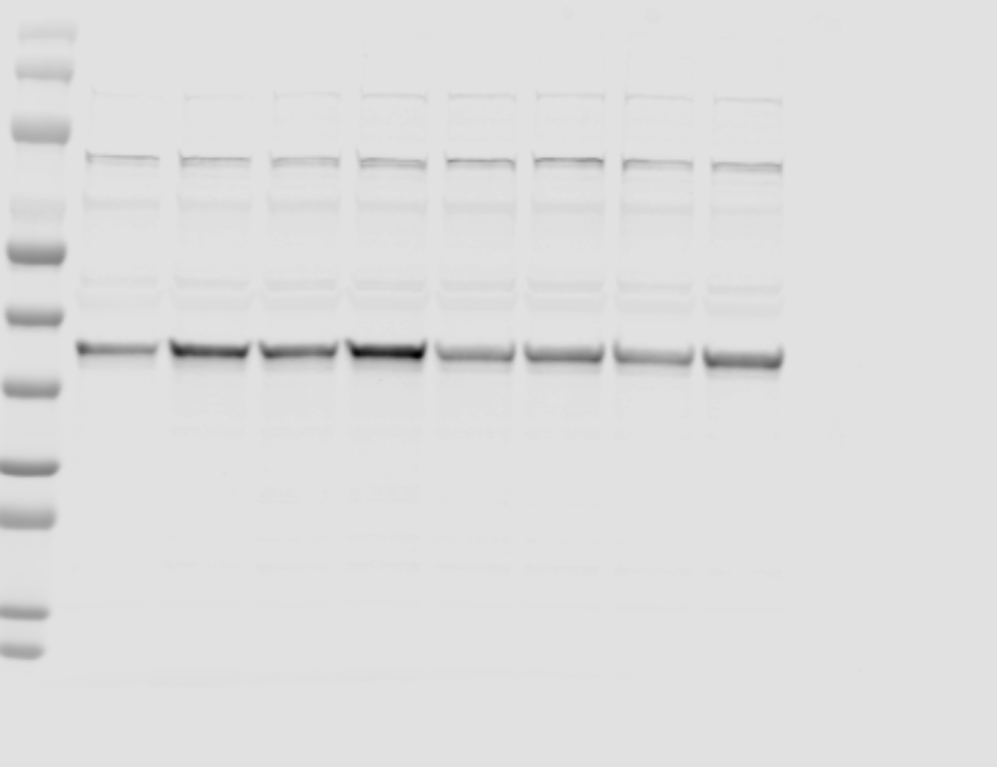

Supplement: Figure 3—source data 1. [file elife-102852-fig3-data1.zip › Figure 3-source data 1/Fig3A_P-eIF2a_original.tif]

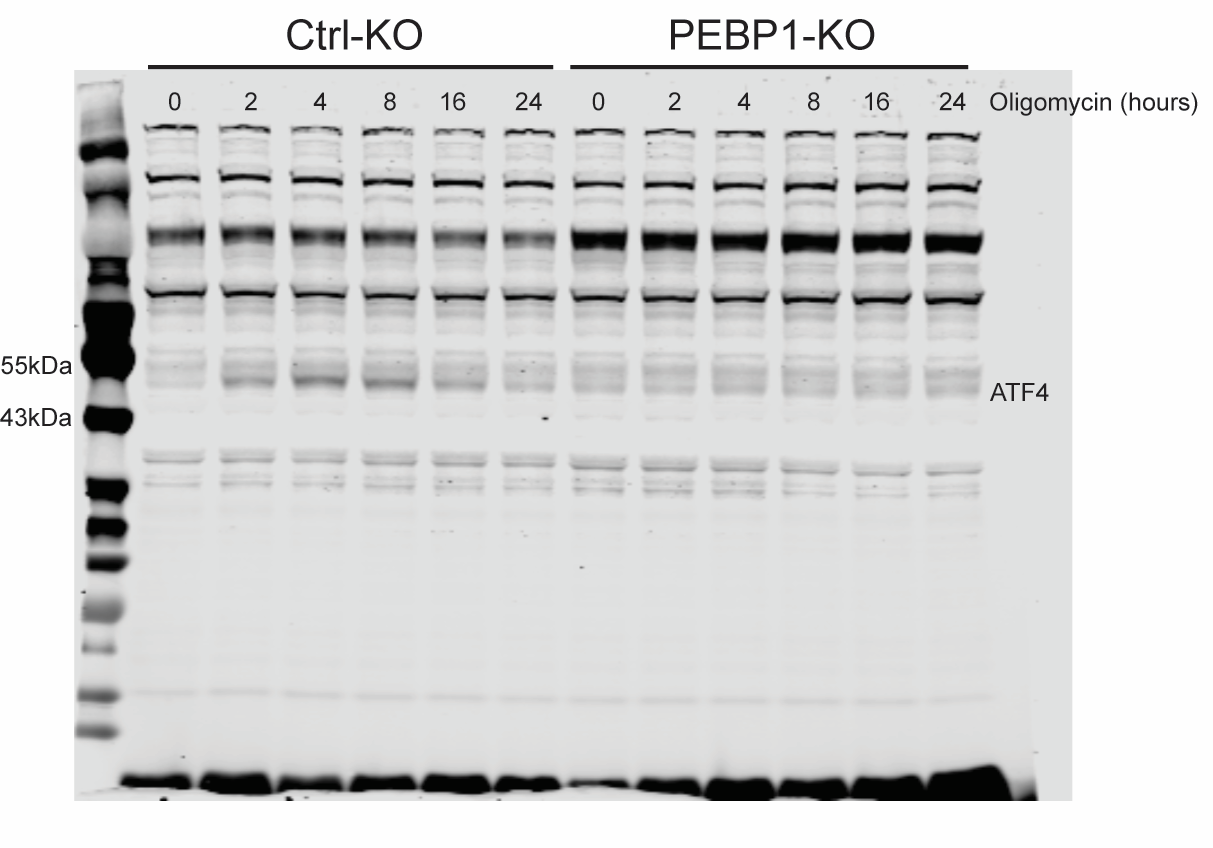

Supplement: Figure 3—source data 1. [file elife-102852-fig3-data1.zip › Figure 3-source data 1/Fig3D_ATF4_band_indicated.tif]

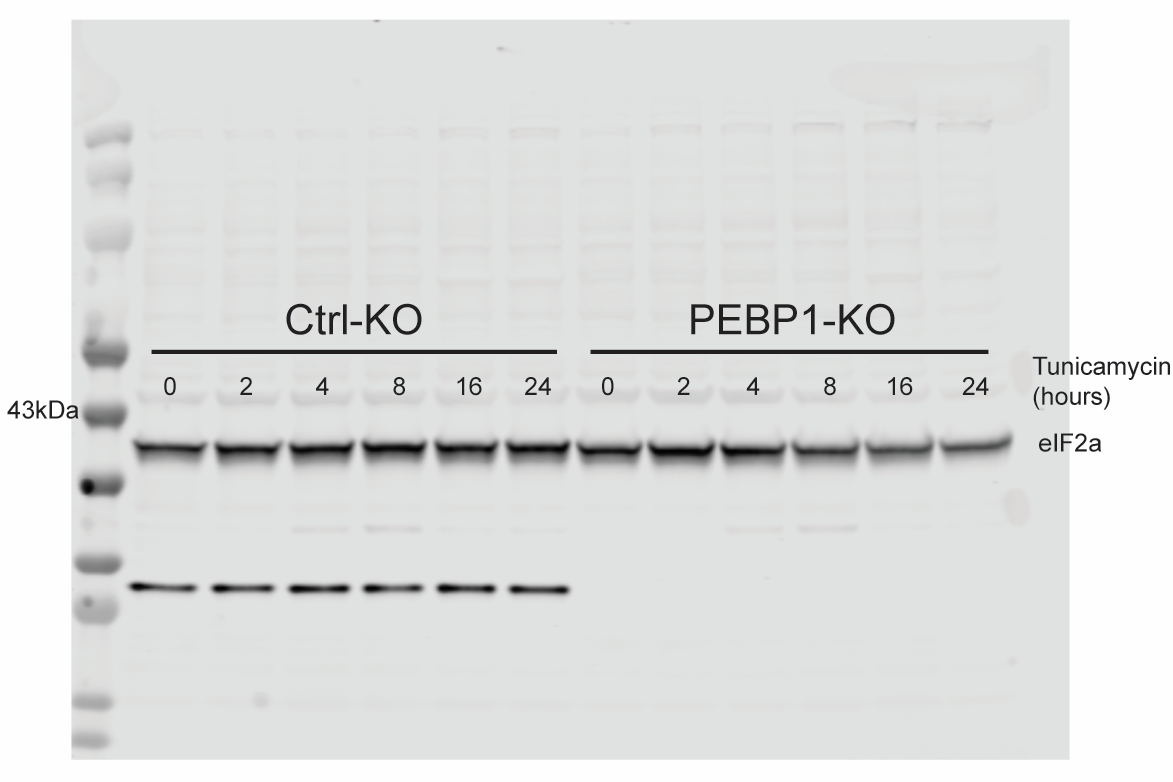

Supplement: Figure 3—source data 1. [file elife-102852-fig3-data1.zip › Figure 3-source data 1/Fig3F_eIF2a_band_indicated.tif]

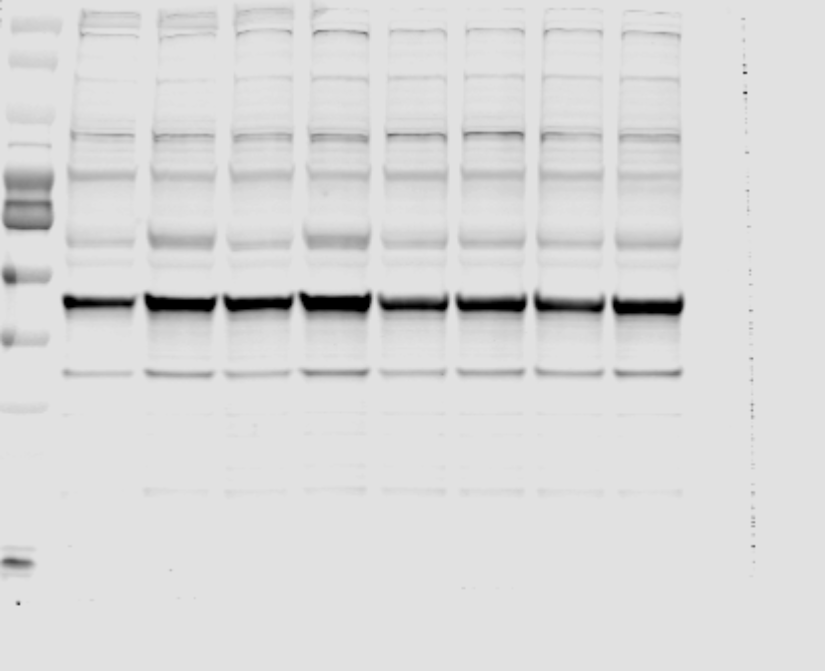

Supplement: Figure 3—source data 1. [file elife-102852-fig3-data1.zip › Figure 3-source data 1/Fig3A_ATF4_original.tif]

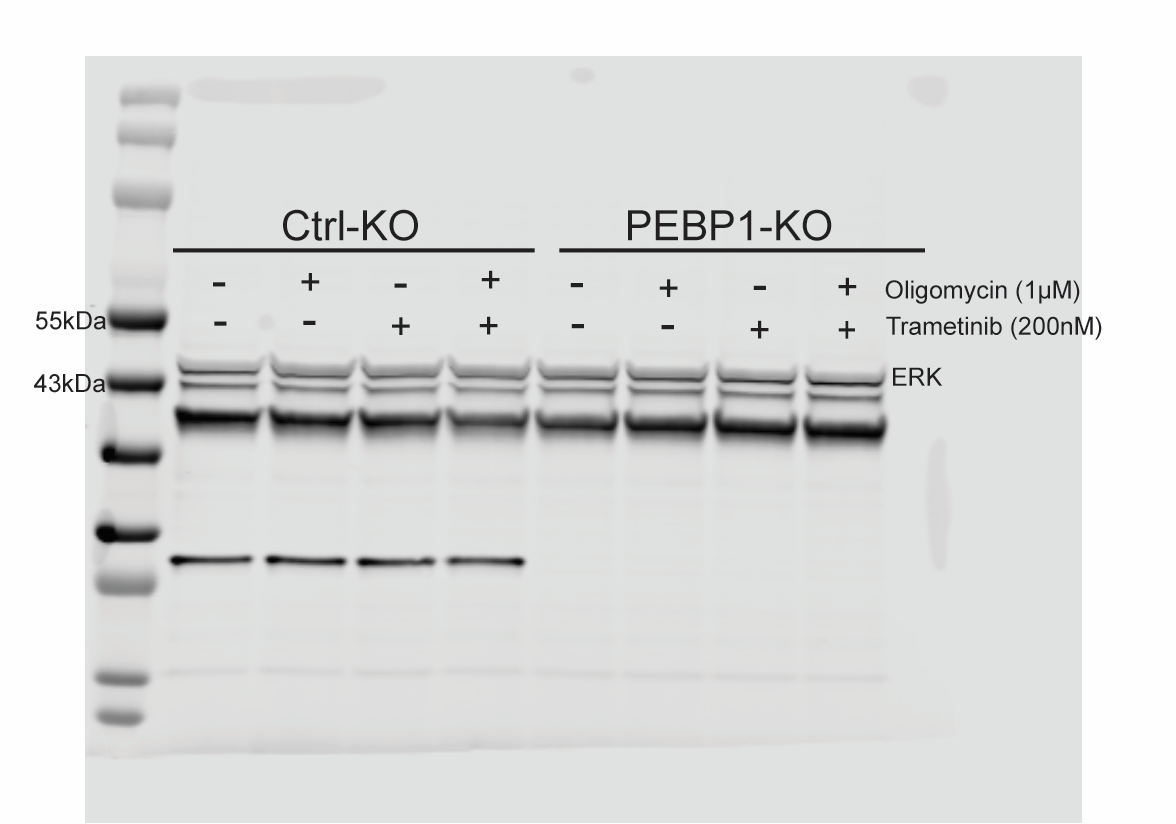

Supplement: Figure 3—source data 1. [file elife-102852-fig3-data1.zip › Figure 3-source data 1/Fig3A_ERK_band_indicated.tif]

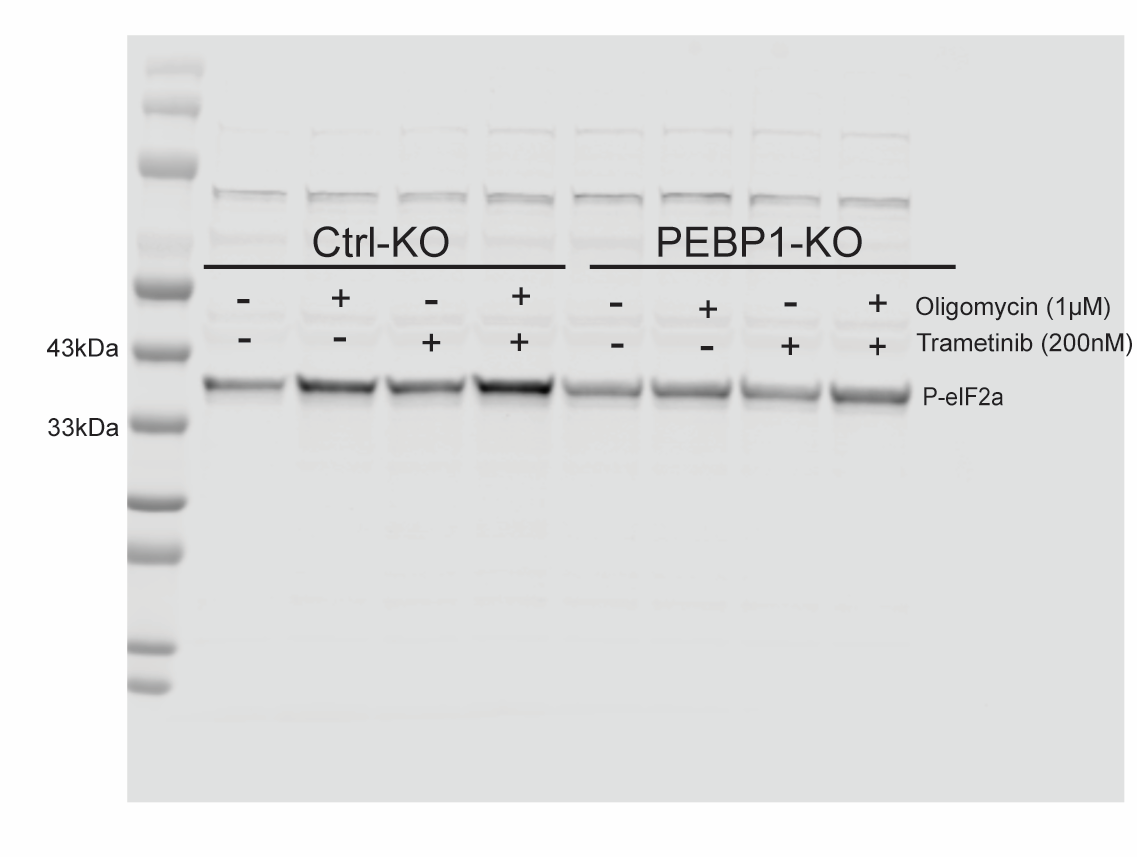

Supplement: Figure 3—source data 1. [file elife-102852-fig3-data1.zip › Figure 3-source data 1/Fig3A_P-eIF2a_band_indicated.tif]

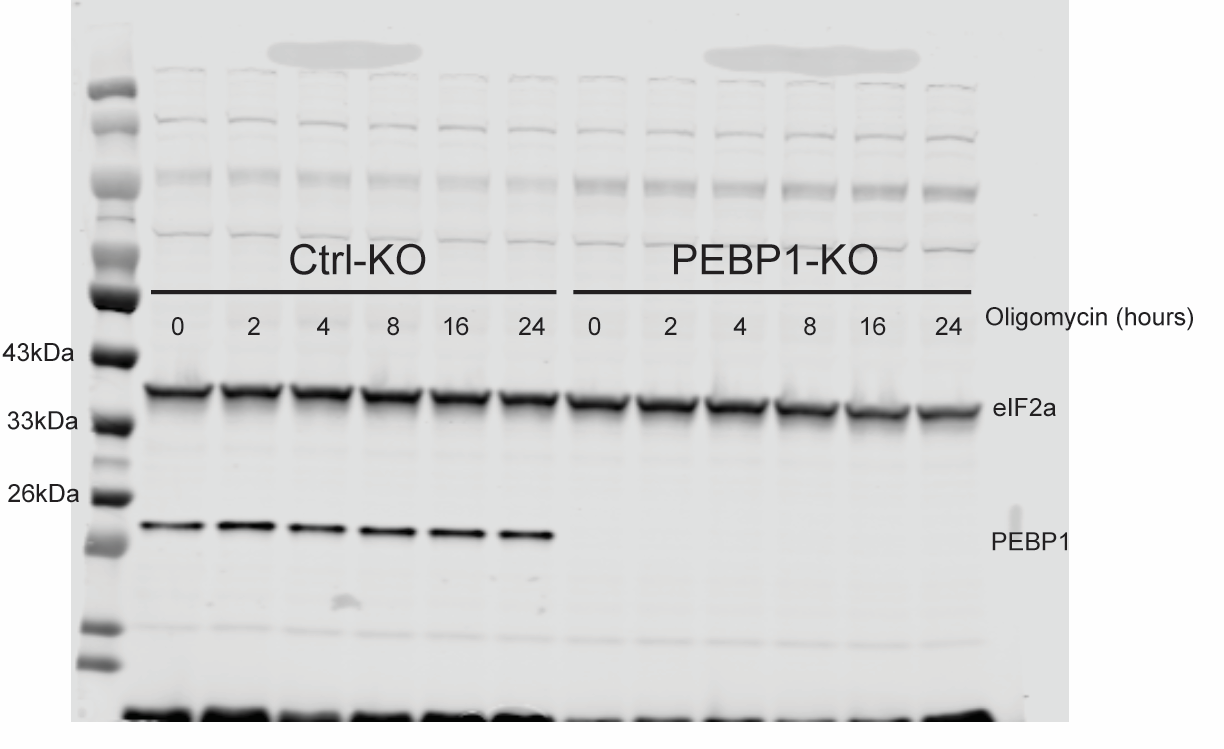

Supplement: Figure 3—source data 1. [file elife-102852-fig3-data1.zip › Figure 3-source data 1/Fig3D_eIF2a_PEBP1_band_indicated.tif]

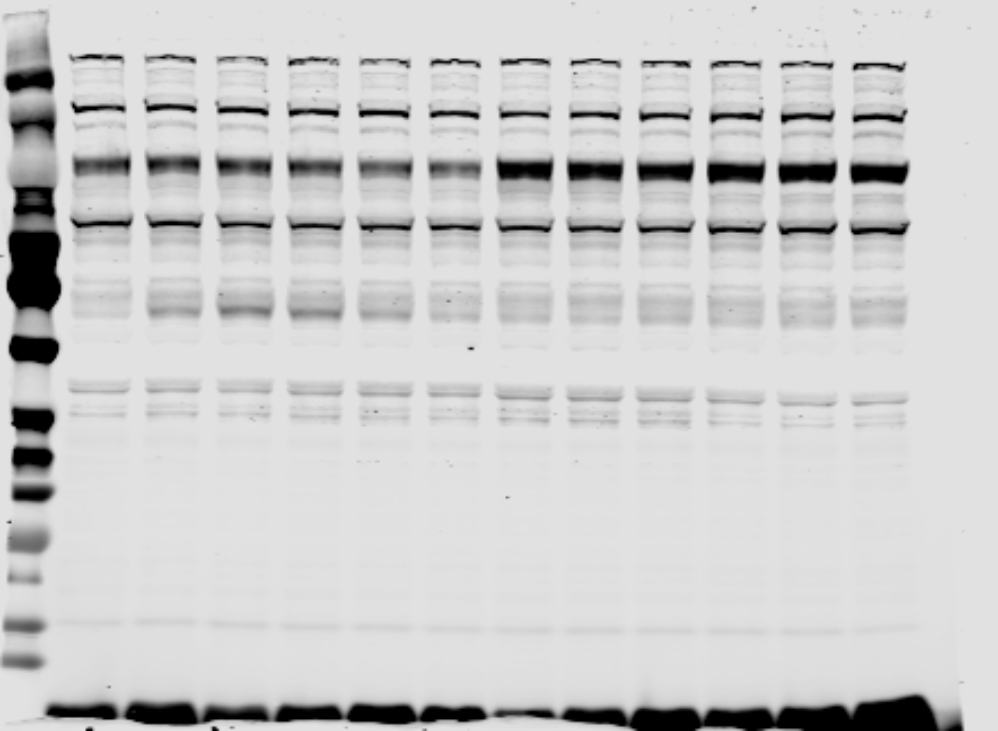

Supplement: Figure 3—source data 1. [file elife-102852-fig3-data1.zip › Figure 3-source data 1/Fig3D_ATF4_original.tif]

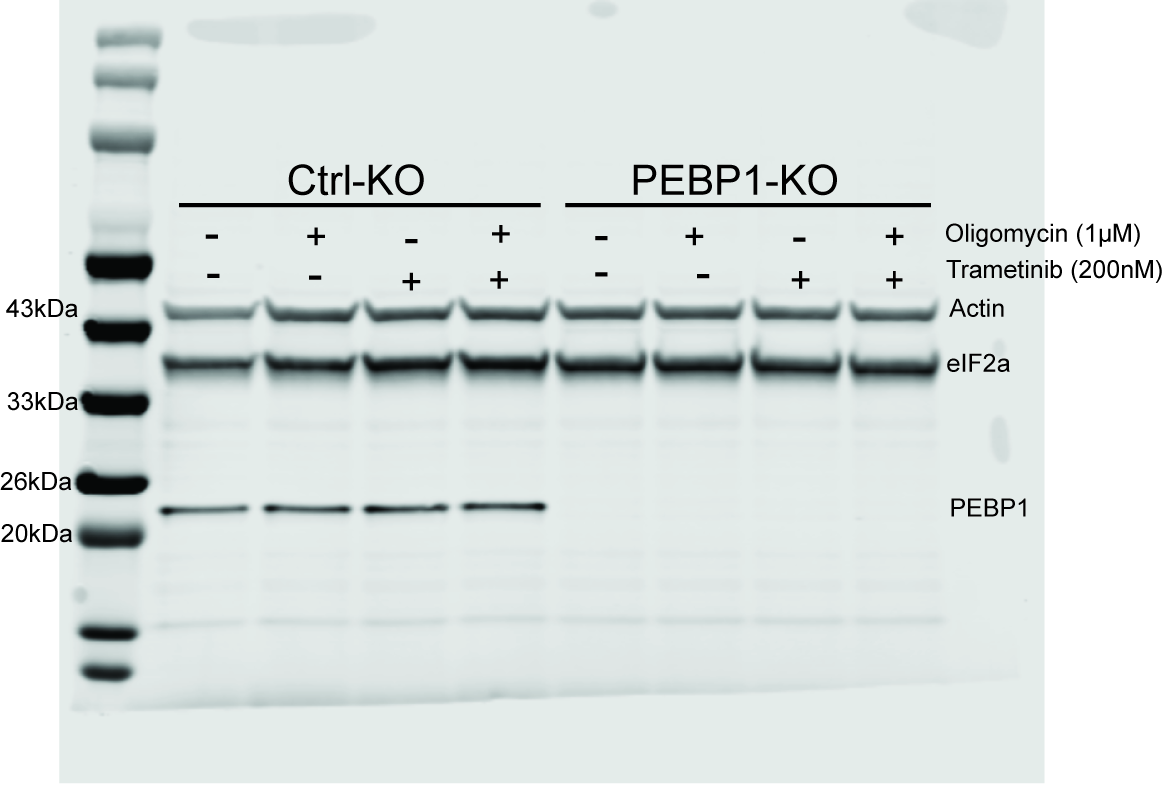

Supplement: Figure 3—source data 1. [file elife-102852-fig3-data1.zip › Figure 3-source data 1/Fig3A_Actin_eIF2a_PEBP1_bands_indicated.tif]

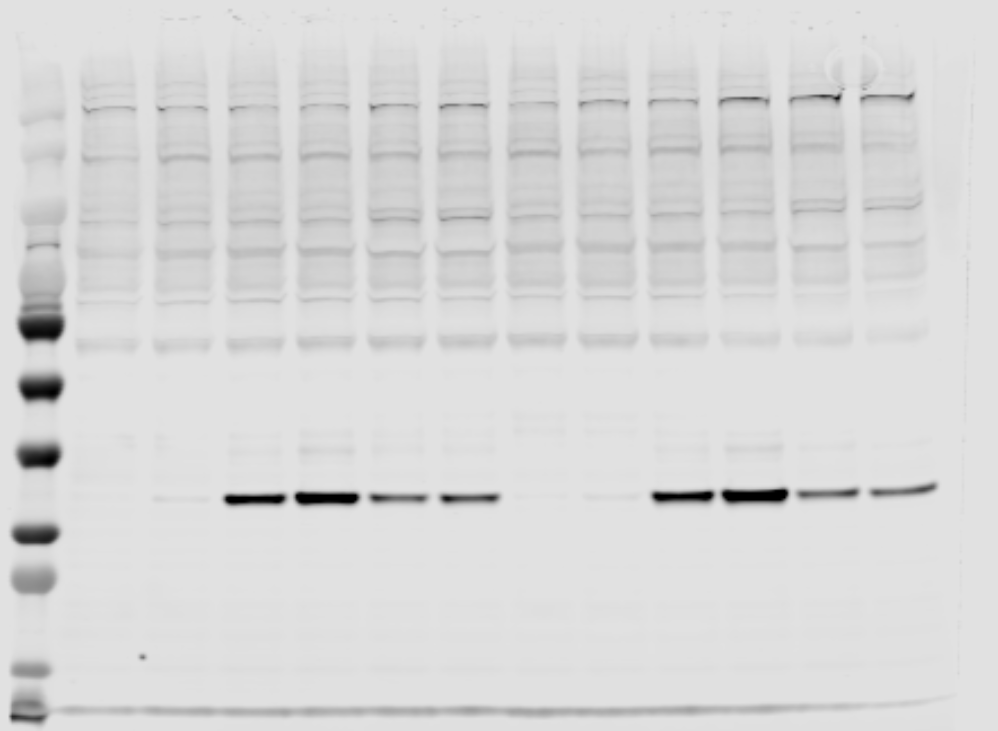

Supplement: Figure 3—source data 1. [file elife-102852-fig3-data1.zip › Figure 3-source data 1/Fig3F_CHOP_original.tif]

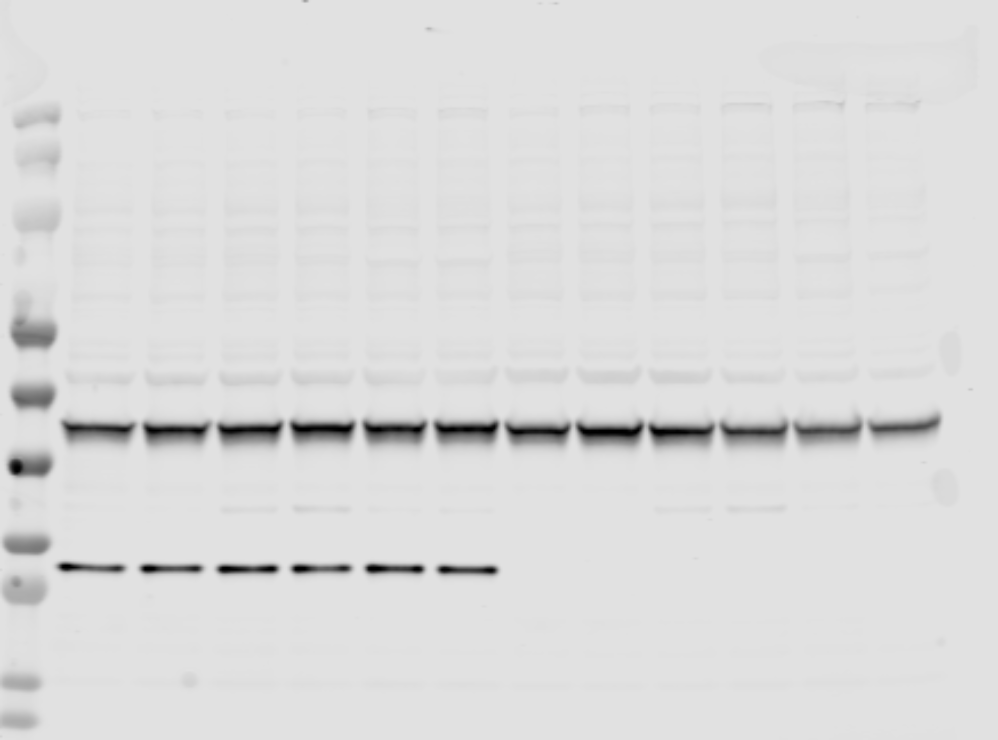

Supplement: Figure 3—source data 1. [file elife-102852-fig3-data1.zip › Figure 3-source data 1/Fig3F_eIF2a_original.tif]

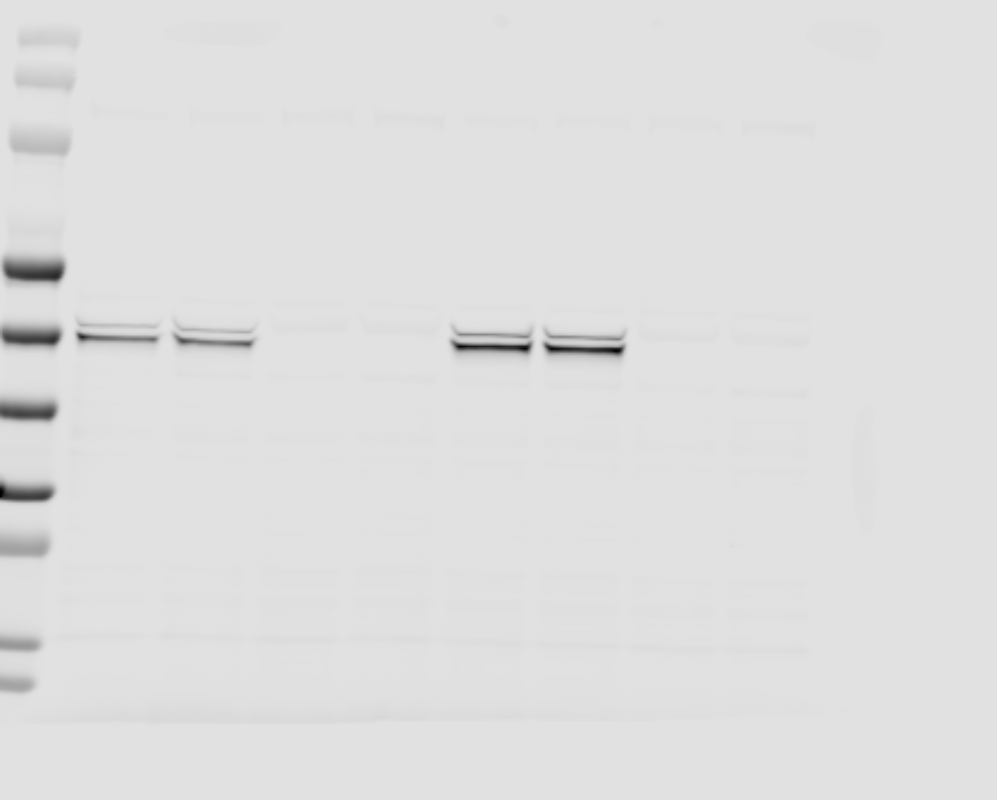

Supplement: Figure 3—source data 1. [file elife-102852-fig3-data1.zip › Figure 3-source data 1/Fig3A_P-ERK_original.tif]

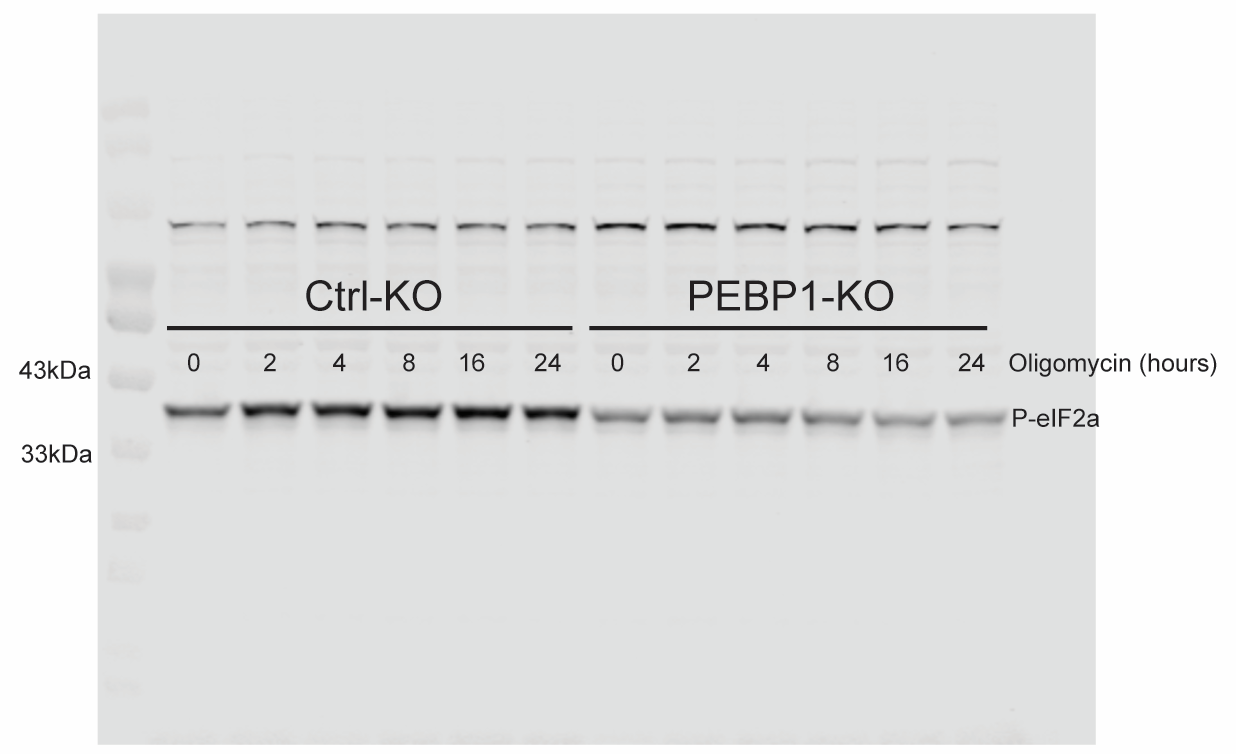

Supplement: Figure 3—source data 1. [file elife-102852-fig3-data1.zip › Figure 3-source data 1/Fig3D_P-eIF2a_band_indicated.tif]

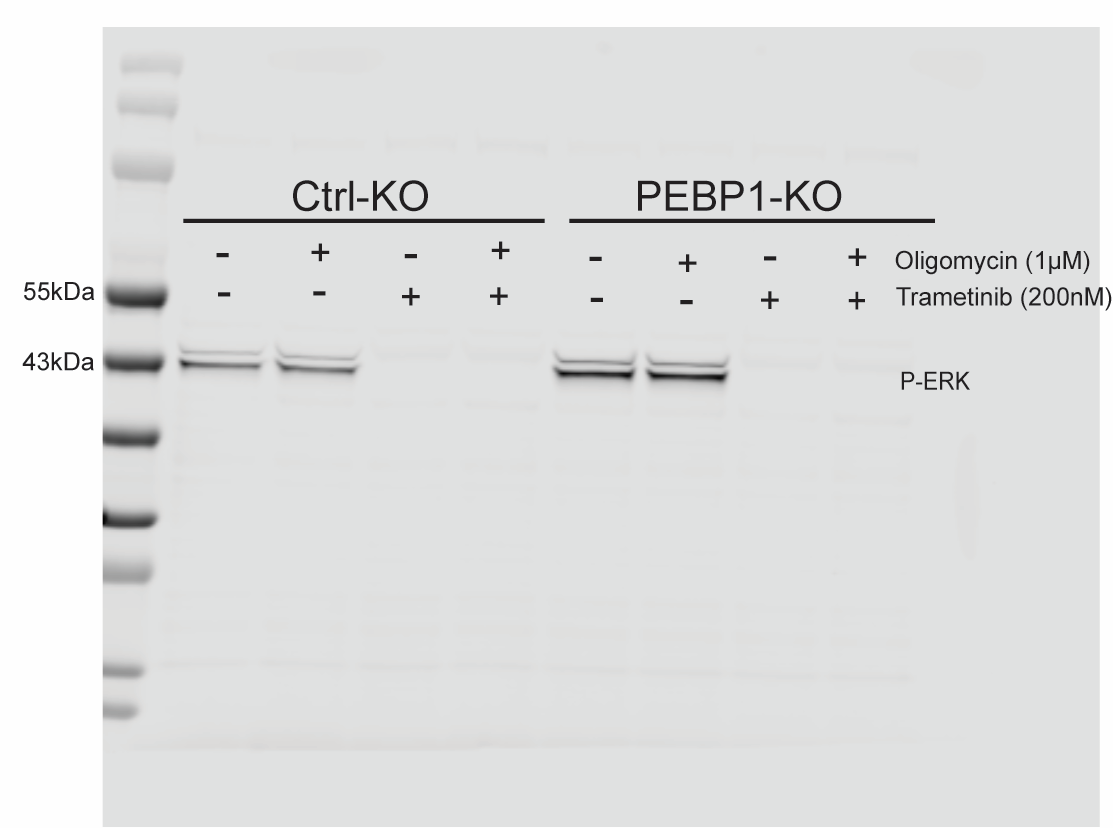

Supplement: Figure 3—source data 1. [file elife-102852-fig3-data1.zip › Figure 3-source data 1/Fig3A_P-ERK_band_indicated.tif]

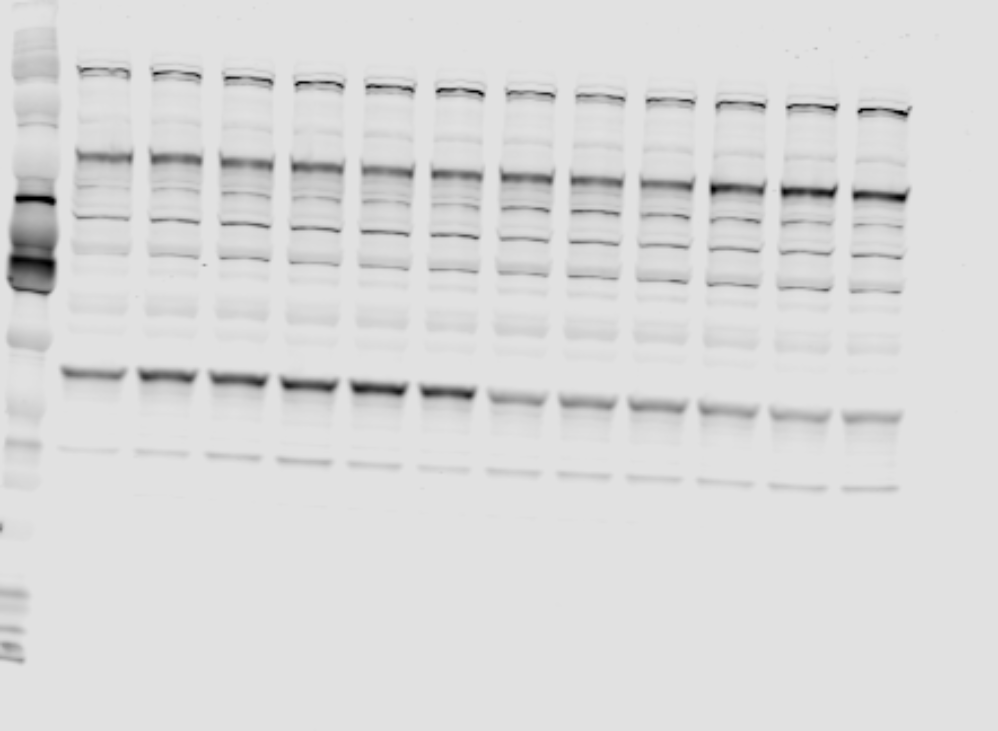

Supplement: Figure 3—source data 1. [file elife-102852-fig3-data1.zip › Figure 3-source data 1/Fig3D_CREP_original.tif]

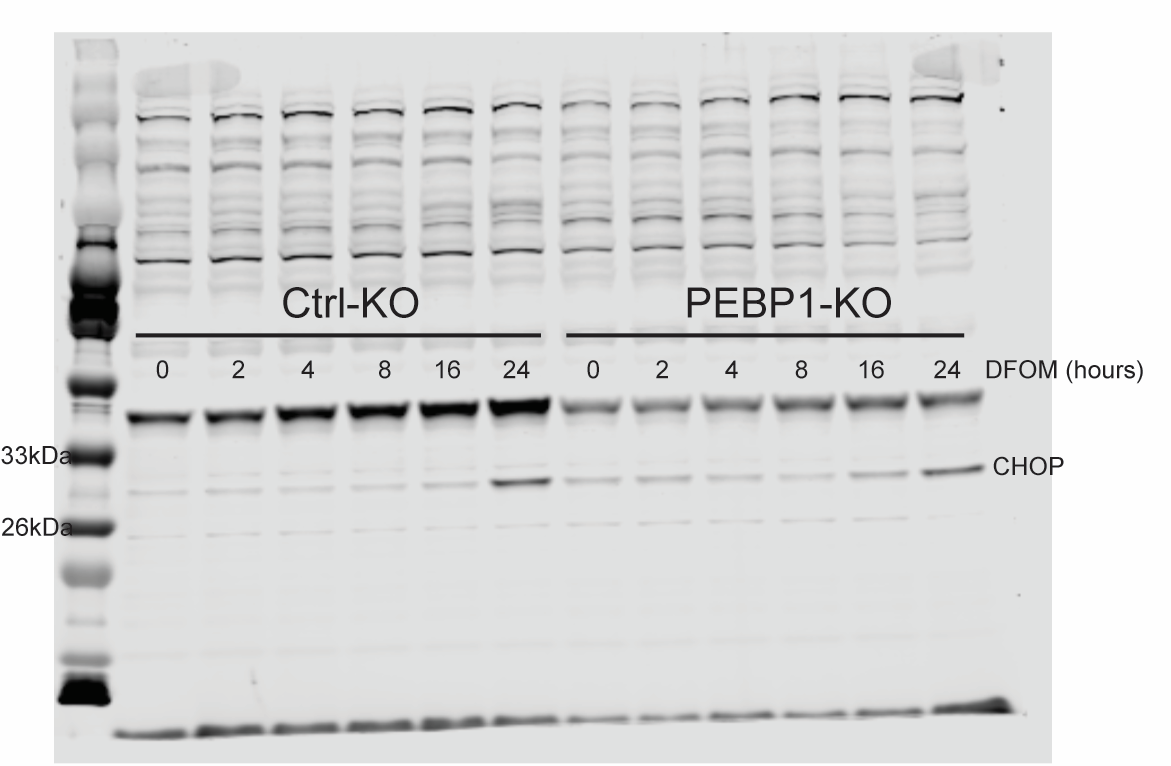

Supplement: Figure 3—source data 1. [file elife-102852-fig3-data1.zip › Figure 3-source data 1/Fig3E_CHOP_band_indicated.tif]

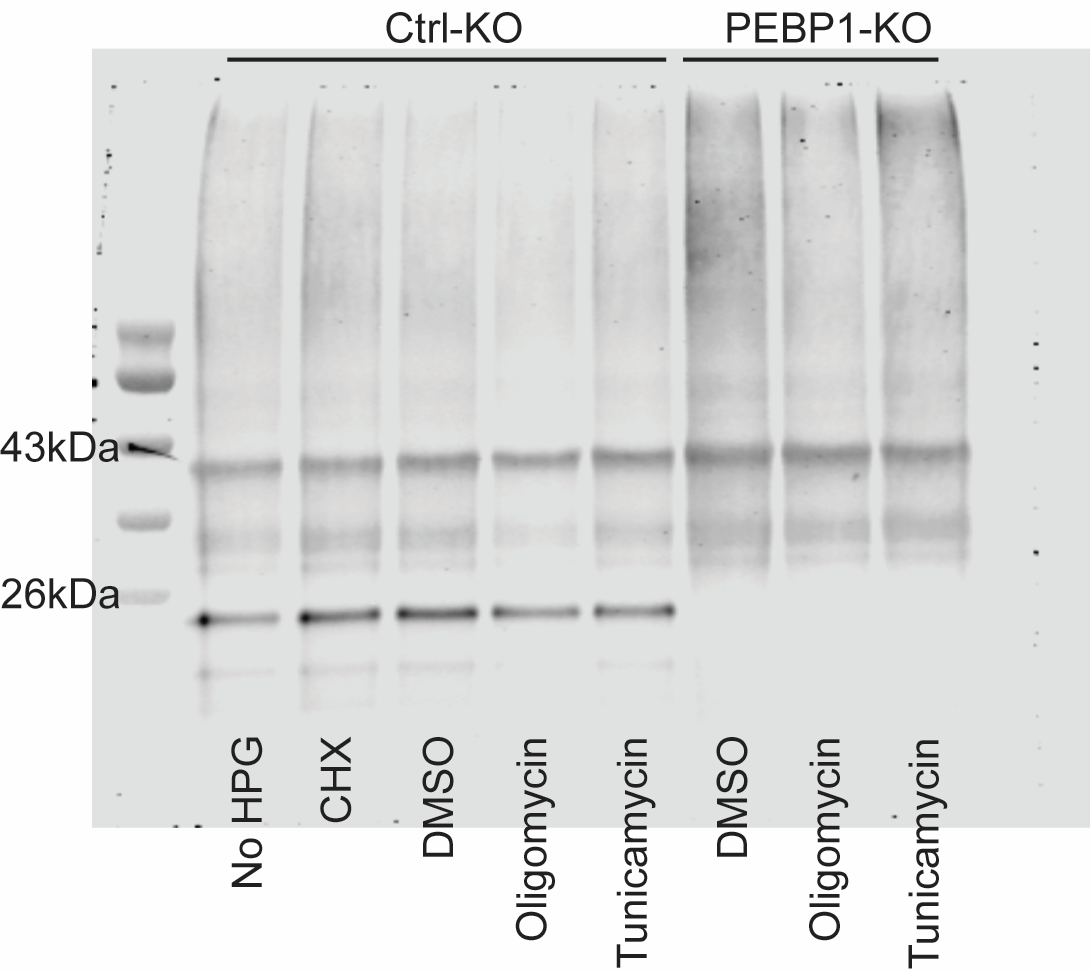

Supplement: Figure 3—source data 1. [file elife-102852-fig3-data1.zip › Figure 3-source data 1/Fig3C_GAPDH_PEBP1_bands_indicated.tif]

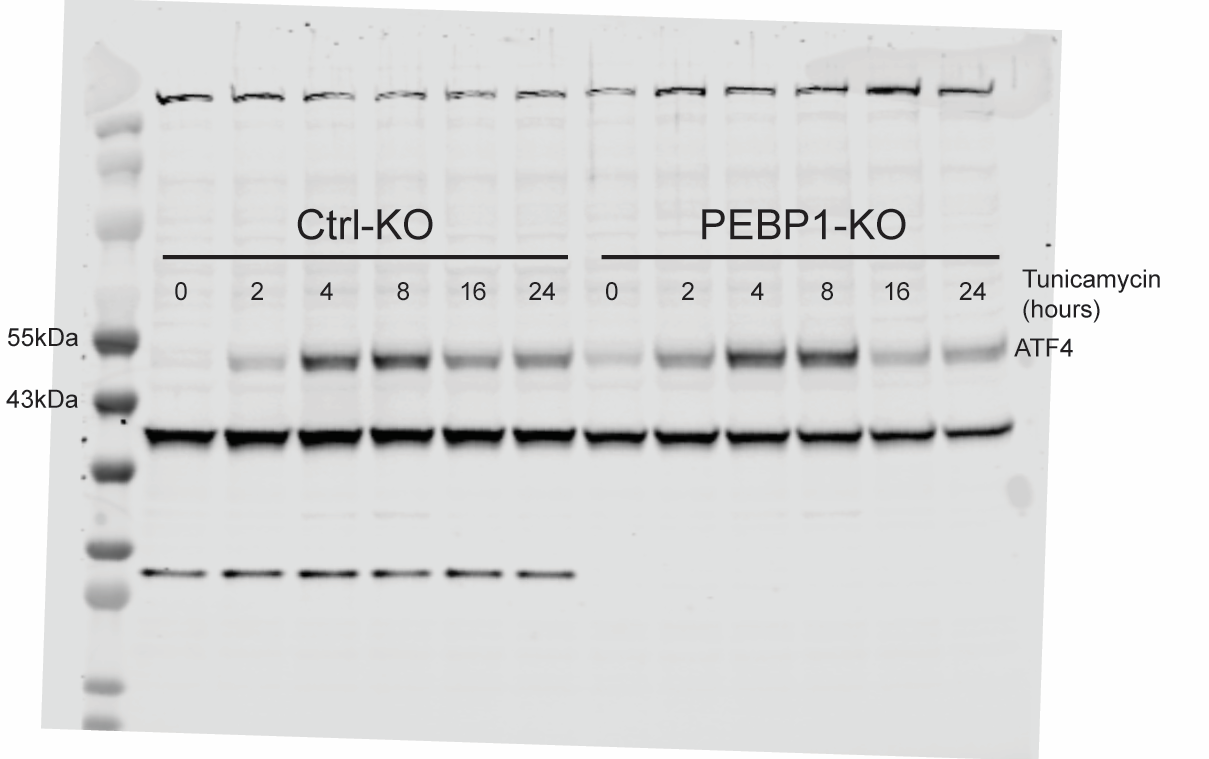

Supplement: Figure 3—source data 1. [file elife-102852-fig3-data1.zip › Figure 3-source data 1/Fig3F_ATF4_band_indicated.tif]

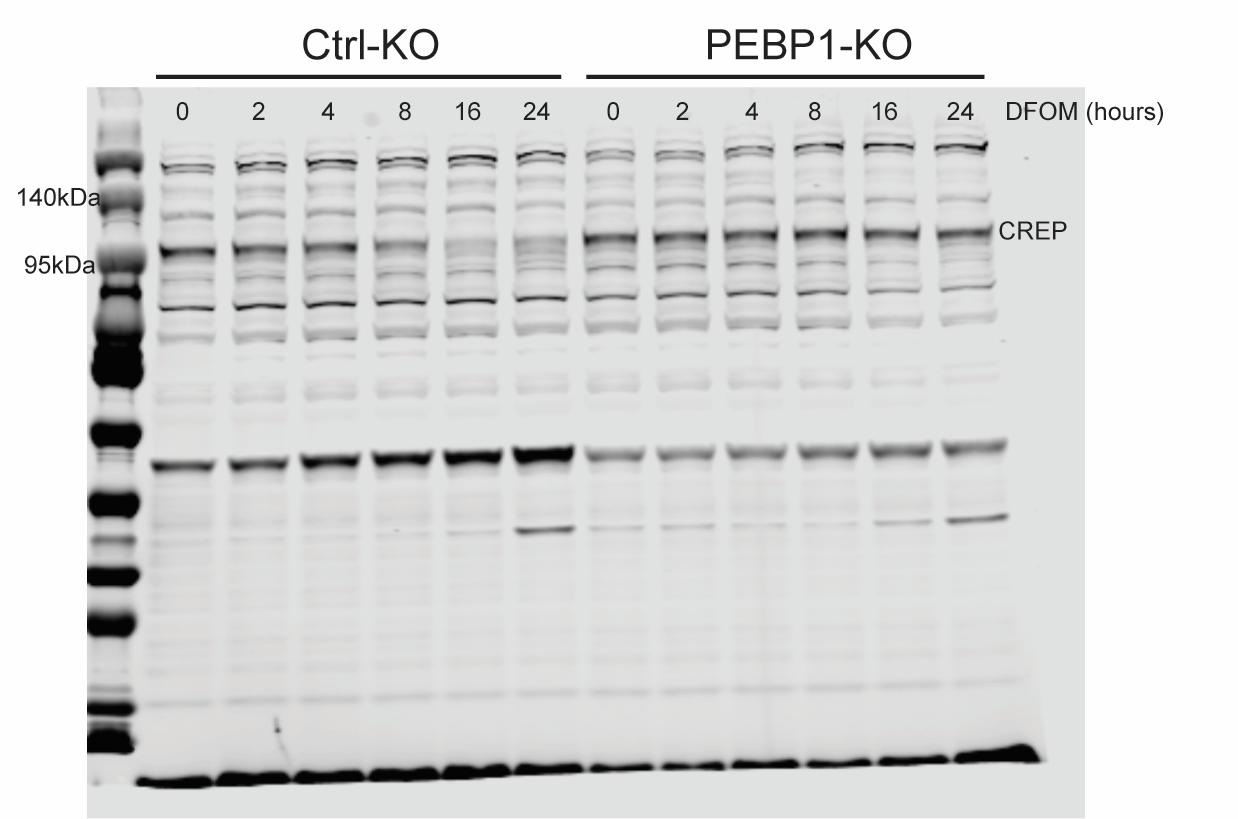

Supplement: Figure 3—source data 1. [file elife-102852-fig3-data1.zip › Figure 3-source data 1/Fig3E_CREP_band_indicated.tif]

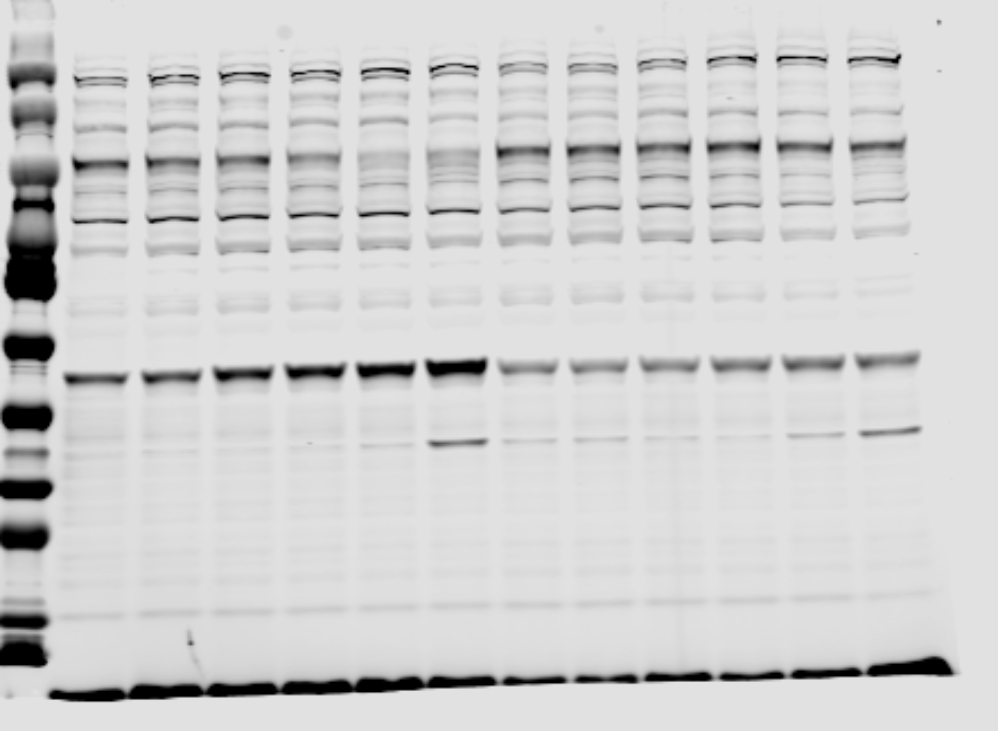

Supplement: Figure 3—source data 1. [file elife-102852-fig3-data1.zip › Figure 3-source data 1/Fig3E_CREP_original.tif]

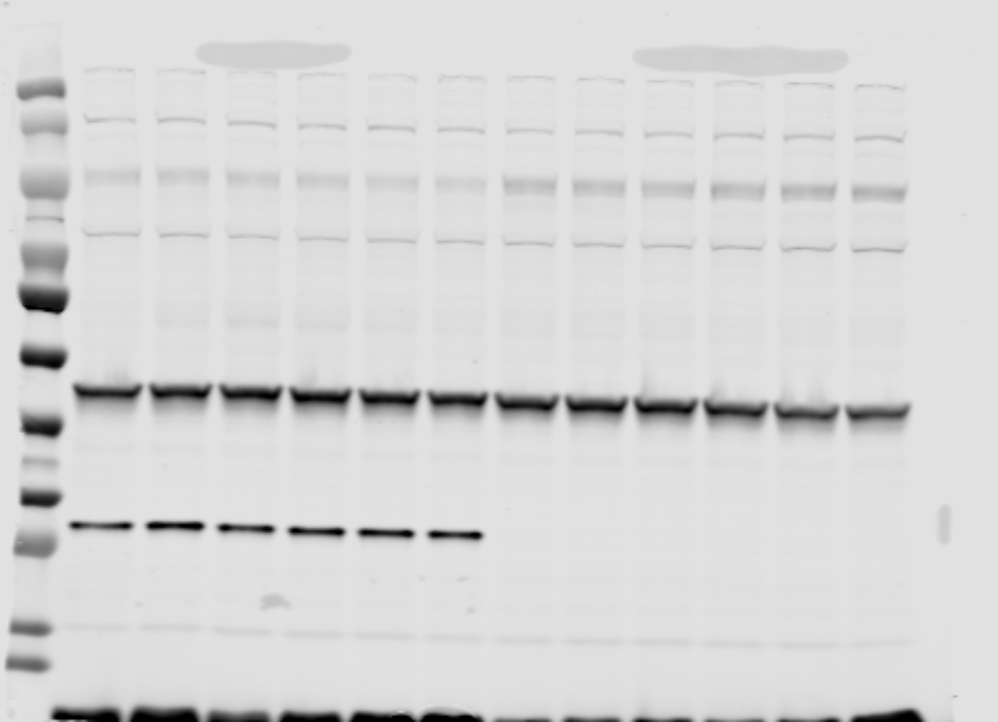

Supplement: Figure 3—source data 1. [file elife-102852-fig3-data1.zip › Figure 3-source data 1/Fig3D_eIF2a_PEBP1_original.tif]

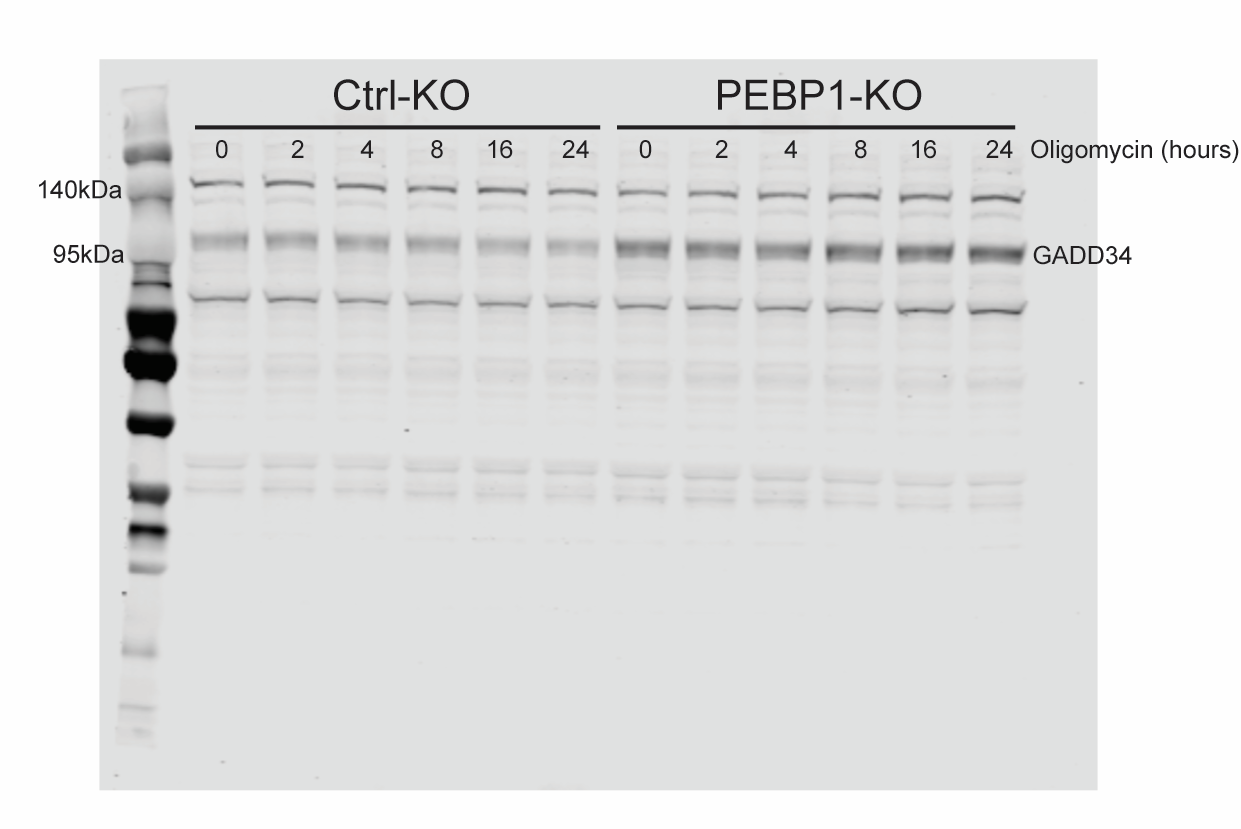

Supplement: Figure 3—source data 1. [file elife-102852-fig3-data1.zip › Figure 3-source data 1/Fig3D_GADD34_band_indicated.tif]

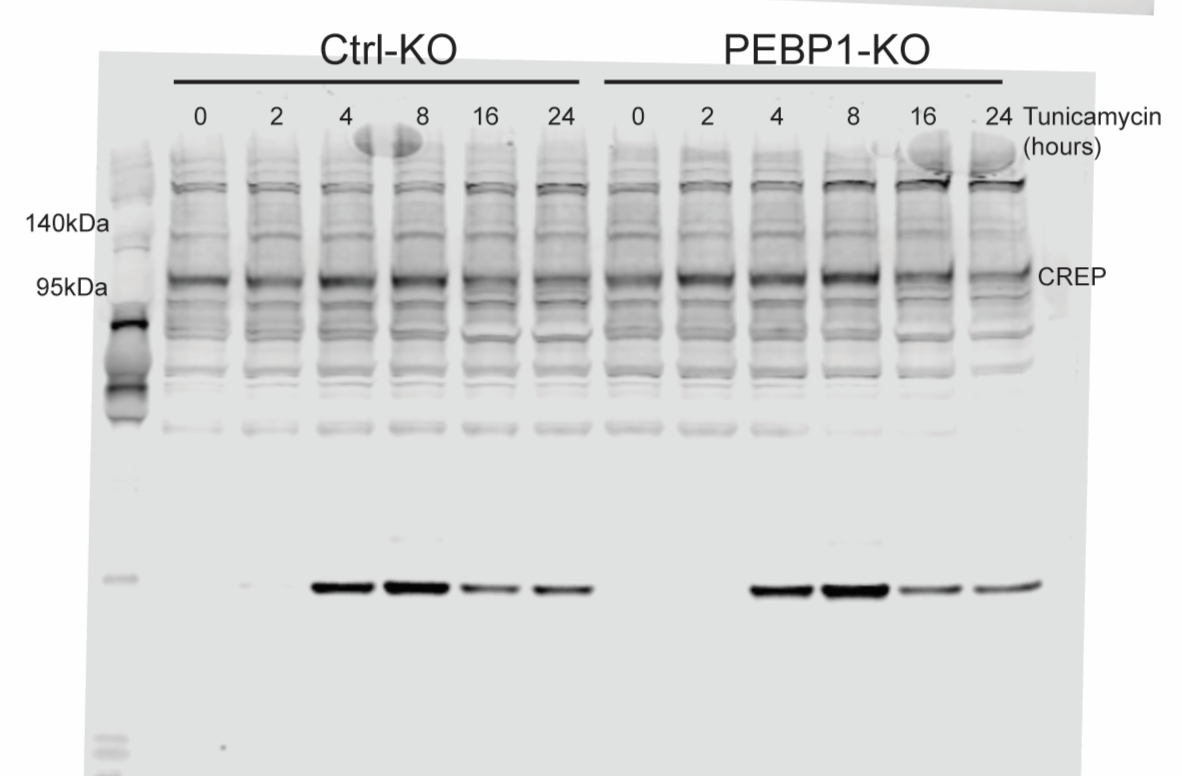

Supplement: Figure 3—source data 1. [file elife-102852-fig3-data1.zip › Figure 3-source data 1/Fig3F_CREP_band_indicated.tif]

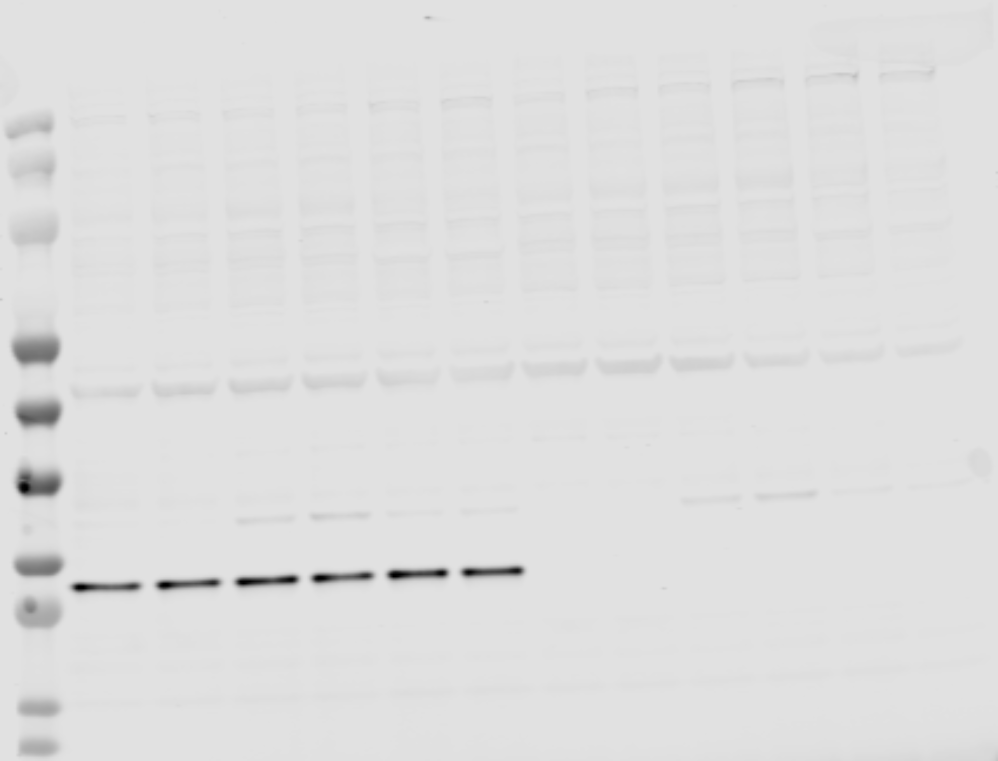

Supplement: Figure 3—source data 1. [file elife-102852-fig3-data1.zip › Figure 3-source data 1/Fig3F_PEBP1_original.tif]

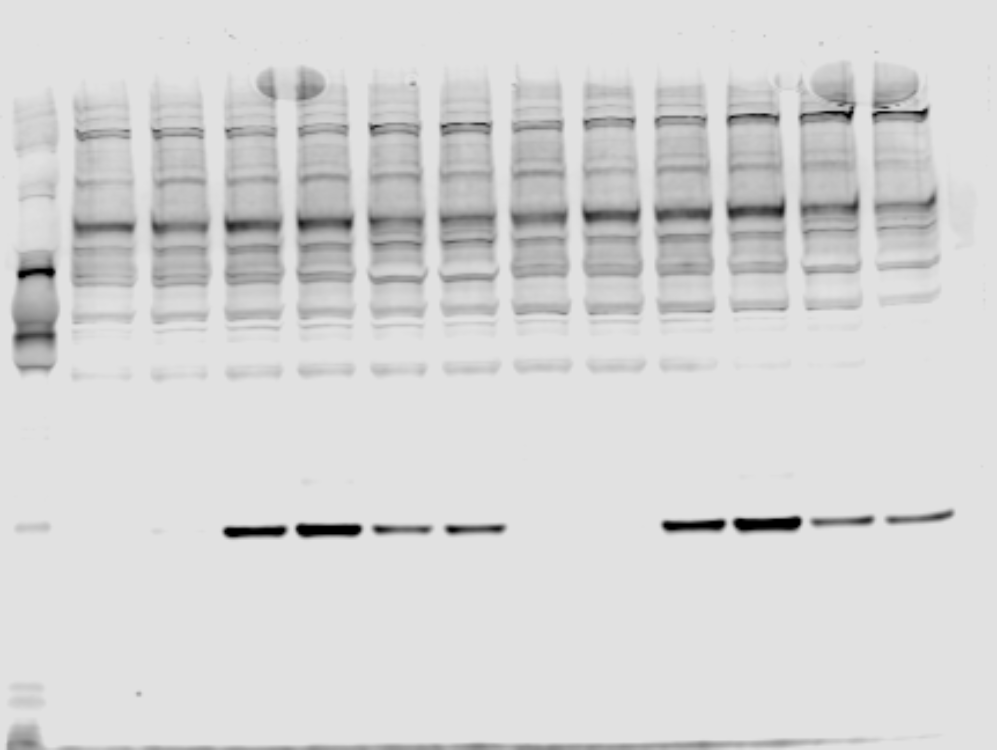

Supplement: Figure 3—source data 1. [file elife-102852-fig3-data1.zip › Figure 3-source data 1/Fig3F_CREP_original.tif]

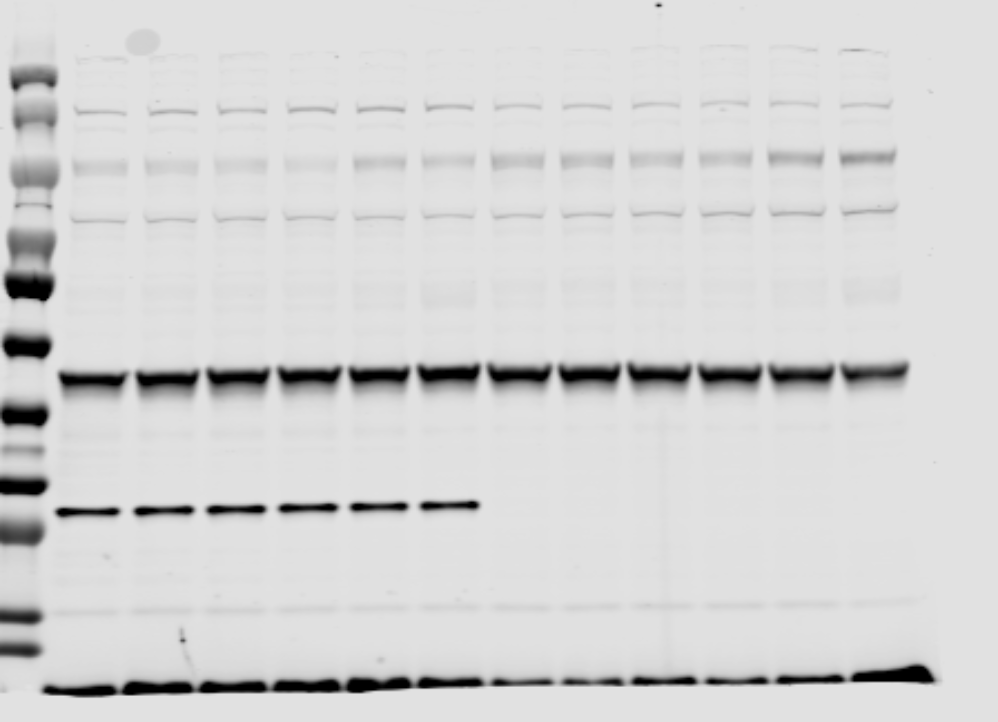

Supplement: Figure 3—source data 1. [file elife-102852-fig3-data1.zip › Figure 3-source data 1/Fig3E_eIF2a_PEBP1_original.tif]

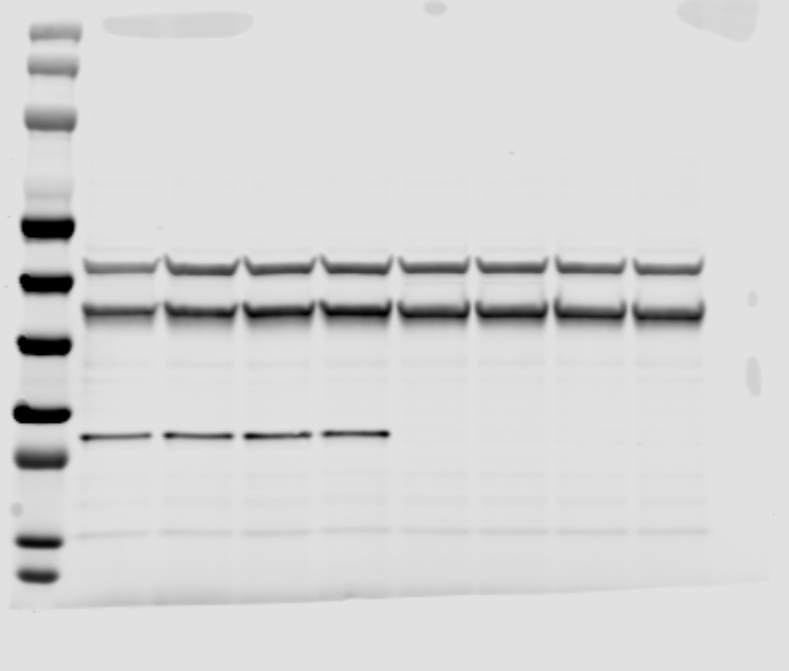

Supplement: Figure 3—source data 1. [file elife-102852-fig3-data1.zip › Figure 3-source data 1/Fig3A_Actin_eIF2a_PEBP1_original.tif]

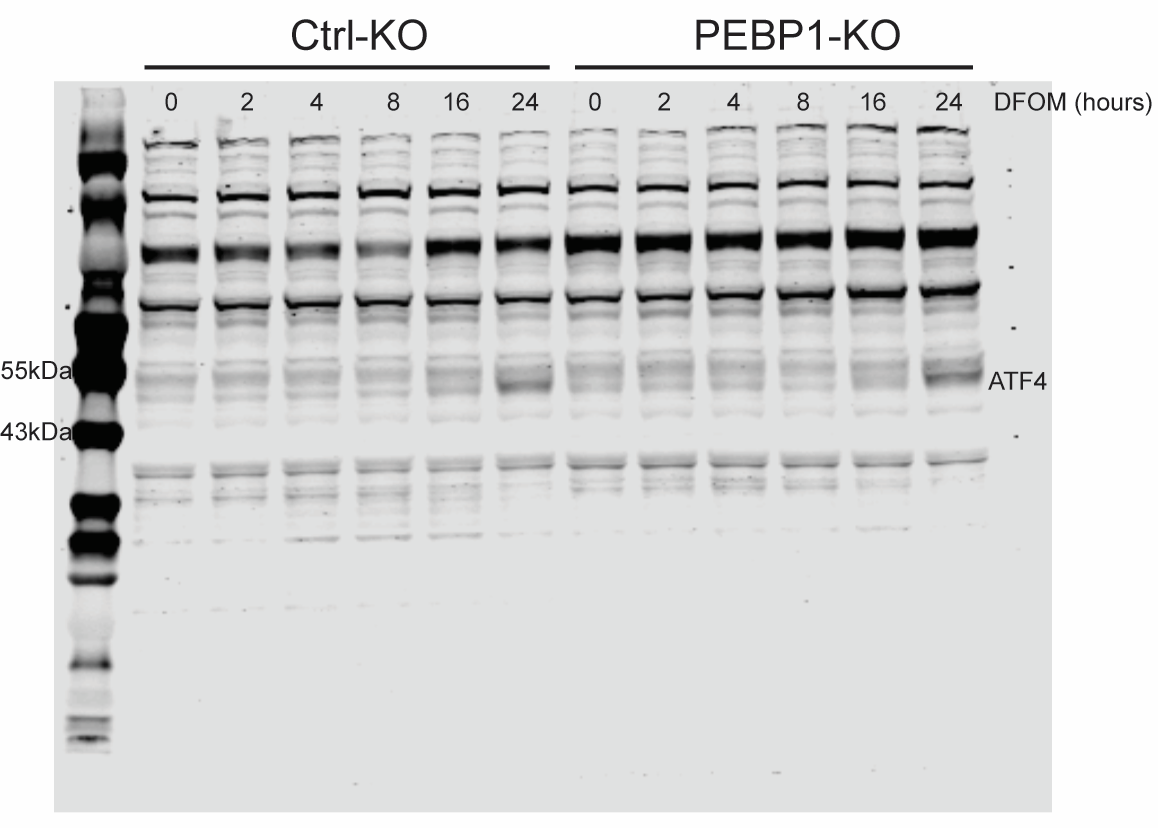

Supplement: Figure 3—source data 1. [file elife-102852-fig3-data1.zip › Figure 3-source data 1/Fig3E_ATF4_band_indicated.tif]

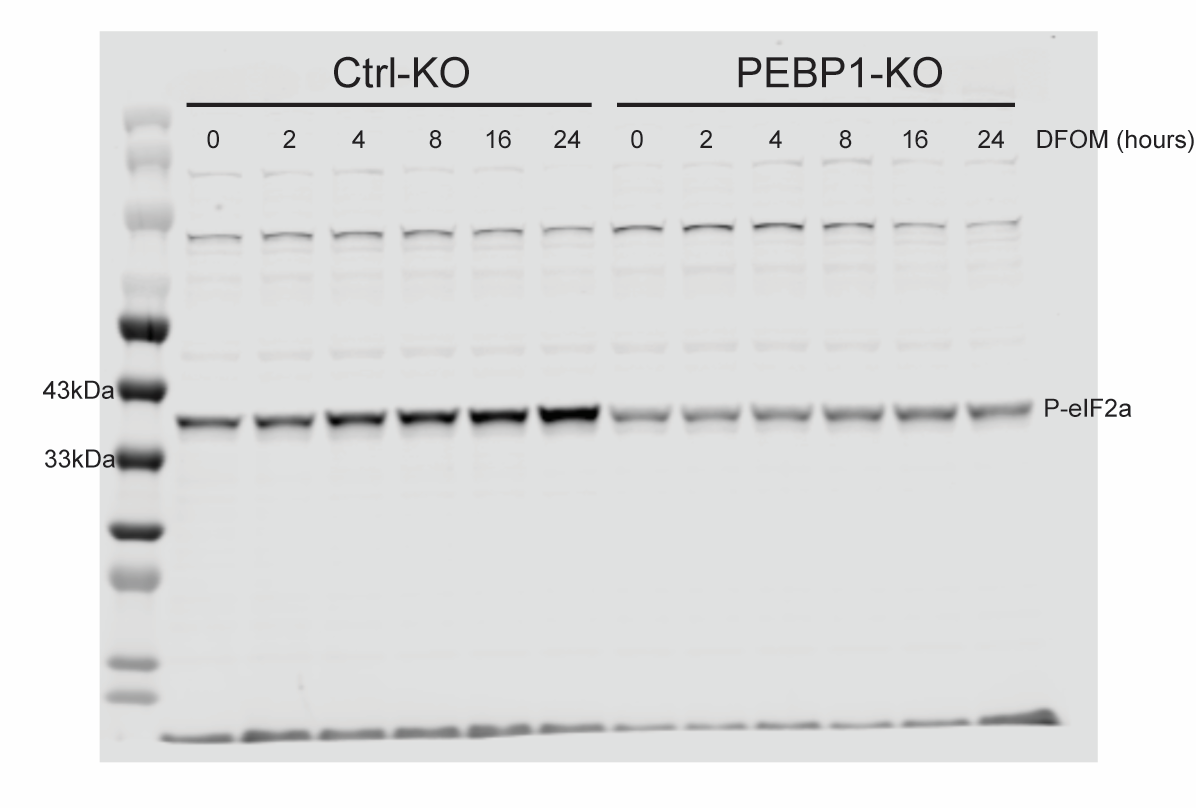

Supplement: Figure 3—source data 1. [file elife-102852-fig3-data1.zip › Figure 3-source data 1/Fig3E_P-eIF2a_band_indicated.tif]

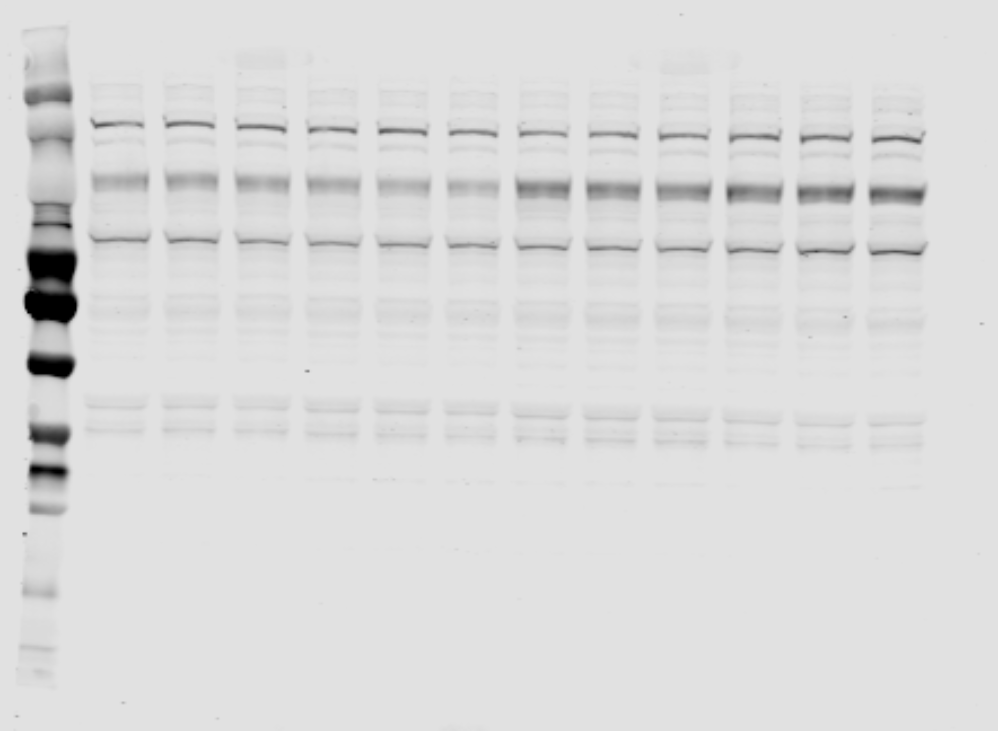

Supplement: Figure 3—source data 1. [file elife-102852-fig3-data1.zip › Figure 3-source data 1/Fig3D_GADD34_original.tif]

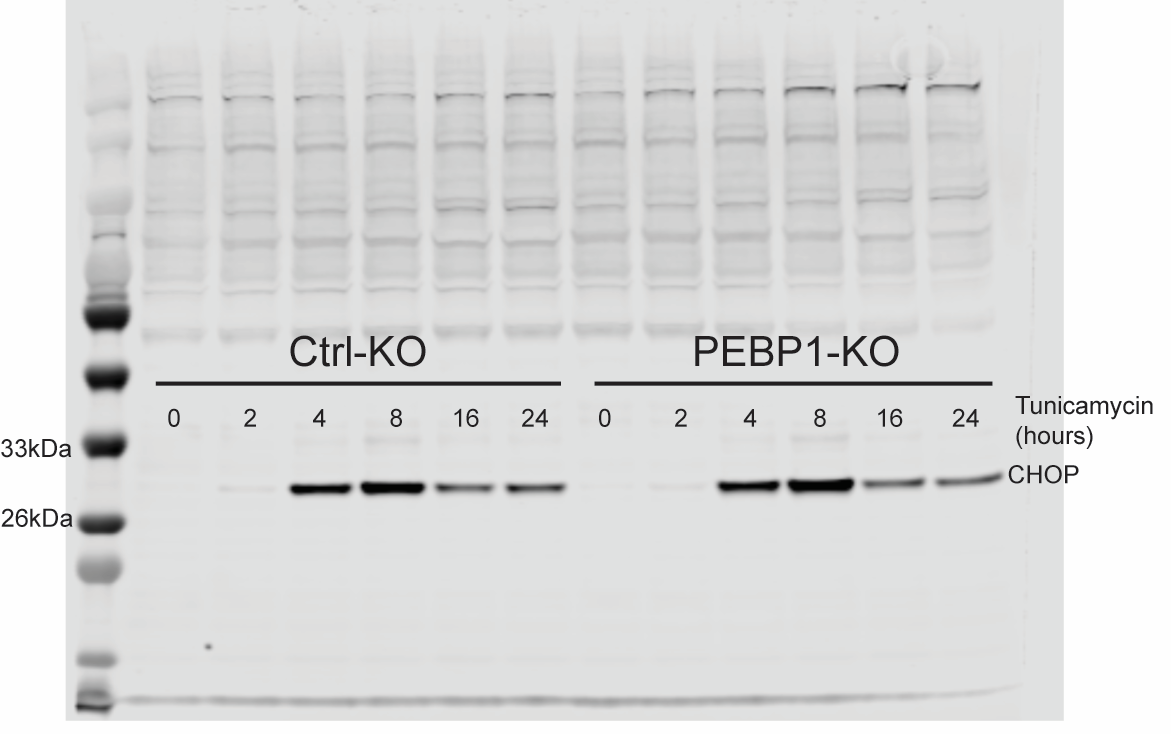

Supplement: Figure 3—source data 1. [file elife-102852-fig3-data1.zip › Figure 3-source data 1/Fig3F_CHOP_band_indicated.tif]

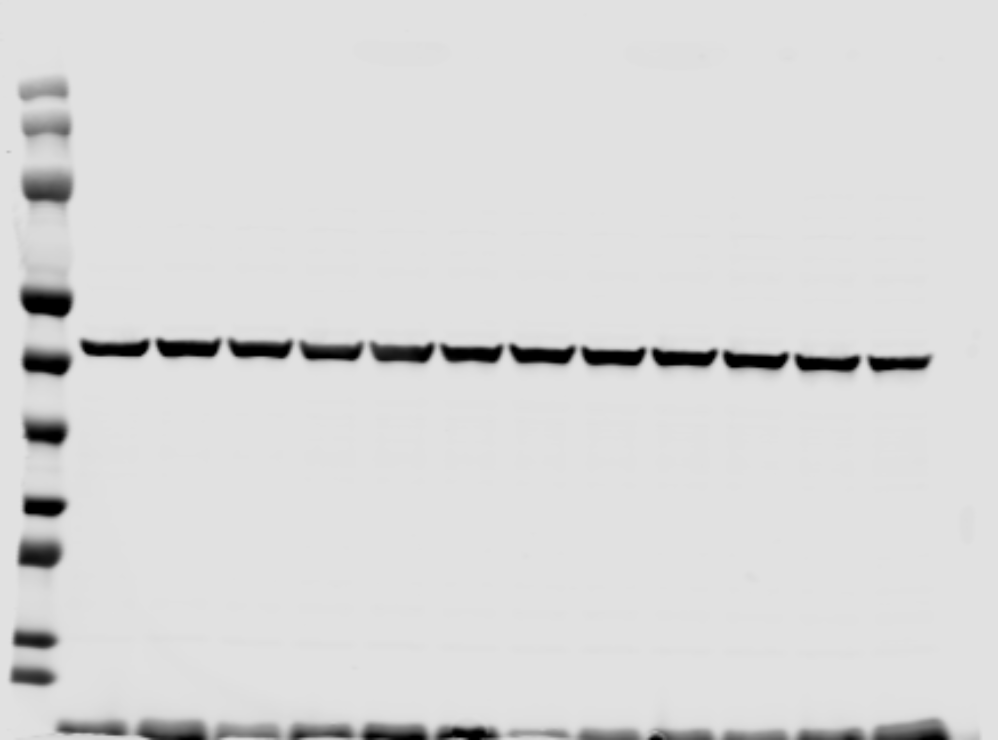

Supplement: Figure 3—source data 1. [file elife-102852-fig3-data1.zip › Figure 3-source data 1/Fig3D_Actin_original.tif]

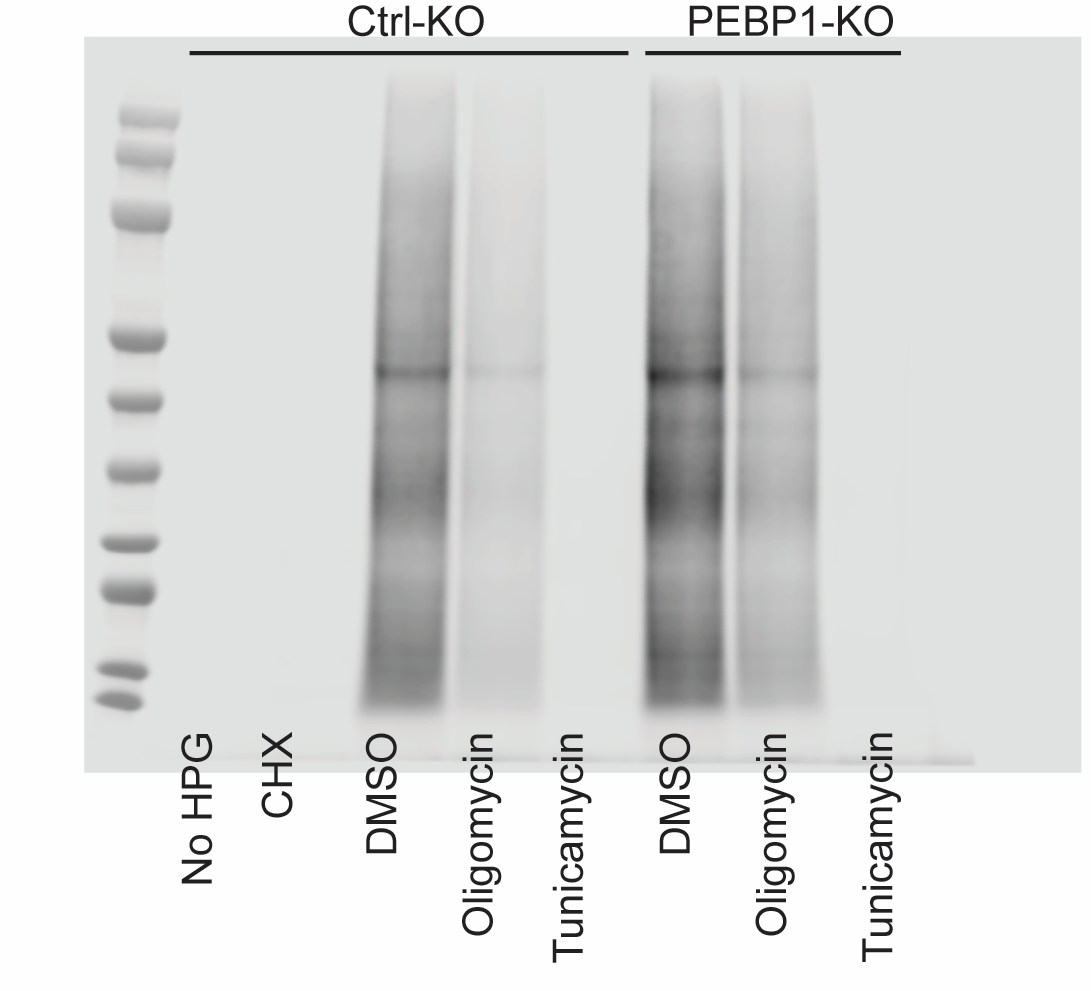

Supplement: Figure 3—source data 1. [file elife-102852-fig3-data1.zip › Figure 3-source data 1/Fig3C_HPG_bands_indicated.tif]

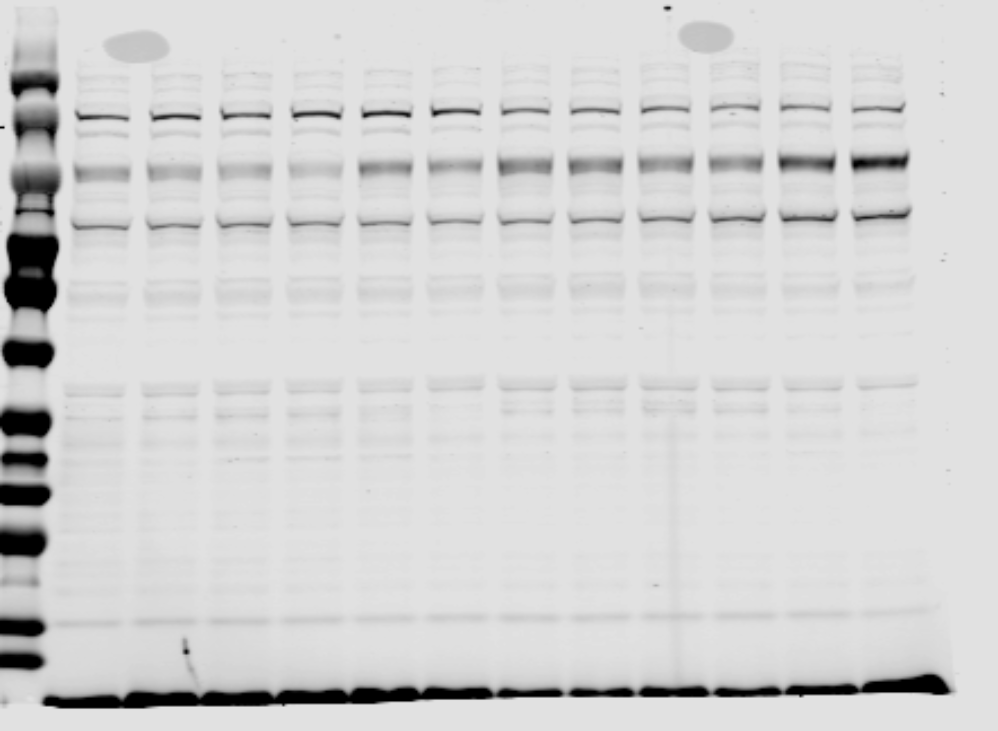

Supplement: Figure 3—source data 1. [file elife-102852-fig3-data1.zip › Figure 3-source data 1/Fig3E_GADD34_original.tif]

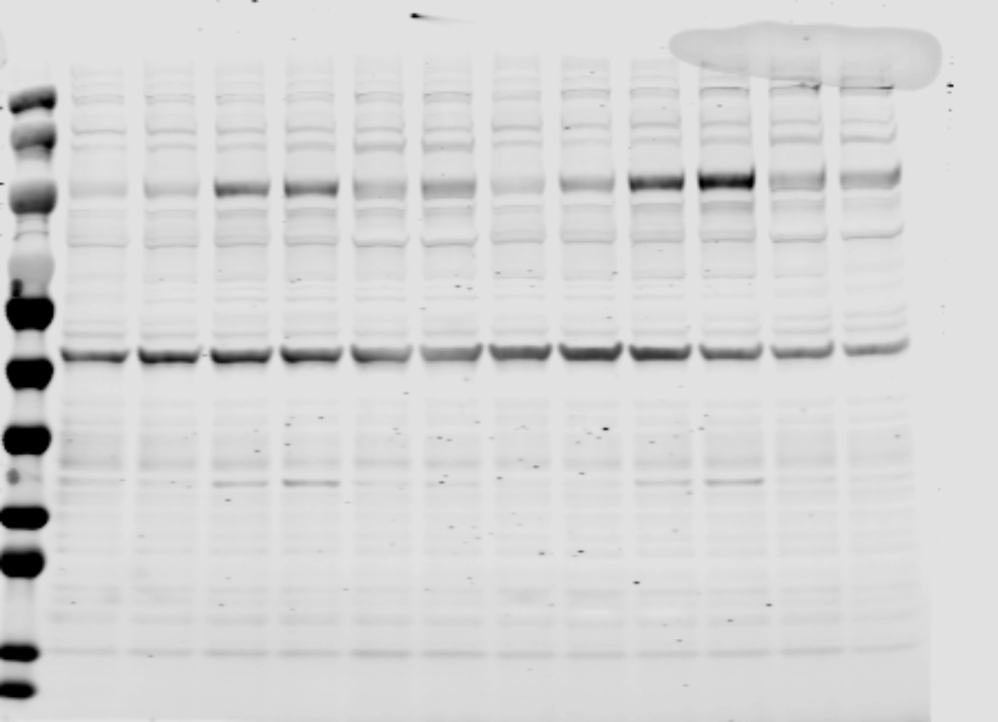

Supplement: Figure 3—source data 1. [file elife-102852-fig3-data1.zip › Figure 3-source data 1/Fig3F_GADD34_original.tif]

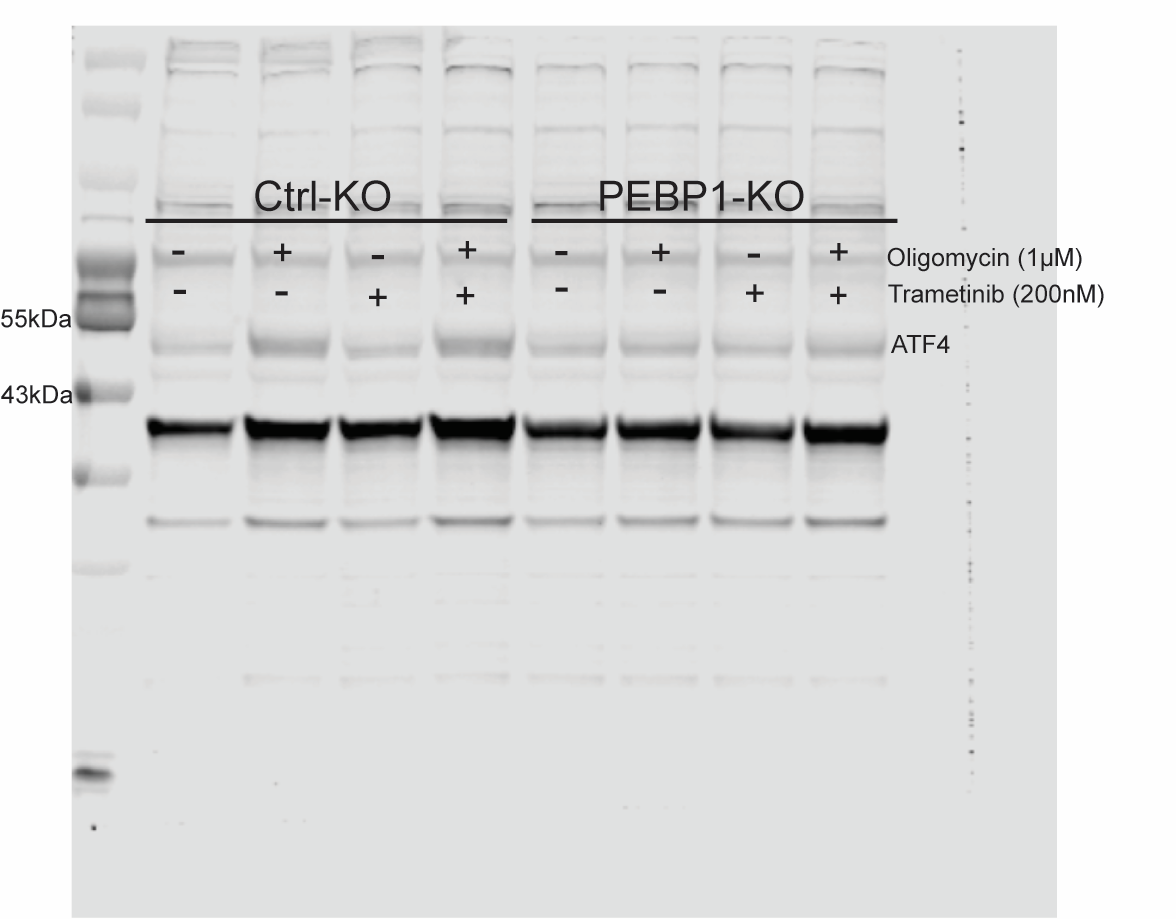

Supplement: Figure 3—source data 1. [file elife-102852-fig3-data1.zip › Figure 3-source data 1/Fig3A_ATF4_band_indicated.tif]

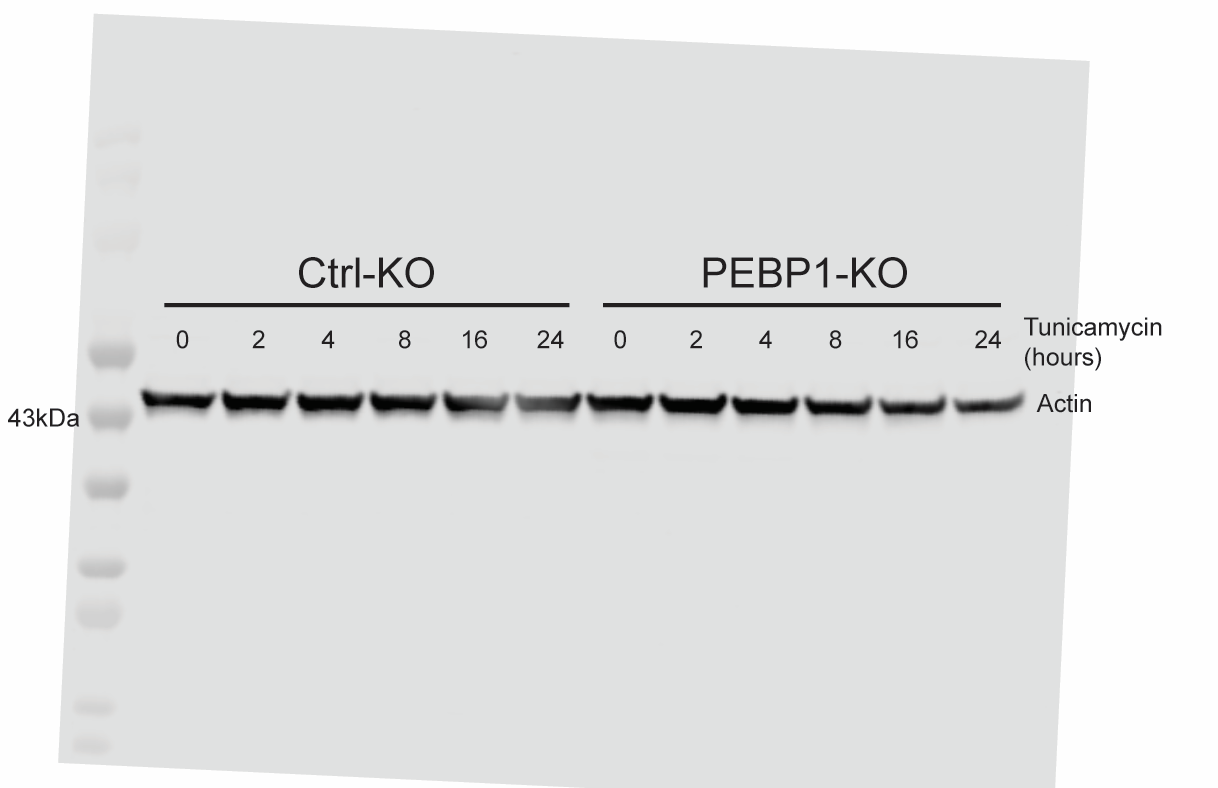

Supplement: Figure 3—source data 1. [file elife-102852-fig3-data1.zip › Figure 3-source data 1/Fig3F_Actin_band_indicated.tif]

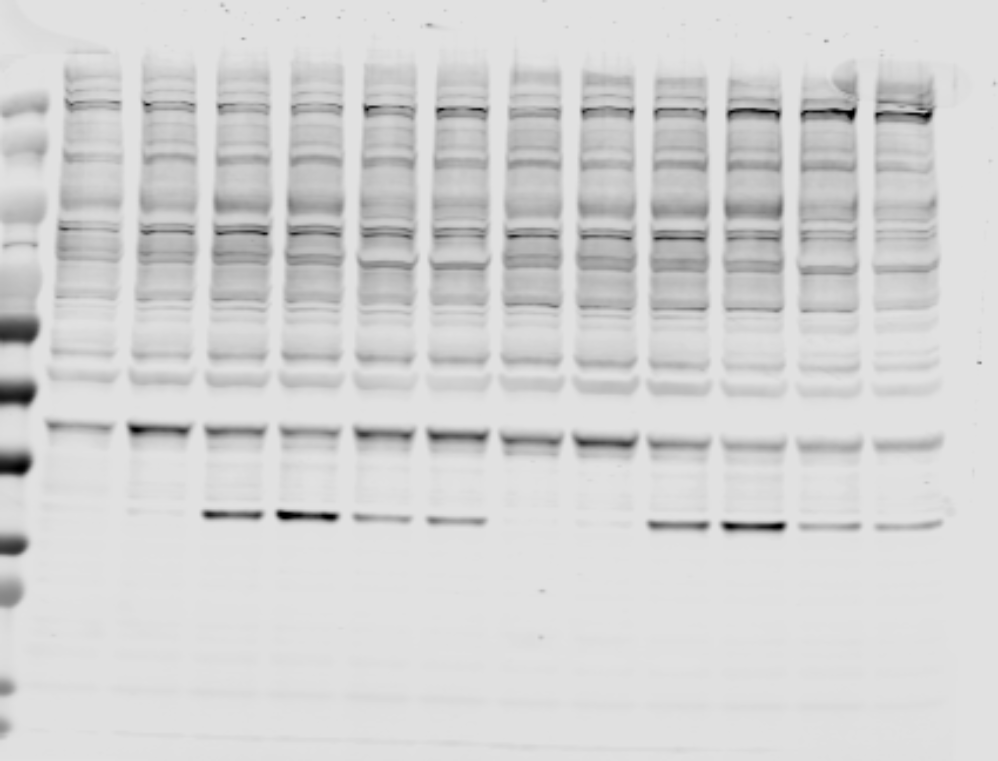

Supplement: Figure 3—source data 1. [file elife-102852-fig3-data1.zip › Figure 3-source data 1/Fig3F_P-eIF2a_original.tif]

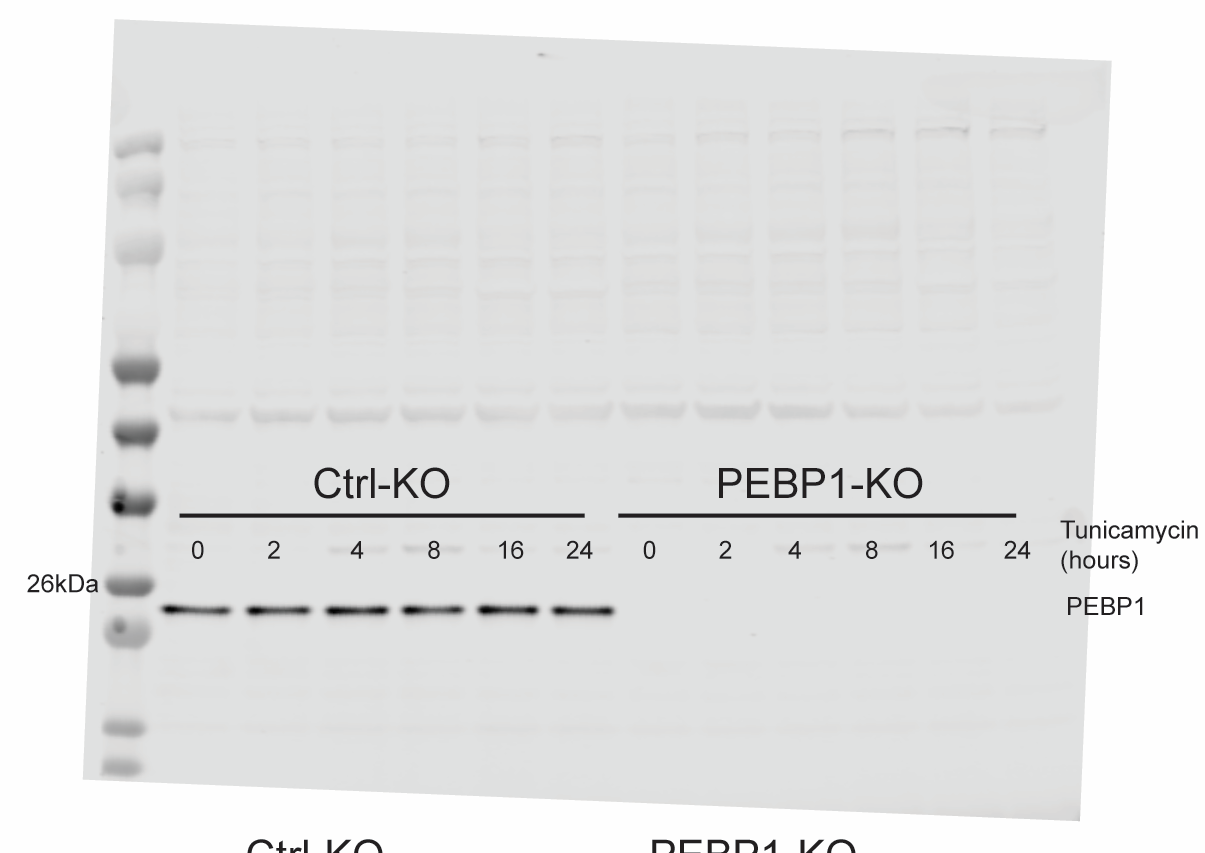

Supplement: Figure 3—source data 1. [file elife-102852-fig3-data1.zip › Figure 3-source data 1/Fig3F_PEBP1_band_indicated.tif]

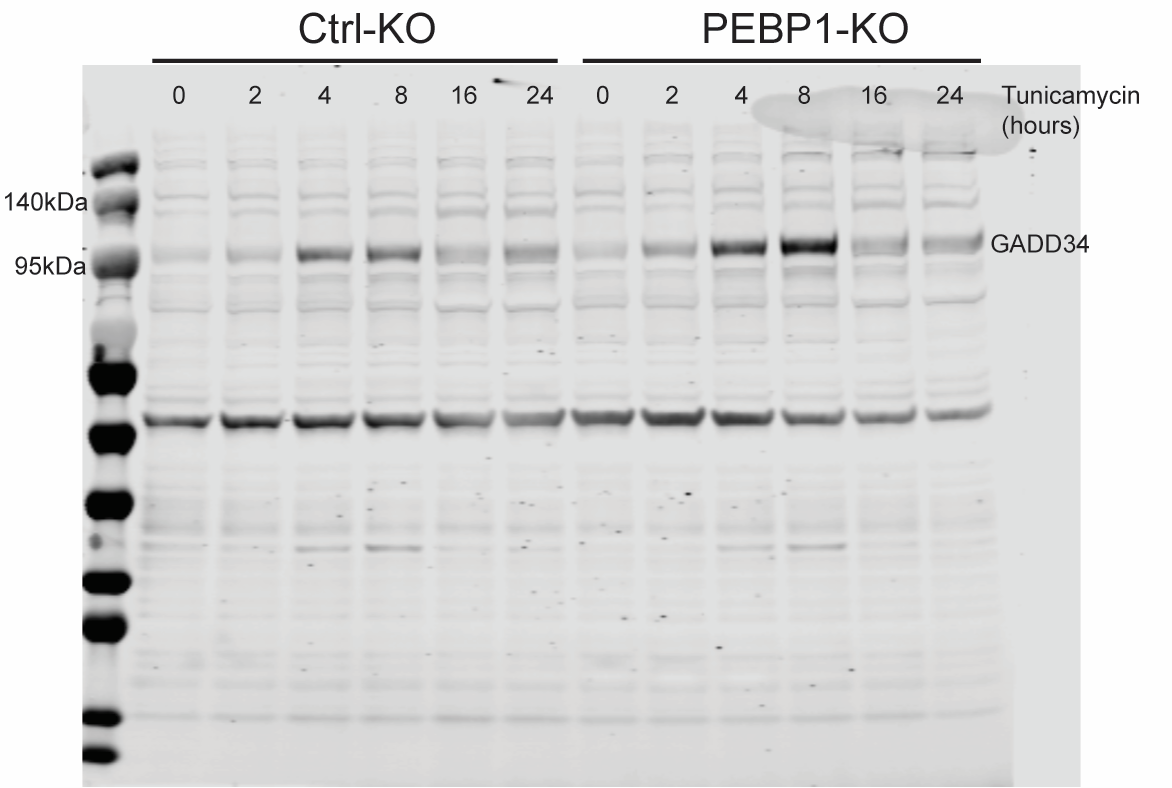

Supplement: Figure 3—source data 1. [file elife-102852-fig3-data1.zip › Figure 3-source data 1/Fig3F_GADD34_band_indicated.tif]

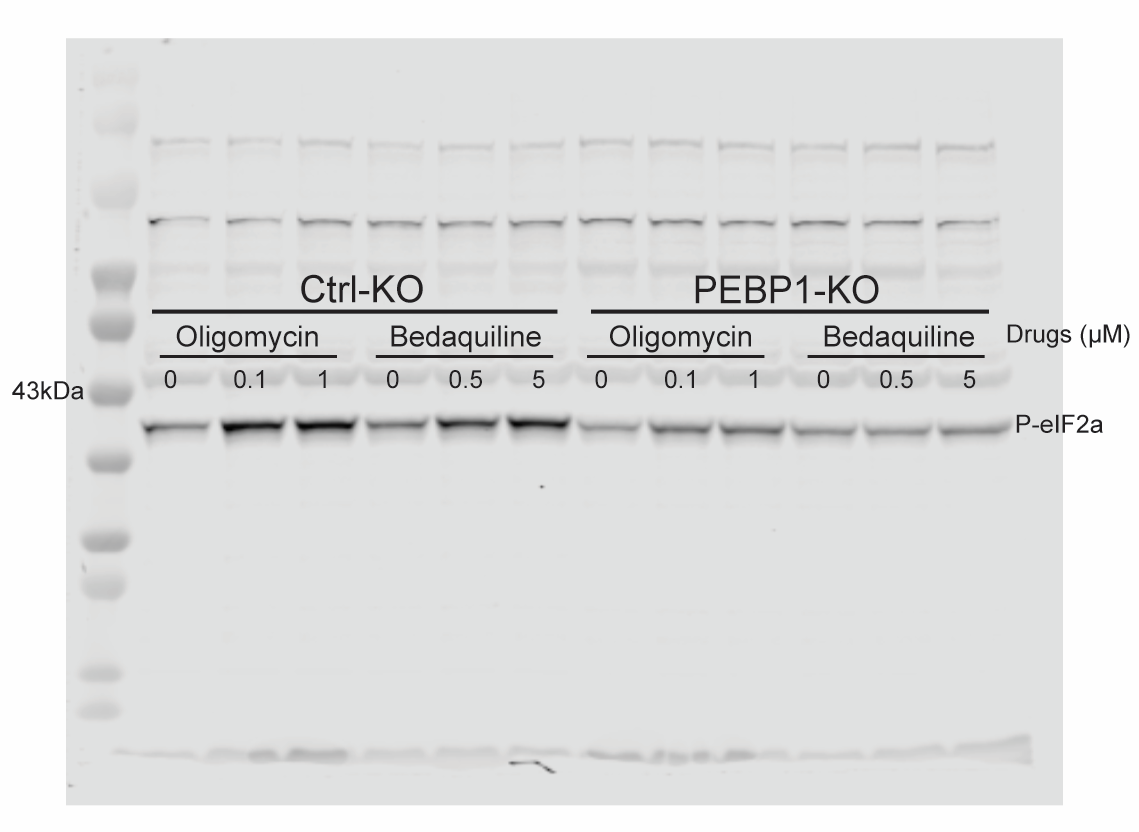

Supplement: Figure 3—figure supplement 1—source data 1. [file elife-102852-fig3-figsupp1-data1.zip › Figure 3-source data 2/Fig3Supplement1B_P-eIF2a_band_indicated.tif]

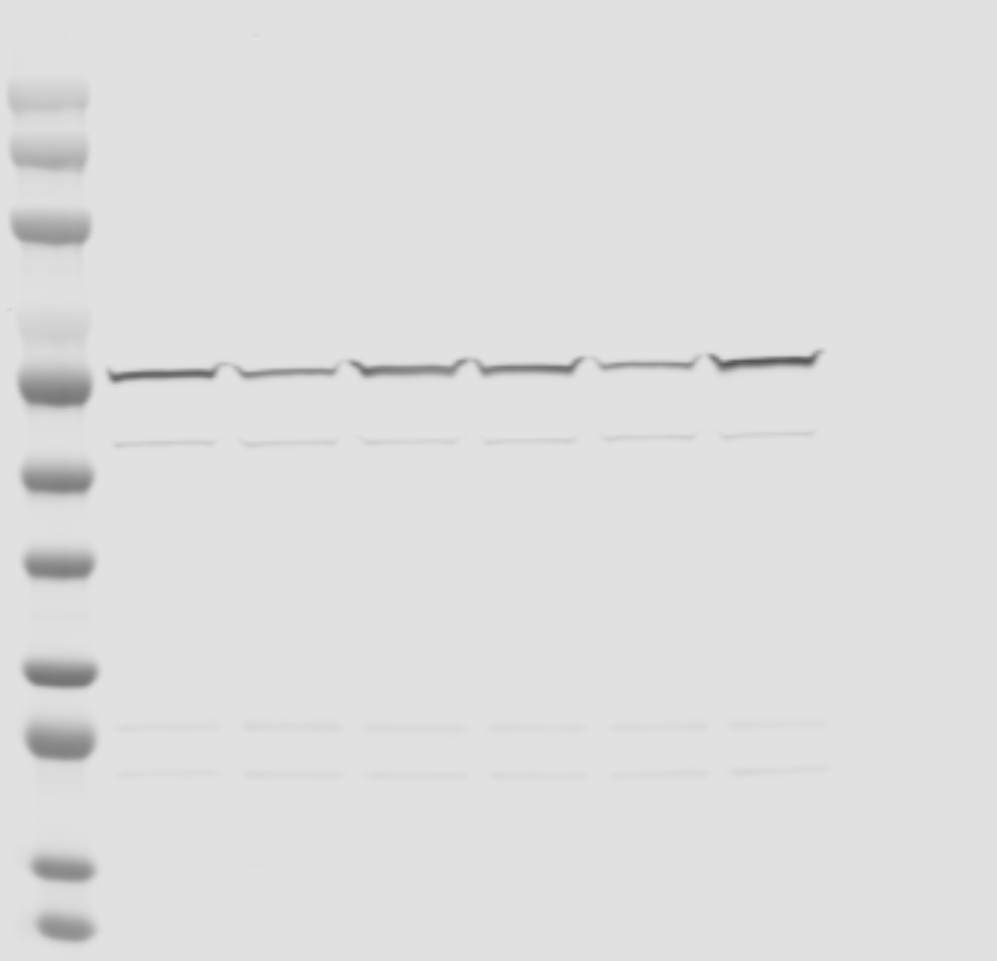

Supplement: Figure 3—figure supplement 1—source data 1. [file elife-102852-fig3-figsupp1-data1.zip › Figure 3-source data 2/Fig3Supplement1D_ATP5F1A_original.tif]

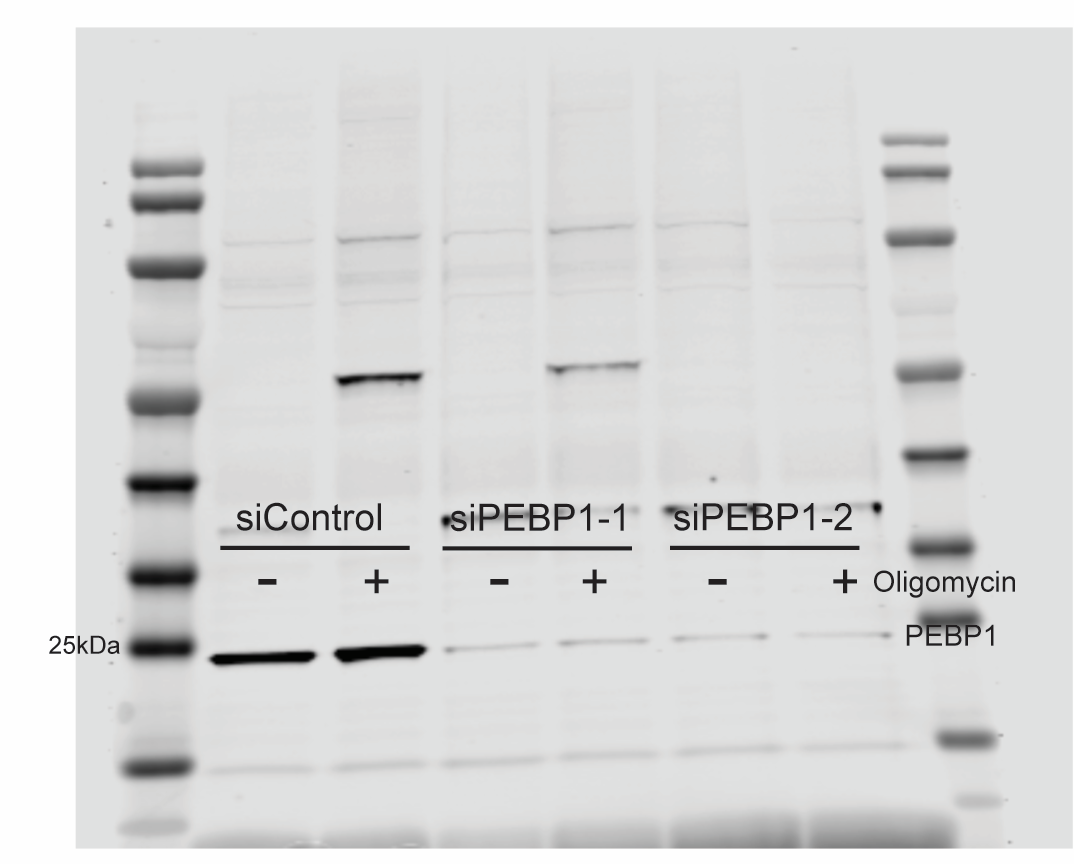

Supplement: Figure 3—figure supplement 1—source data 1. [file elife-102852-fig3-figsupp1-data1.zip › Figure 3-source data 2/Fig3Supplement1A_PEBP1_band_indicated.tif]

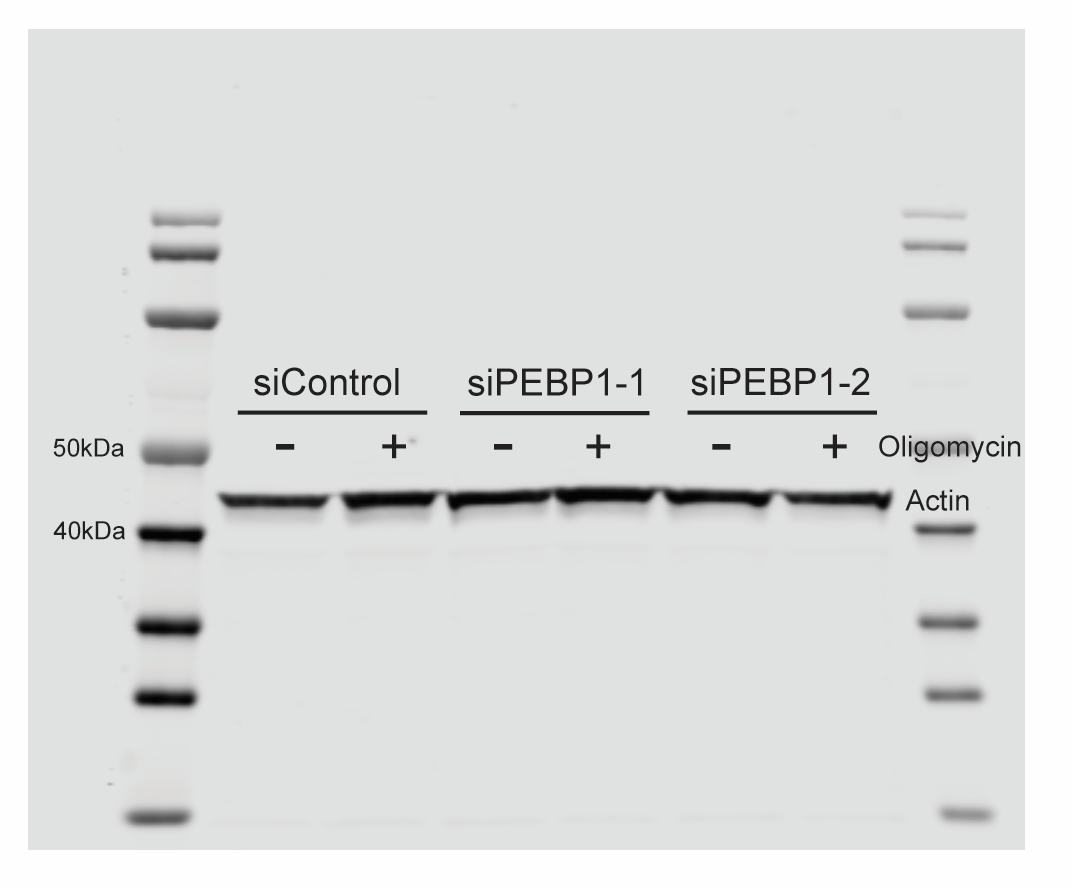

Supplement: Figure 3—figure supplement 1—source data 1. [file elife-102852-fig3-figsupp1-data1.zip › Figure 3-source data 2/Fig3Supplement1A_Actin_band_indicated.tif]

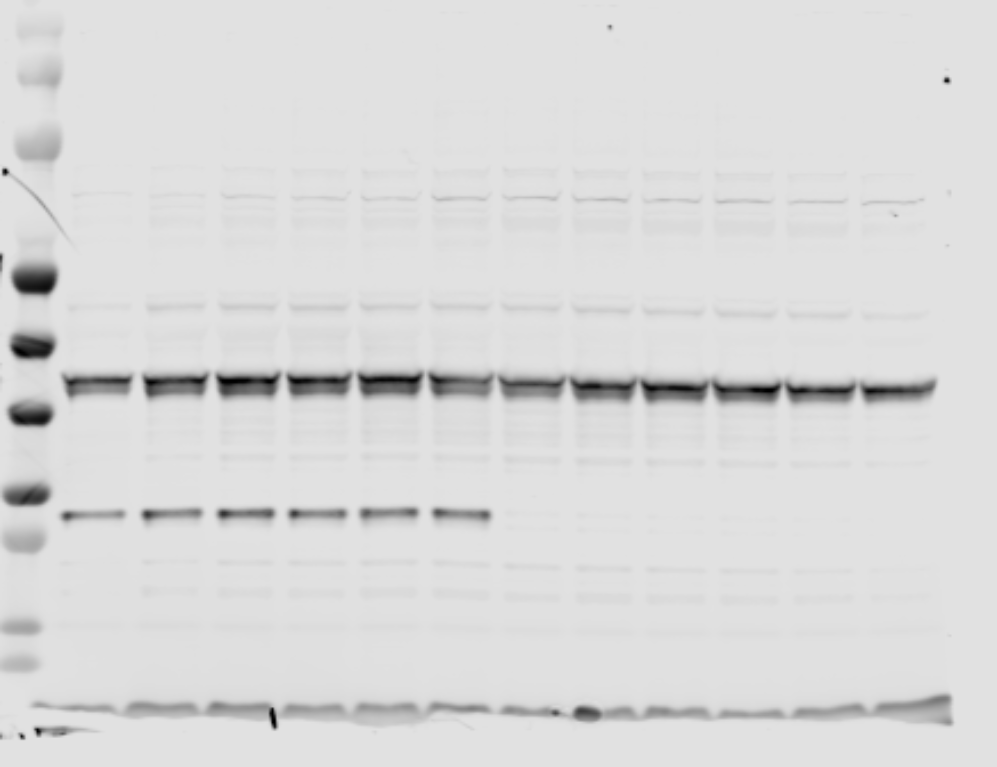

Supplement: Figure 3—figure supplement 1—source data 1. [file elife-102852-fig3-figsupp1-data1.zip › Figure 3-source data 2/Fig3Supplement1B_eIF2a_PEBP1_original.tif]

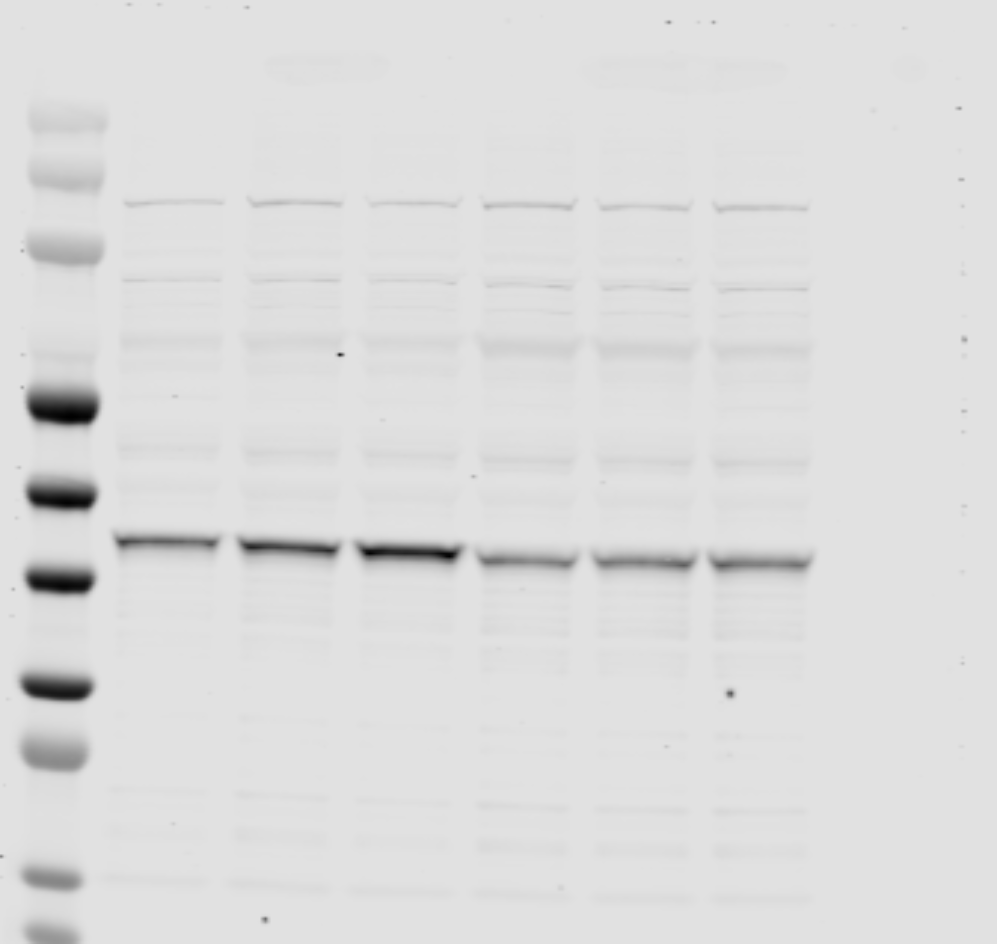

Supplement: Figure 3—figure supplement 1—source data 1. [file elife-102852-fig3-figsupp1-data1.zip › Figure 3-source data 2/Fig3Supplement1D_P-eIF2a_original.tif]

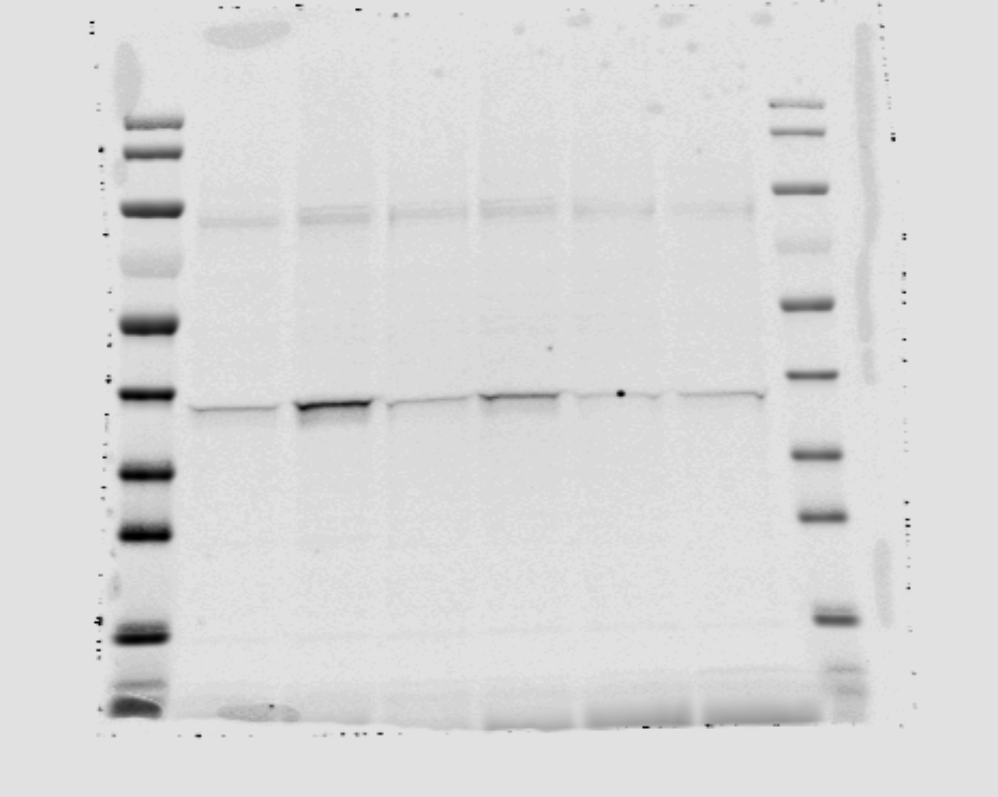

Supplement: Figure 3—figure supplement 1—source data 1. [file elife-102852-fig3-figsupp1-data1.zip › Figure 3-source data 2/Fig3Supplement1A_P-eIF2a_original.tif]

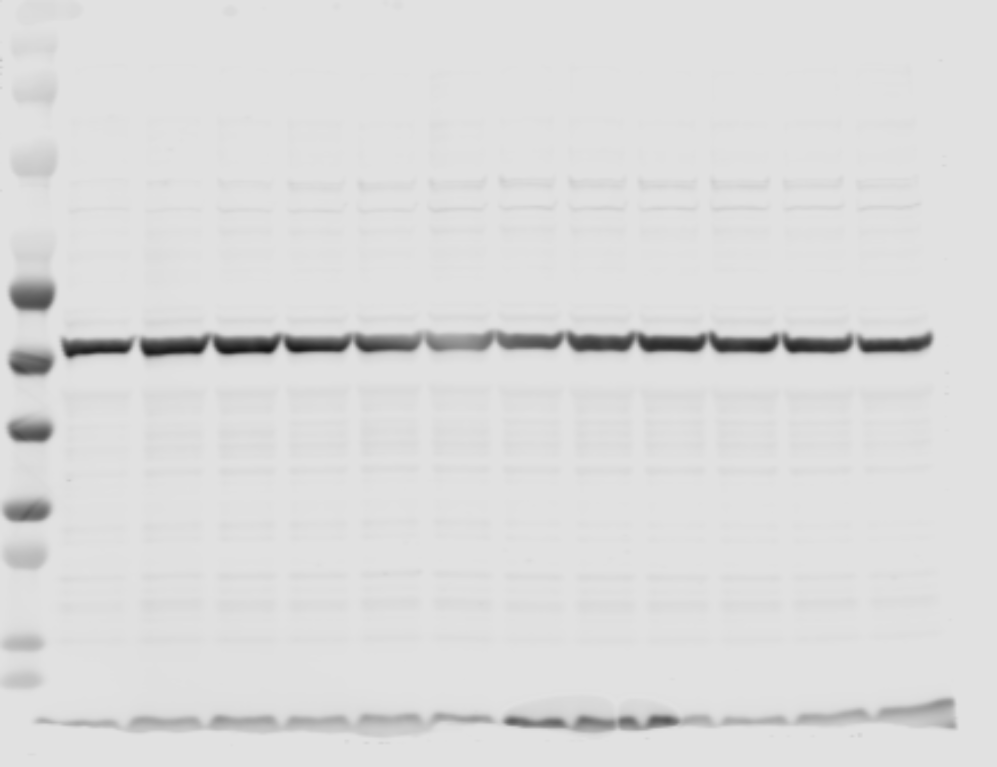

Supplement: Figure 3—figure supplement 1—source data 1. [file elife-102852-fig3-figsupp1-data1.zip › Figure 3-source data 2/Fig3Supplement1B_Actin_original.tif]

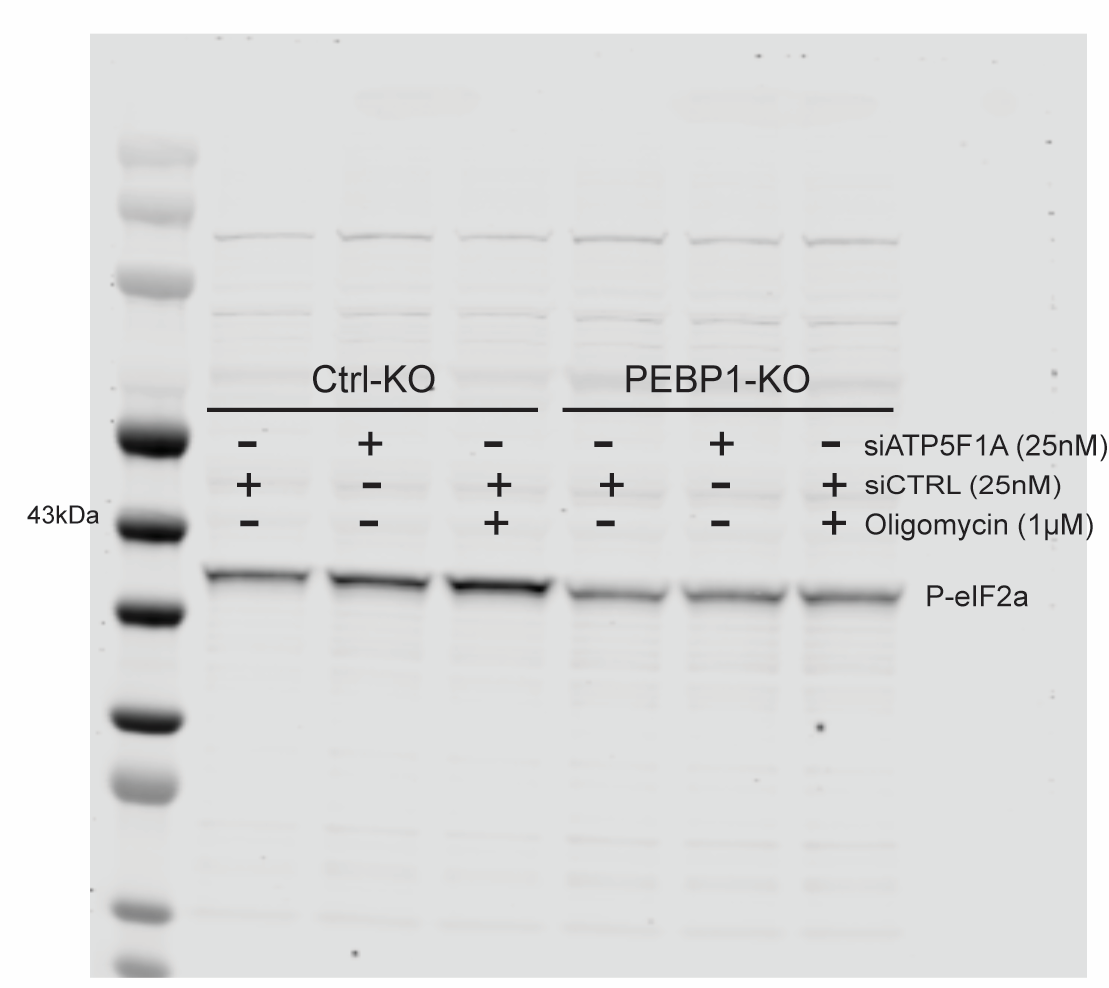

Supplement: Figure 3—figure supplement 1—source data 1. [file elife-102852-fig3-figsupp1-data1.zip › Figure 3-source data 2/Fig3Supplement1D_P-eIF2a_band_indicated.tif]

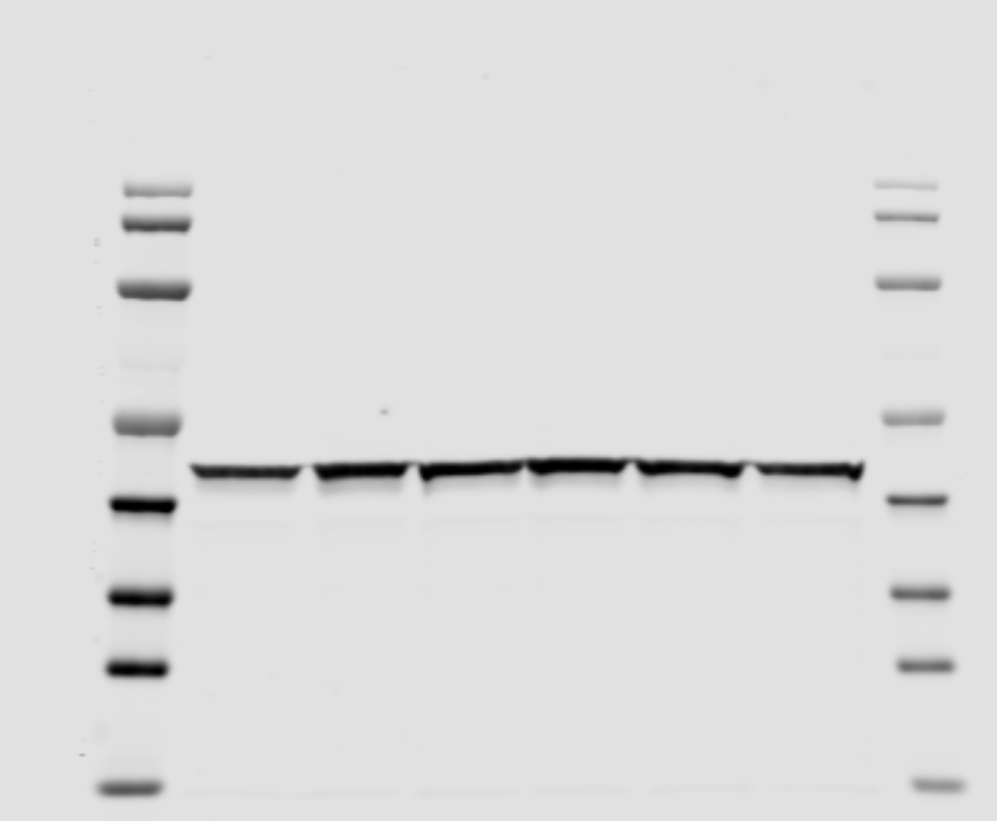

Supplement: Figure 3—figure supplement 1—source data 1. [file elife-102852-fig3-figsupp1-data1.zip › Figure 3-source data 2/Fig3Supplement1A_Actin_original.tif]
