# Supplementary figures and images for: PEBP1 amplifies mitochondrial dysfunction-induced integrated stress response (part 2 of 3)
Source: eLife. 2025 Jan 29;13:RP102852. doi: 10.7554/eLife.102852 (PMC11778924; doi:10.7554/eLife.102852)

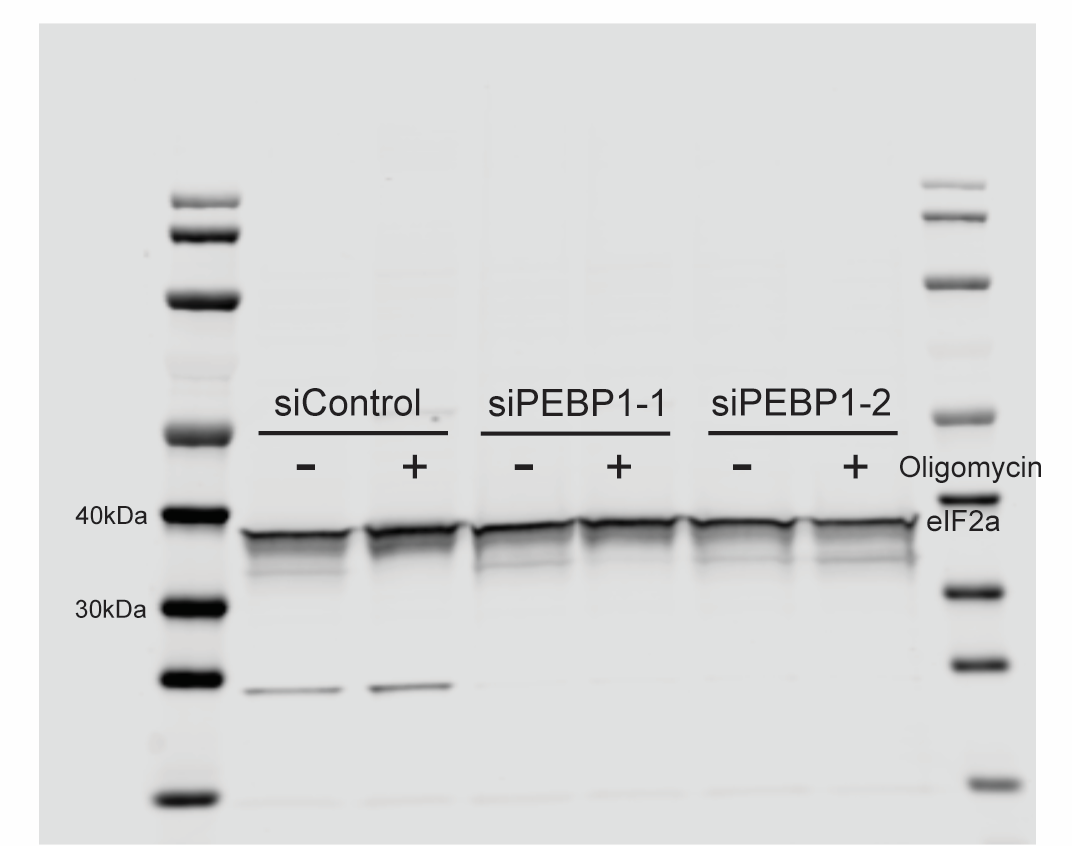

Supplement: Figure 3—figure supplement 1—source data 1. [file elife-102852-fig3-figsupp1-data1.zip › Figure 3-source data 2/Fig3Supplement1A_eIF2a_band_indicated.tif]

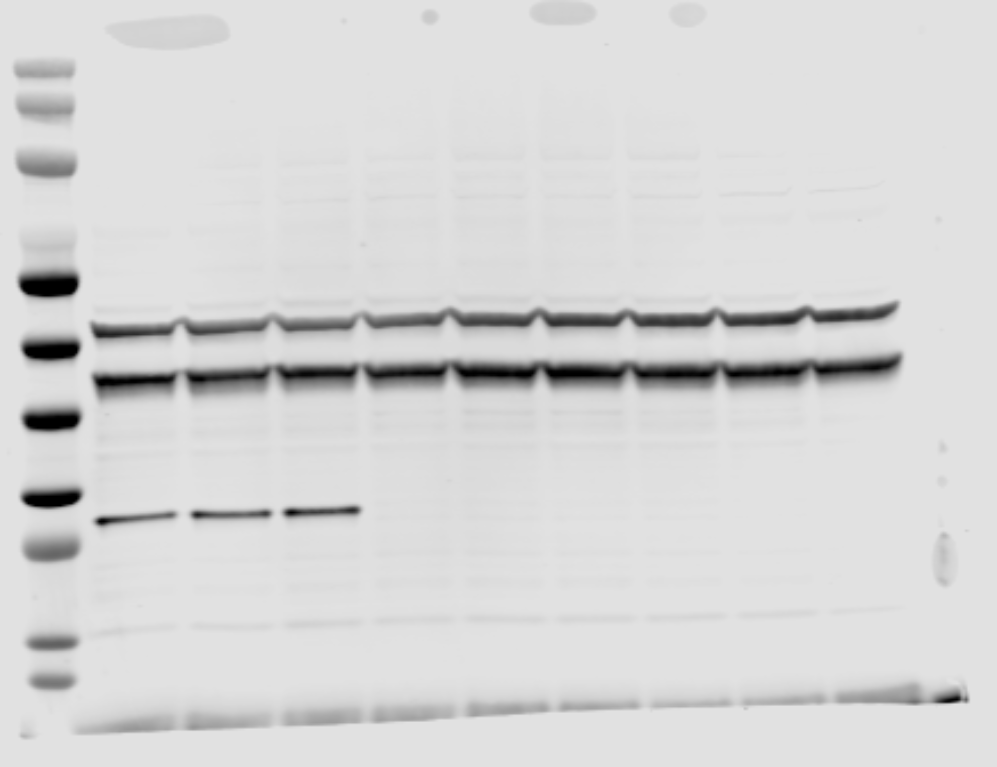

Supplement: Figure 3—figure supplement 1—source data 1. [file elife-102852-fig3-figsupp1-data1.zip › Figure 3-source data 2/Fig3Supplement1C_Actin_eIF2a_PEBP1_original.tif]

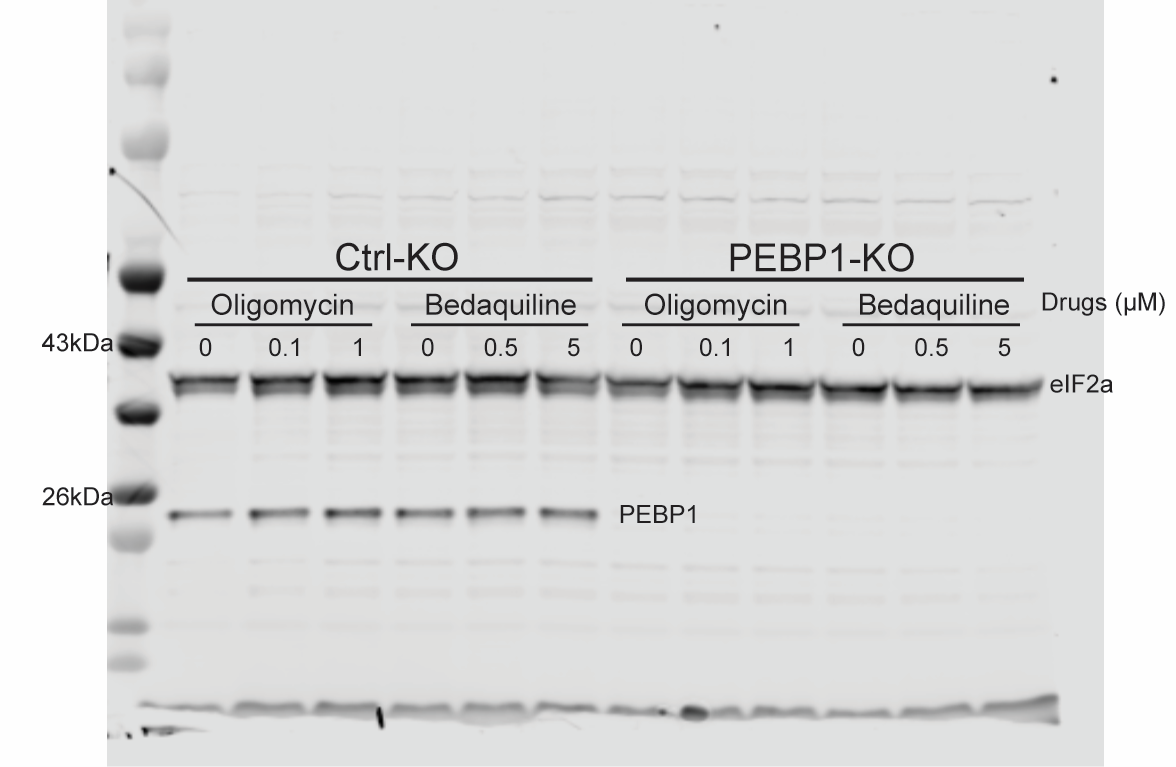

Supplement: Figure 3—figure supplement 1—source data 1. [file elife-102852-fig3-figsupp1-data1.zip › Figure 3-source data 2/Fig3Supplement1B_eIF2a_PEBP1_bands_indicated.tif]

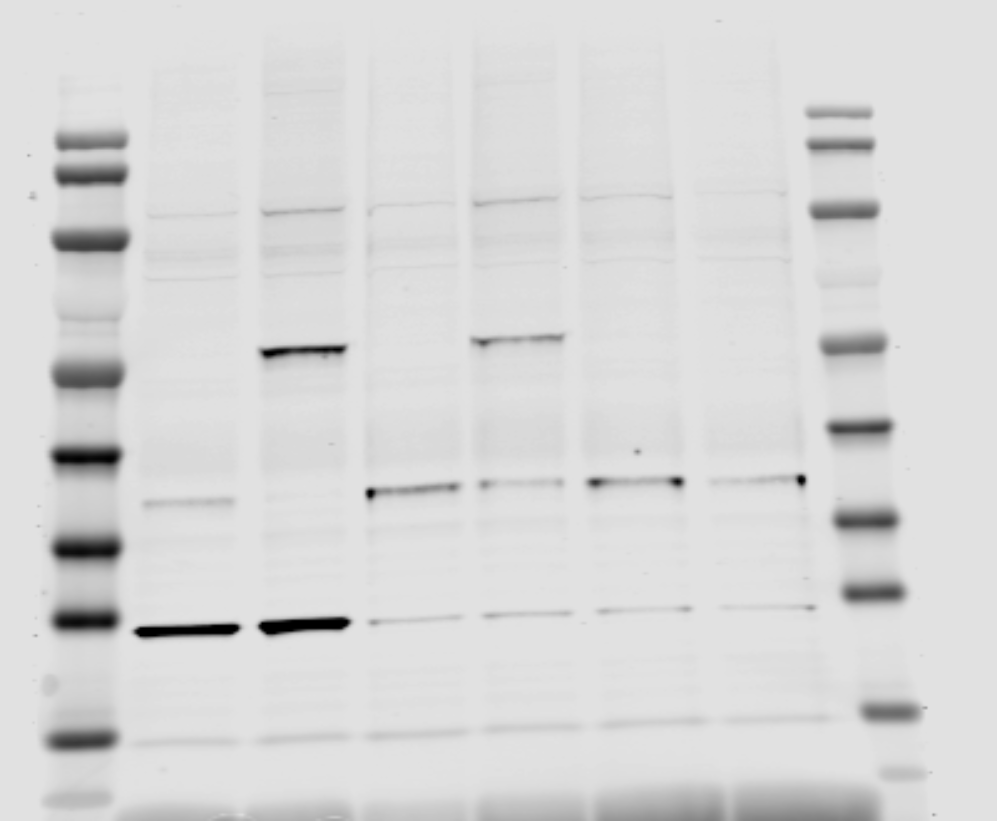

Supplement: Figure 3—figure supplement 1—source data 1. [file elife-102852-fig3-figsupp1-data1.zip › Figure 3-source data 2/Fig3Supplement1A_PEBP1_original.tif]

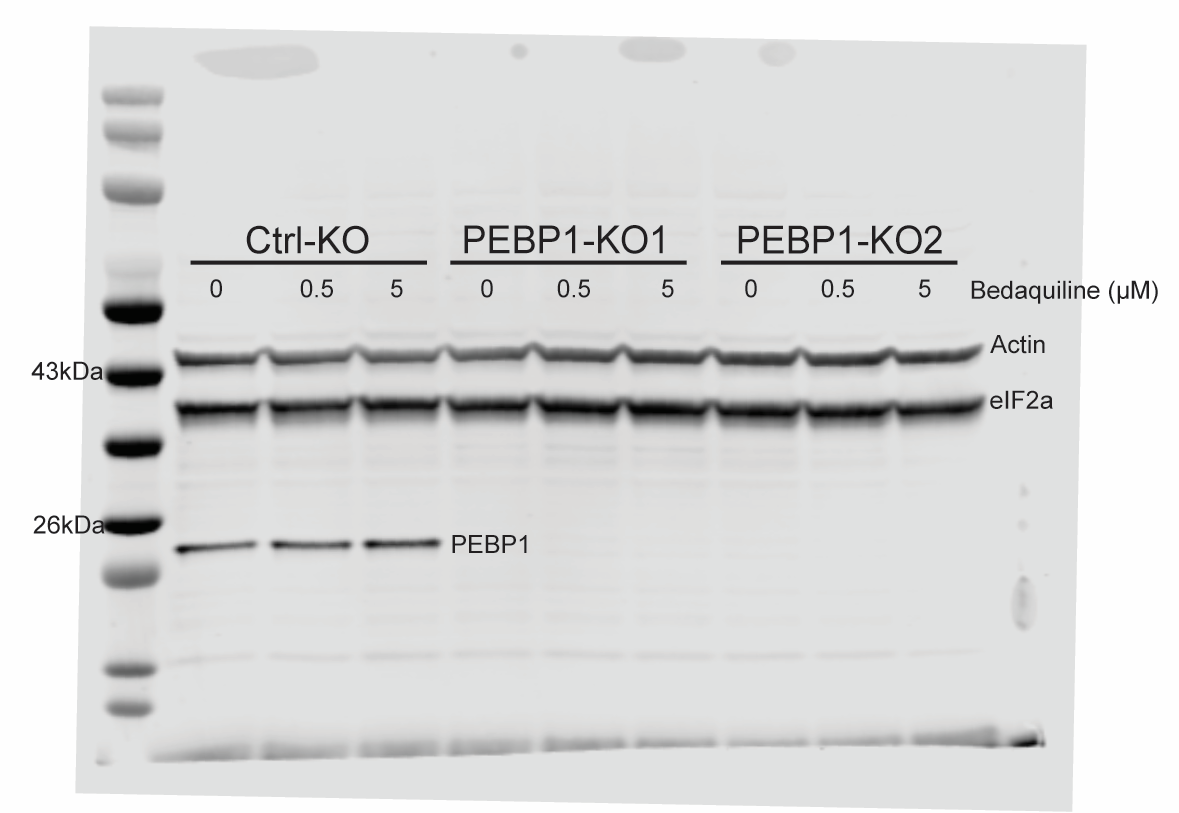

Supplement: Figure 3—figure supplement 1—source data 1. [file elife-102852-fig3-figsupp1-data1.zip › Figure 3-source data 2/Fig3Supplement1C_Actin_eIF2a_PEBP1_bands_indicated.tif]

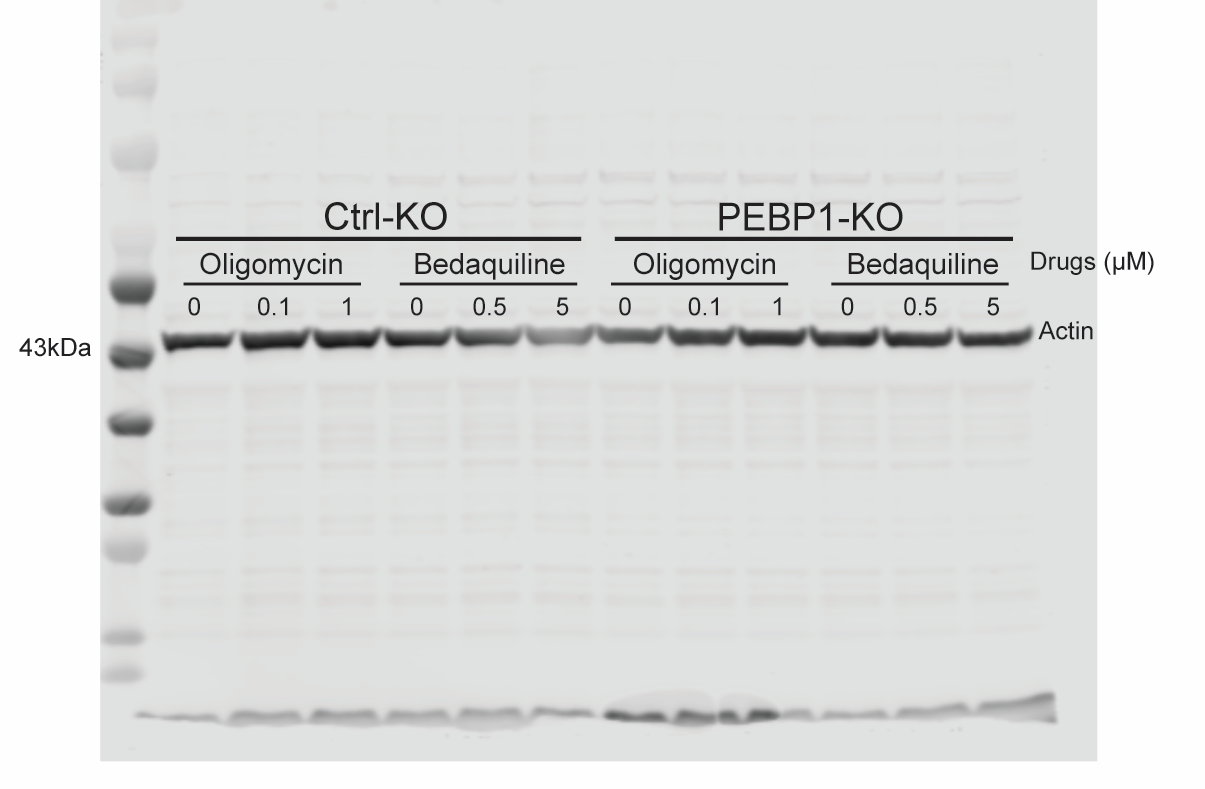

Supplement: Figure 3—figure supplement 1—source data 1. [file elife-102852-fig3-figsupp1-data1.zip › Figure 3-source data 2/Fig3Supplement1B_Actin_band_indicated.tif]

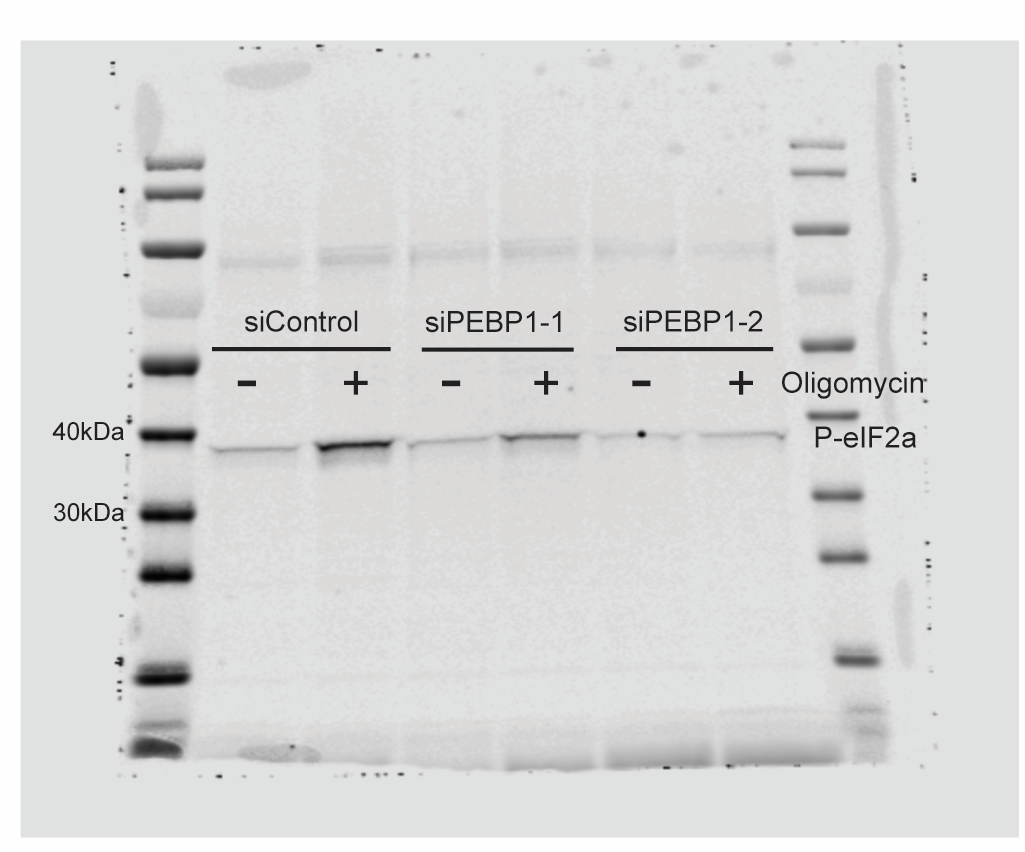

Supplement: Figure 3—figure supplement 1—source data 1. [file elife-102852-fig3-figsupp1-data1.zip › Figure 3-source data 2/Fig3Supplement1A_P-eIF2a_band_indicated.tif]

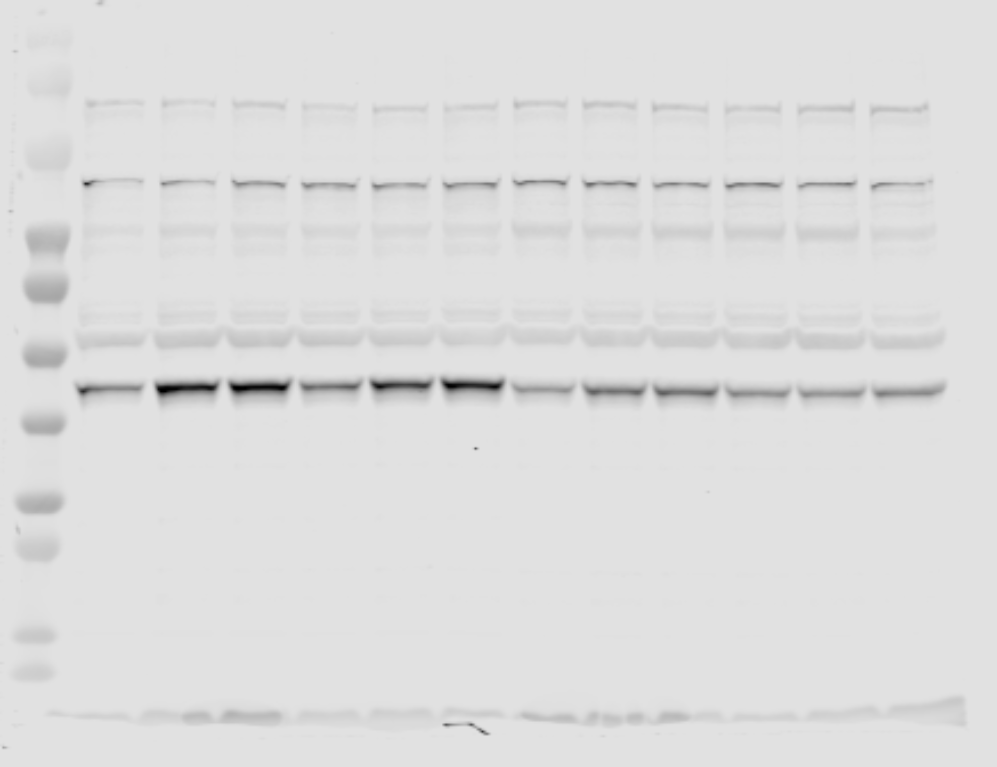

Supplement: Figure 3—figure supplement 1—source data 1. [file elife-102852-fig3-figsupp1-data1.zip › Figure 3-source data 2/Fig3Supplement1B_P-eIF2a_original.tif]

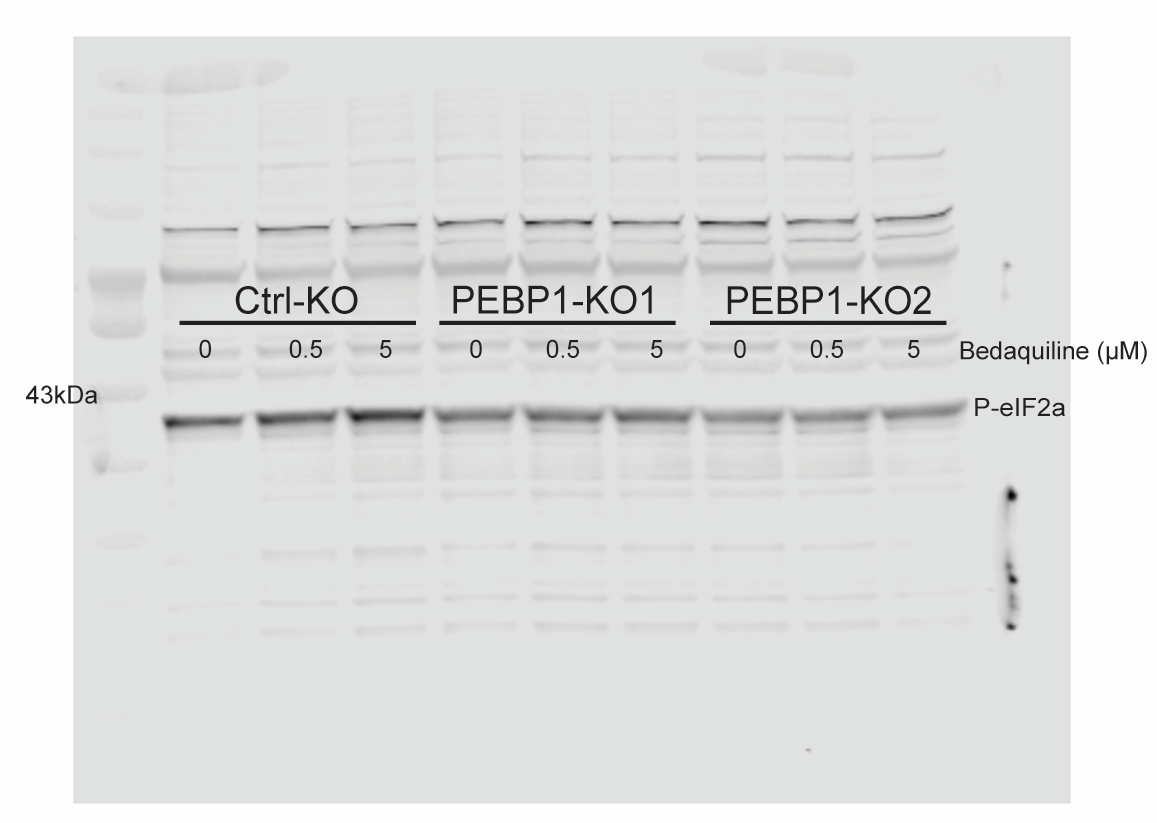

Supplement: Figure 3—figure supplement 1—source data 1. [file elife-102852-fig3-figsupp1-data1.zip › Figure 3-source data 2/Fig3Supplement1C_P-eIF2a_bands_indicated.tif]

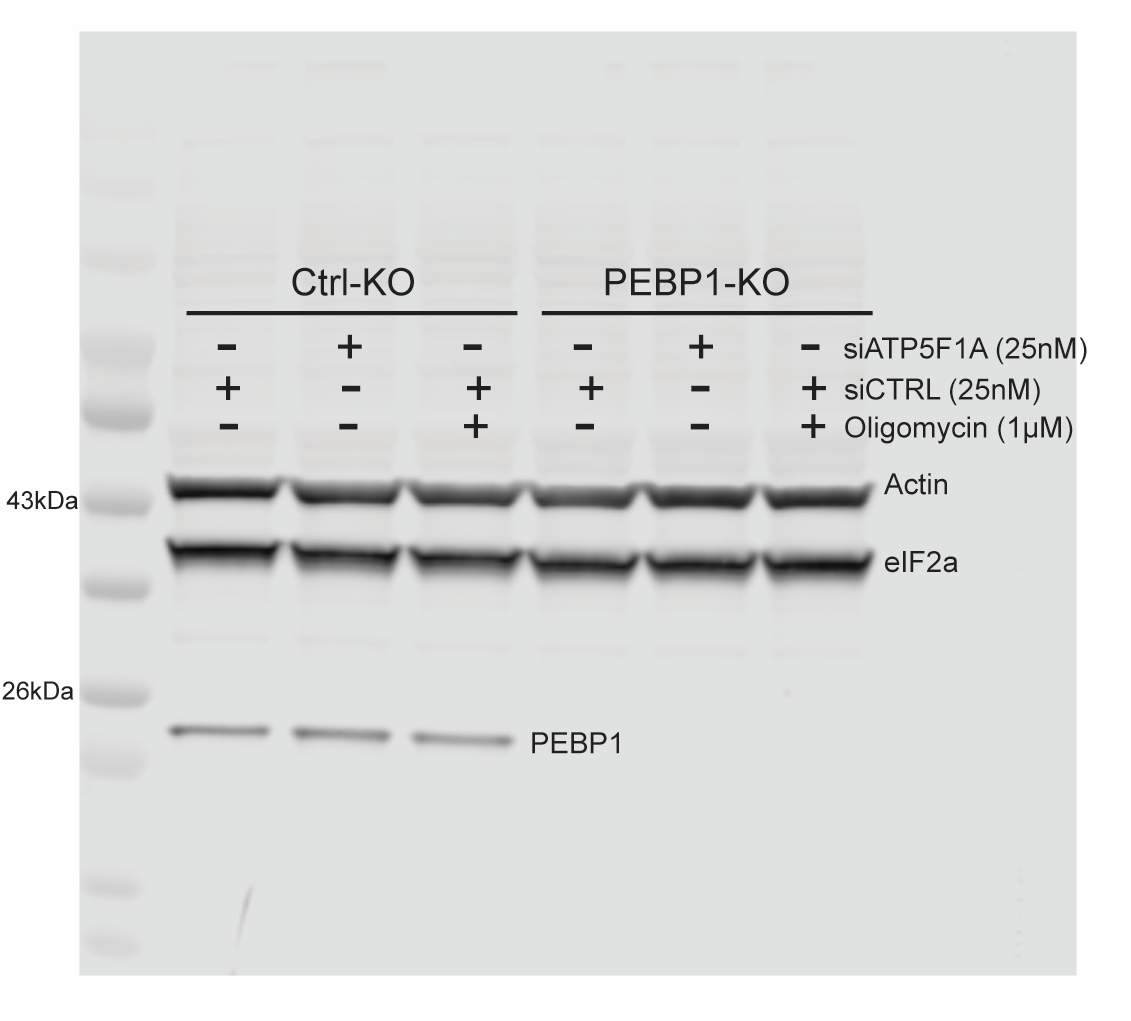

Supplement: Figure 3—figure supplement 1—source data 1. [file elife-102852-fig3-figsupp1-data1.zip › Figure 3-source data 2/Fig3Supplement1D_Actin_eIF2a_PEBP1_bands_indicated.tif]

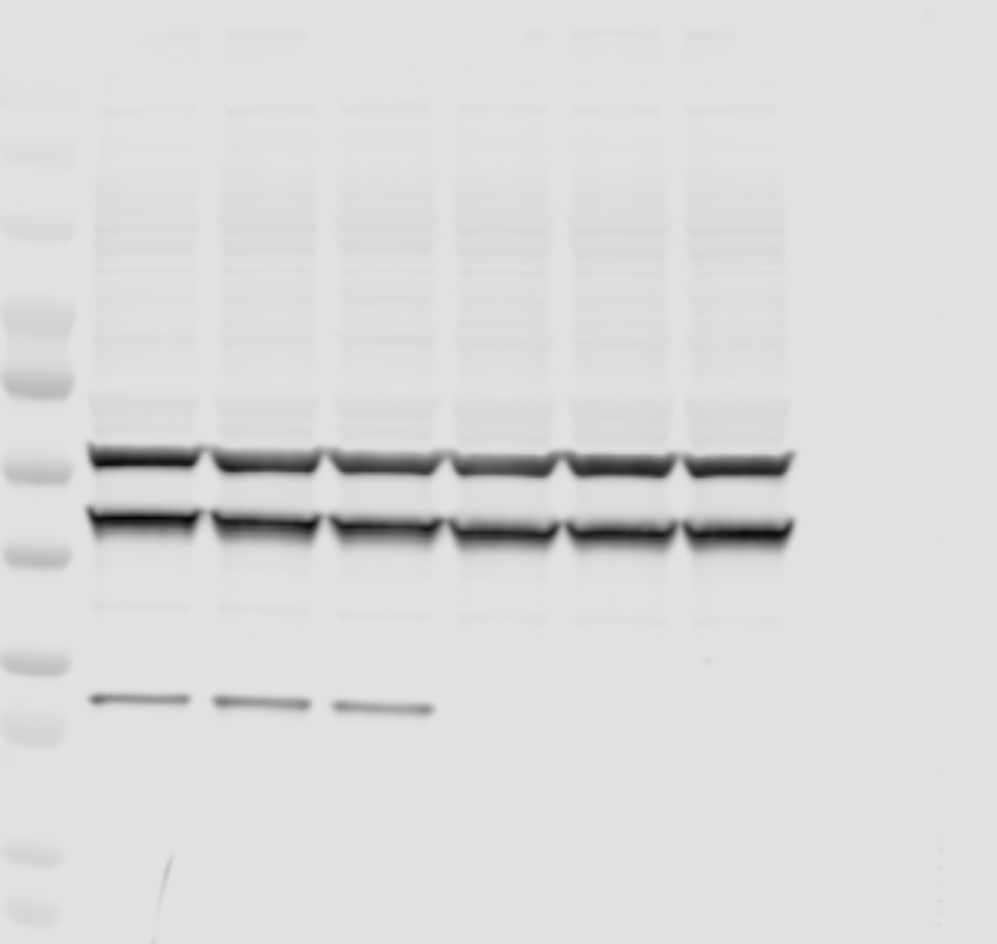

Supplement: Figure 3—figure supplement 1—source data 1. [file elife-102852-fig3-figsupp1-data1.zip › Figure 3-source data 2/Fig3Supplement1D_Actin_eIF2a_PEBP1_original.tif]

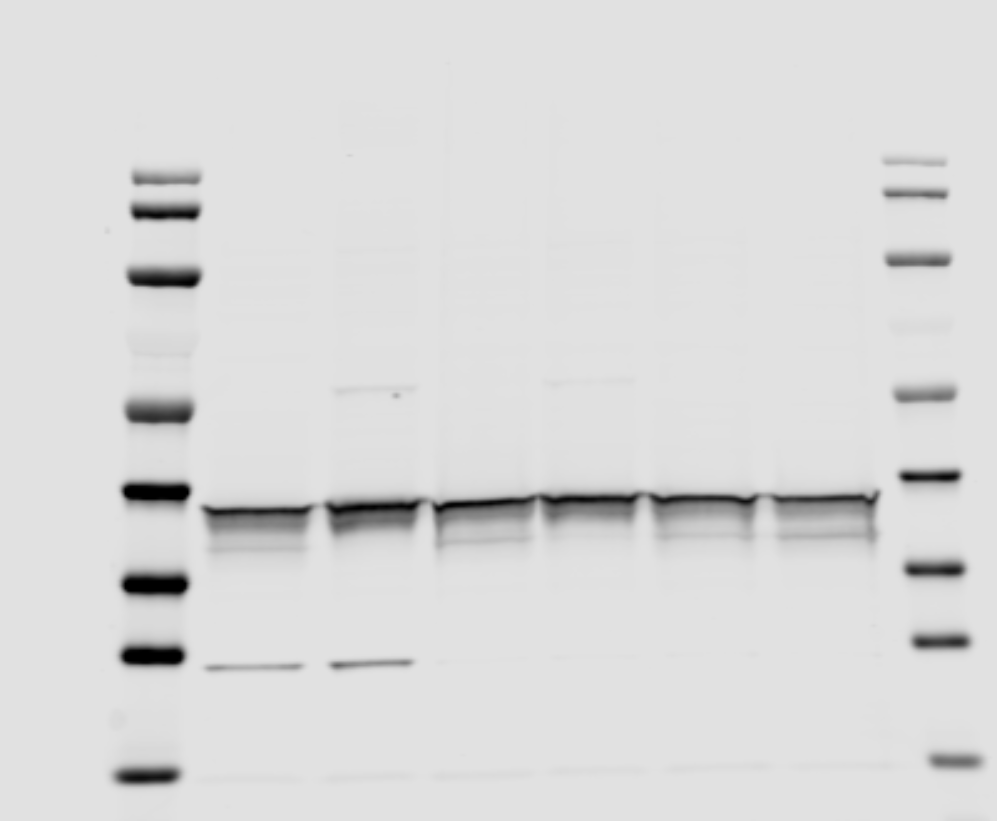

Supplement: Figure 3—figure supplement 1—source data 1. [file elife-102852-fig3-figsupp1-data1.zip › Figure 3-source data 2/Fig3Supplement1A_eIF2a_original.tif]

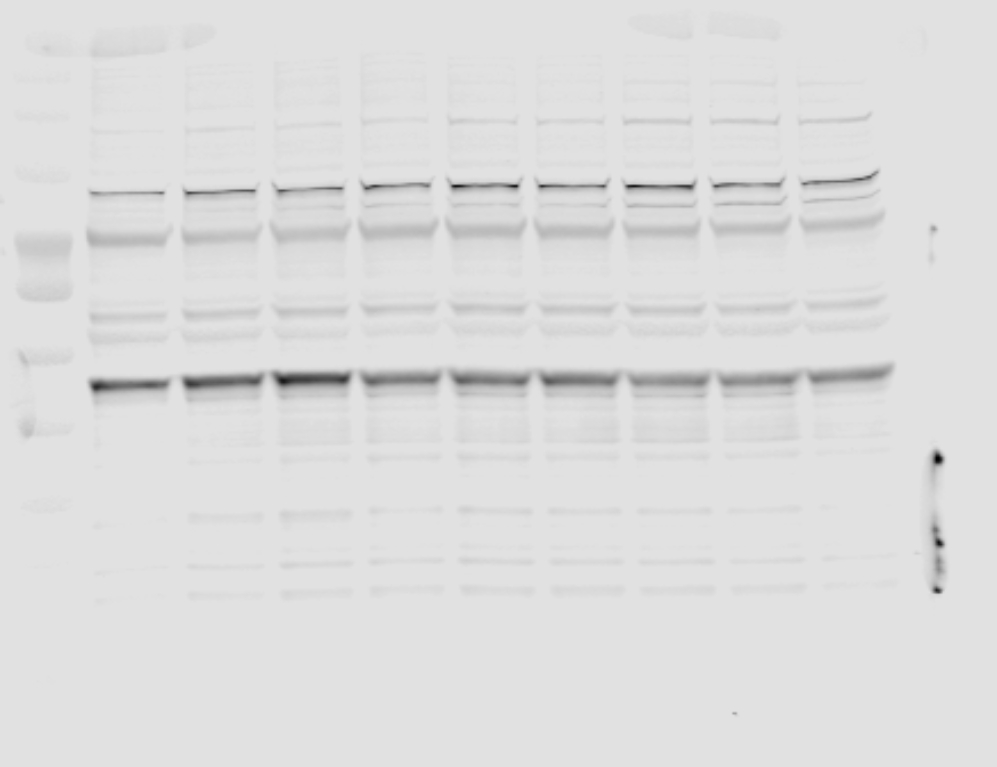

Supplement: Figure 3—figure supplement 1—source data 1. [file elife-102852-fig3-figsupp1-data1.zip › Figure 3-source data 2/Fig3Supplement1C_P-eIF2a_original.tif]

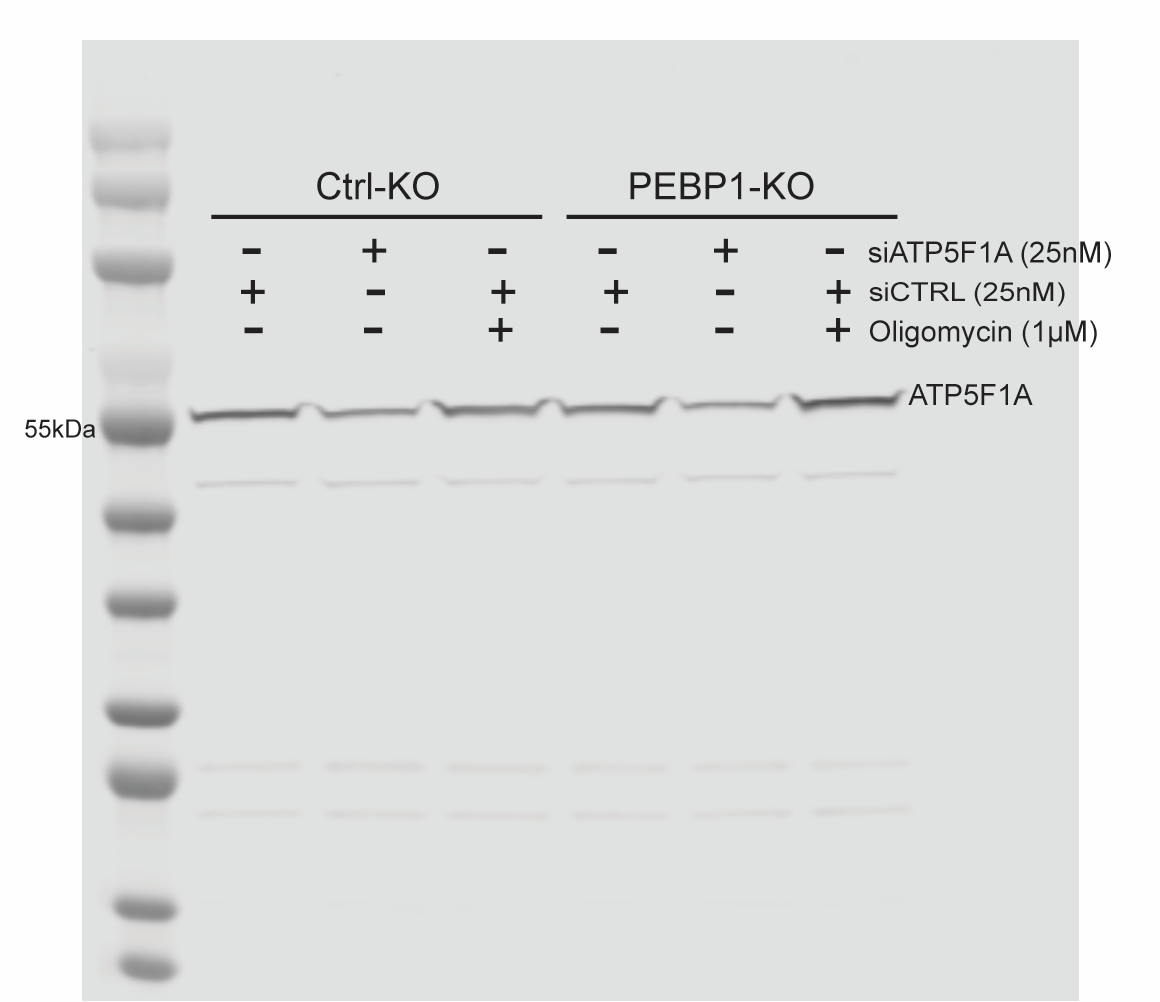

Supplement: Figure 3—figure supplement 1—source data 1. [file elife-102852-fig3-figsupp1-data1.zip › Figure 3-source data 2/Fig3Supplement1D_ATP5F1A_band_indicated.tif]

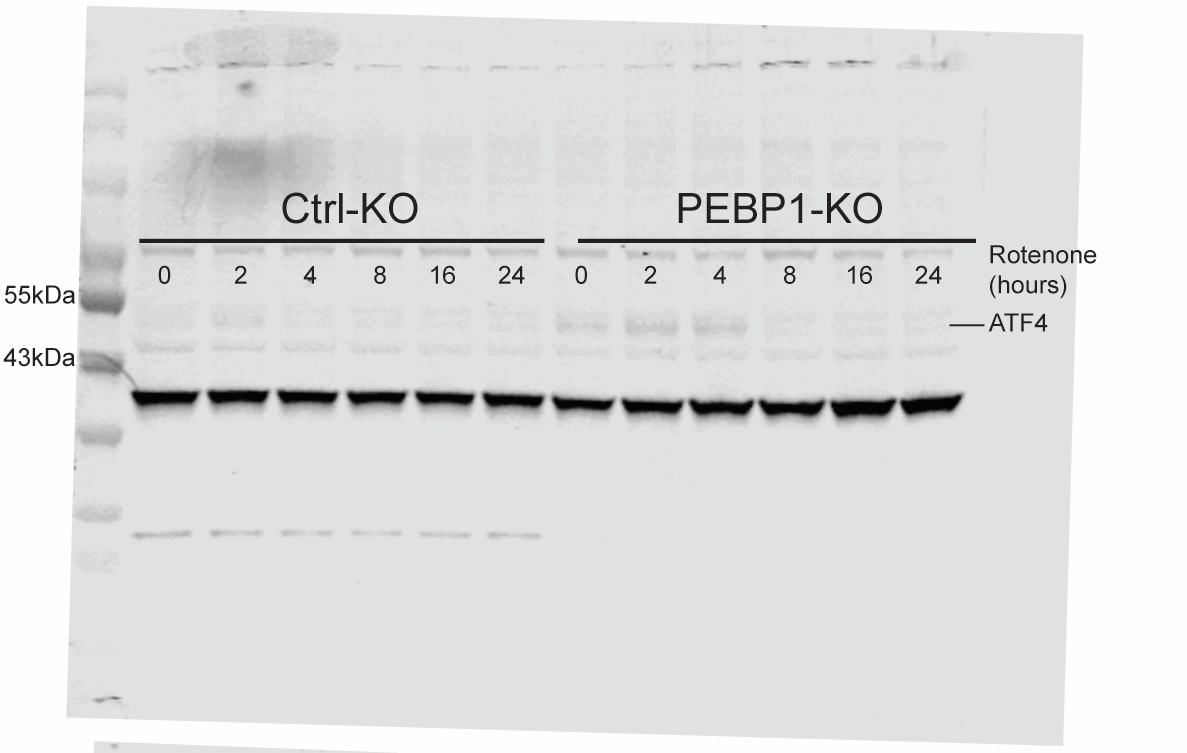

Supplement: Figure 3—figure supplement 2—source data 1. [file elife-102852-fig3-figsupp2-data1.zip › Figure 3-source data 3/Fig3Supplement2E_ATF4_band_indicated.tif]

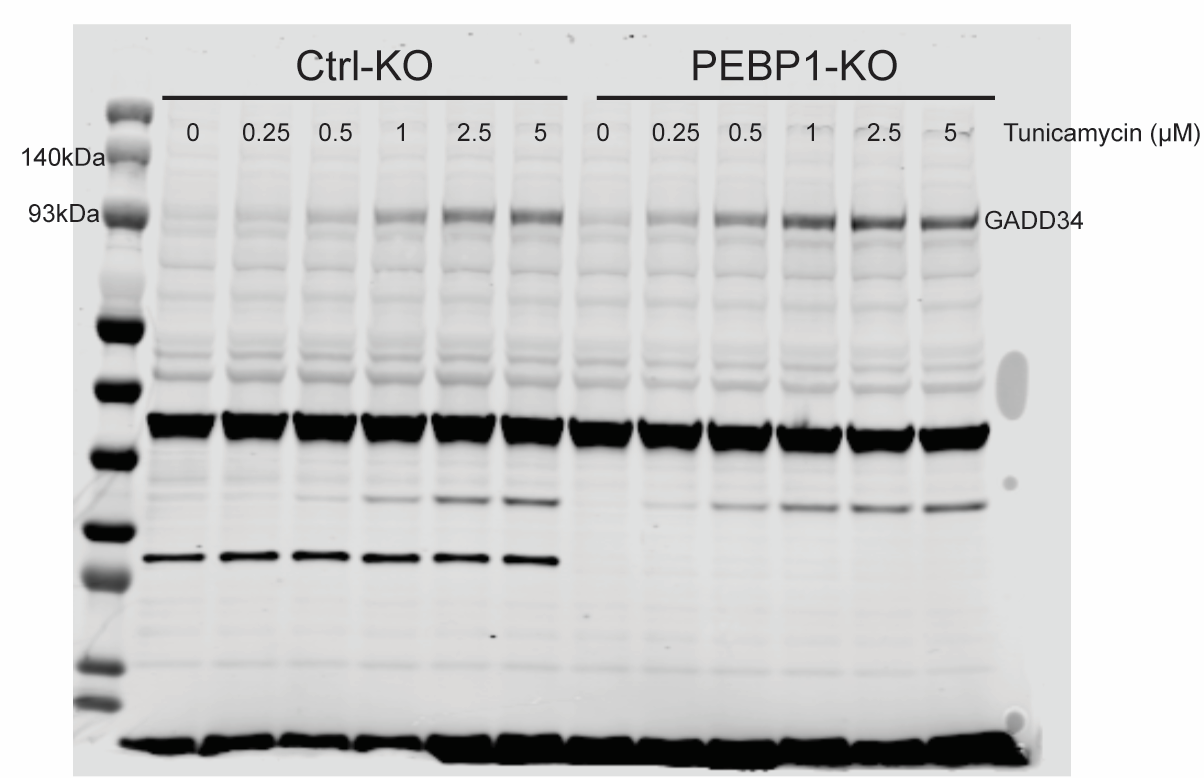

Supplement: Figure 3—figure supplement 2—source data 1. [file elife-102852-fig3-figsupp2-data1.zip › Figure 3-source data 3/Fig3Supplement2G_GADD34_band_indicated.tif]

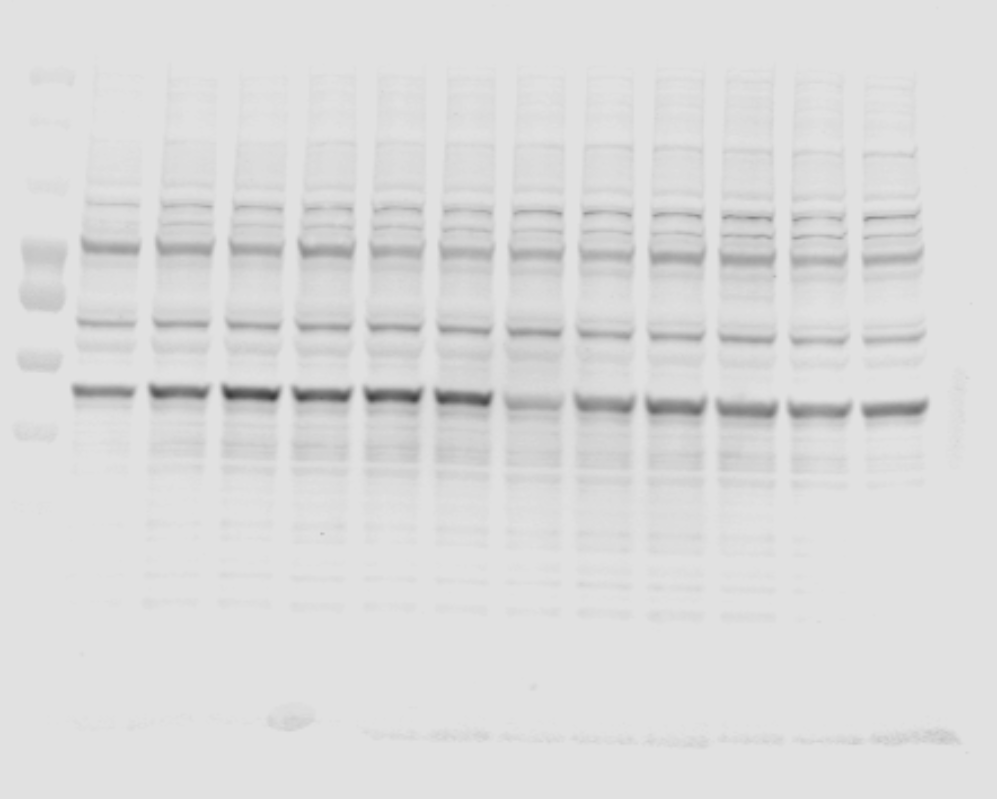

Supplement: Figure 3—figure supplement 2—source data 1. [file elife-102852-fig3-figsupp2-data1.zip › Figure 3-source data 3/Fig3Supplement2G_P-eIF2a_original.tif]

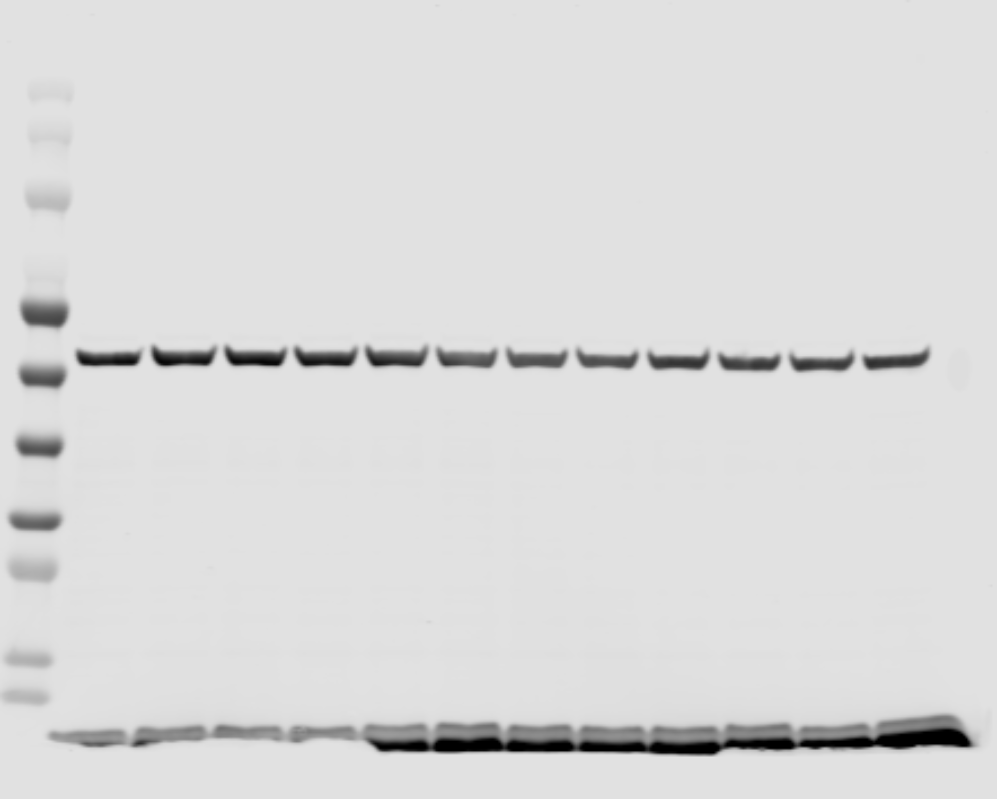

Supplement: Figure 3—figure supplement 2—source data 1. [file elife-102852-fig3-figsupp2-data1.zip › Figure 3-source data 3/Fig3Supplement2G_Actin_original.tif]

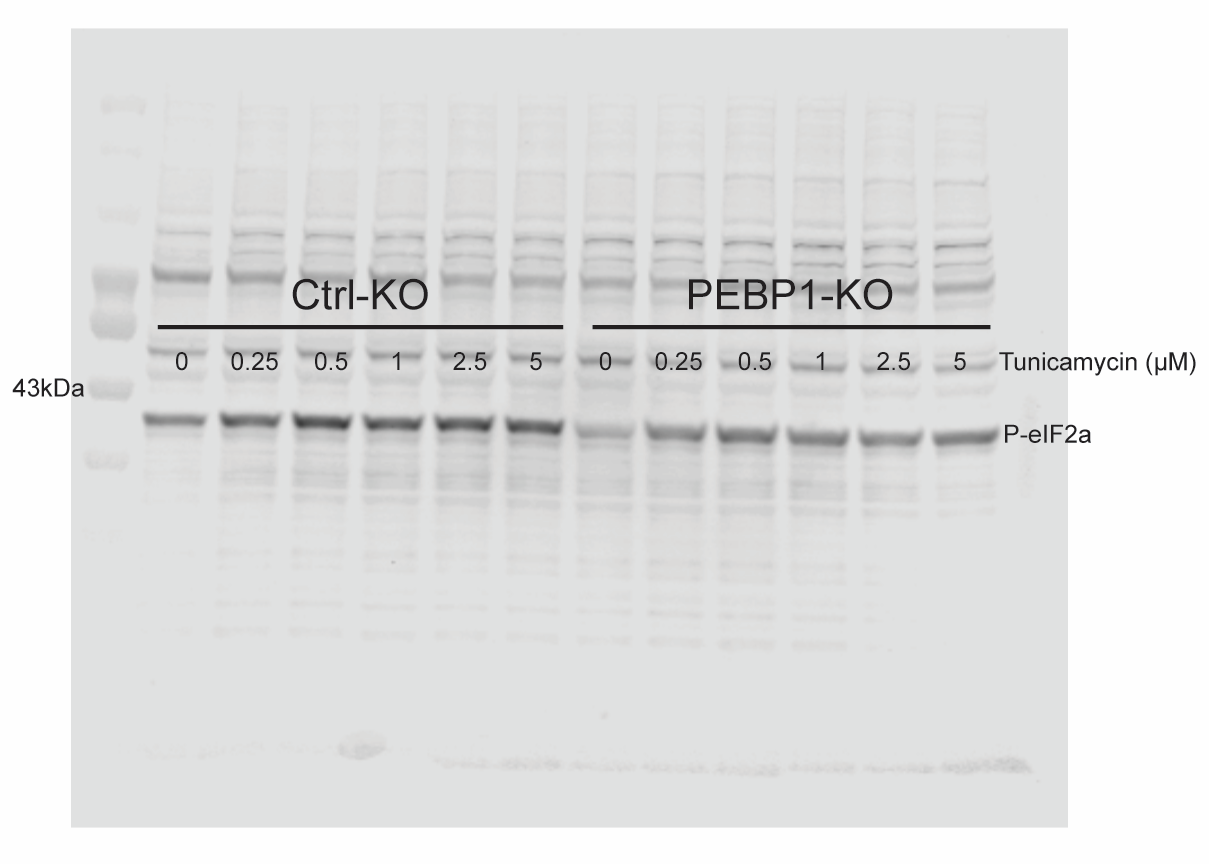

Supplement: Figure 3—figure supplement 2—source data 1. [file elife-102852-fig3-figsupp2-data1.zip › Figure 3-source data 3/Fig3Supplement2G_P-eIF2a_band_indicated.tif]

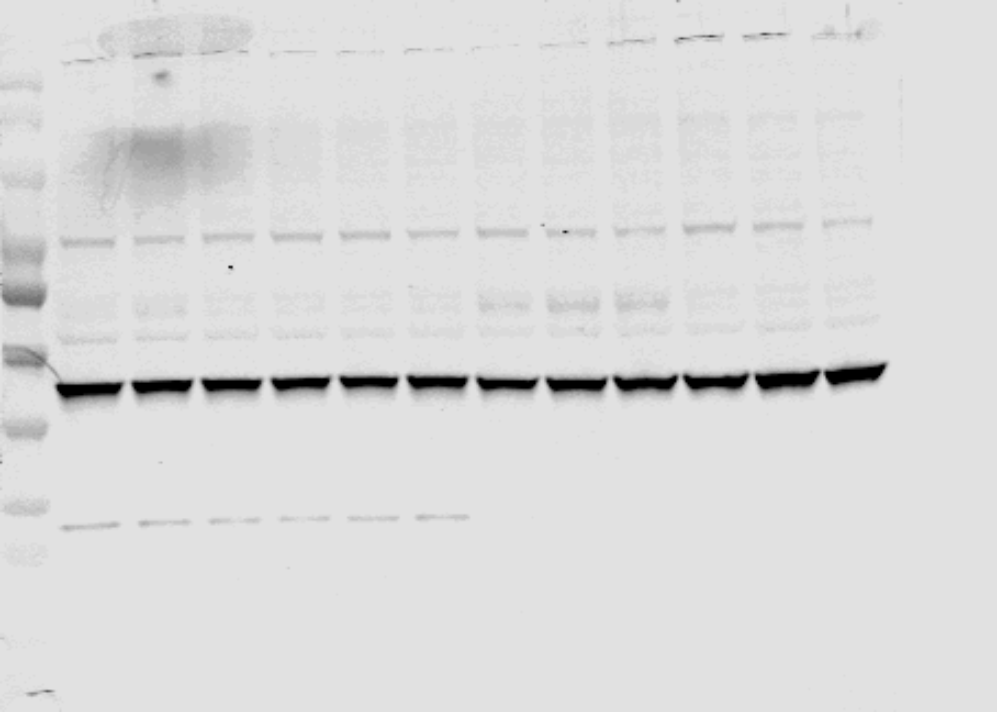

Supplement: Figure 3—figure supplement 2—source data 1. [file elife-102852-fig3-figsupp2-data1.zip › Figure 3-source data 3/Fig3Supplement2E_ATF4_original.tif]

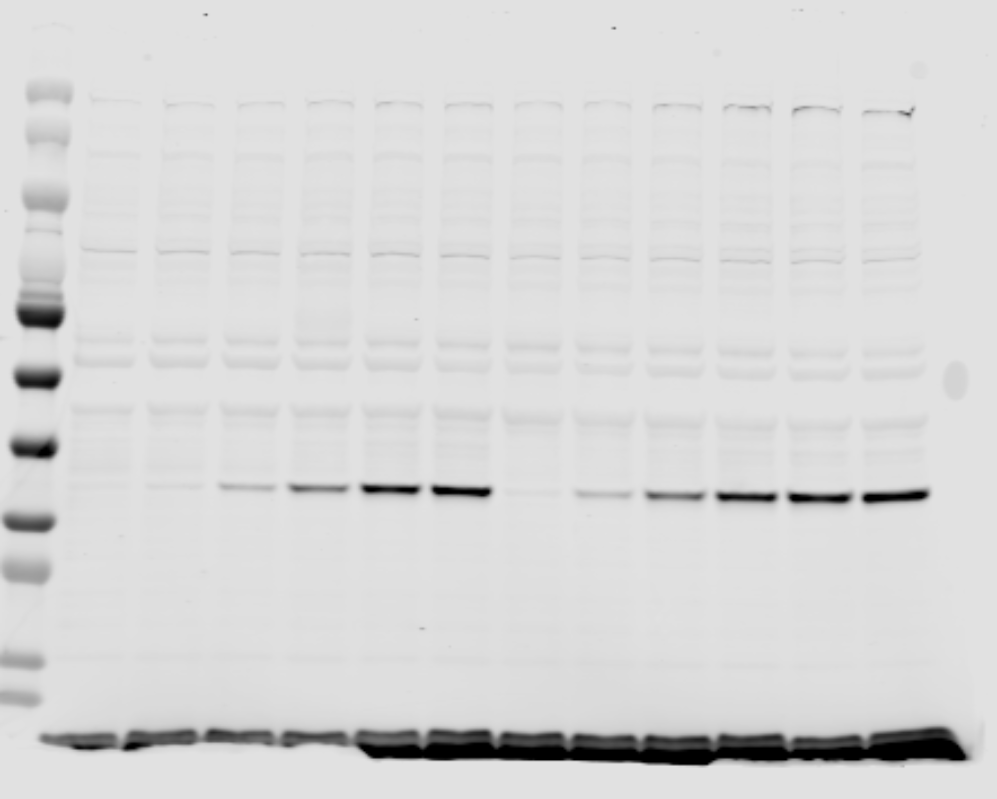

Supplement: Figure 3—figure supplement 2—source data 1. [file elife-102852-fig3-figsupp2-data1.zip › Figure 3-source data 3/Fig3Supplement2G_CHOP_original.tif]

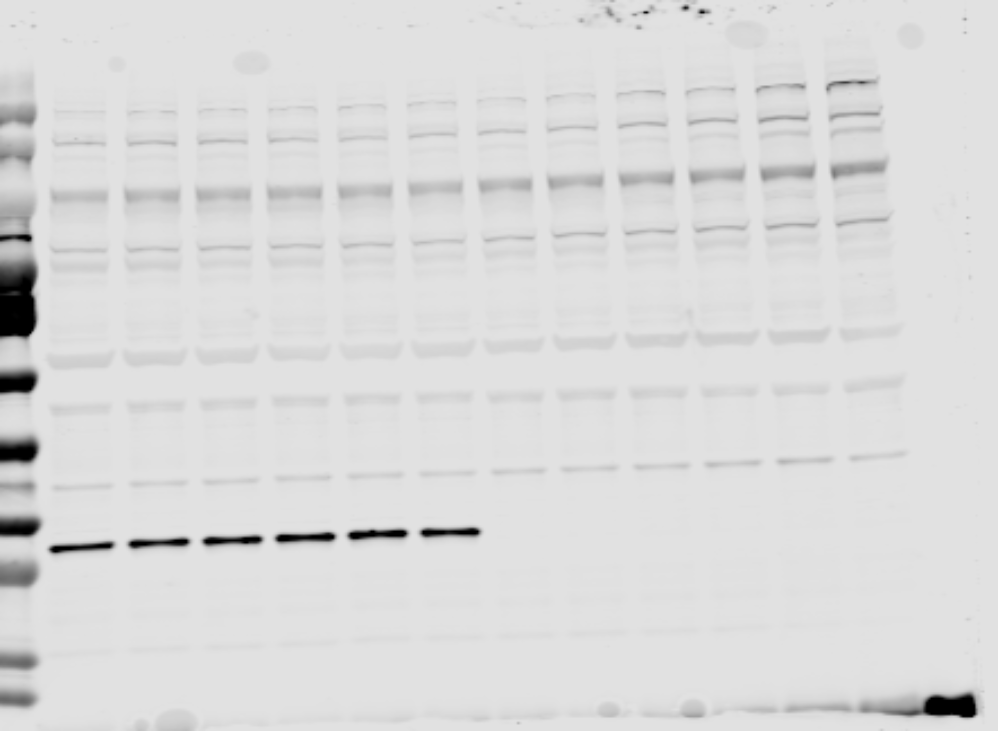

Supplement: Figure 3—figure supplement 2—source data 1. [file elife-102852-fig3-figsupp2-data1.zip › Figure 3-source data 3/Fig3Supplement2F_PEBP1_original.tif]

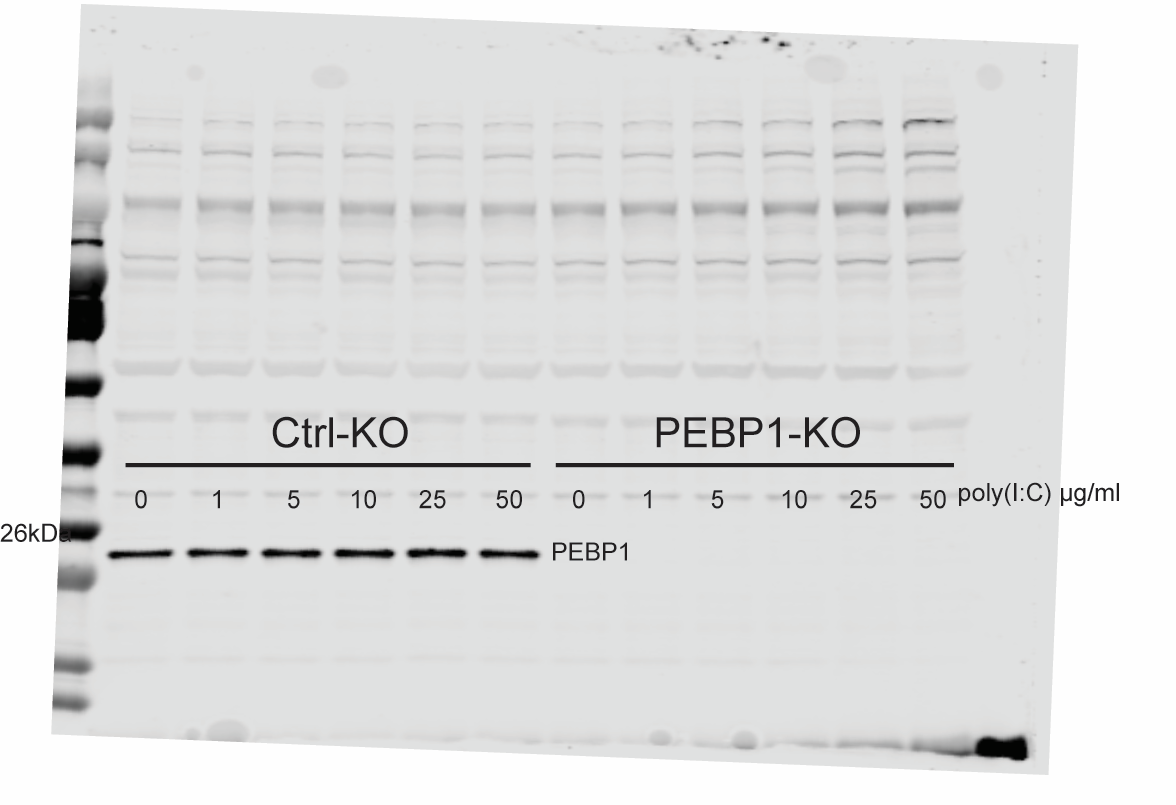

Supplement: Figure 3—figure supplement 2—source data 1. [file elife-102852-fig3-figsupp2-data1.zip › Figure 3-source data 3/Fig3Supplement2F_PEBP1_band_indicated.tif]

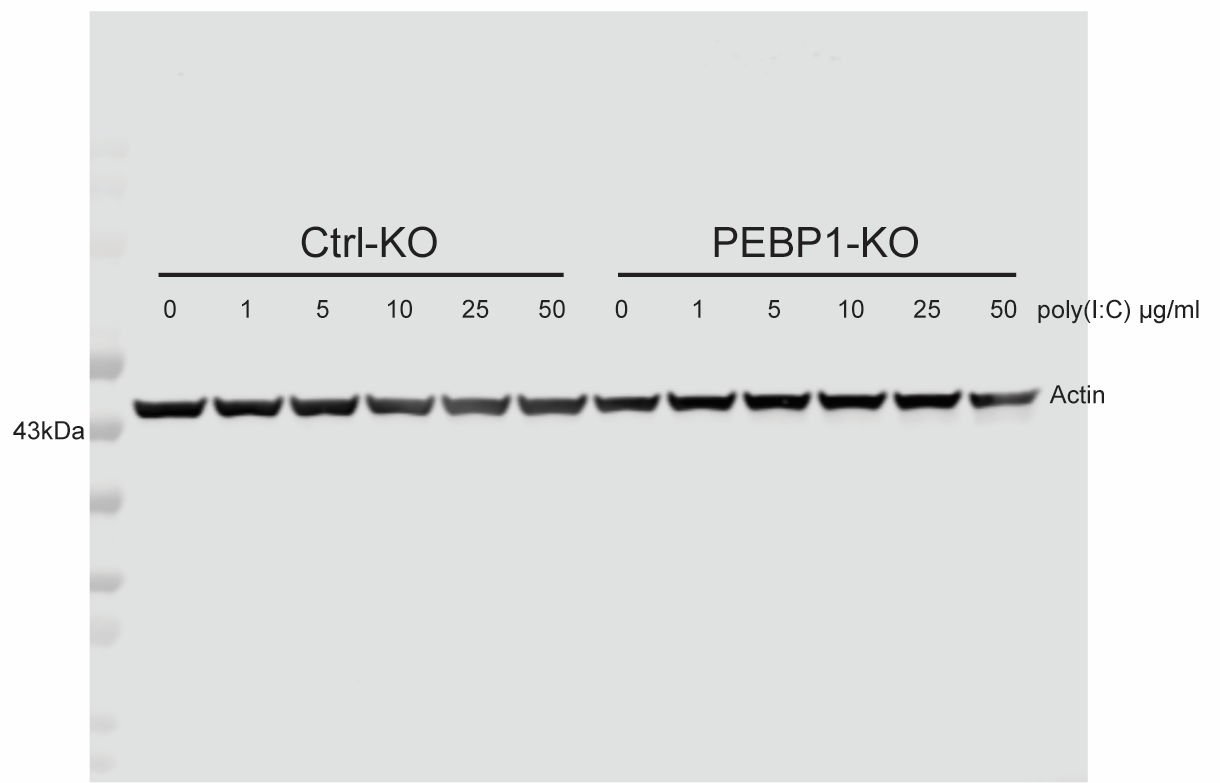

Supplement: Figure 3—figure supplement 2—source data 1. [file elife-102852-fig3-figsupp2-data1.zip › Figure 3-source data 3/Fig3Supplement2F_Actin_band_indicated.tif]

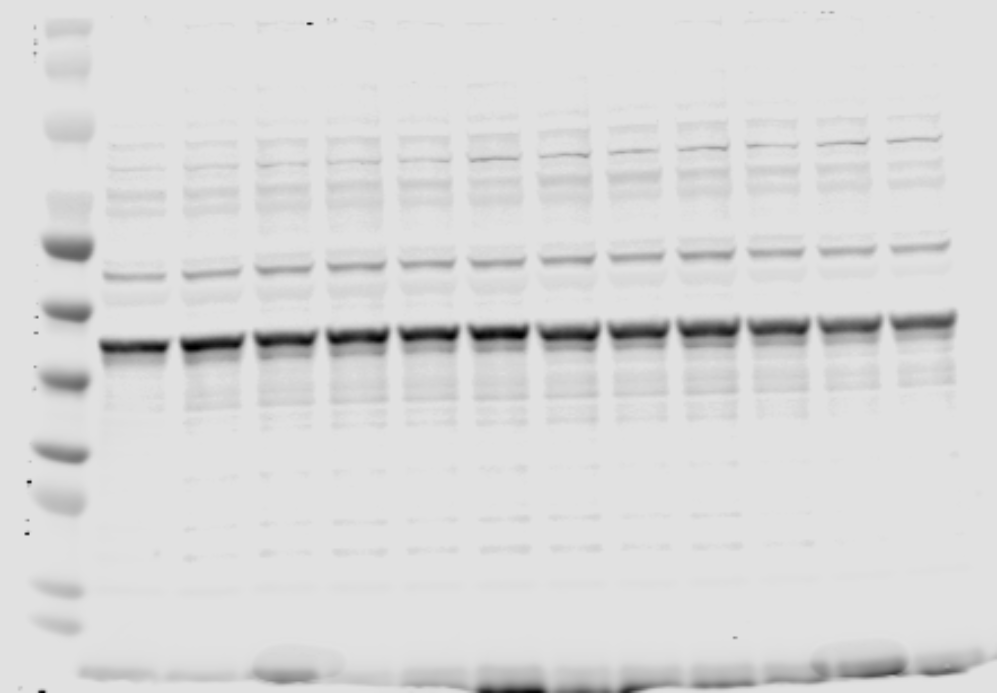

Supplement: Figure 3—figure supplement 2—source data 1. [file elife-102852-fig3-figsupp2-data1.zip › Figure 3-source data 3/Fig3Supplement2C_eIF2a_original.tif]

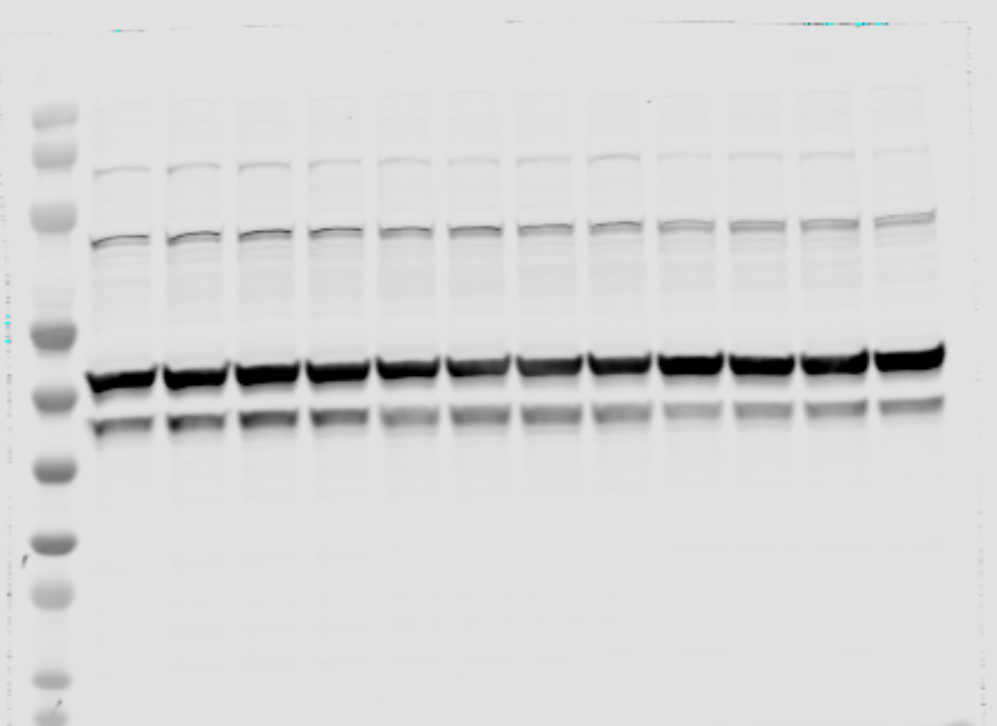

Supplement: Figure 3—figure supplement 2—source data 1. [file elife-102852-fig3-figsupp2-data1.zip › Figure 3-source data 3/Fig3Supplement2H_P-eIF2a_original.tif]

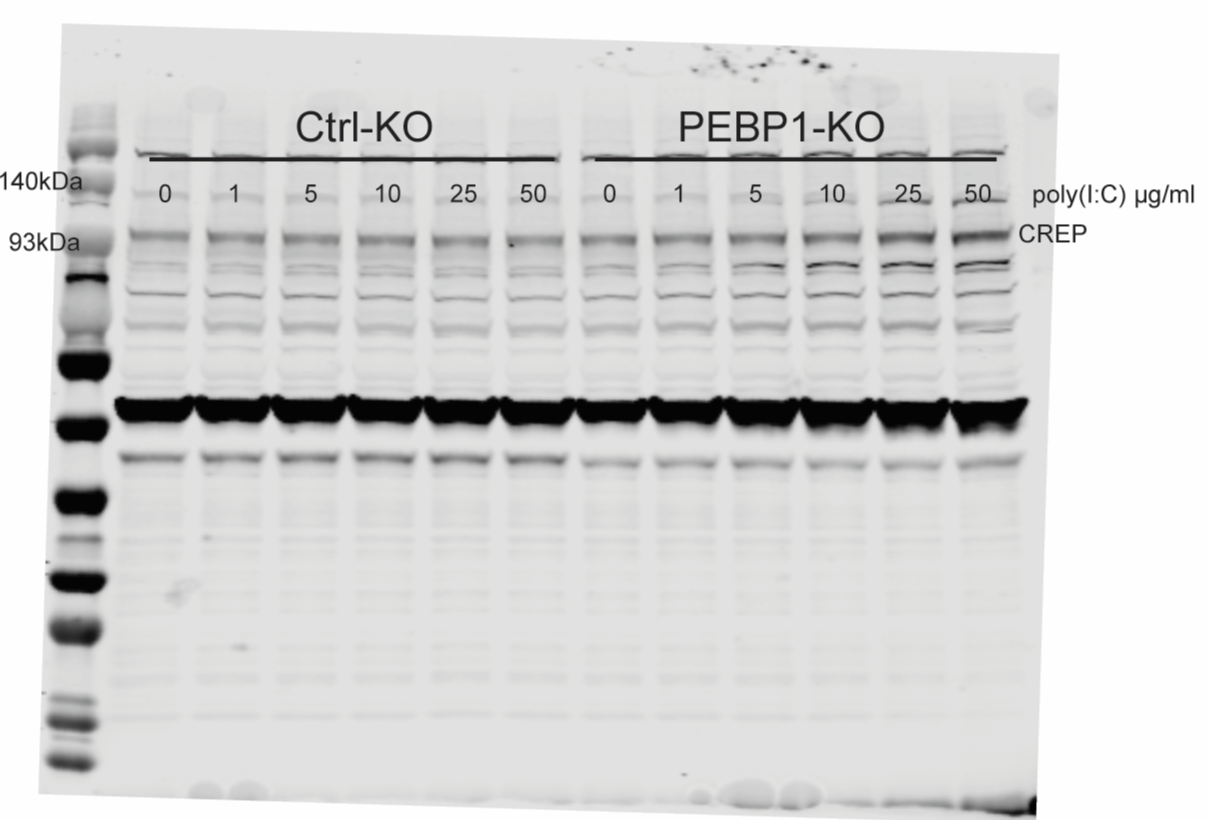

Supplement: Figure 3—figure supplement 2—source data 1. [file elife-102852-fig3-figsupp2-data1.zip › Figure 3-source data 3/Fig3Supplement2F_CREP_band_indicated.tif]

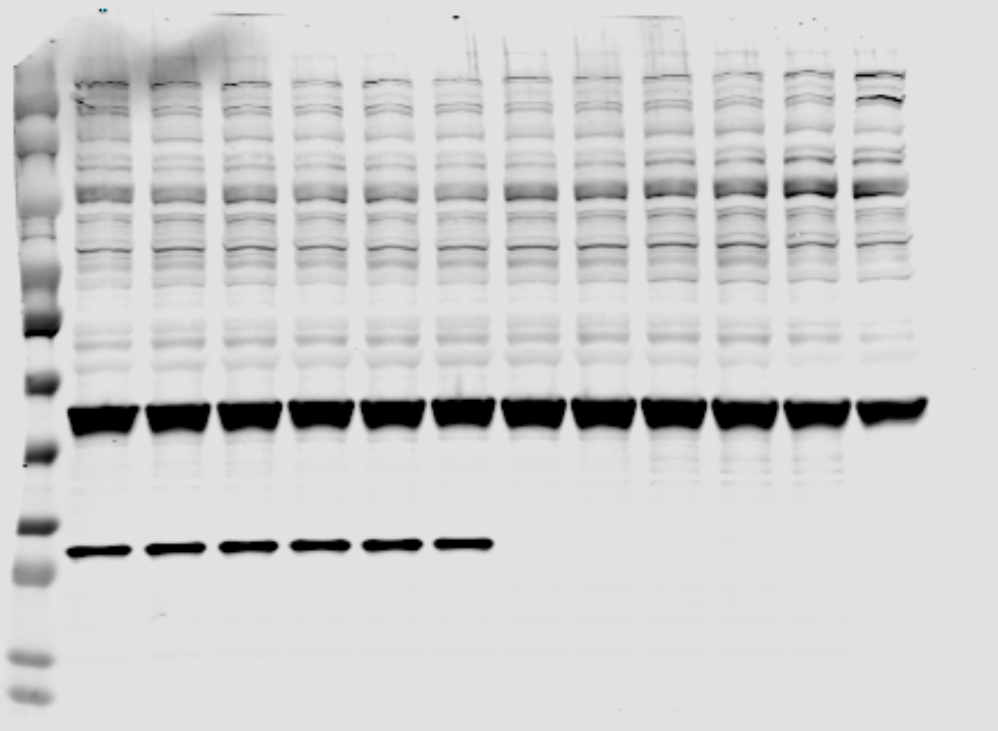

Supplement: Figure 3—figure supplement 2—source data 1. [file elife-102852-fig3-figsupp2-data1.zip › Figure 3-source data 3/Fig3Supplement2D_ATF4_original.tif]

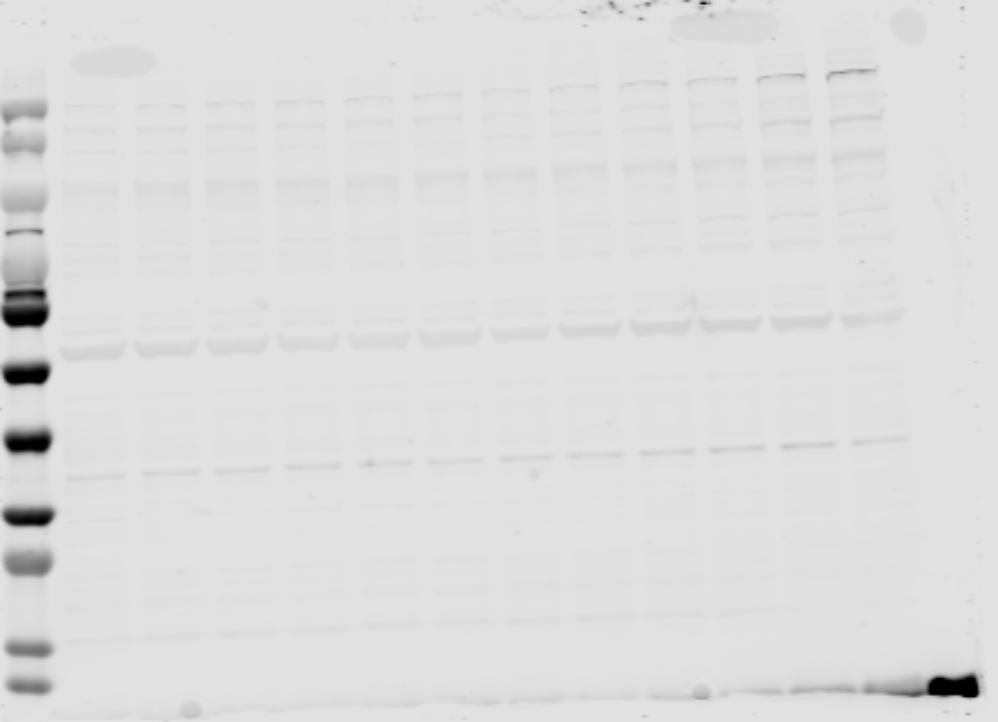

Supplement: Figure 3—figure supplement 2—source data 1. [file elife-102852-fig3-figsupp2-data1.zip › Figure 3-source data 3/Fig3Supplement2F_CHOP_original.tif]

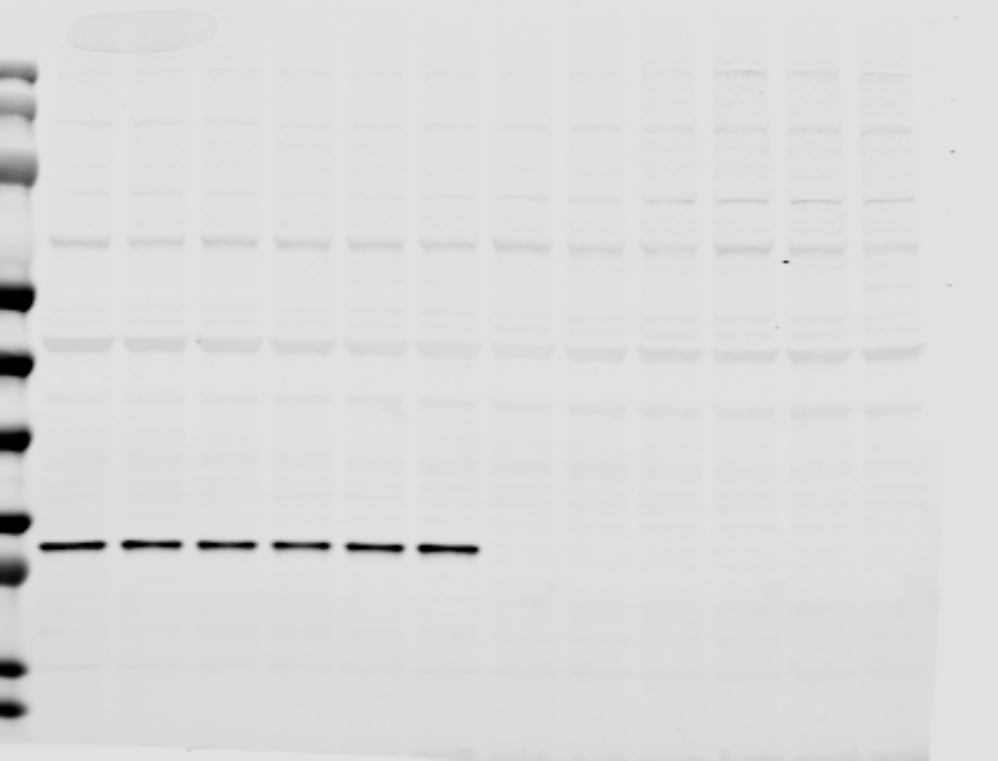

Supplement: Figure 3—figure supplement 2—source data 1. [file elife-102852-fig3-figsupp2-data1.zip › Figure 3-source data 3/Fig3Supplement2E_PEBP1_original.tif]

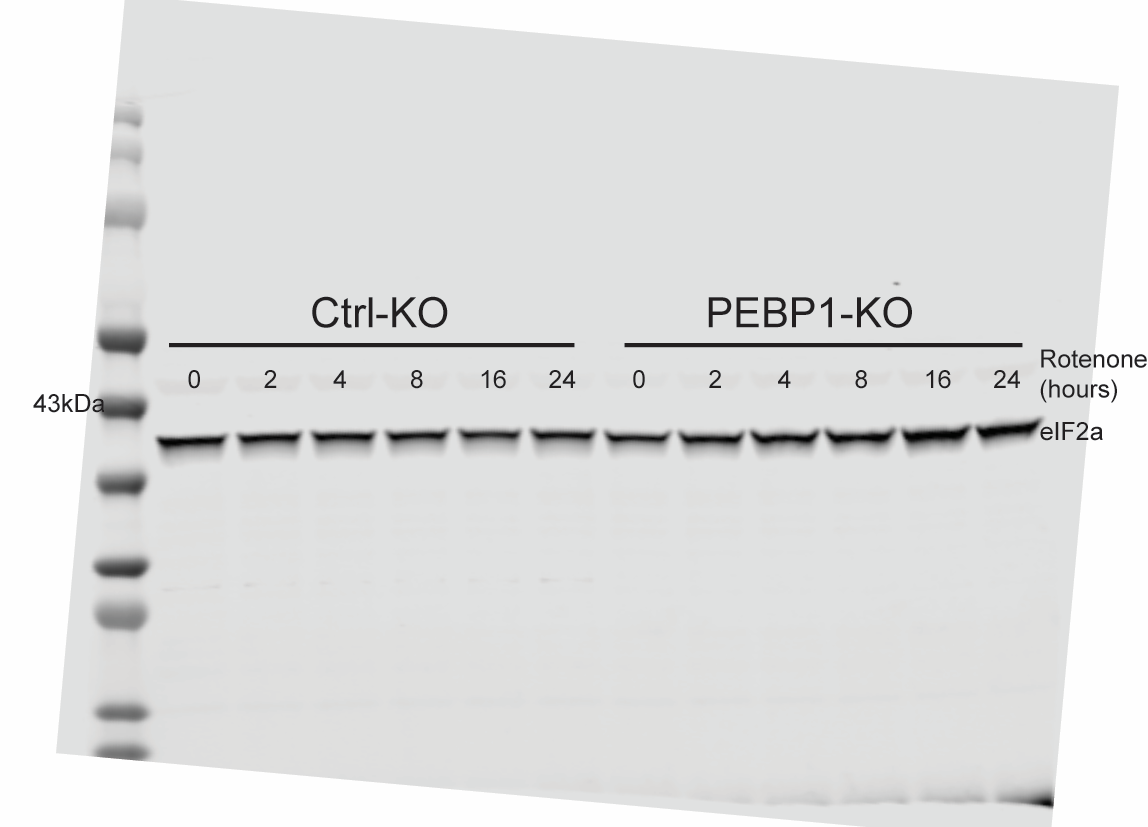

Supplement: Figure 3—figure supplement 2—source data 1. [file elife-102852-fig3-figsupp2-data1.zip › Figure 3-source data 3/Fig3Supplement2E_eIF2a_band_indicated.tif]

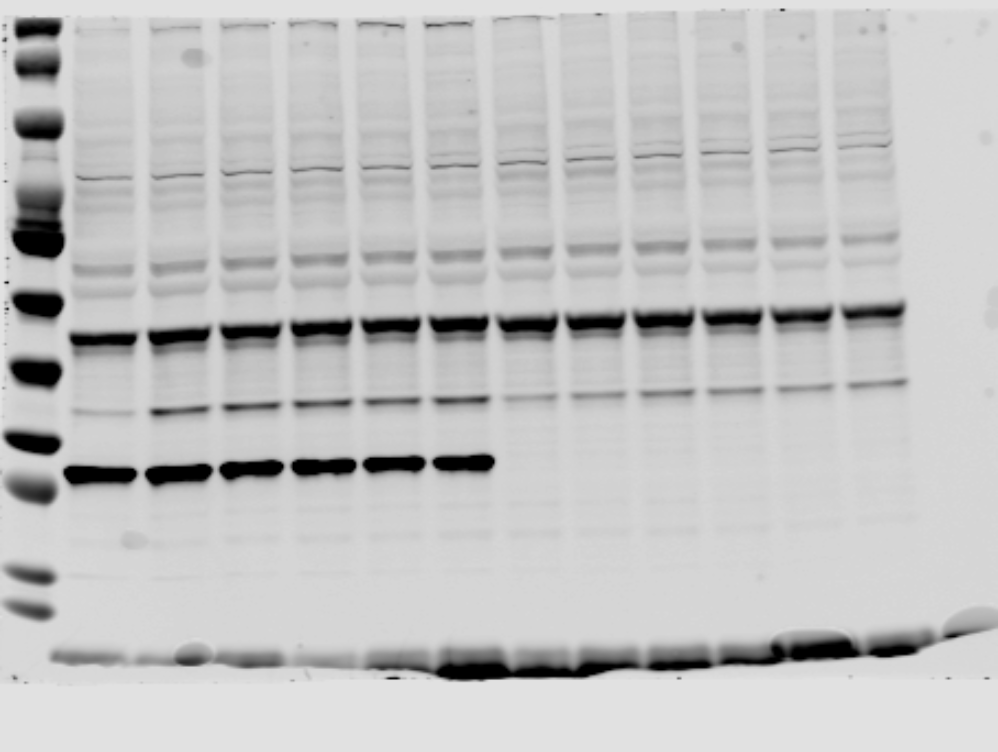

Supplement: Figure 3—figure supplement 2—source data 1. [file elife-102852-fig3-figsupp2-data1.zip › Figure 3-source data 3/Fig3Supplement2C_CHOP_original.tif]

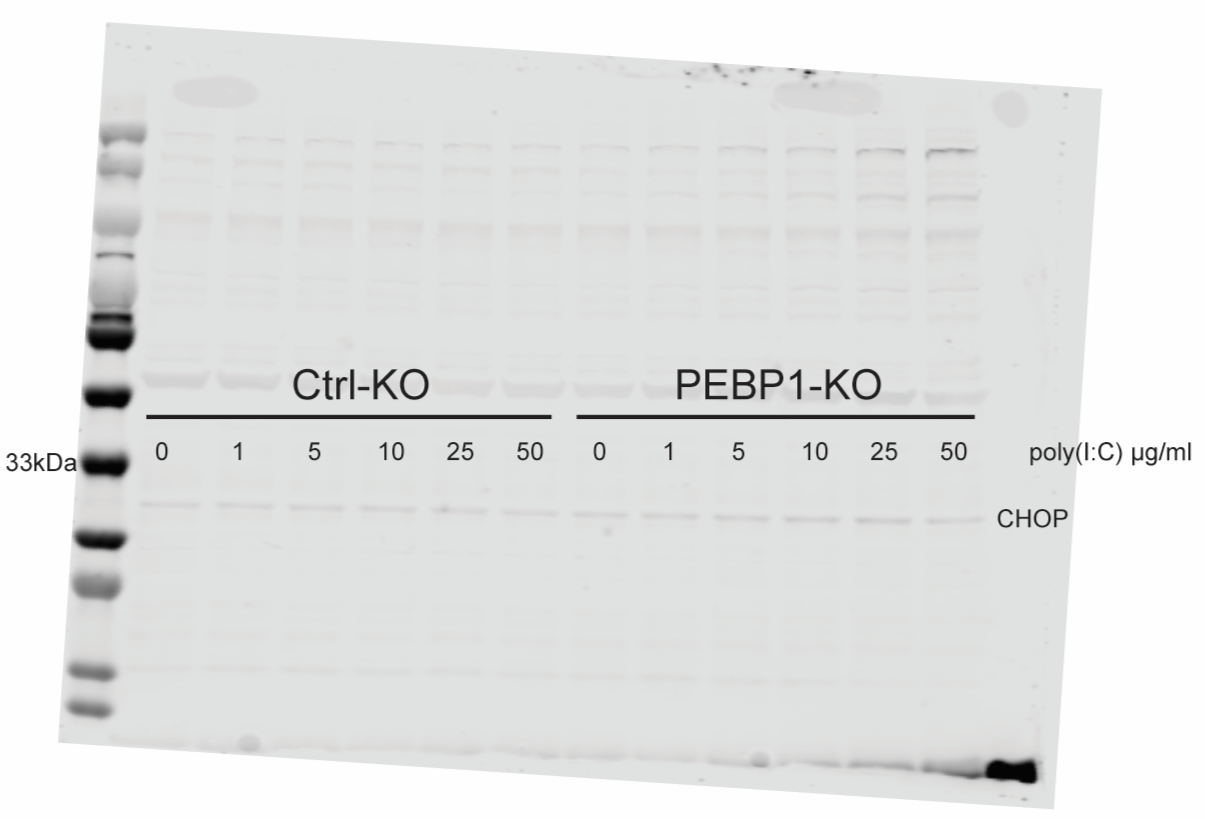

Supplement: Figure 3—figure supplement 2—source data 1. [file elife-102852-fig3-figsupp2-data1.zip › Figure 3-source data 3/Fig3Supplement2F_CHOP_band_indicated.tif]

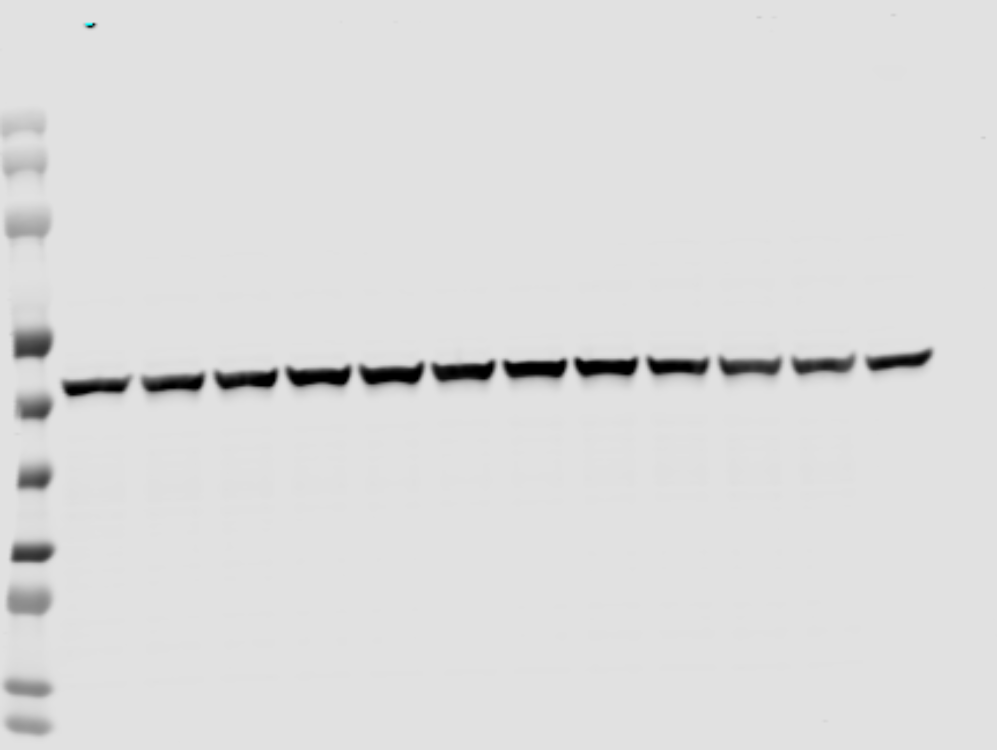

Supplement: Figure 3—figure supplement 2—source data 1. [file elife-102852-fig3-figsupp2-data1.zip › Figure 3-source data 3/Fig3Supplement2D_Actin_original.tif]

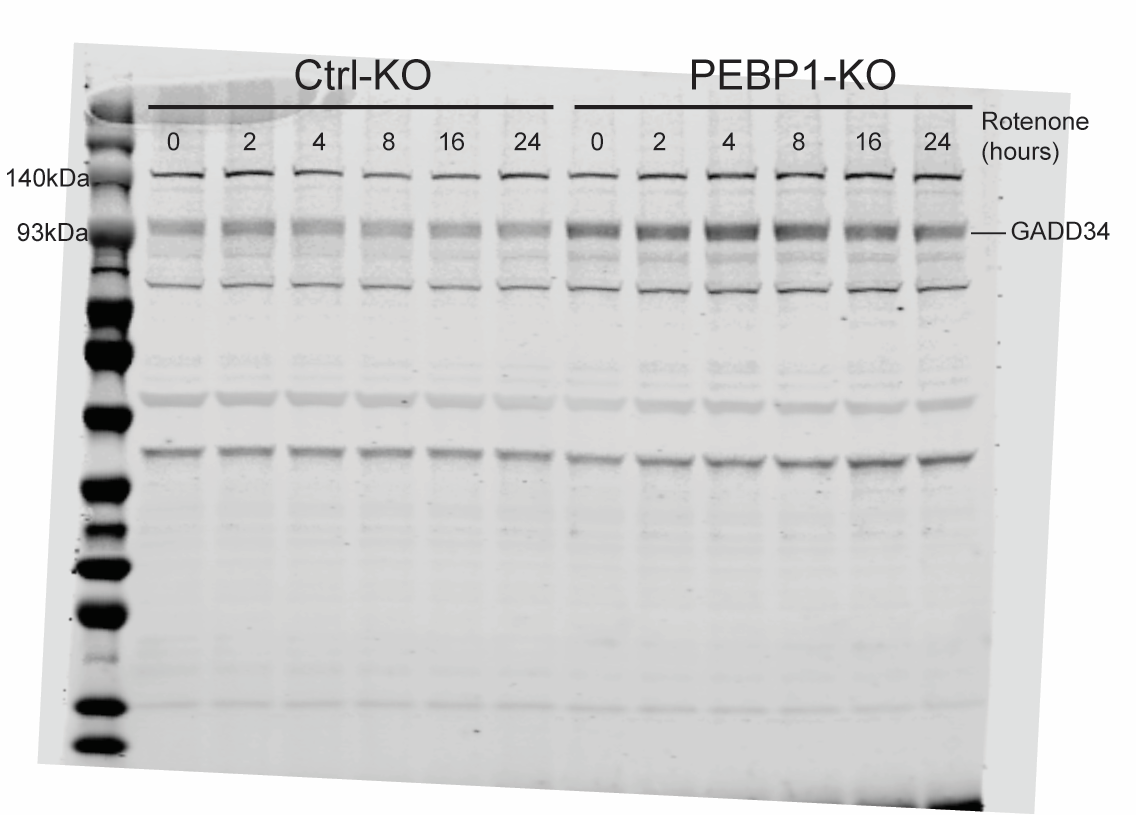

Supplement: Figure 3—figure supplement 2—source data 1. [file elife-102852-fig3-figsupp2-data1.zip › Figure 3-source data 3/Fig3Supplement2E_GADD34_band_indicated.tif]

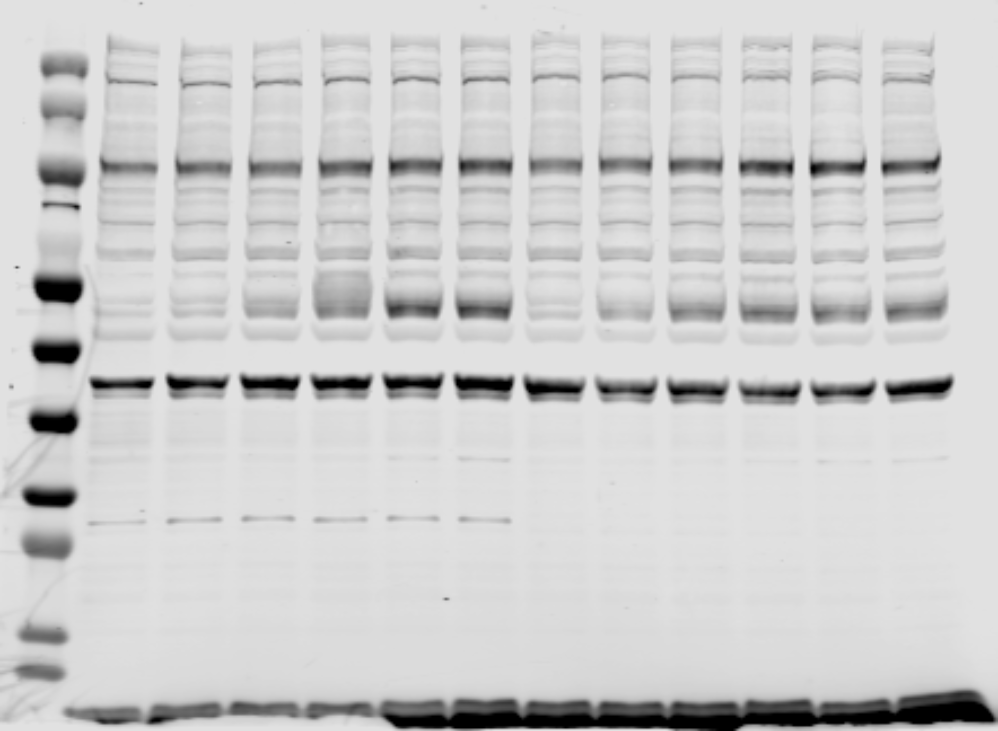

Supplement: Figure 3—figure supplement 2—source data 1. [file elife-102852-fig3-figsupp2-data1.zip › Figure 3-source data 3/Fig3Supplement2G_ATF4_original.tif]

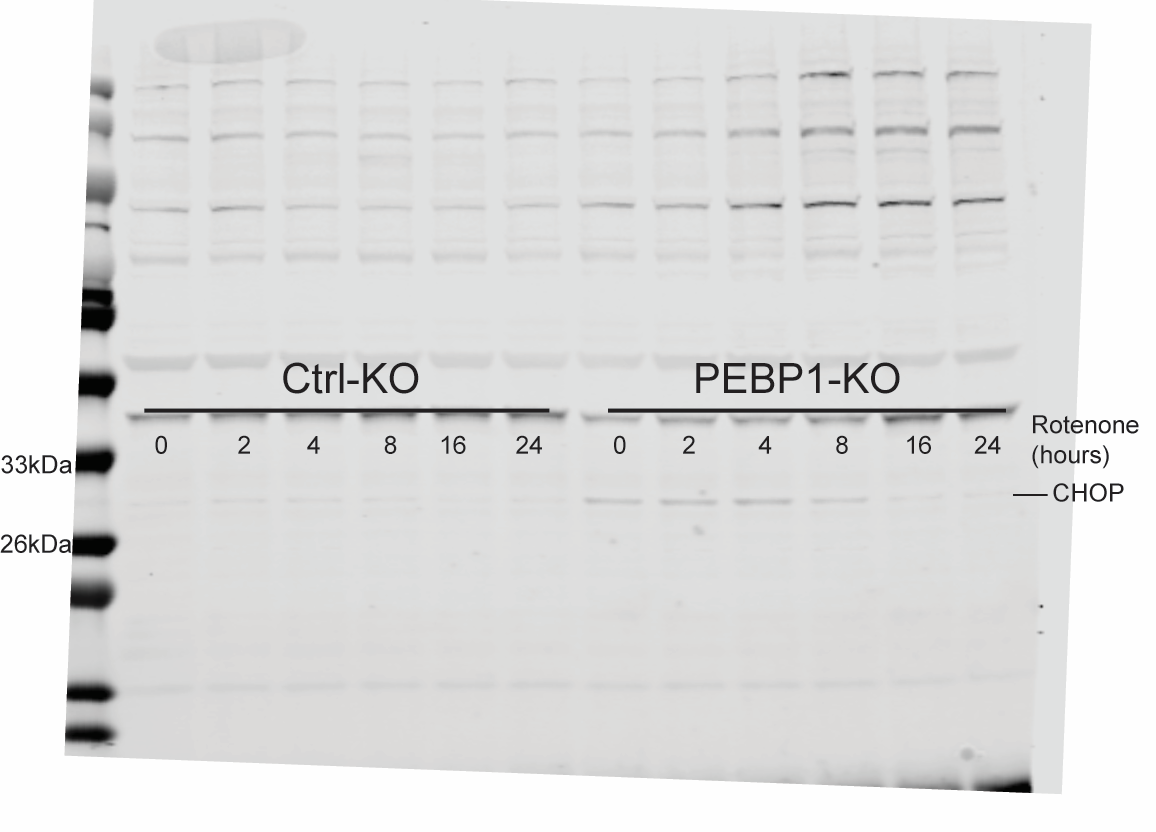

Supplement: Figure 3—figure supplement 2—source data 1. [file elife-102852-fig3-figsupp2-data1.zip › Figure 3-source data 3/Fig3Supplement2E_CHOP_band_indicated.tif]

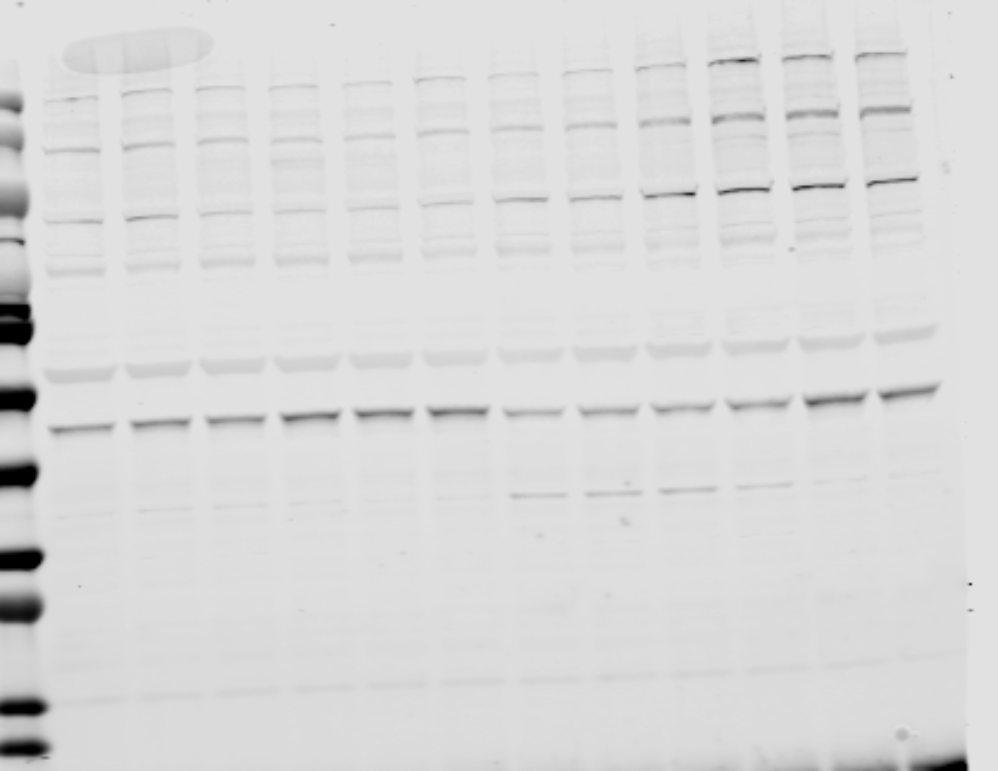

Supplement: Figure 3—figure supplement 2—source data 1. [file elife-102852-fig3-figsupp2-data1.zip › Figure 3-source data 3/Fig3Supplement2E_CHOP_original.tif]

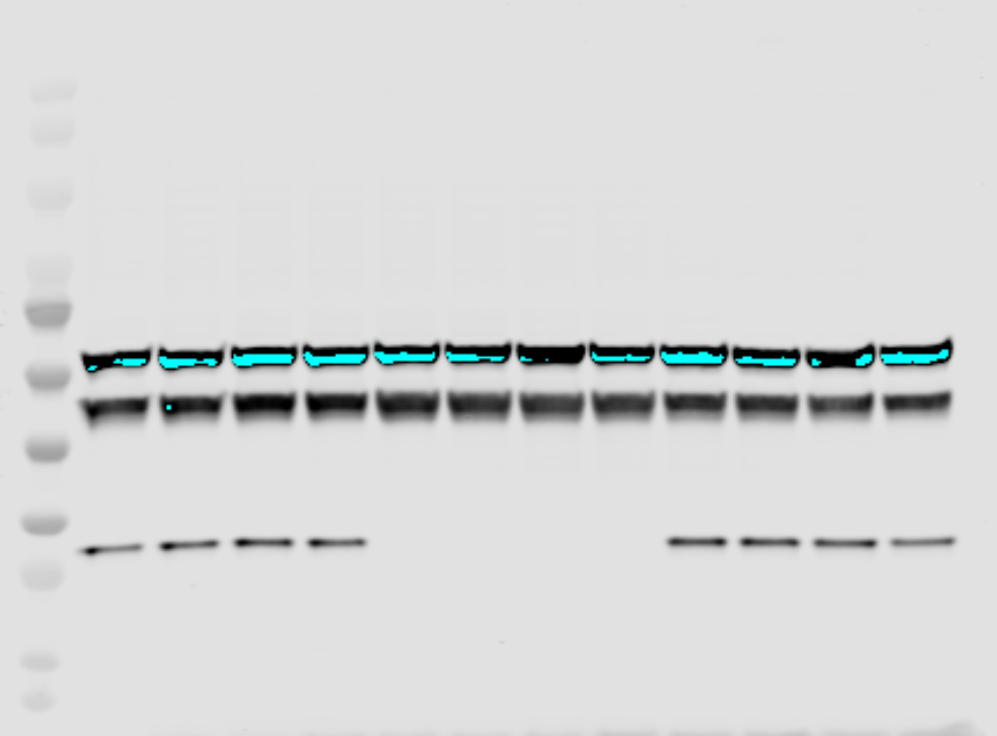

Supplement: Figure 3—figure supplement 2—source data 1. [file elife-102852-fig3-figsupp2-data1.zip › Figure 3-source data 3/Fig3Supplement2H_eIF2a_PEBP1_original.tif]

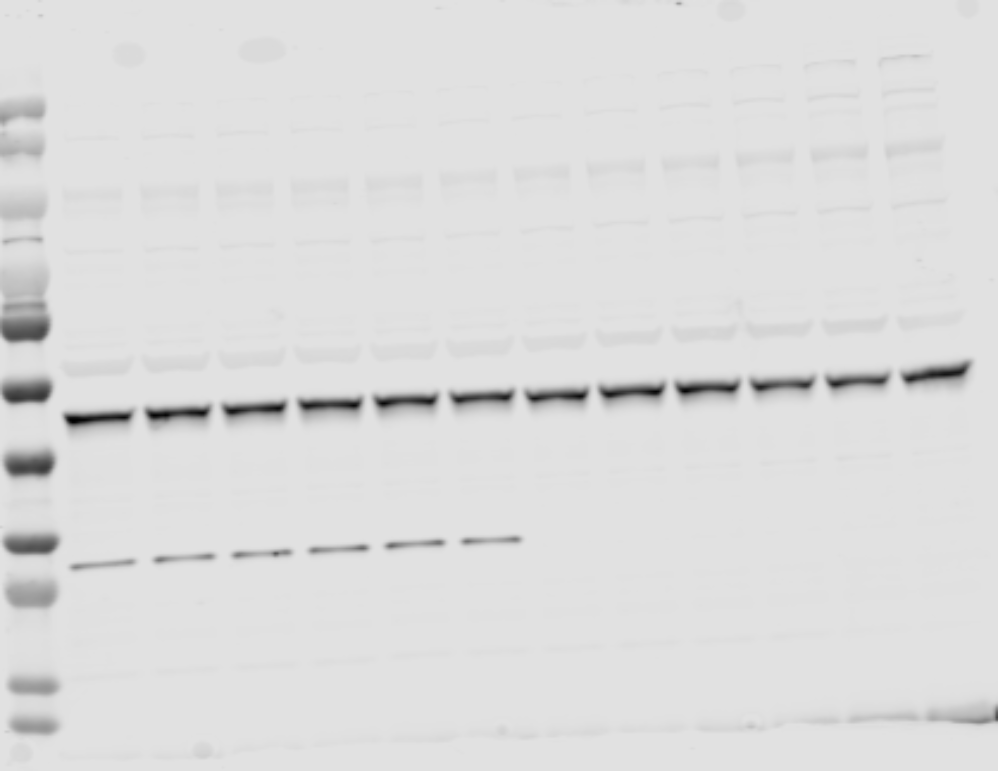

Supplement: Figure 3—figure supplement 2—source data 1. [file elife-102852-fig3-figsupp2-data1.zip › Figure 3-source data 3/Fig3Supplement2F_eIF2a_original.tif]

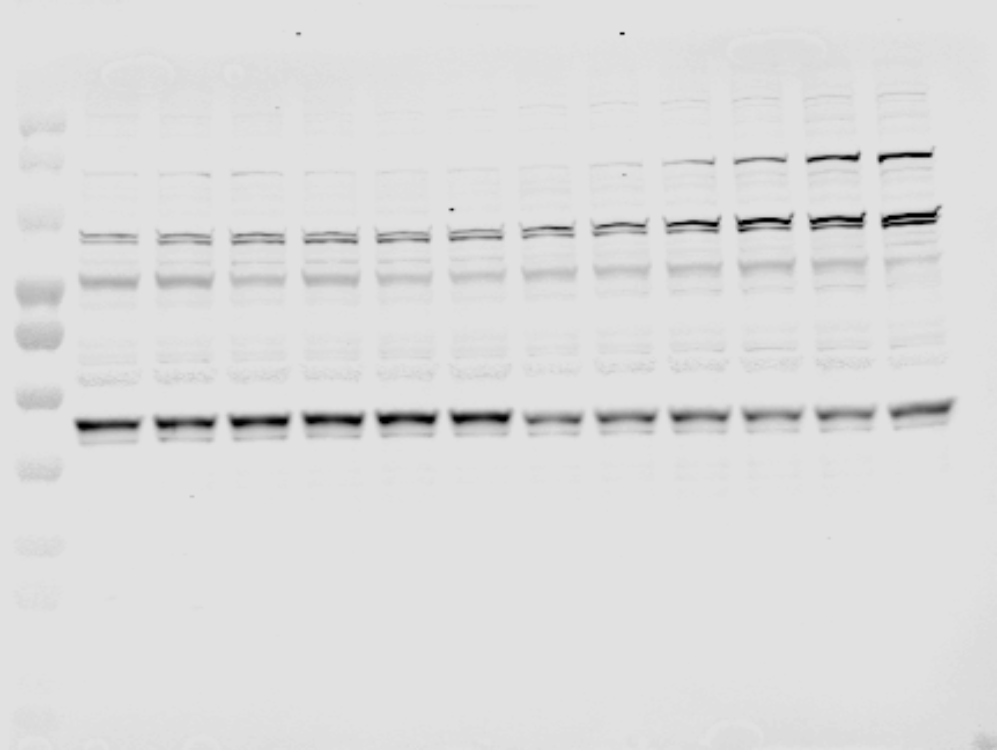

Supplement: Figure 3—figure supplement 2—source data 1. [file elife-102852-fig3-figsupp2-data1.zip › Figure 3-source data 3/Fig3Supplement2F_P-eIF2a_original.tif]

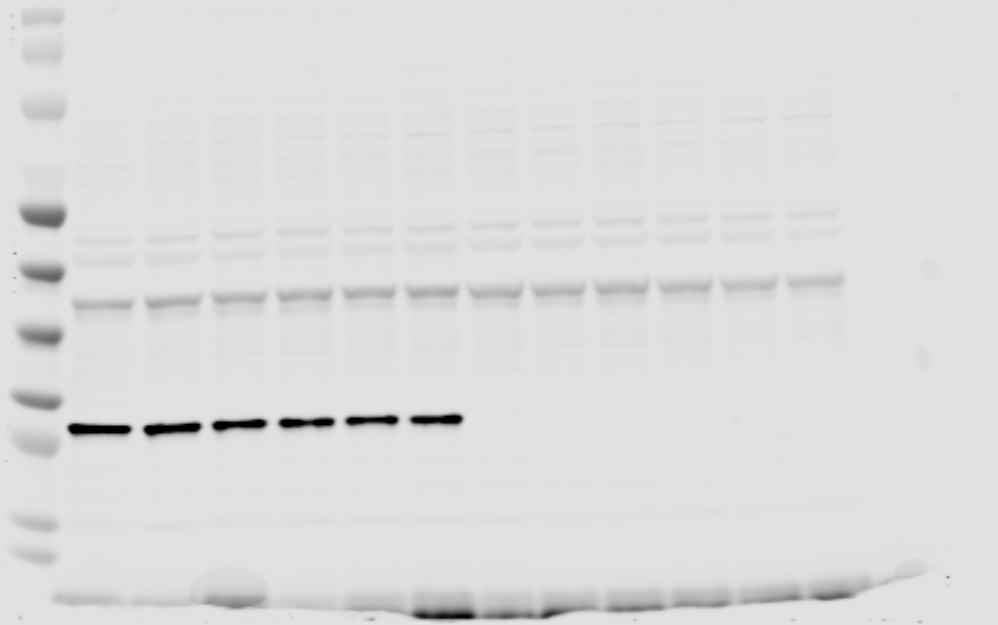

Supplement: Figure 3—figure supplement 2—source data 1. [file elife-102852-fig3-figsupp2-data1.zip › Figure 3-source data 3/Fig3Supplement2C_PEBP1_original.tif]

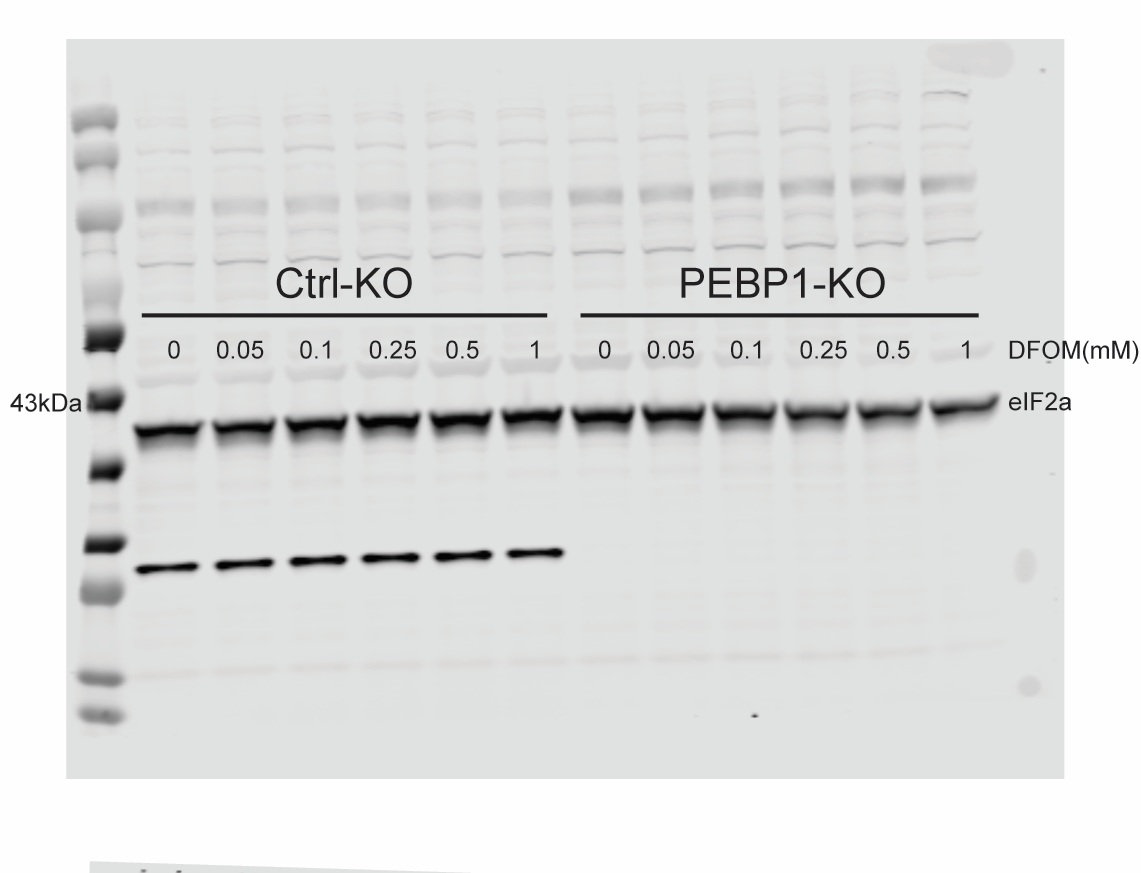

Supplement: Figure 3—figure supplement 2—source data 1. [file elife-102852-fig3-figsupp2-data1.zip › Figure 3-source data 3/Fig3Supplement2D_eIF2a_band_indicated.tif]

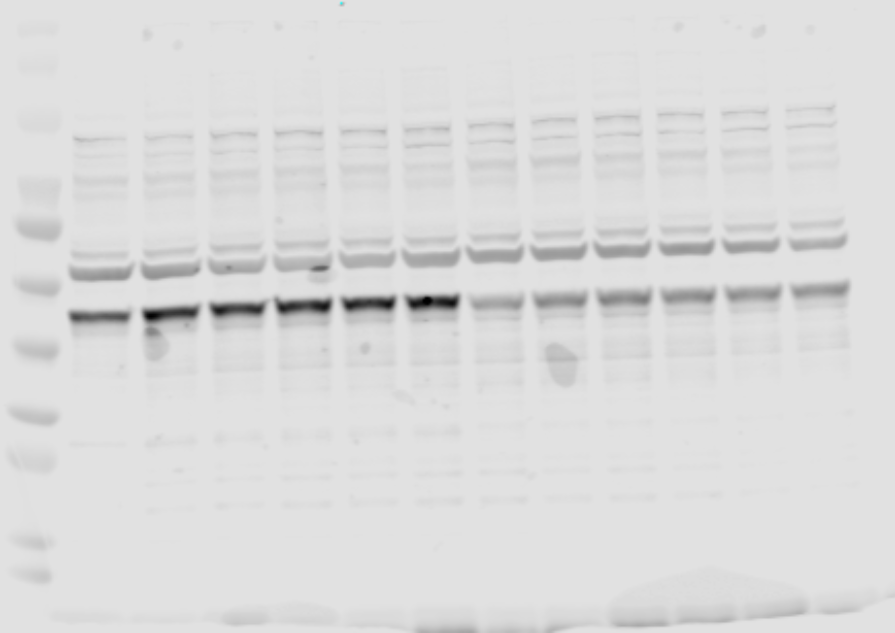

Supplement: Figure 3—figure supplement 2—source data 1. [file elife-102852-fig3-figsupp2-data1.zip › Figure 3-source data 3/Fig3Supplement2C_P-eIF2a_original.tif]

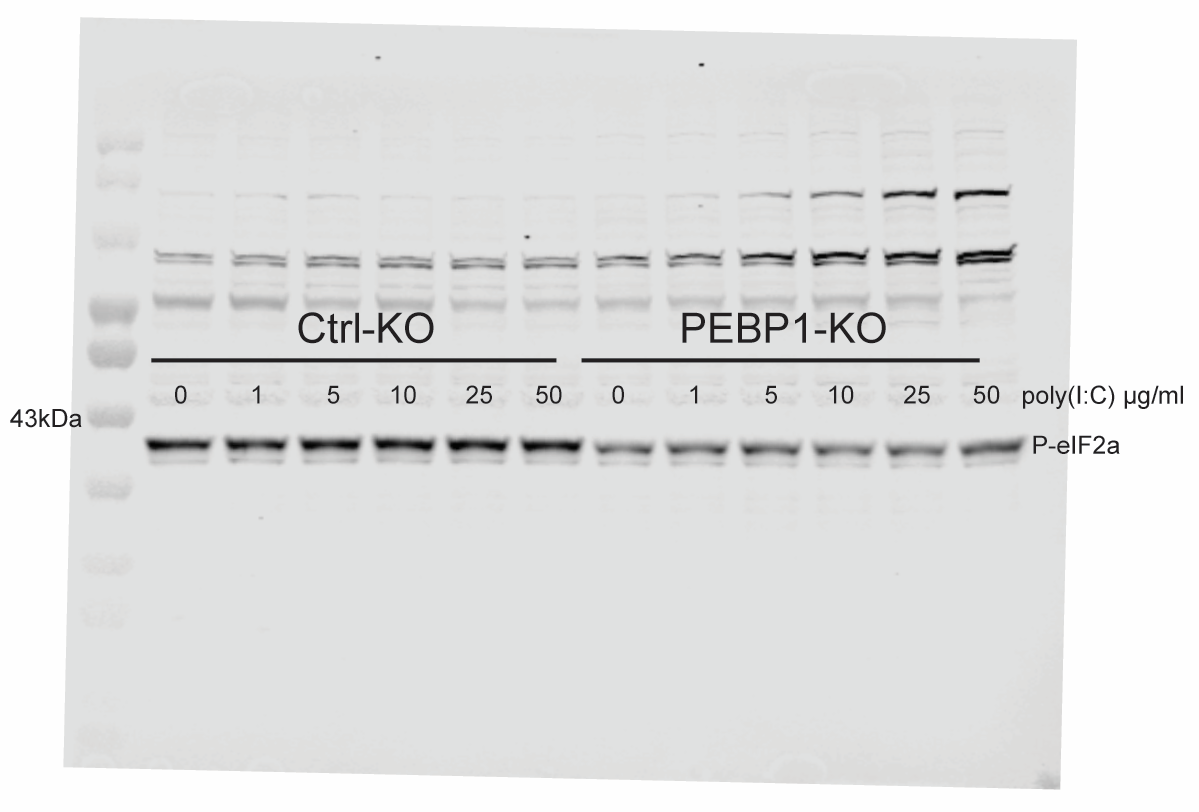

Supplement: Figure 3—figure supplement 2—source data 1. [file elife-102852-fig3-figsupp2-data1.zip › Figure 3-source data 3/Fig3Supplement2F_P-eIF2a_band_indicated.tif]

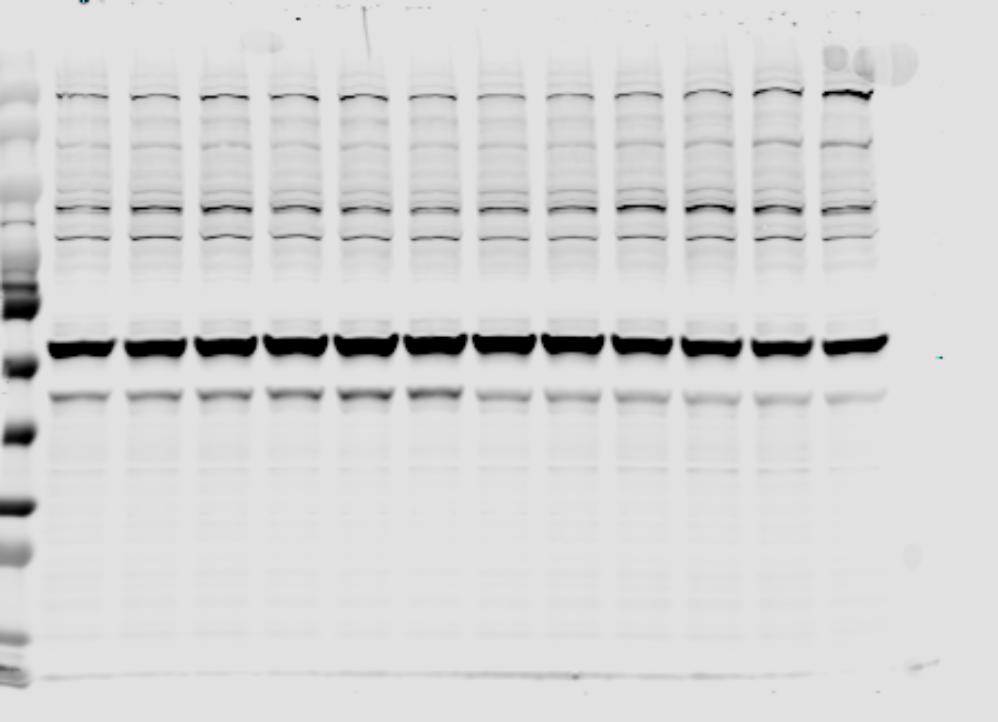

Supplement: Figure 3—figure supplement 2—source data 1. [file elife-102852-fig3-figsupp2-data1.zip › Figure 3-source data 3/Fig3Supplement2D_CHOP_original.tif]

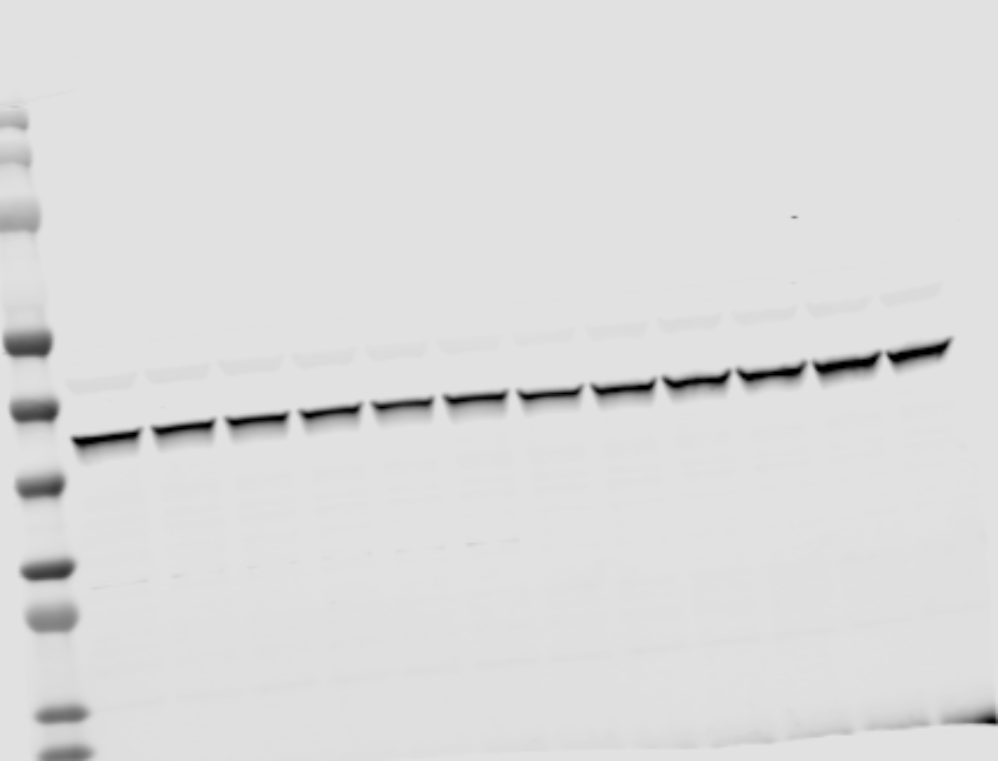

Supplement: Figure 3—figure supplement 2—source data 1. [file elife-102852-fig3-figsupp2-data1.zip › Figure 3-source data 3/Fig3Supplement2E_eIF2a_original.tif]

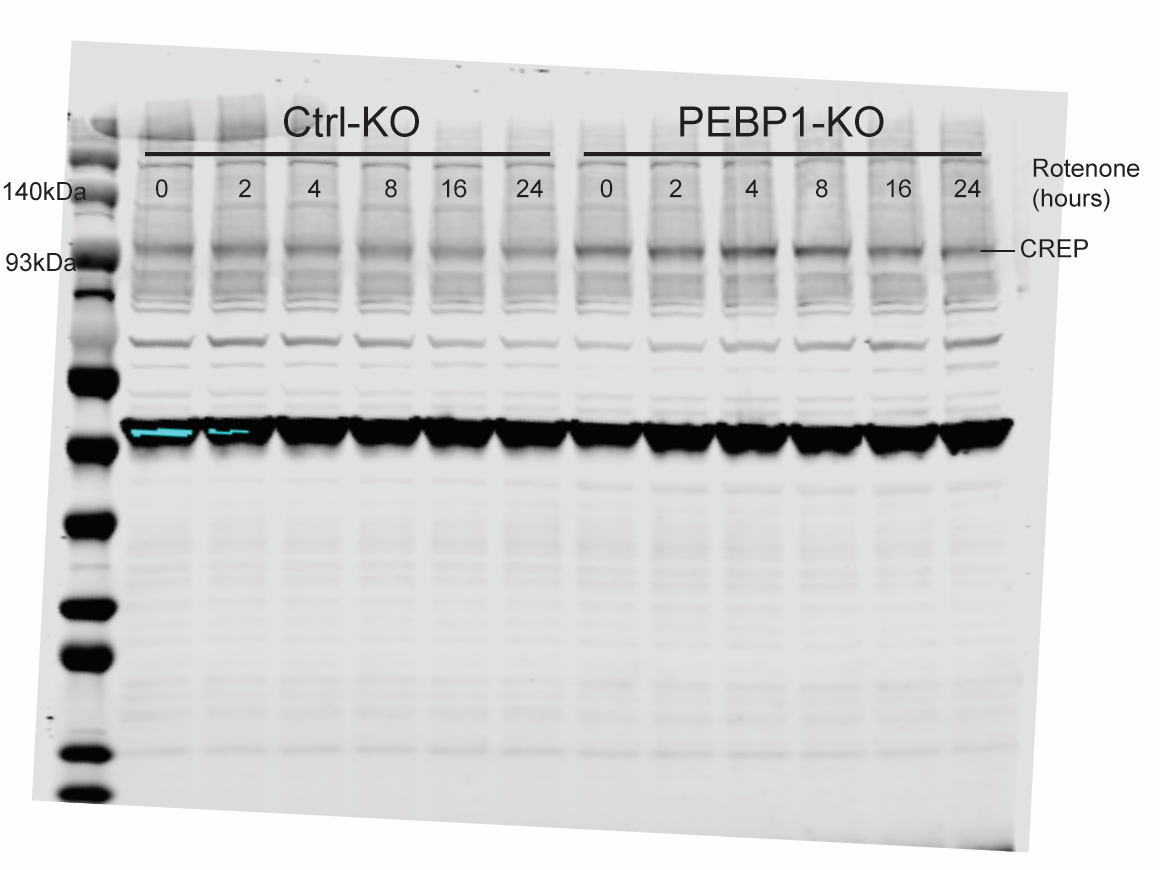

Supplement: Figure 3—figure supplement 2—source data 1. [file elife-102852-fig3-figsupp2-data1.zip › Figure 3-source data 3/Fig3Supplement2E_CREP_band_indicated.tif]

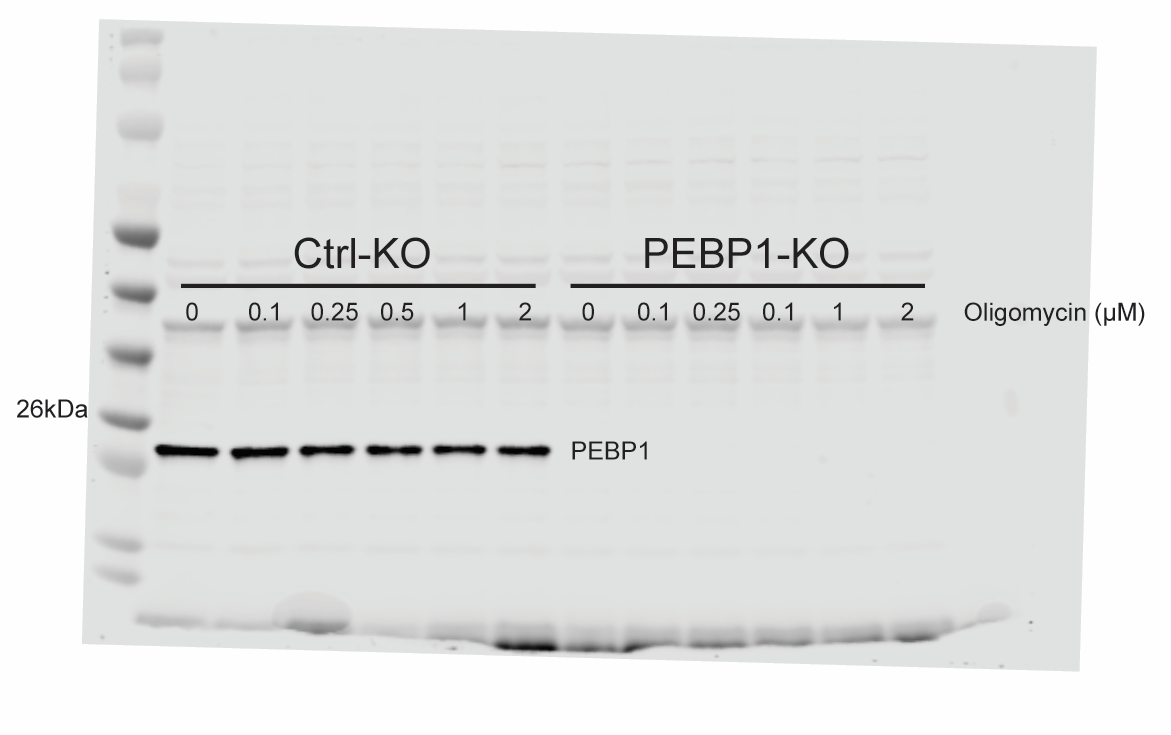

Supplement: Figure 3—figure supplement 2—source data 1. [file elife-102852-fig3-figsupp2-data1.zip › Figure 3-source data 3/Fig3Supplement2C_PEBP1_band_indicated.tif]

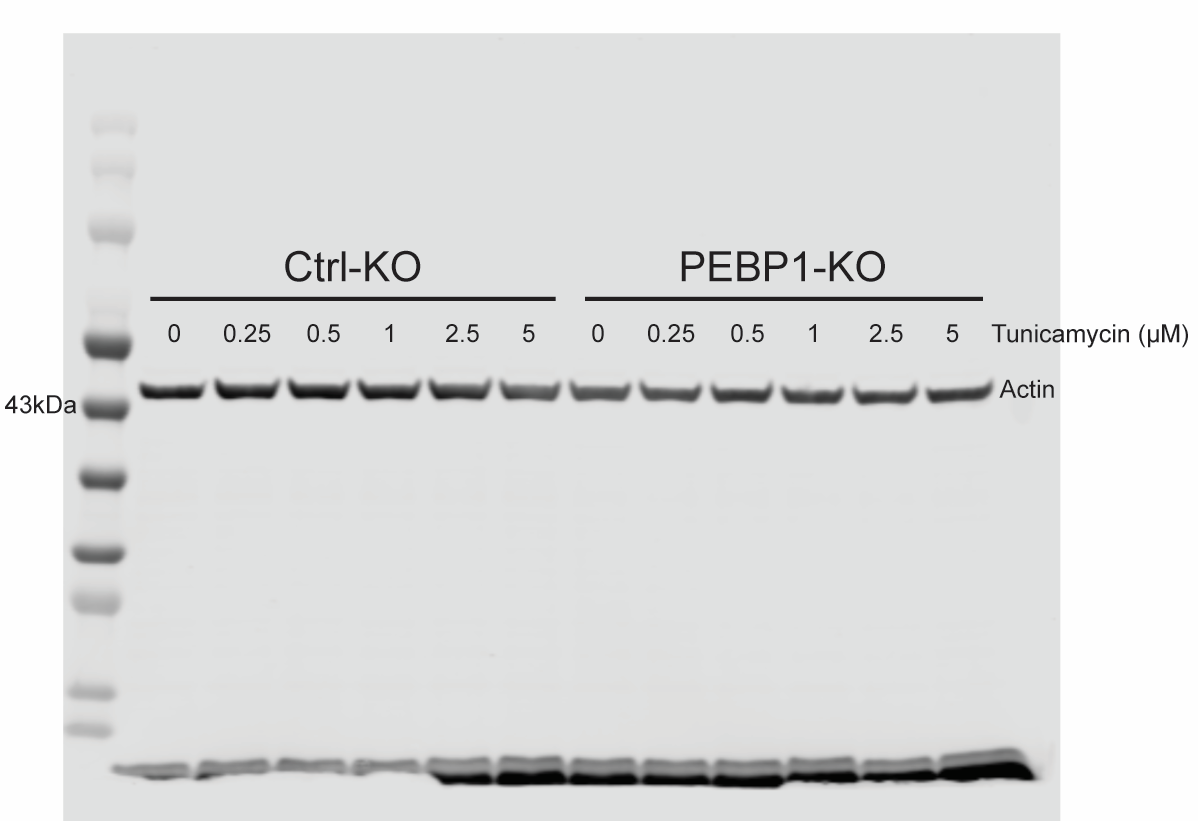

Supplement: Figure 3—figure supplement 2—source data 1. [file elife-102852-fig3-figsupp2-data1.zip › Figure 3-source data 3/Fig3Supplement2G_Actin_band_indicated.tif]

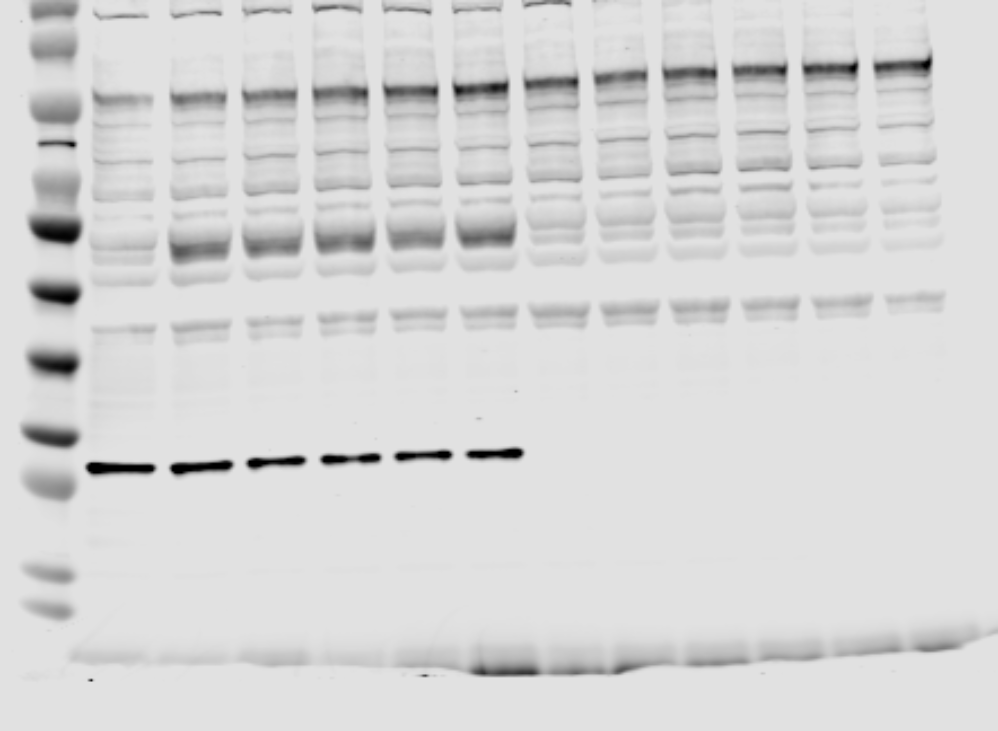

Supplement: Figure 3—figure supplement 2—source data 1. [file elife-102852-fig3-figsupp2-data1.zip › Figure 3-source data 3/Fig3Supplement2C_ATF4_original.tif]

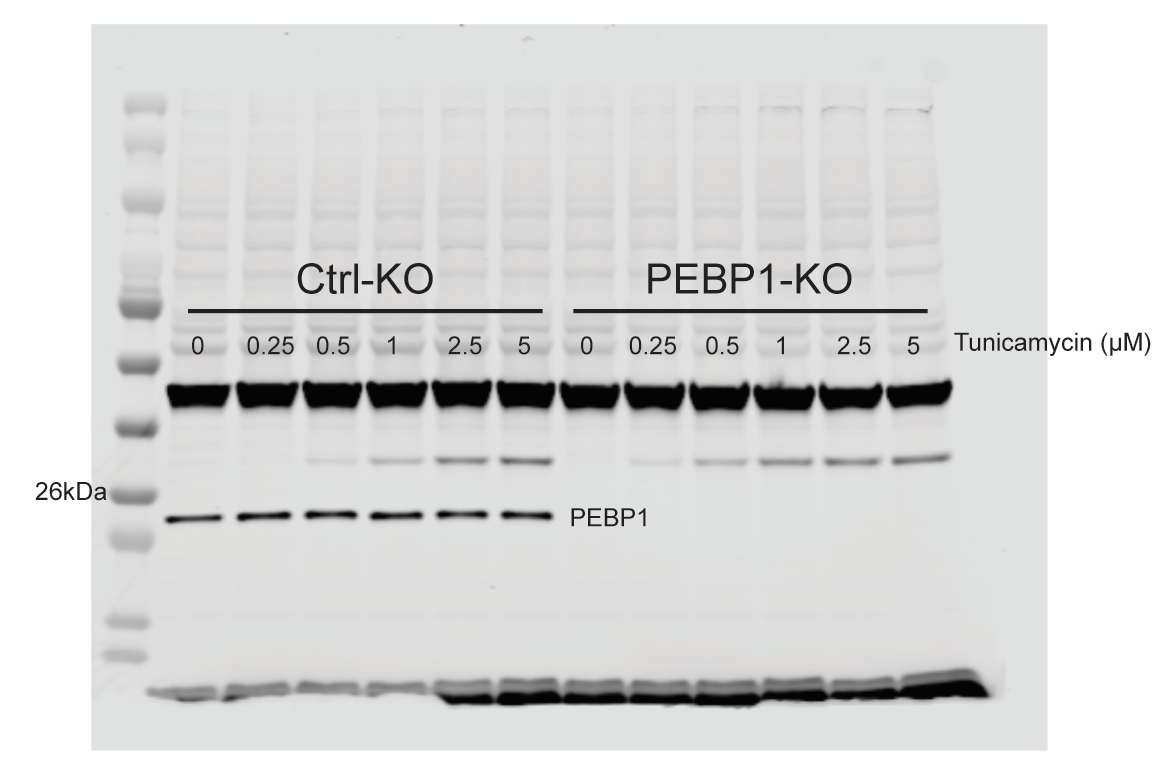

Supplement: Figure 3—figure supplement 2—source data 1. [file elife-102852-fig3-figsupp2-data1.zip › Figure 3-source data 3/Fig3Supplement2G_PEBP1_band_indicated.tif]

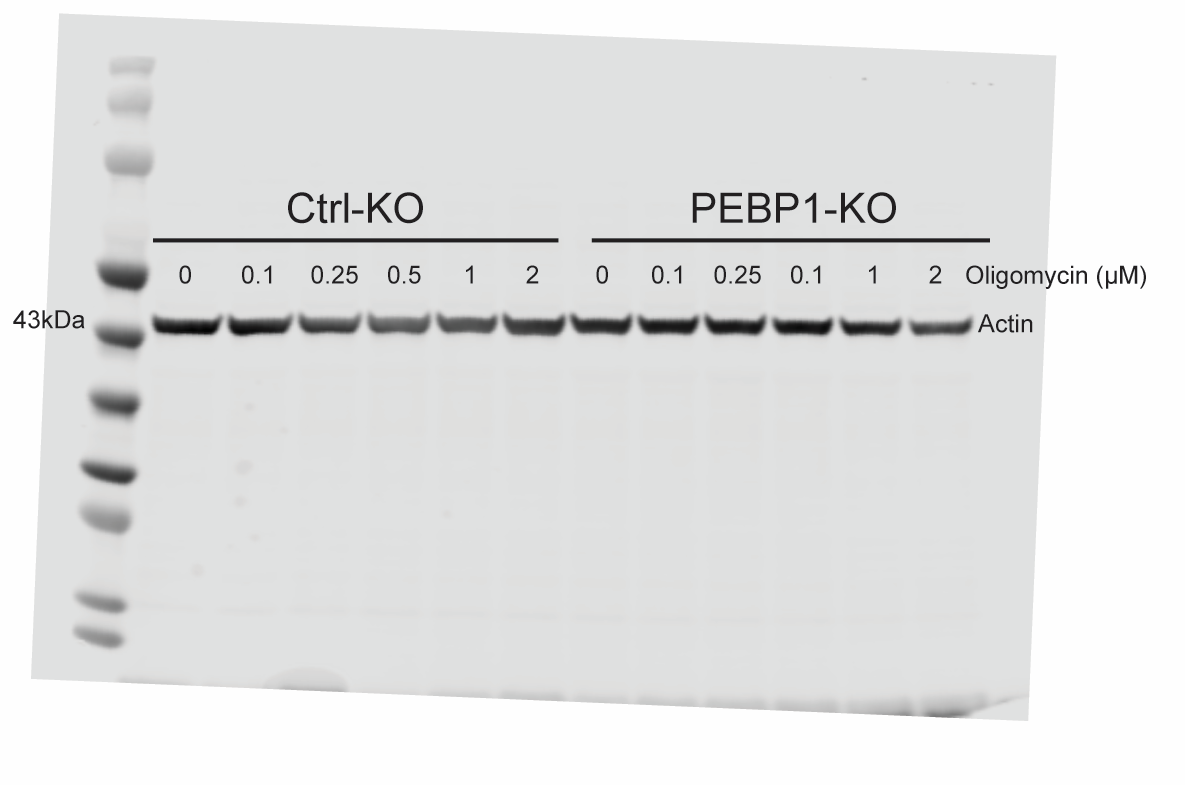

Supplement: Figure 3—figure supplement 2—source data 1. [file elife-102852-fig3-figsupp2-data1.zip › Figure 3-source data 3/Fig3Supplement2C_Actin_band_indicated.tif]

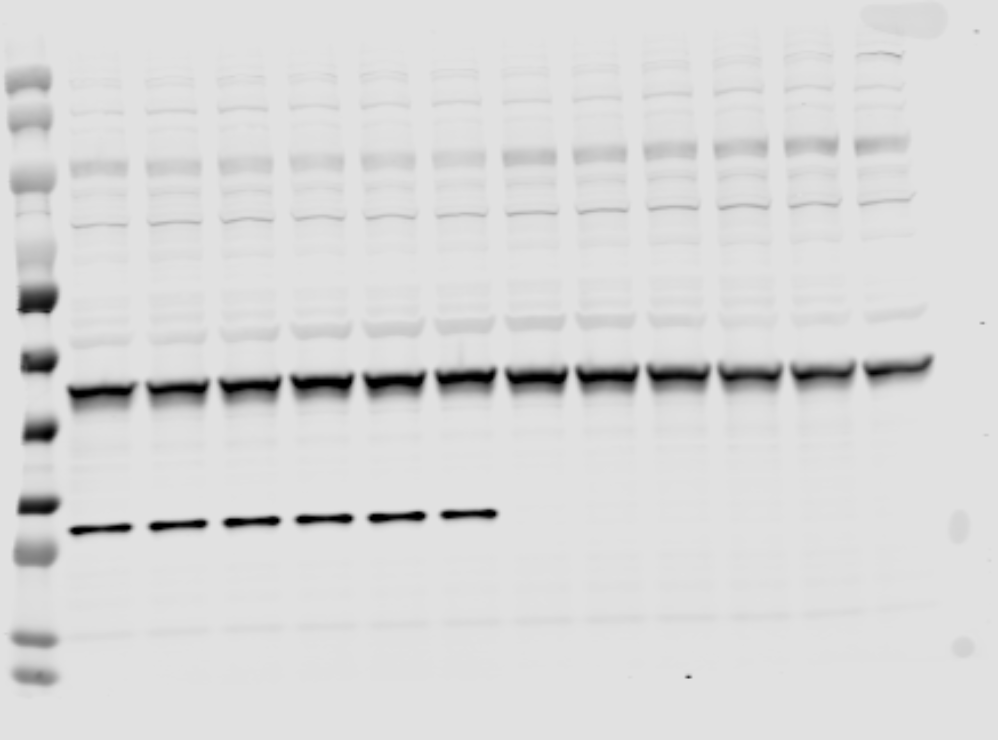

Supplement: Figure 3—figure supplement 2—source data 1. [file elife-102852-fig3-figsupp2-data1.zip › Figure 3-source data 3/Fig3Supplement2D_eIF2a_original.tif]

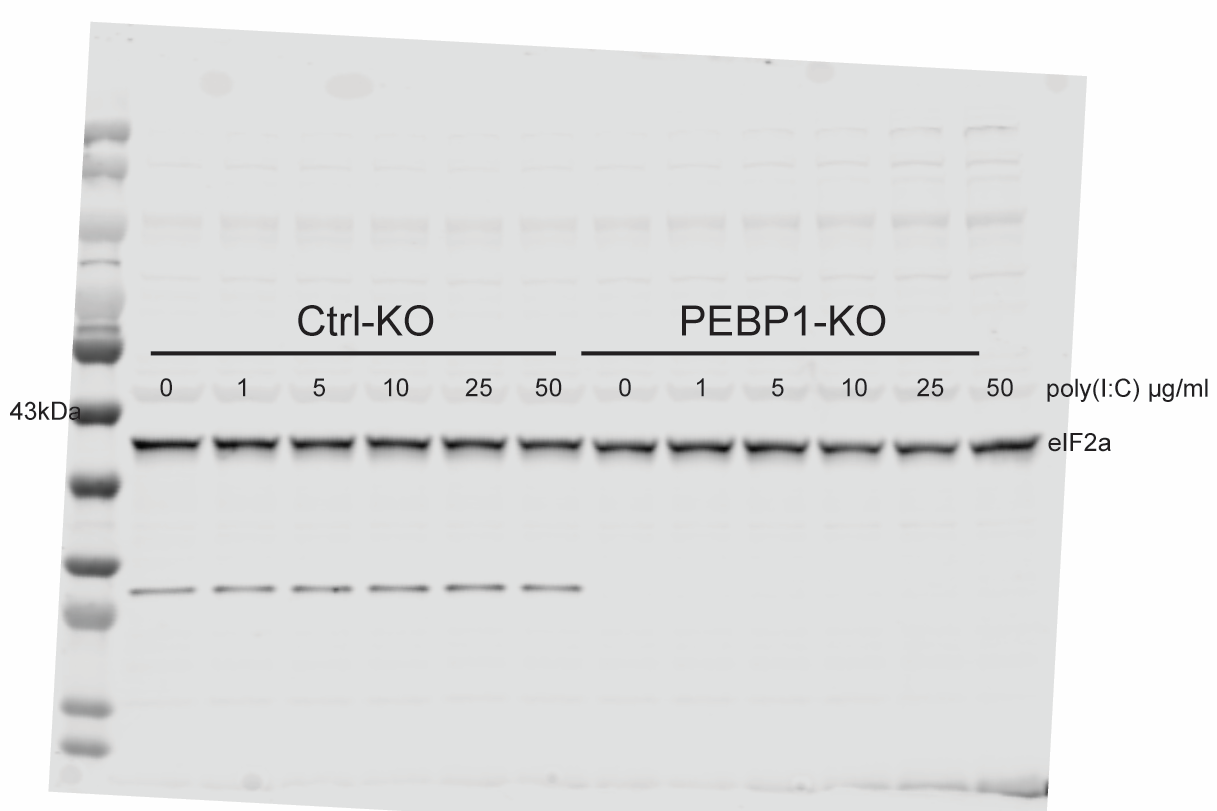

Supplement: Figure 3—figure supplement 2—source data 1. [file elife-102852-fig3-figsupp2-data1.zip › Figure 3-source data 3/Fig3Supplement2F_eIF2a_band_indicated.tif]

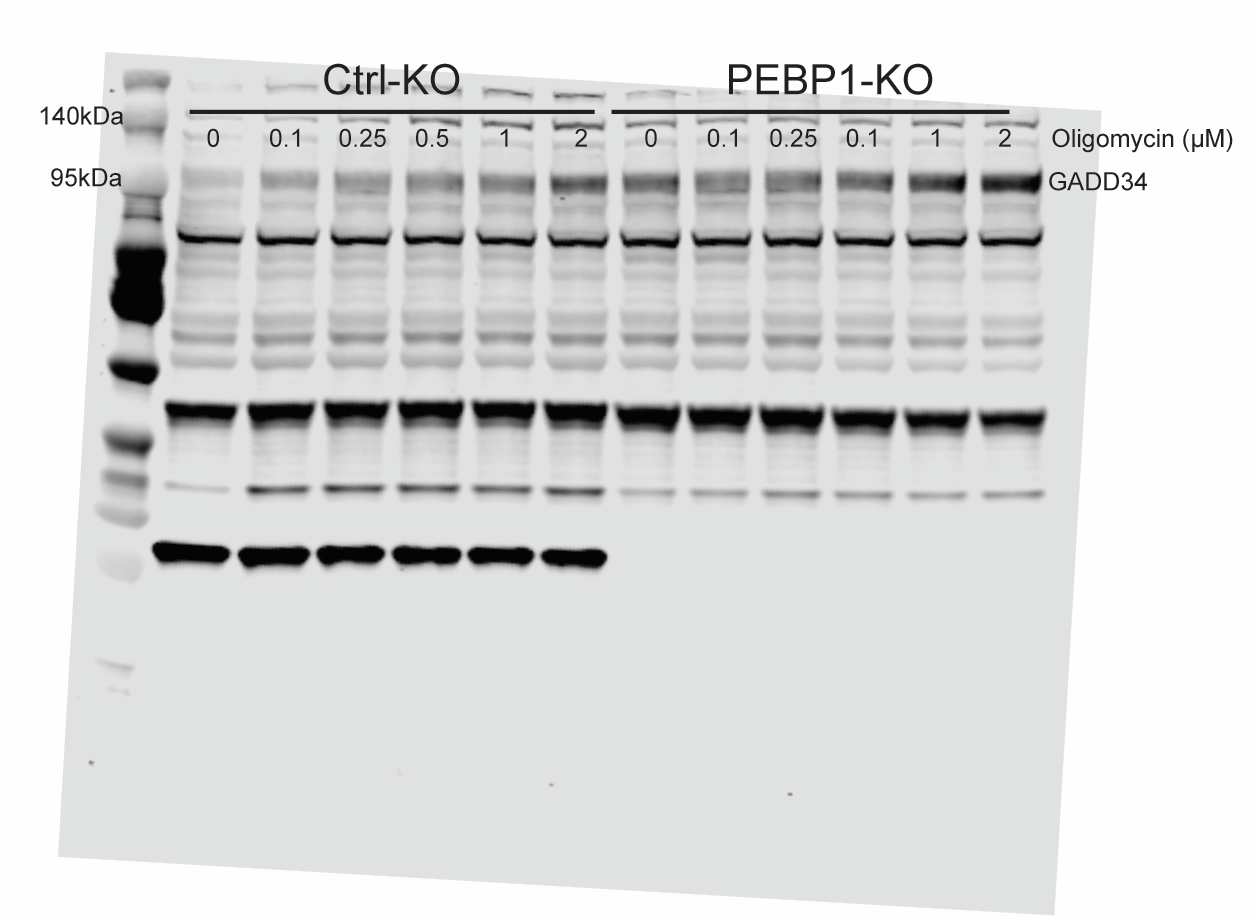

Supplement: Figure 3—figure supplement 2—source data 1. [file elife-102852-fig3-figsupp2-data1.zip › Figure 3-source data 3/Fig3Supplement2C_GADD34_band_indicated.tif]

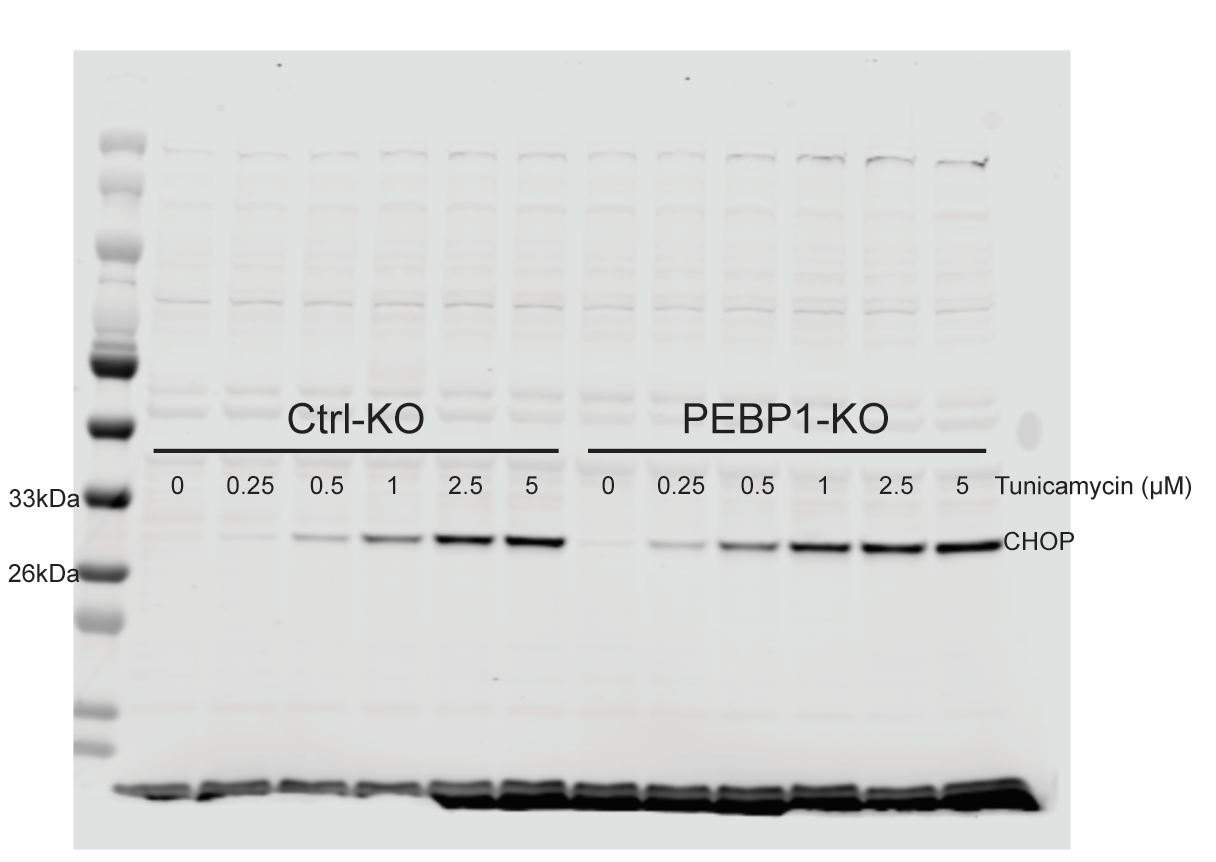

Supplement: Figure 3—figure supplement 2—source data 1. [file elife-102852-fig3-figsupp2-data1.zip › Figure 3-source data 3/Fig3Supplement2G_CHOP_band_indicated.tif]

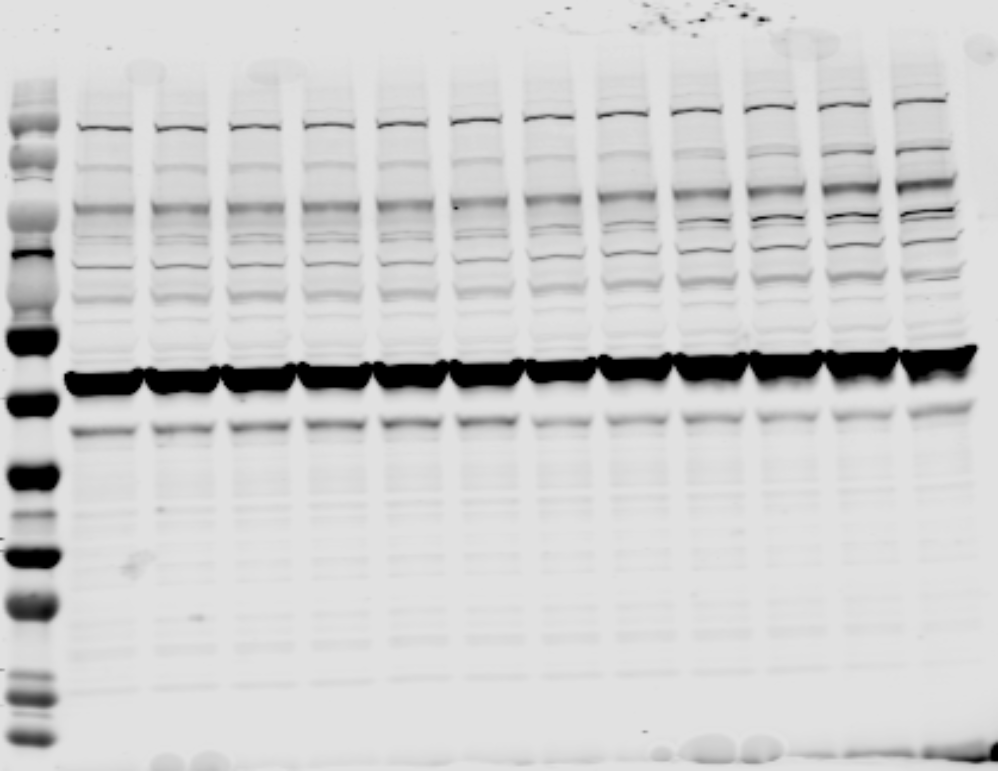

Supplement: Figure 3—figure supplement 2—source data 1. [file elife-102852-fig3-figsupp2-data1.zip › Figure 3-source data 3/Fig3Supplement2F_CREP_original.tif]

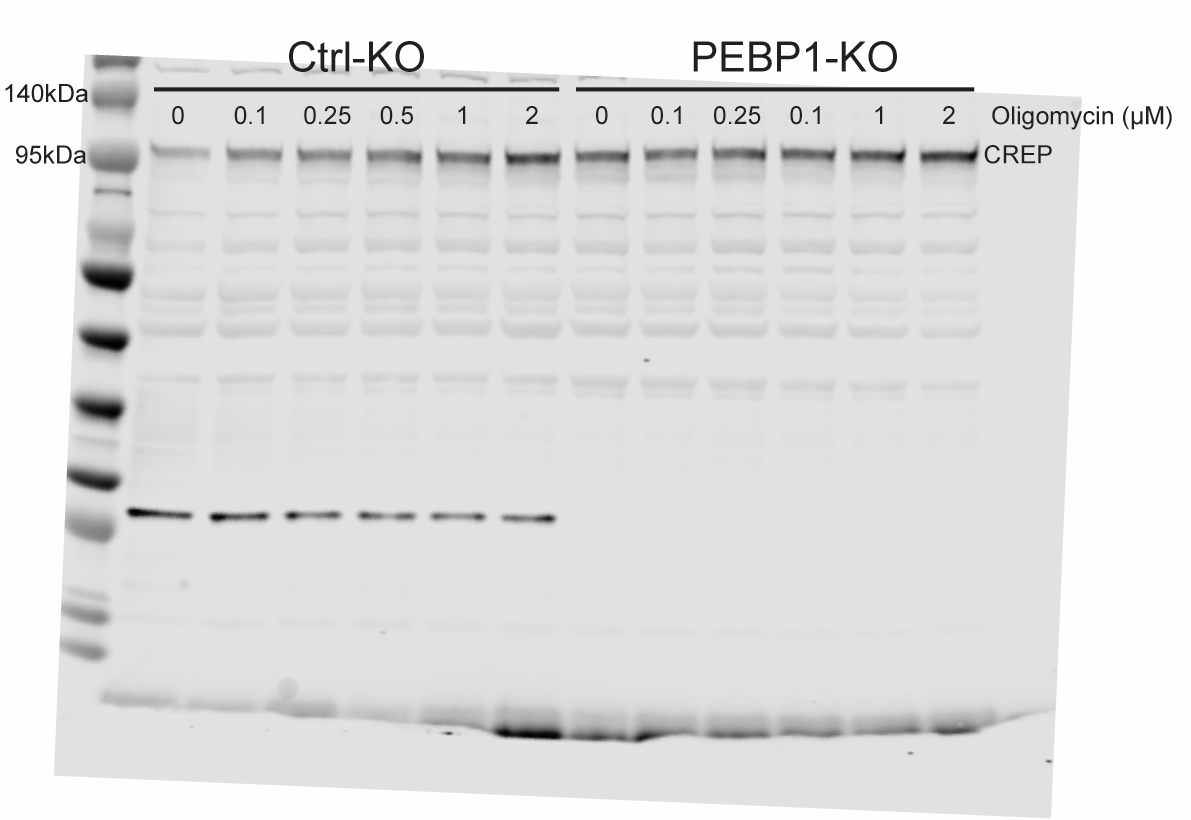

Supplement: Figure 3—figure supplement 2—source data 1. [file elife-102852-fig3-figsupp2-data1.zip › Figure 3-source data 3/Fig3Supplement2C_CREP_band_indicated.tif]

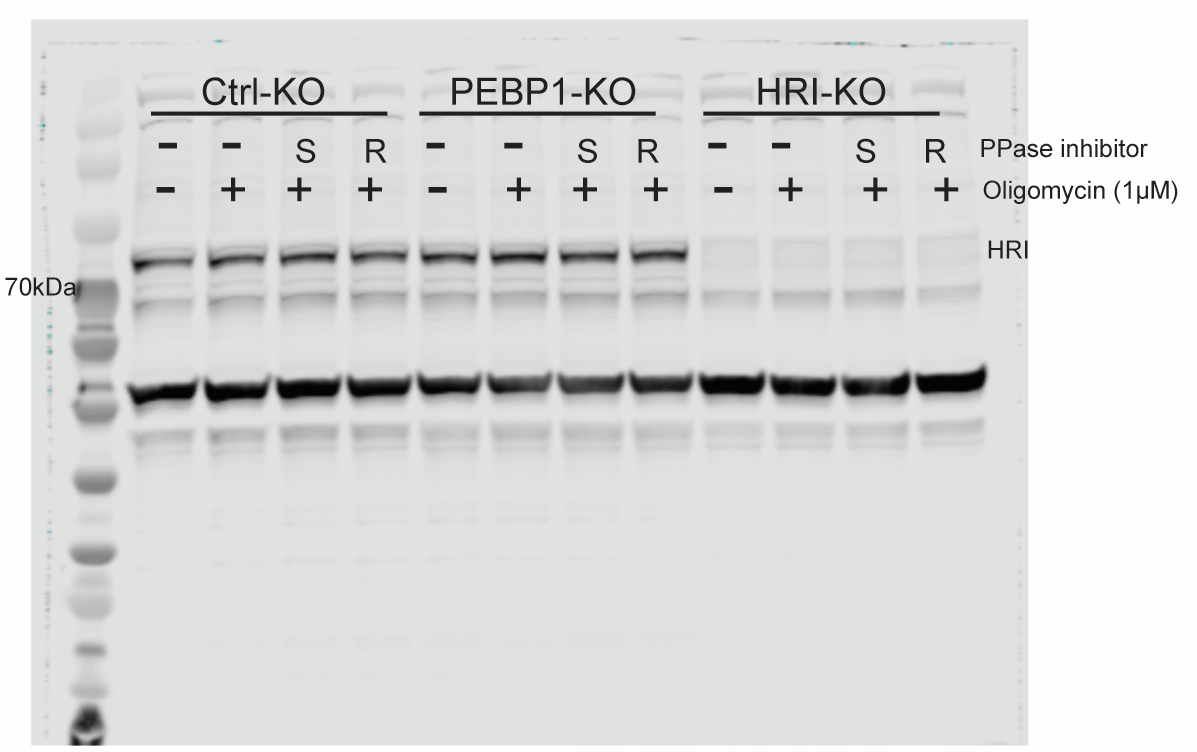

Supplement: Figure 3—figure supplement 2—source data 1. [file elife-102852-fig3-figsupp2-data1.zip › Figure 3-source data 3/Fig3Supplement2H_HRI_band_indicated.tif]

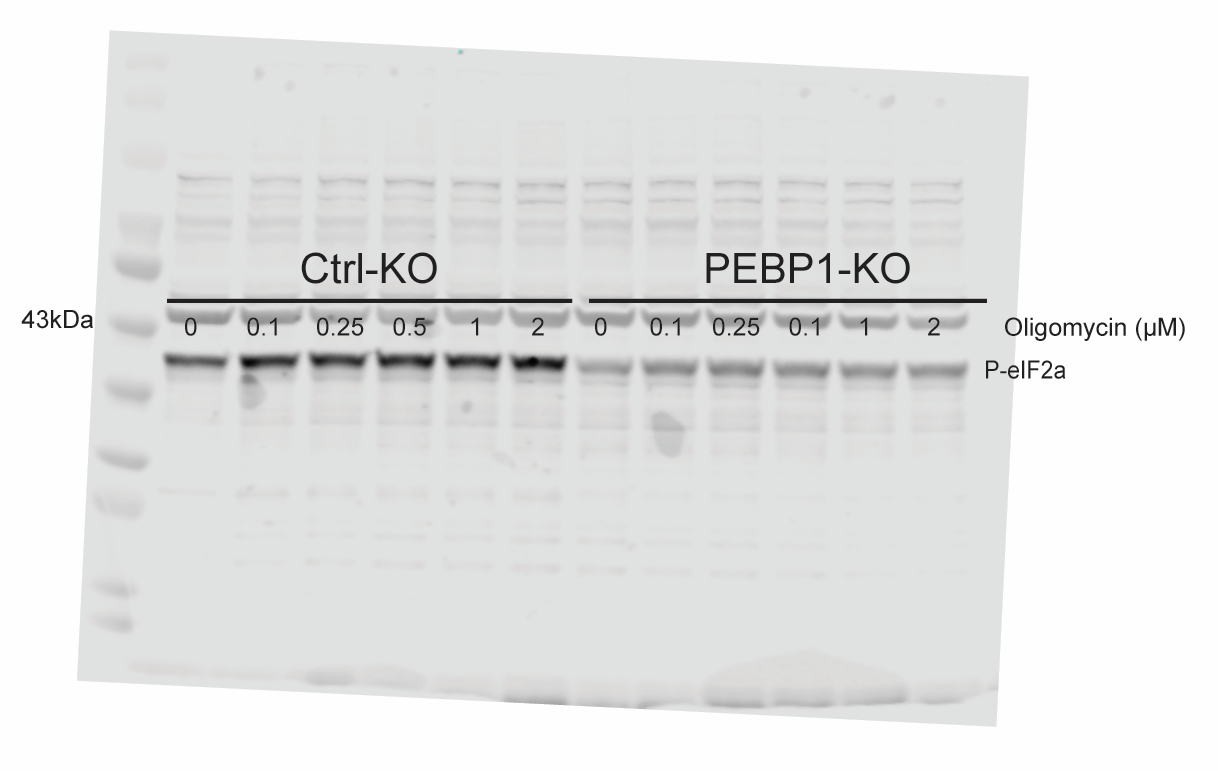

Supplement: Figure 3—figure supplement 2—source data 1. [file elife-102852-fig3-figsupp2-data1.zip › Figure 3-source data 3/Fig3Supplement2C_P-eIF2a_band_indicated.tif]

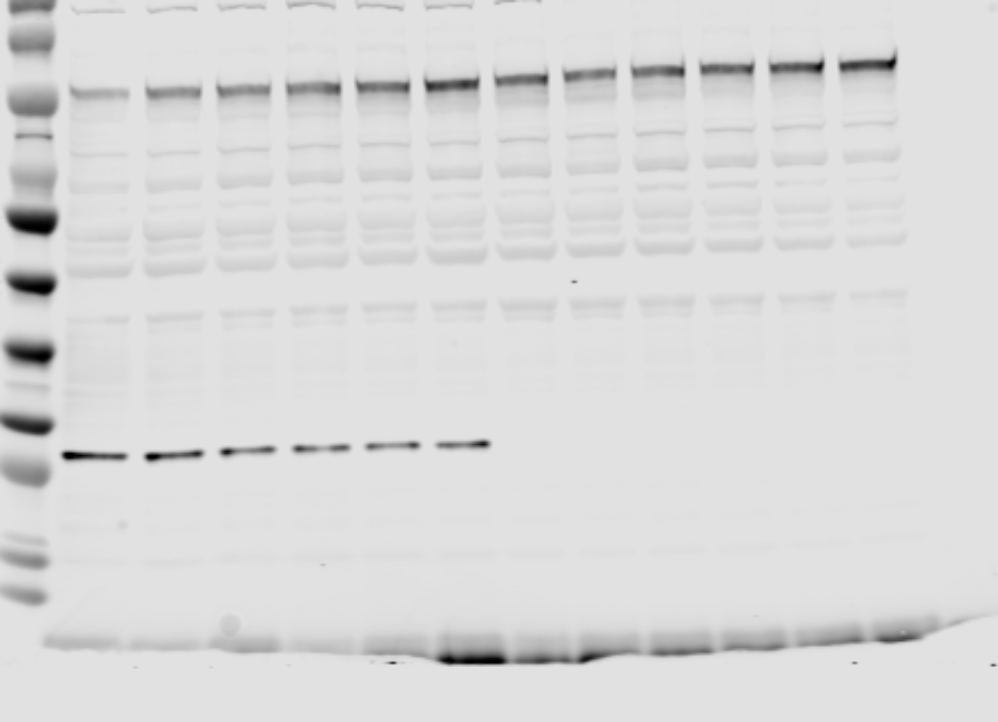

Supplement: Figure 3—figure supplement 2—source data 1. [file elife-102852-fig3-figsupp2-data1.zip › Figure 3-source data 3/Fig3Supplement2C_CREP_original.tif]

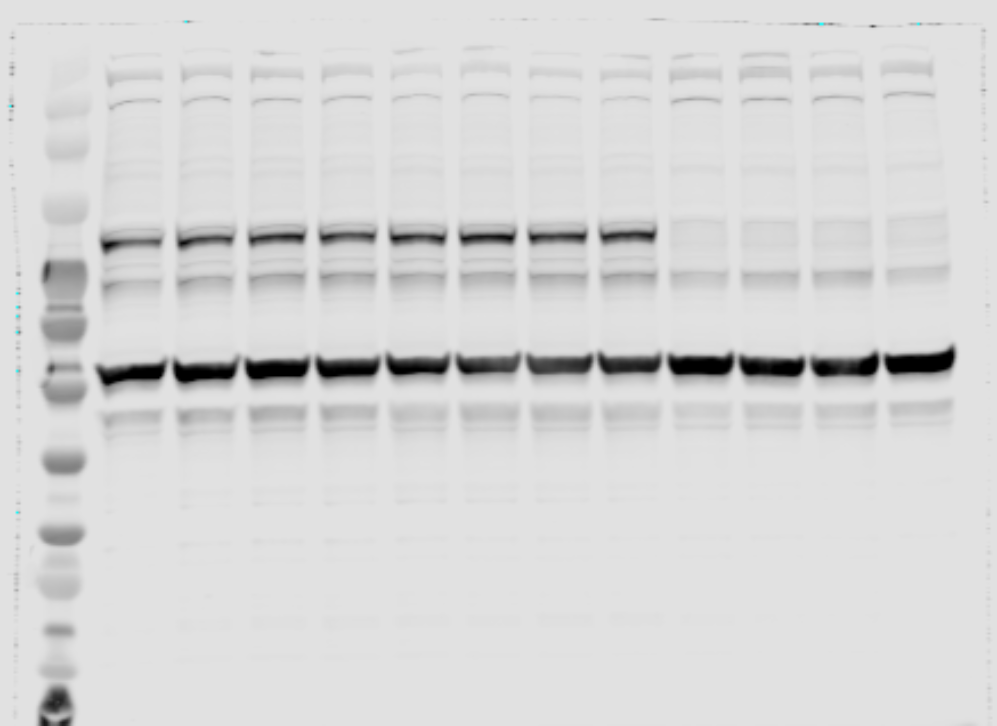

Supplement: Figure 3—figure supplement 2—source data 1. [file elife-102852-fig3-figsupp2-data1.zip › Figure 3-source data 3/Fig3Supplement2H_HRI_original.tif]

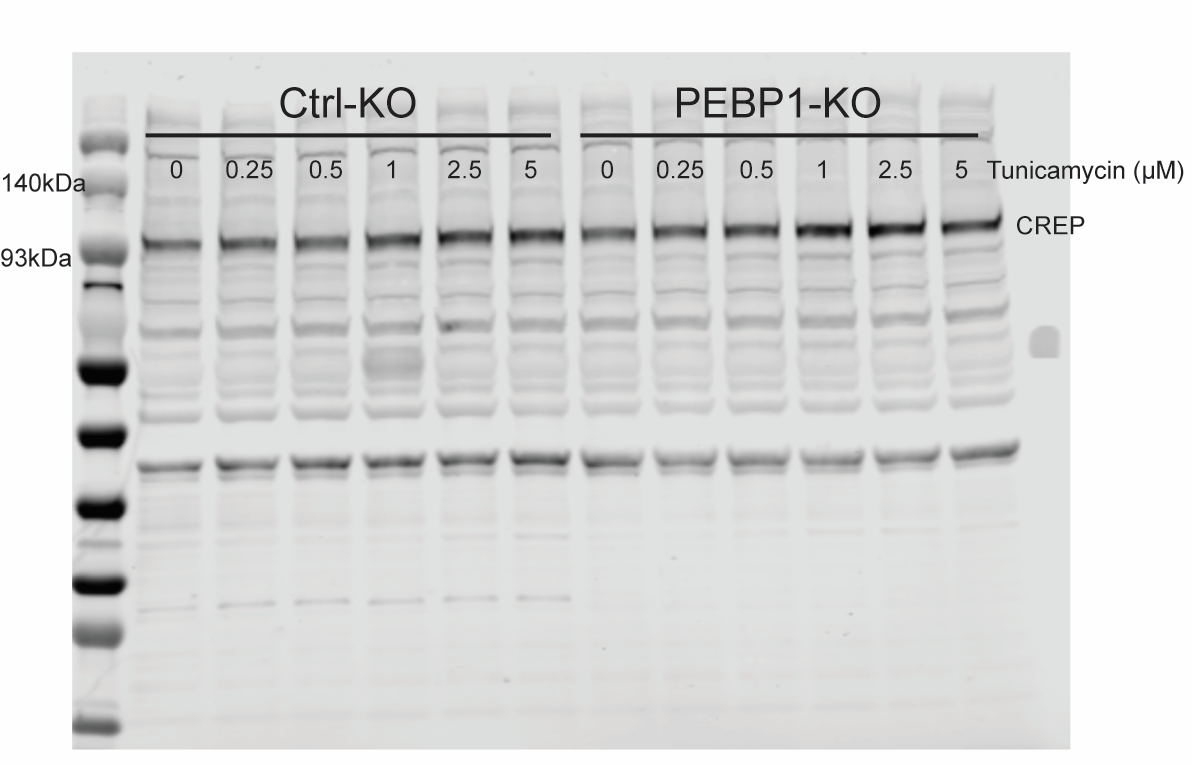

Supplement: Figure 3—figure supplement 2—source data 1. [file elife-102852-fig3-figsupp2-data1.zip › Figure 3-source data 3/Fig3Supplement2G_CREP_band_indicated.tif]

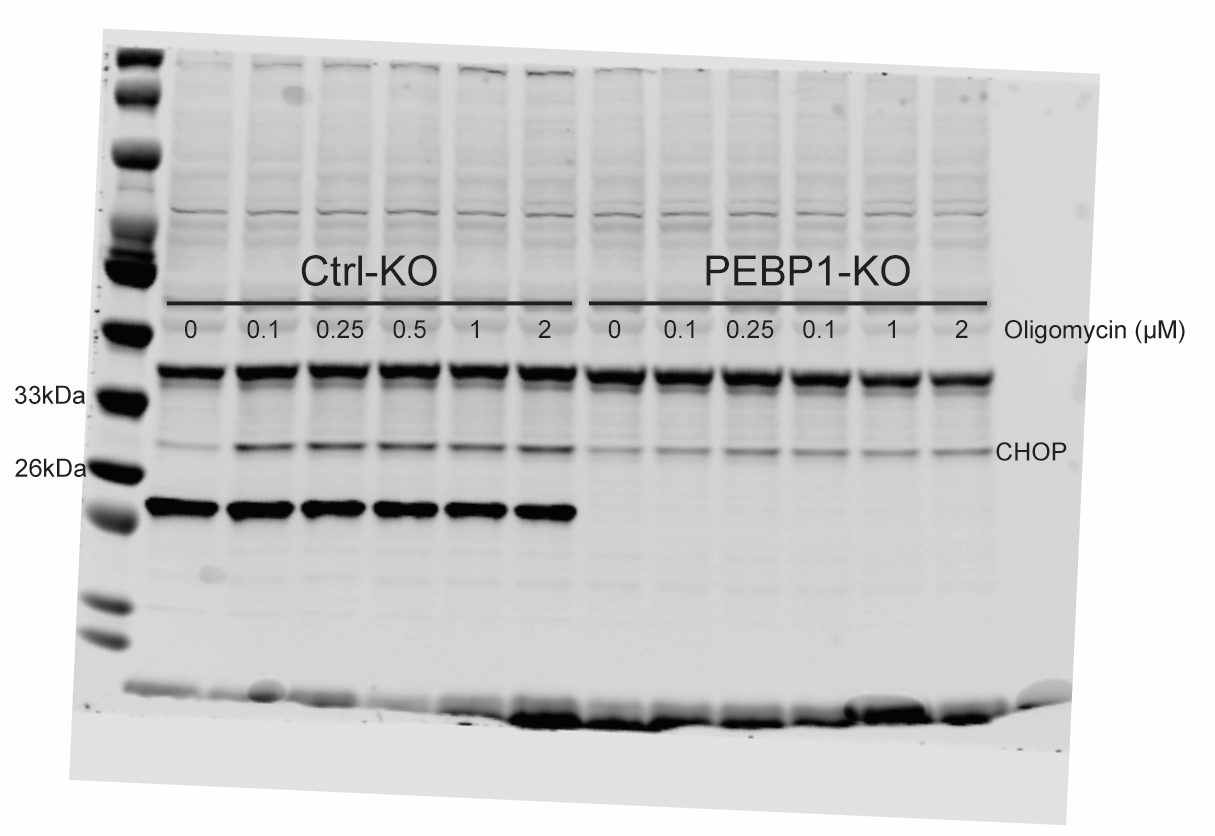

Supplement: Figure 3—figure supplement 2—source data 1. [file elife-102852-fig3-figsupp2-data1.zip › Figure 3-source data 3/Fig3Supplement2C_CHOP_band_indicated.tif]

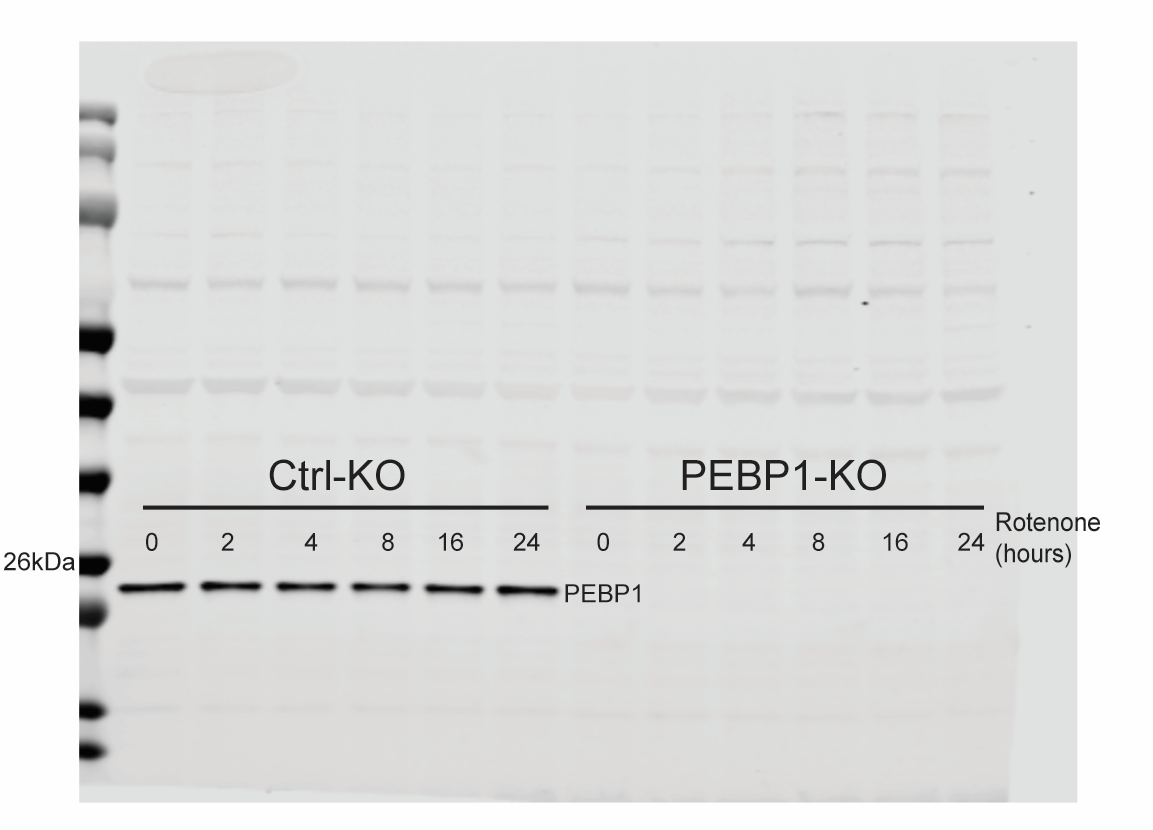

Supplement: Figure 3—figure supplement 2—source data 1. [file elife-102852-fig3-figsupp2-data1.zip › Figure 3-source data 3/Fig3Supplement2E_PEBP1_band_indicated.tif]

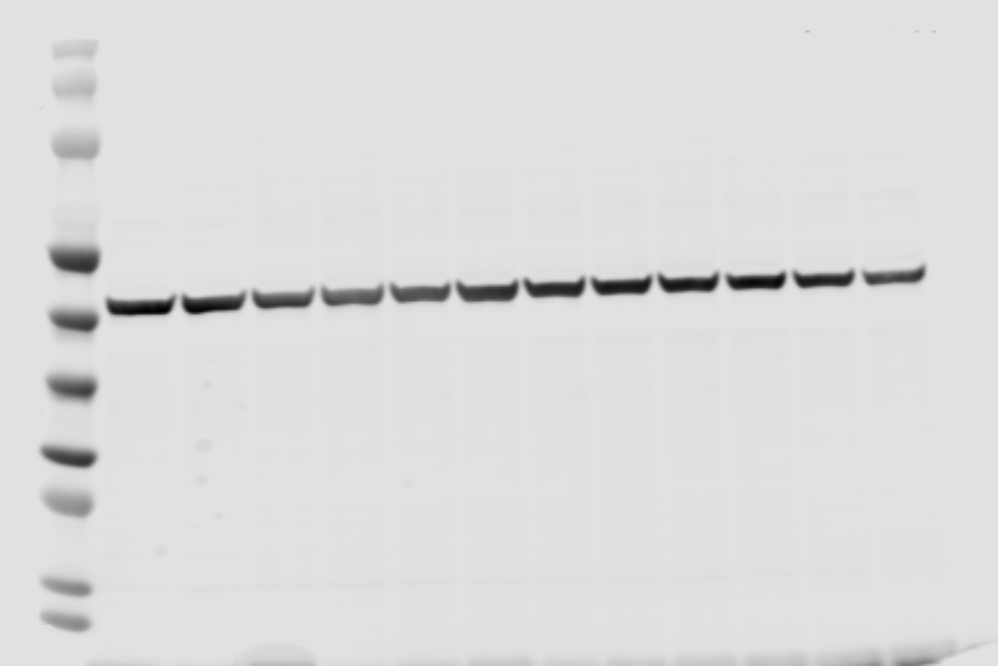

Supplement: Figure 3—figure supplement 2—source data 1. [file elife-102852-fig3-figsupp2-data1.zip › Figure 3-source data 3/Fig3Supplement2C_Actin_original.tif]

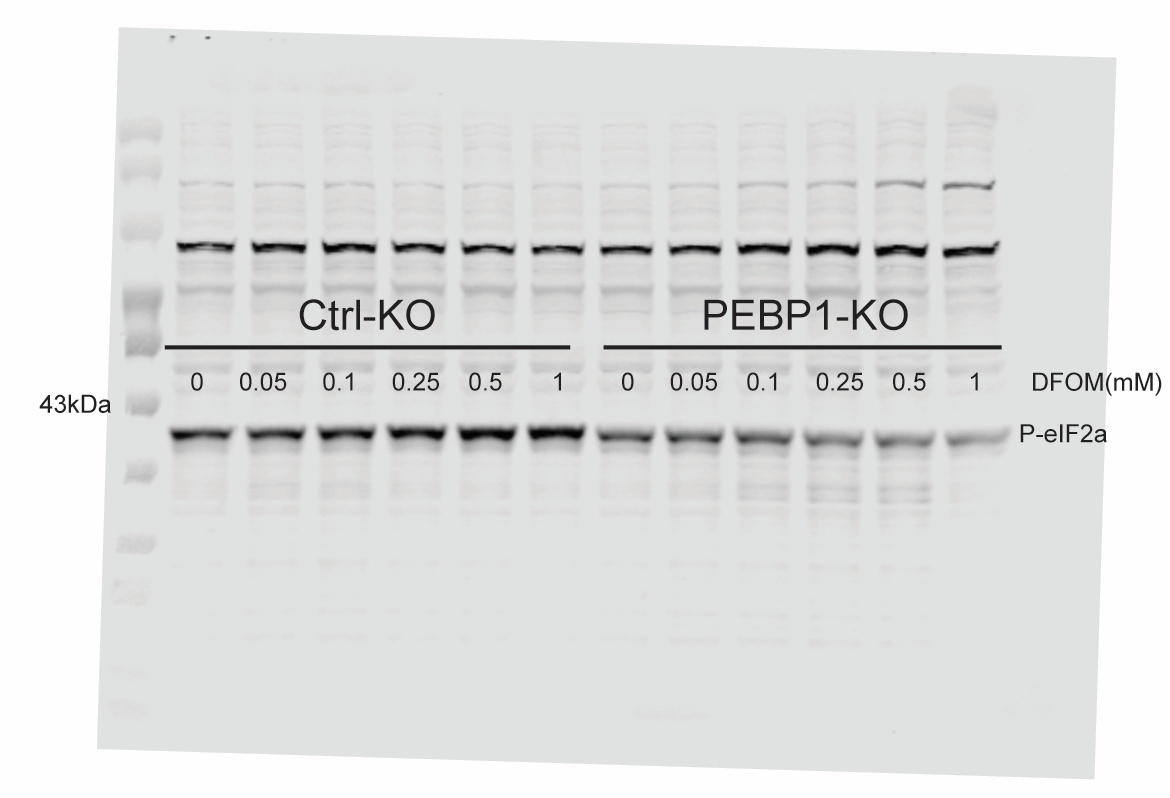

Supplement: Figure 3—figure supplement 2—source data 1. [file elife-102852-fig3-figsupp2-data1.zip › Figure 3-source data 3/Fig3Supplement2D_P-eIF2a_band_indicated.tif]

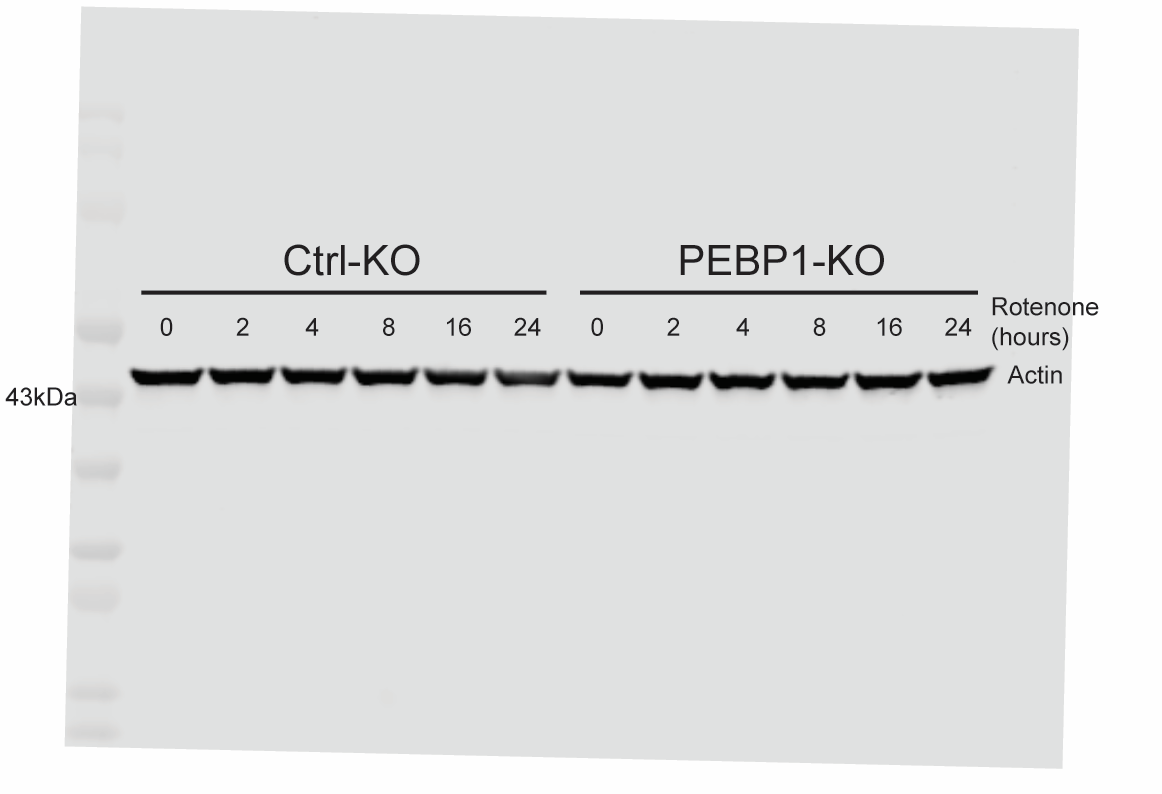

Supplement: Figure 3—figure supplement 2—source data 1. [file elife-102852-fig3-figsupp2-data1.zip › Figure 3-source data 3/Fig3Supplement2E_Actin_band_indicated.tif]

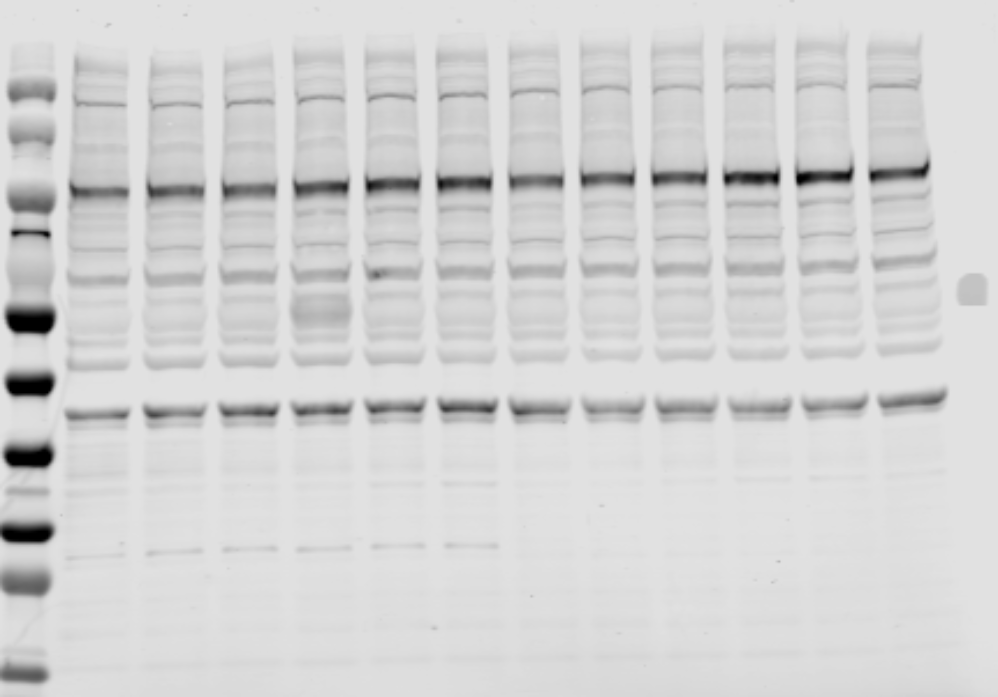

Supplement: Figure 3—figure supplement 2—source data 1. [file elife-102852-fig3-figsupp2-data1.zip › Figure 3-source data 3/Fig3Supplement2G_CREP_original.tif]

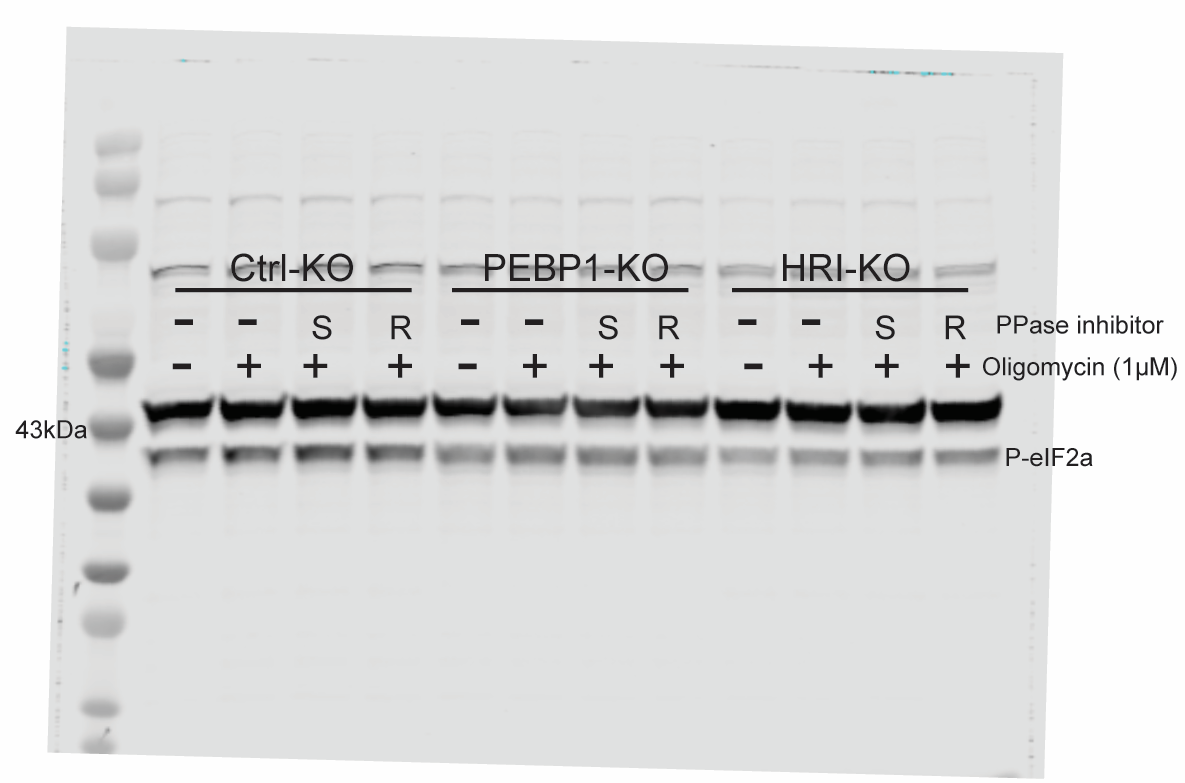

Supplement: Figure 3—figure supplement 2—source data 1. [file elife-102852-fig3-figsupp2-data1.zip › Figure 3-source data 3/Fig3Supplement2H_P-eIF2a_band_indicated.tif]

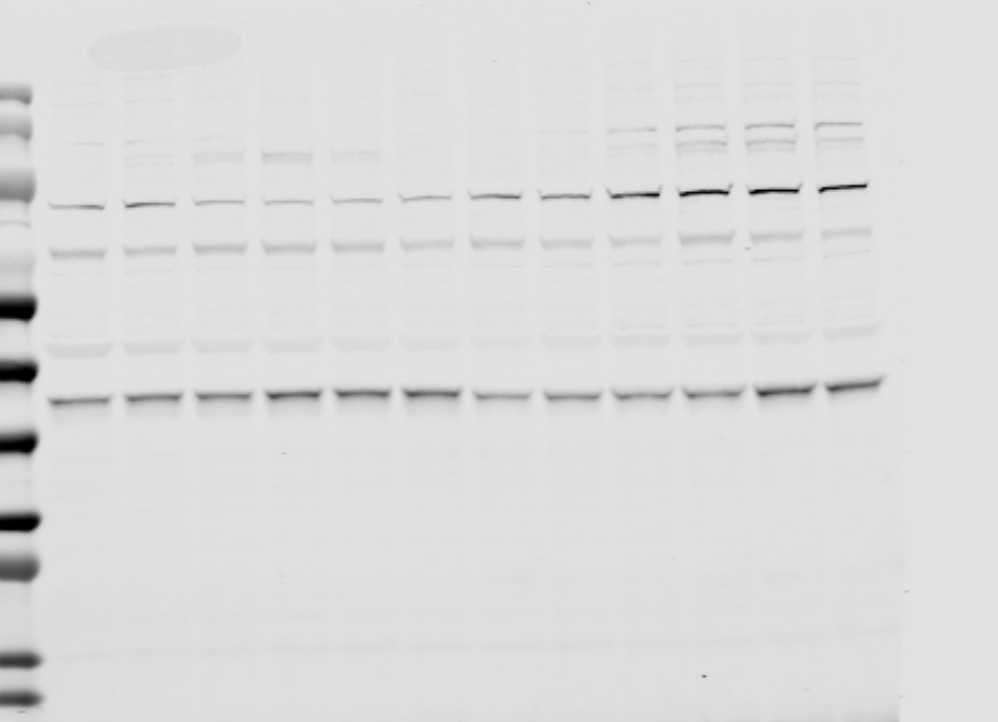

Supplement: Figure 3—figure supplement 2—source data 1. [file elife-102852-fig3-figsupp2-data1.zip › Figure 3-source data 3/Fig3Supplement2E_P-eIF2a_original.tif]

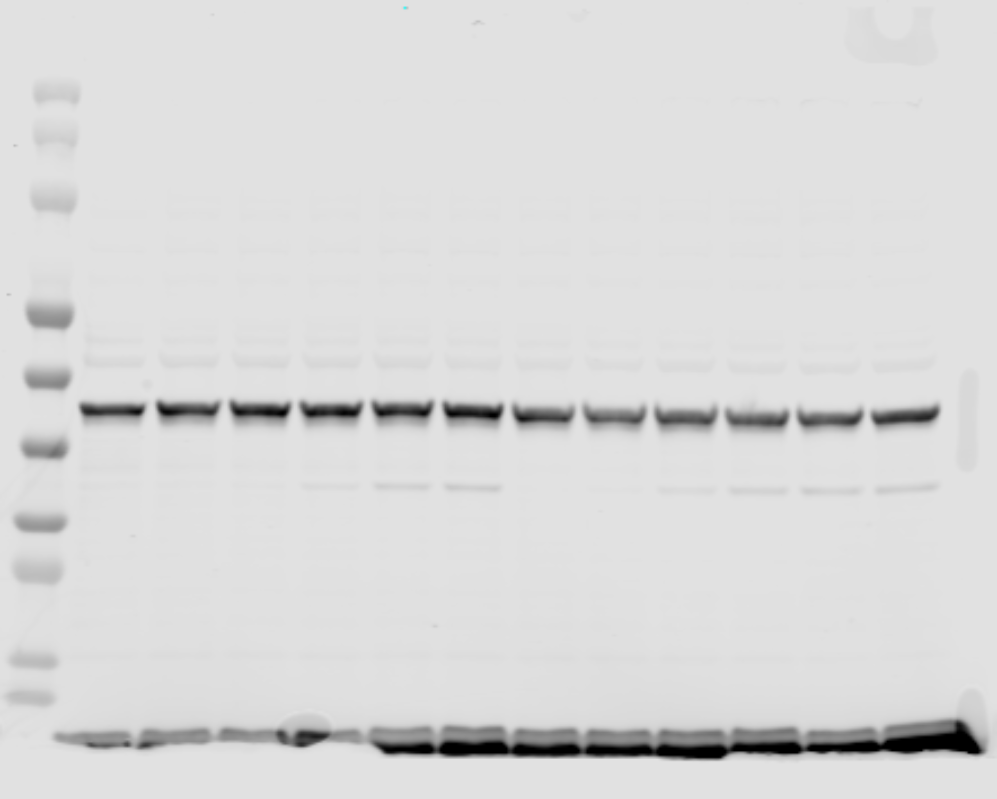

Supplement: Figure 3—figure supplement 2—source data 1. [file elife-102852-fig3-figsupp2-data1.zip › Figure 3-source data 3/Fig3Supplement2G_eIF2a_original.tif]

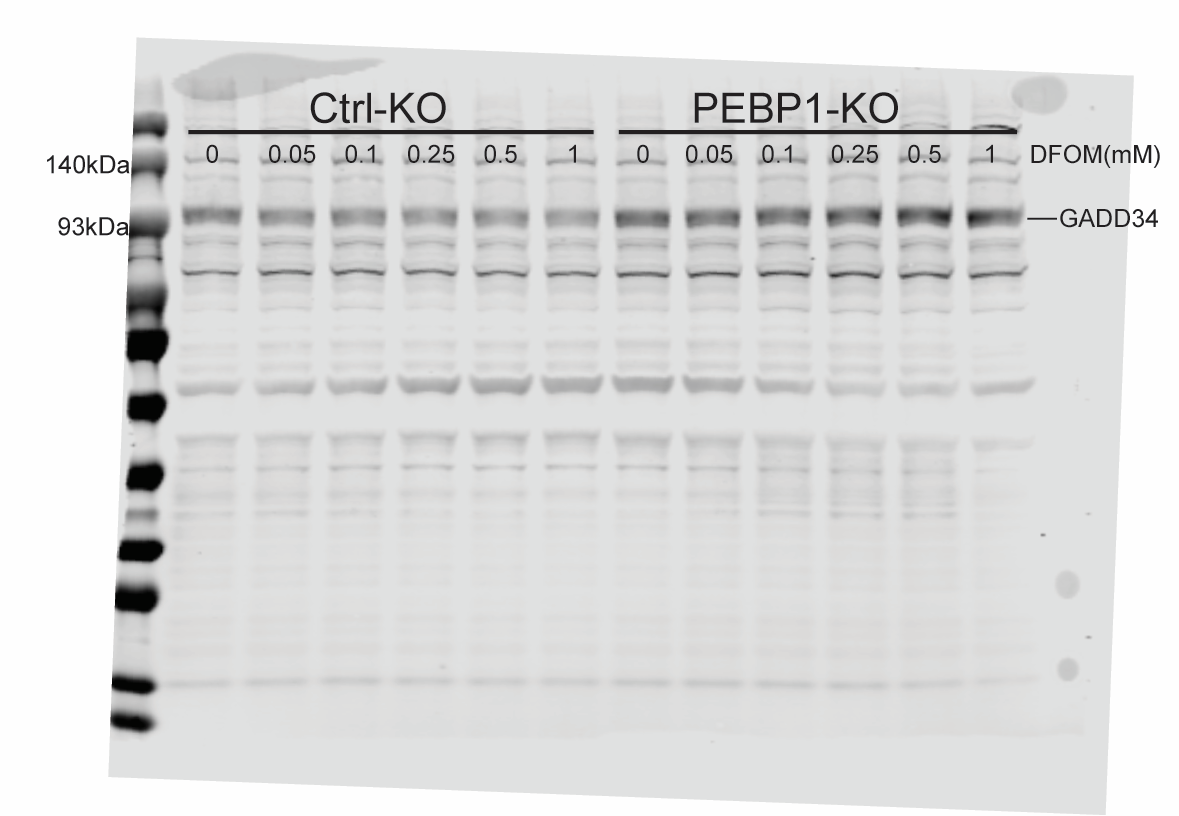

Supplement: Figure 3—figure supplement 2—source data 1. [file elife-102852-fig3-figsupp2-data1.zip › Figure 3-source data 3/Fig3Supplement2D_GADD34_band_indicated.tif]

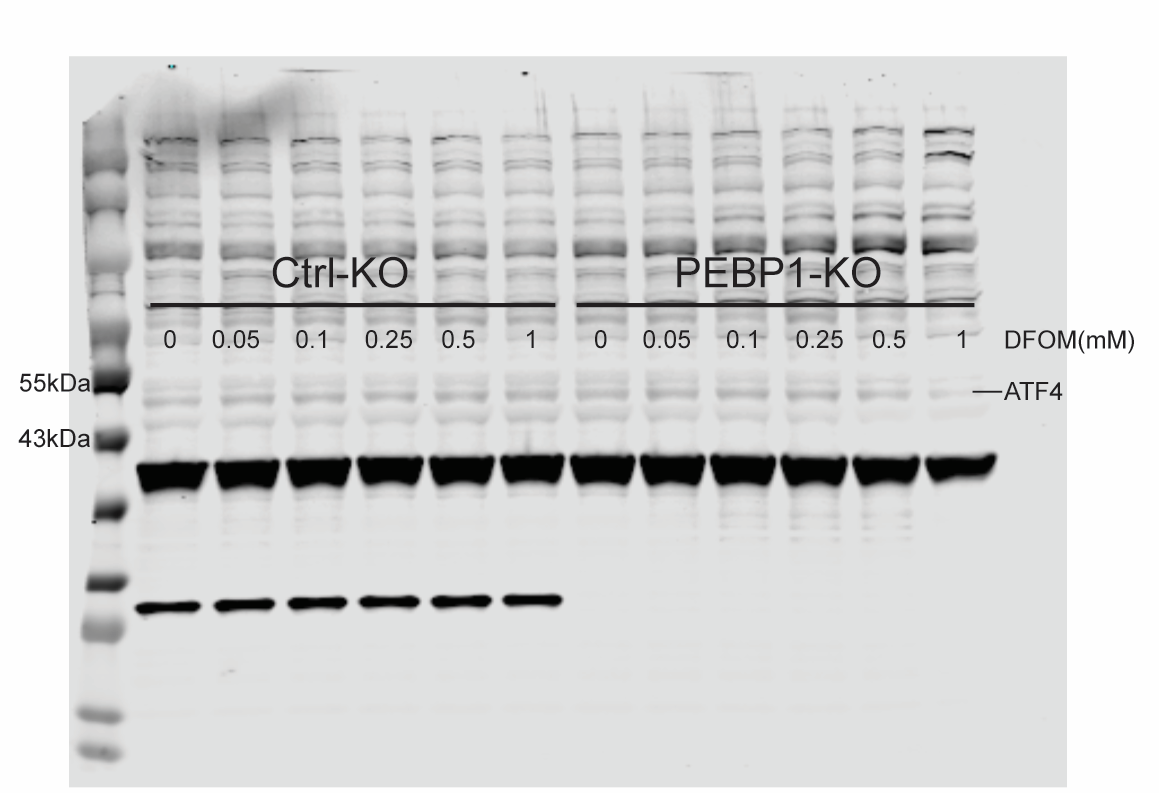

Supplement: Figure 3—figure supplement 2—source data 1. [file elife-102852-fig3-figsupp2-data1.zip › Figure 3-source data 3/Fig3Supplement2D_ATF4_band_indicated.tif]

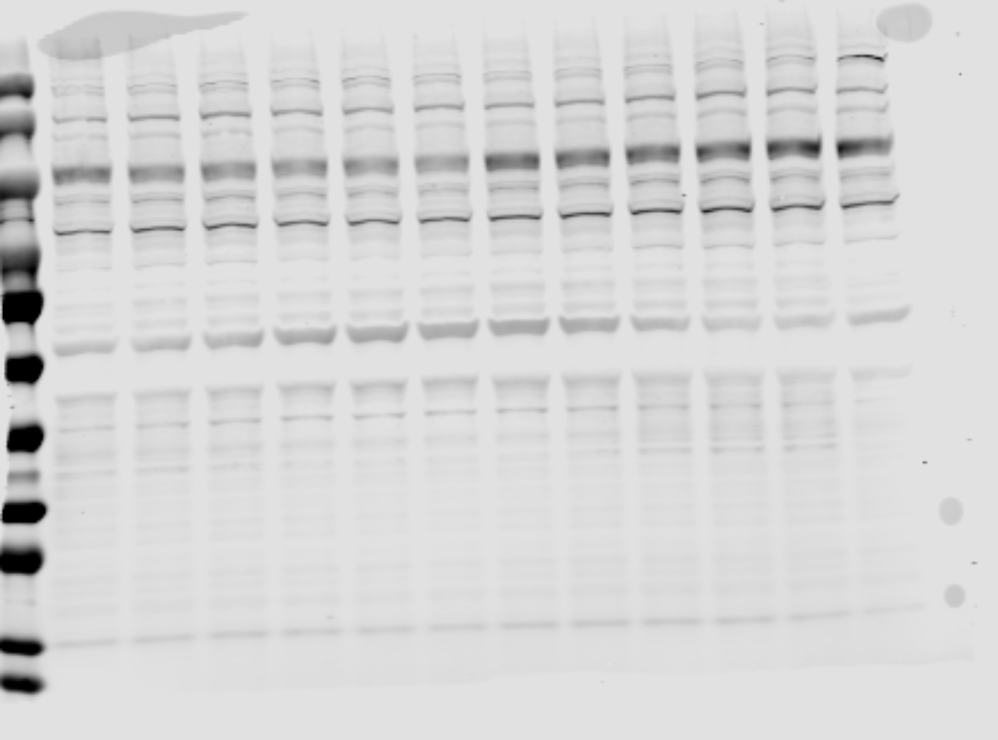

Supplement: Figure 3—figure supplement 2—source data 1. [file elife-102852-fig3-figsupp2-data1.zip › Figure 3-source data 3/Fig3Supplement2D_GADD34_original.tif]

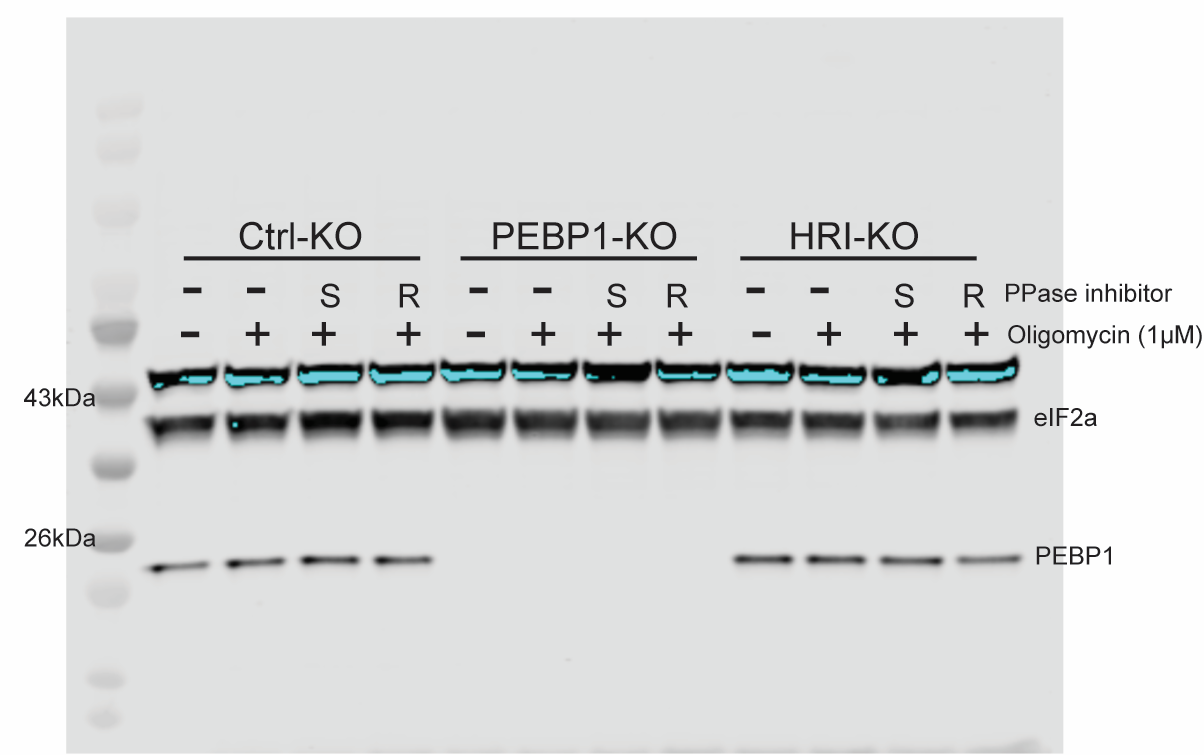

Supplement: Figure 3—figure supplement 2—source data 1. [file elife-102852-fig3-figsupp2-data1.zip › Figure 3-source data 3/Fig3Supplement2H_eIF2a_PEBP1_band_indicated.tif]

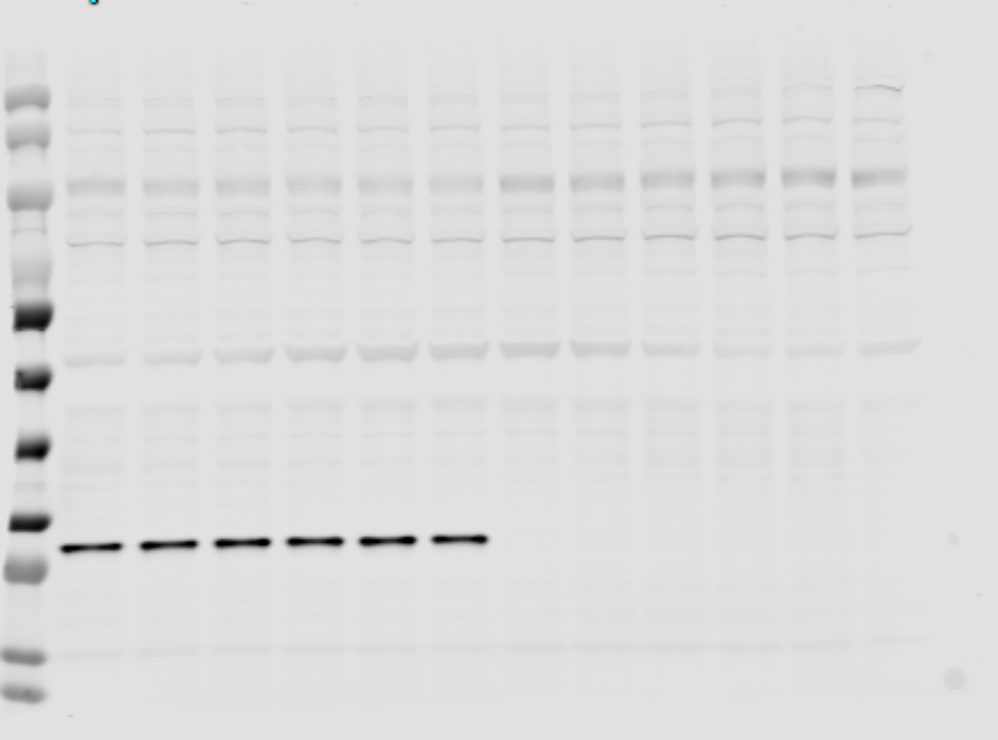

Supplement: Figure 3—figure supplement 2—source data 1. [file elife-102852-fig3-figsupp2-data1.zip › Figure 3-source data 3/Fig3Supplement2D_PEBP1_original.tif]

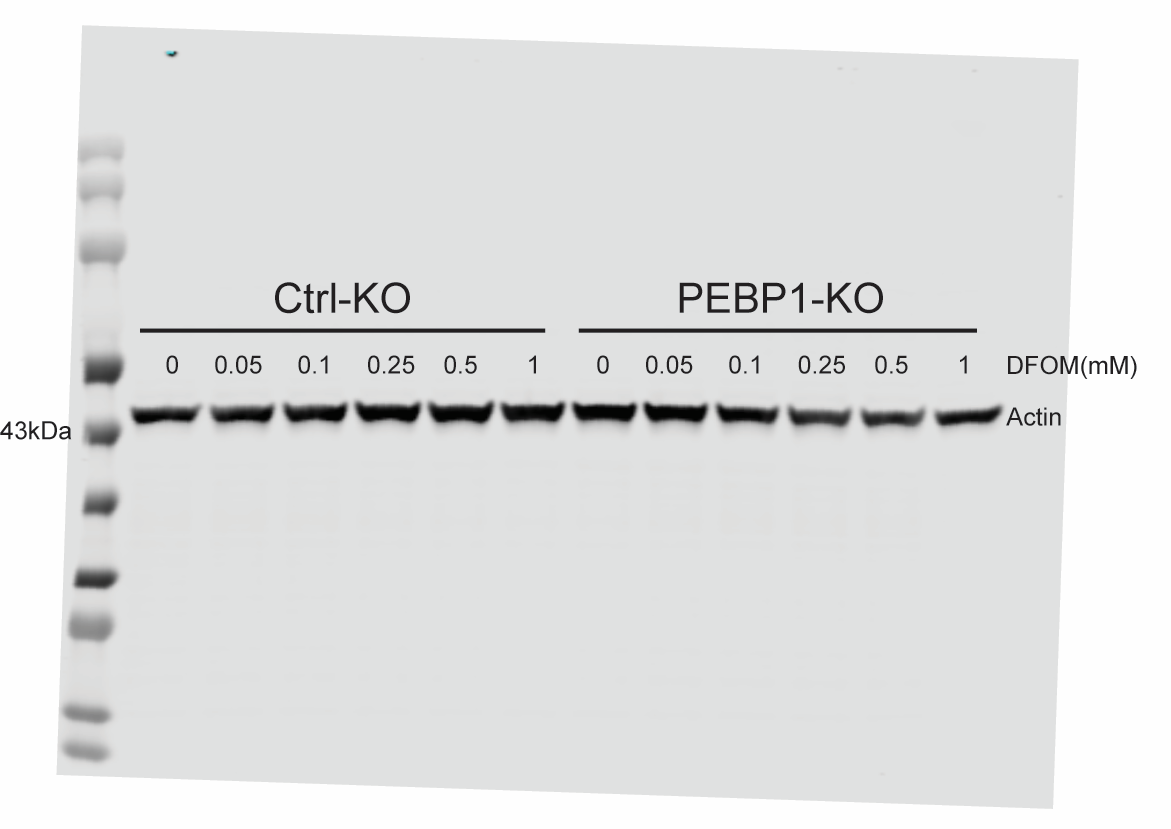

Supplement: Figure 3—figure supplement 2—source data 1. [file elife-102852-fig3-figsupp2-data1.zip › Figure 3-source data 3/Fig3Supplement2D_Actin_band_indicated.tif]

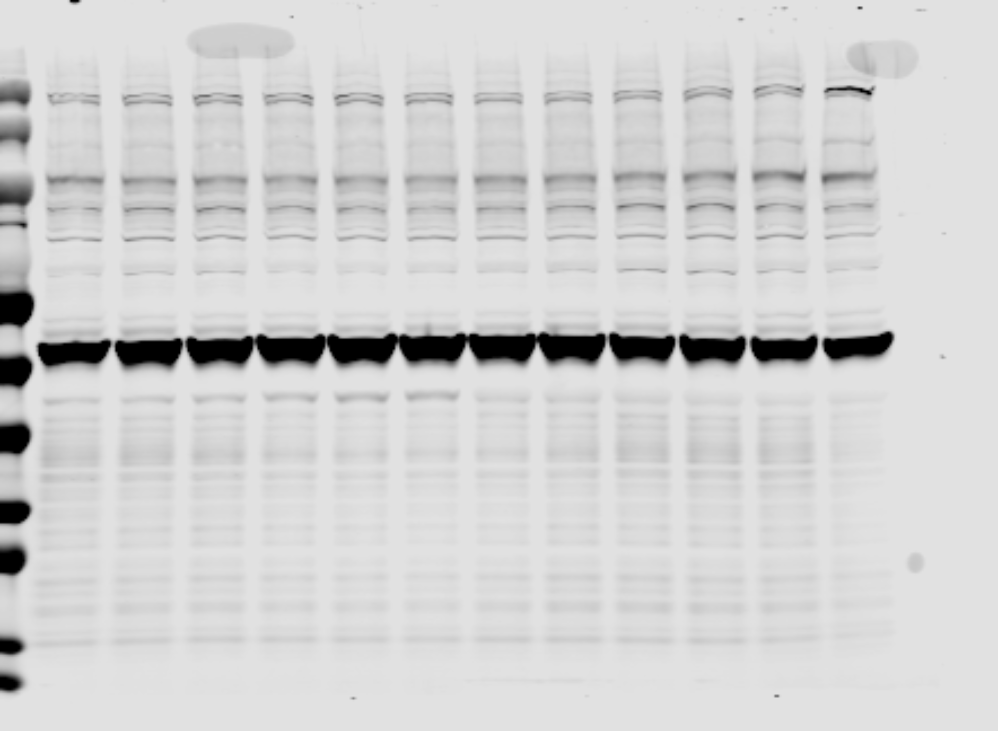

Supplement: Figure 3—figure supplement 2—source data 1. [file elife-102852-fig3-figsupp2-data1.zip › Figure 3-source data 3/Fig3Supplement2D_CREP_original.tif]

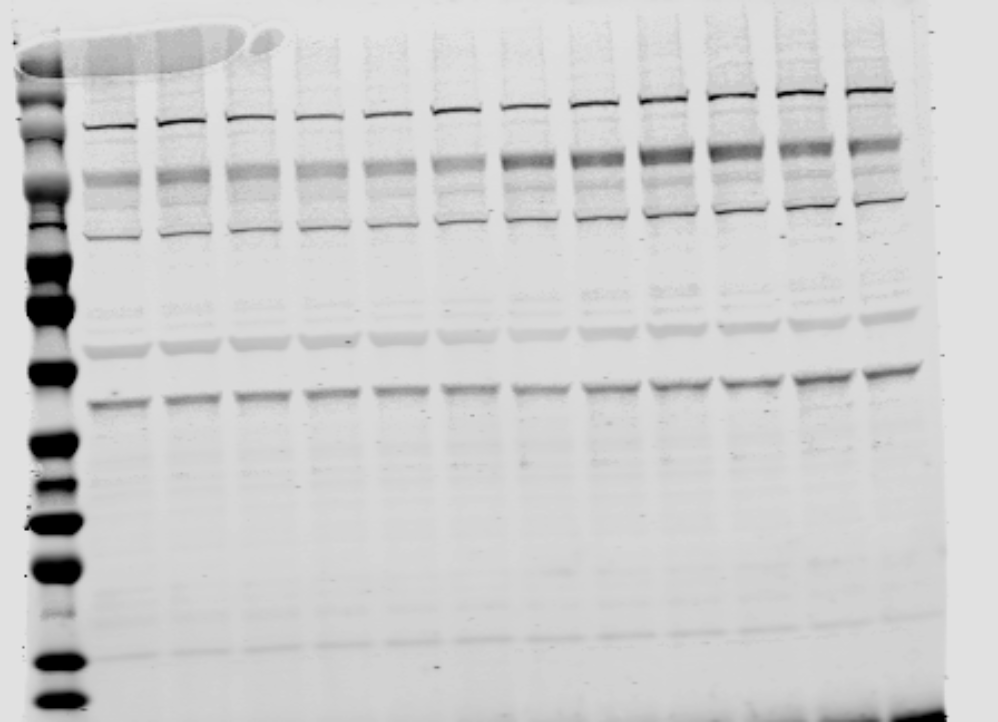

Supplement: Figure 3—figure supplement 2—source data 1. [file elife-102852-fig3-figsupp2-data1.zip › Figure 3-source data 3/Fig3Supplement2E_GADD34_original.tif]

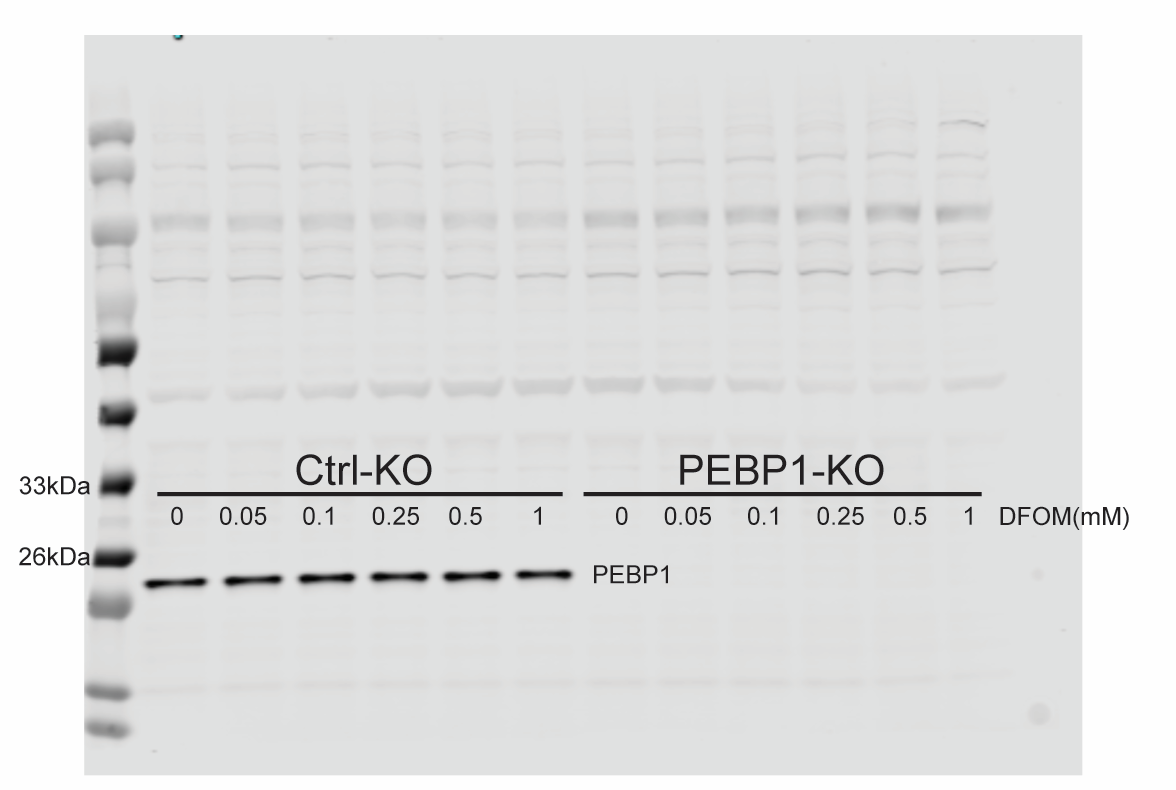

Supplement: Figure 3—figure supplement 2—source data 1. [file elife-102852-fig3-figsupp2-data1.zip › Figure 3-source data 3/Fig3Supplement2D_PEBP1_band_indicated.tif]

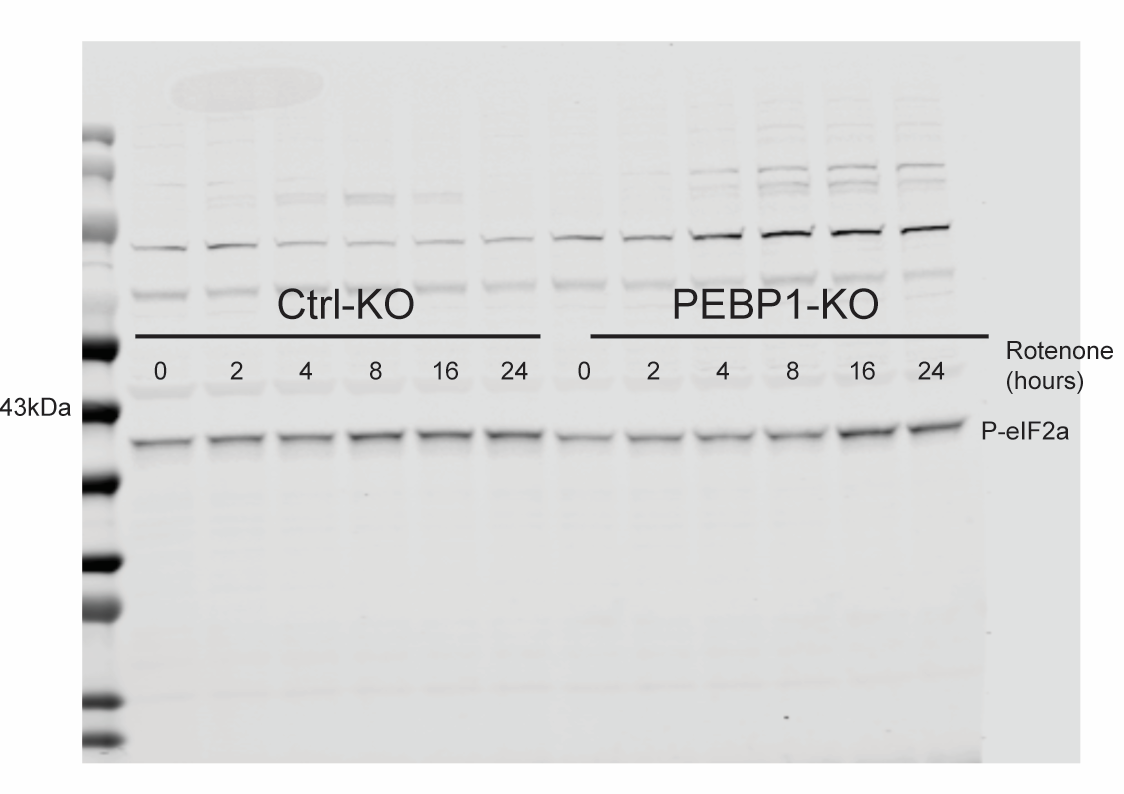

Supplement: Figure 3—figure supplement 2—source data 1. [file elife-102852-fig3-figsupp2-data1.zip › Figure 3-source data 3/Fig3Supplement2E_P-eIF2a_band_indicated.tif]

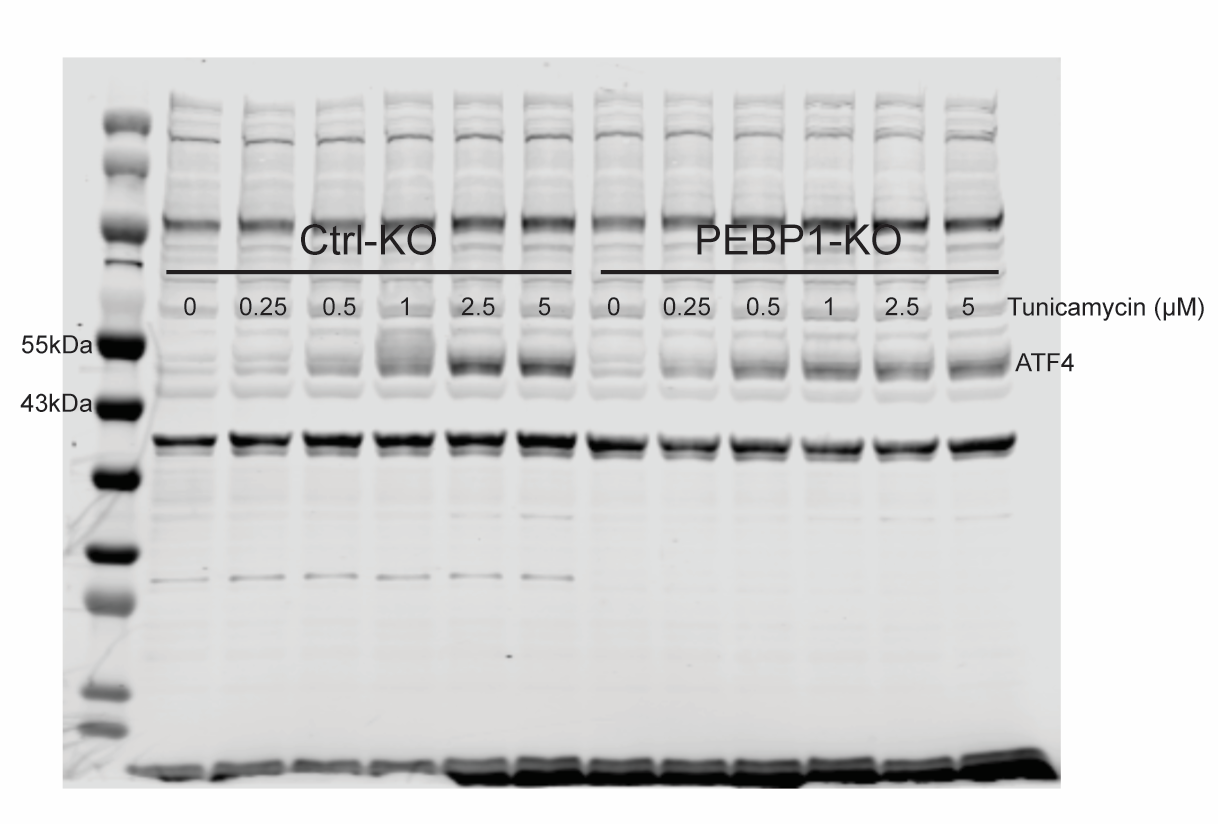

Supplement: Figure 3—figure supplement 2—source data 1. [file elife-102852-fig3-figsupp2-data1.zip › Figure 3-source data 3/Fig3Supplement2G_ATF4_band_indicated.tif]

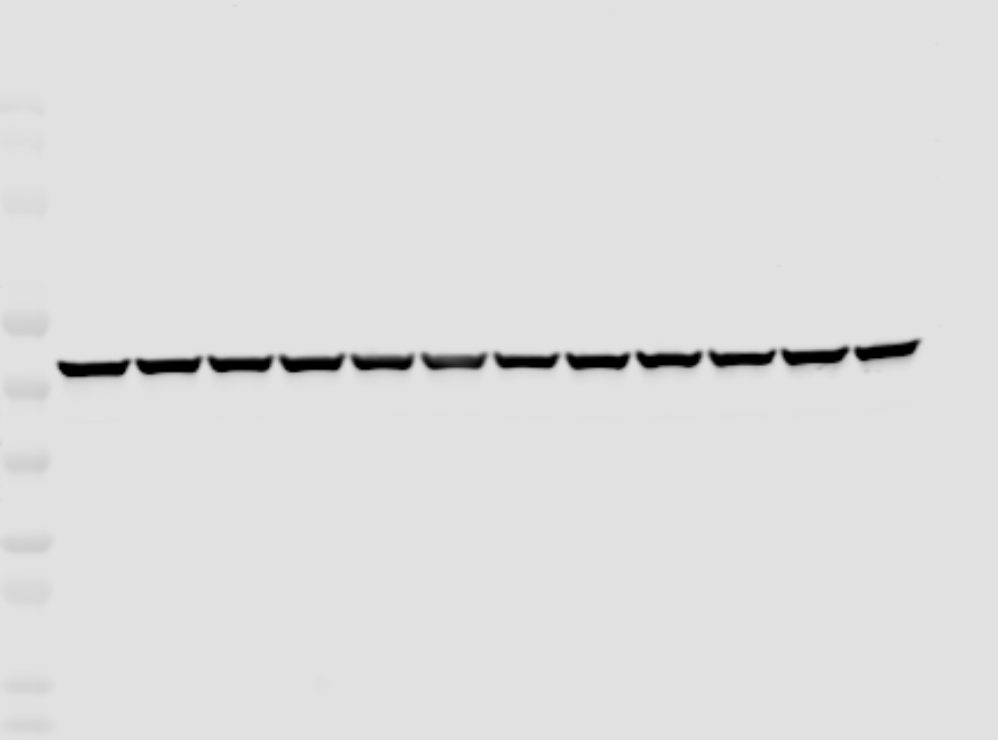

Supplement: Figure 3—figure supplement 2—source data 1. [file elife-102852-fig3-figsupp2-data1.zip › Figure 3-source data 3/Fig3Supplement2E_Actin_original.tif]

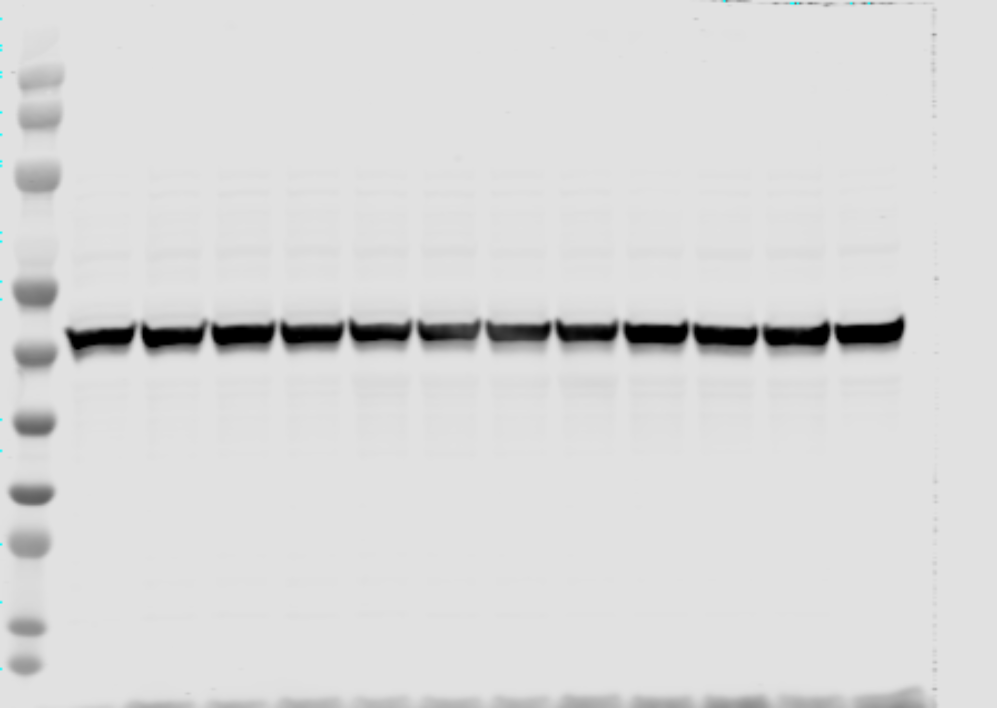

Supplement: Figure 3—figure supplement 2—source data 1. [file elife-102852-fig3-figsupp2-data1.zip › Figure 3-source data 3/Fig3Supplement2H_Actin_original.tif]

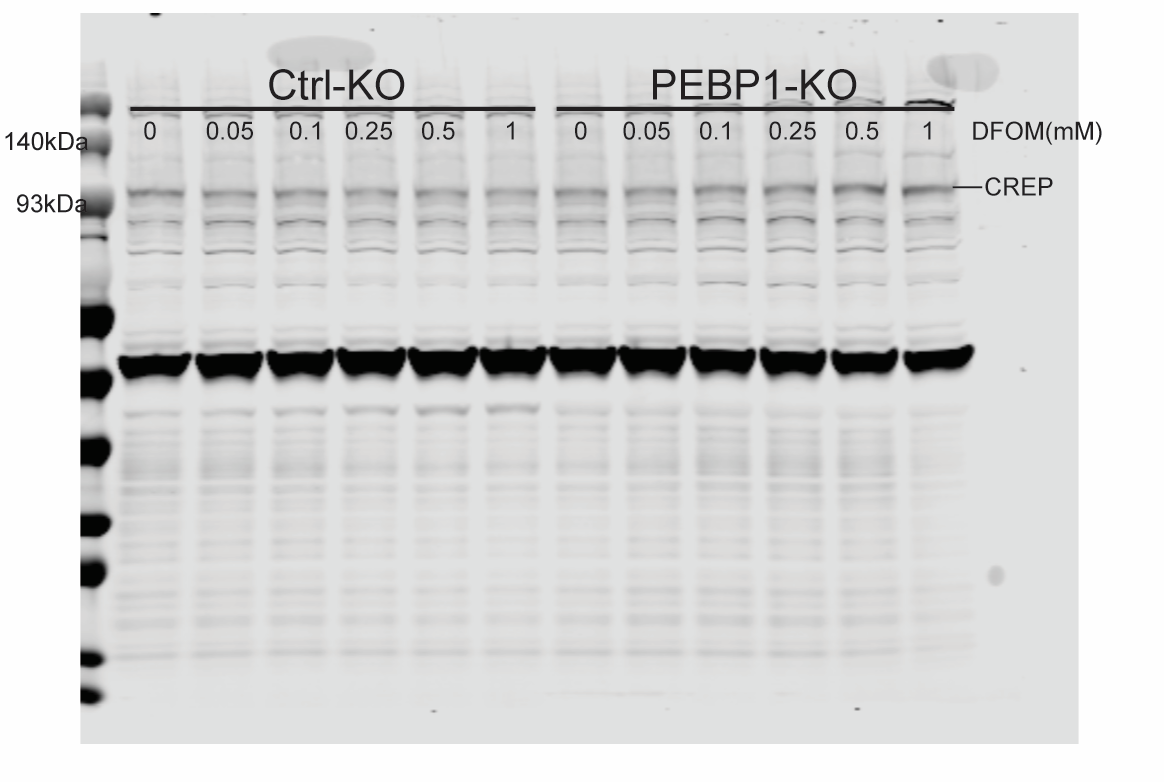

Supplement: Figure 3—figure supplement 2—source data 1. [file elife-102852-fig3-figsupp2-data1.zip › Figure 3-source data 3/Fig3Supplement2D_CREP_band_indicated.tif]

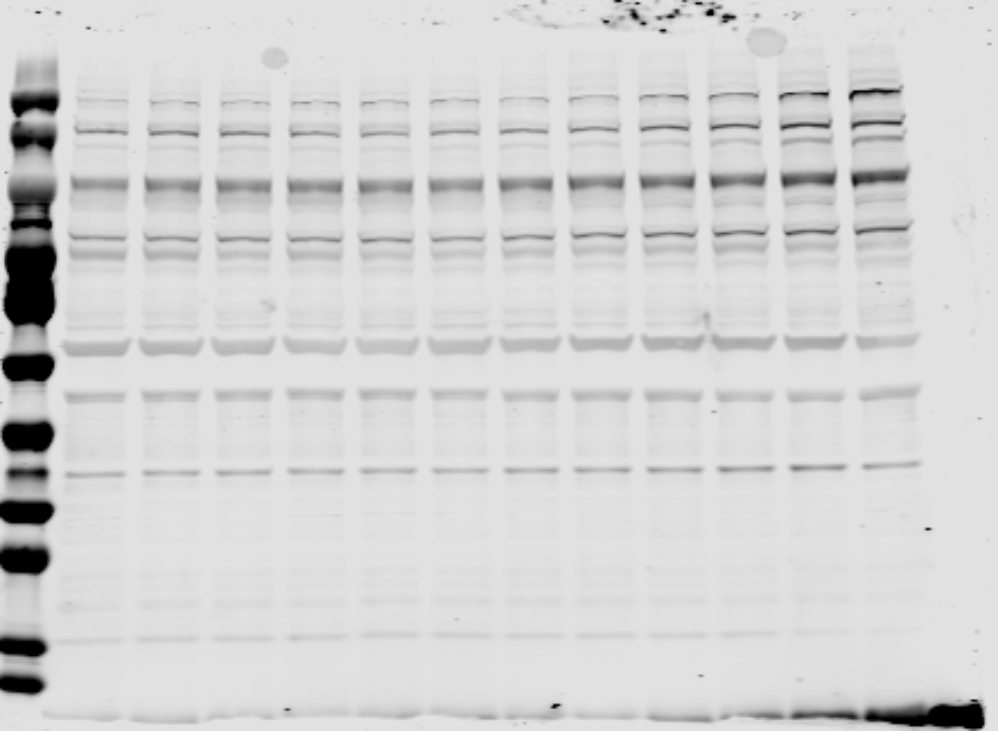

Supplement: Figure 3—figure supplement 2—source data 1. [file elife-102852-fig3-figsupp2-data1.zip › Figure 3-source data 3/Fig3Supplement2F_GADD34_original.tif]

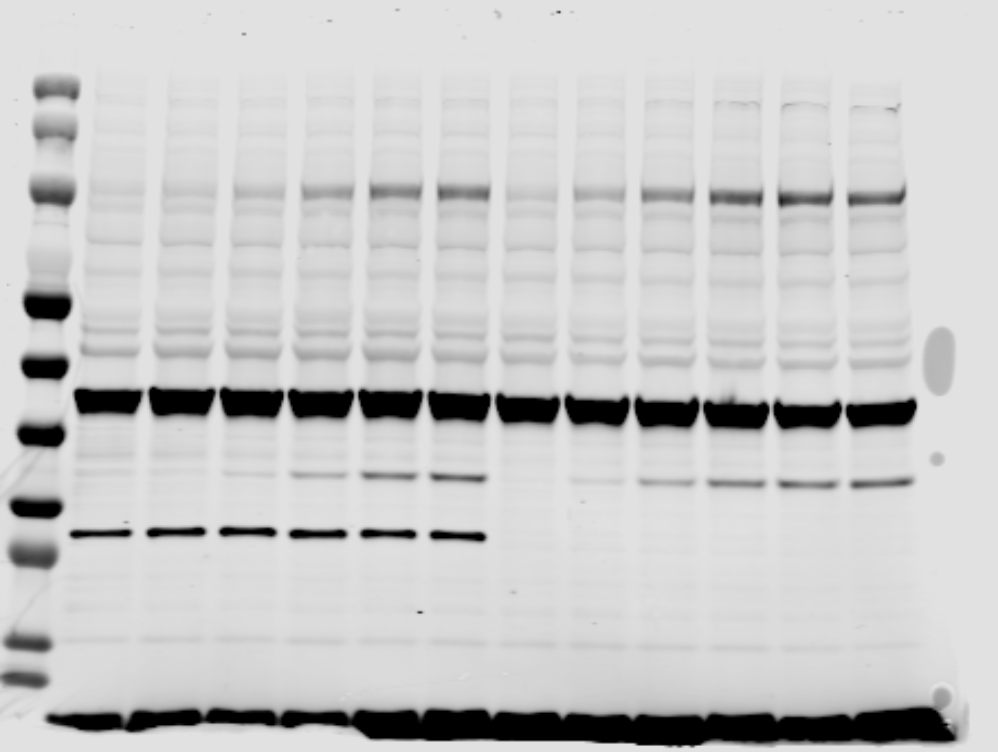

Supplement: Figure 3—figure supplement 2—source data 1. [file elife-102852-fig3-figsupp2-data1.zip › Figure 3-source data 3/Fig3Supplement2G_GADD34_original.tif]

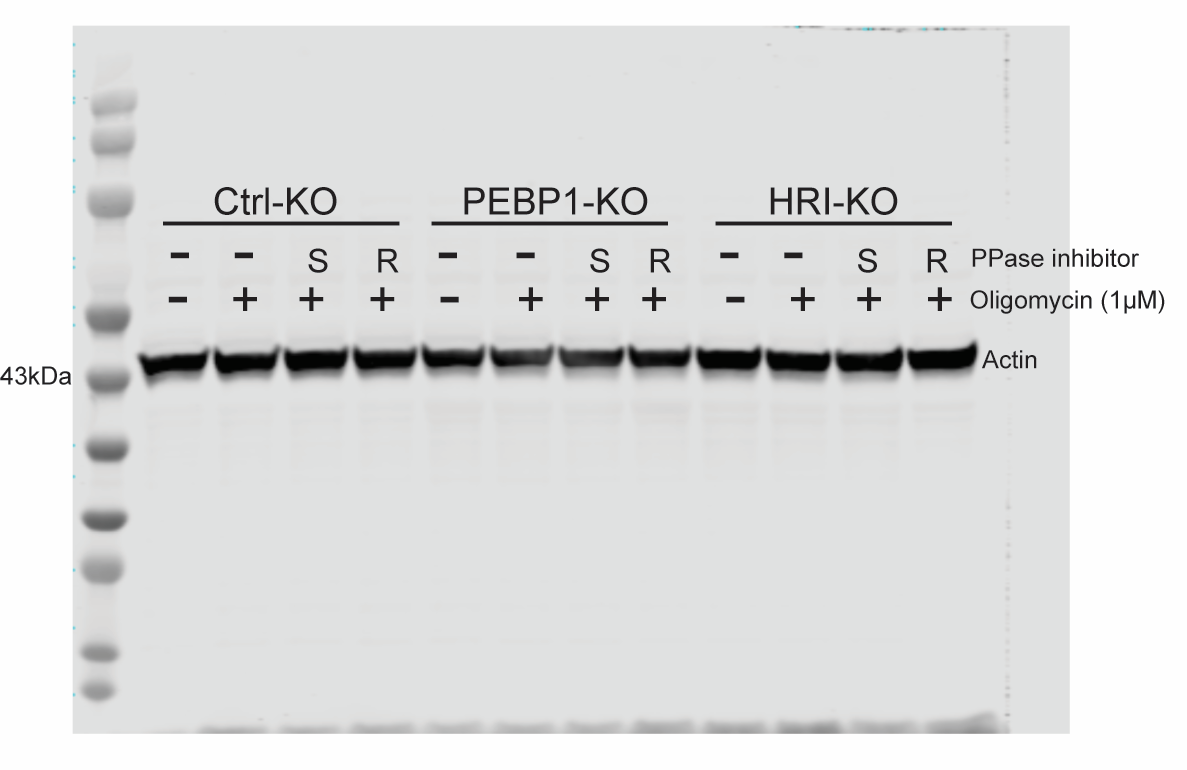

Supplement: Figure 3—figure supplement 2—source data 1. [file elife-102852-fig3-figsupp2-data1.zip › Figure 3-source data 3/Fig3Supplement2H_Actin_band_indicated.tif]

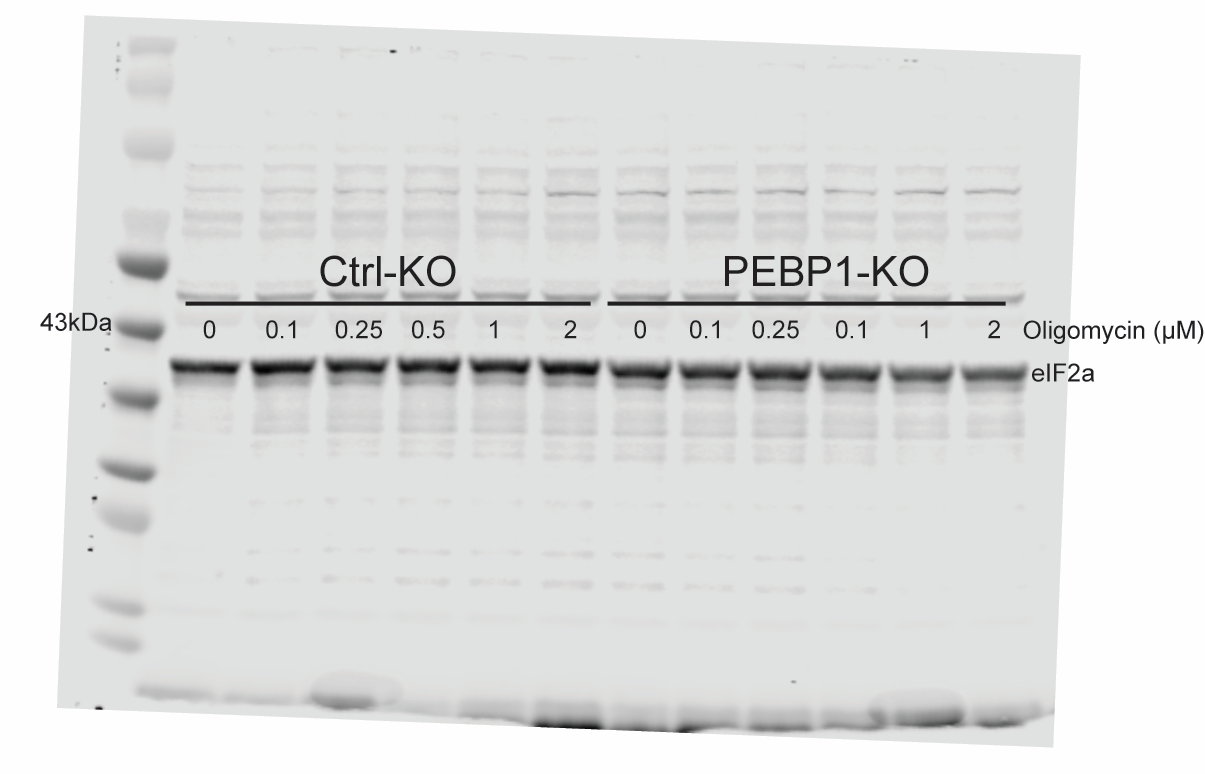

Supplement: Figure 3—figure supplement 2—source data 1. [file elife-102852-fig3-figsupp2-data1.zip › Figure 3-source data 3/Fig3Supplement2C_eIF2a_band_indicated.tif]

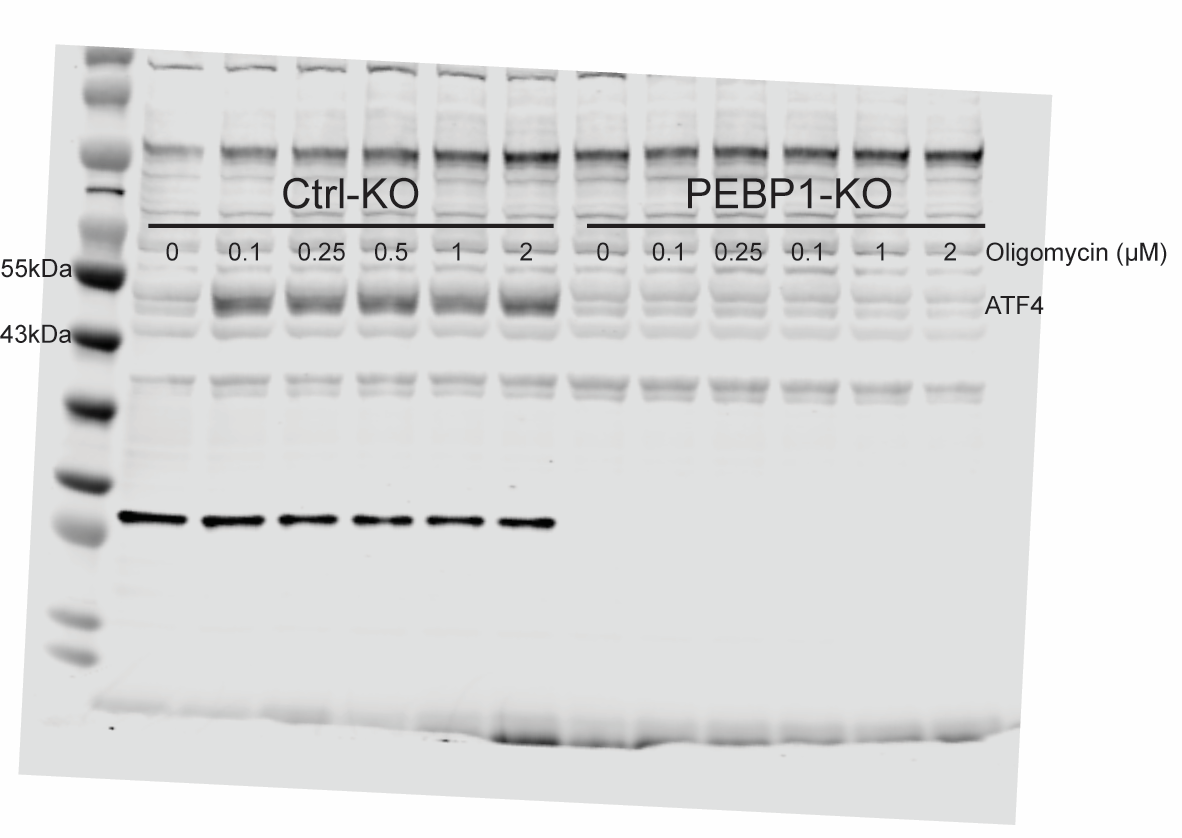

Supplement: Figure 3—figure supplement 2—source data 1. [file elife-102852-fig3-figsupp2-data1.zip › Figure 3-source data 3/Fig3Supplement2C_ATF4_band_indicated.tif]

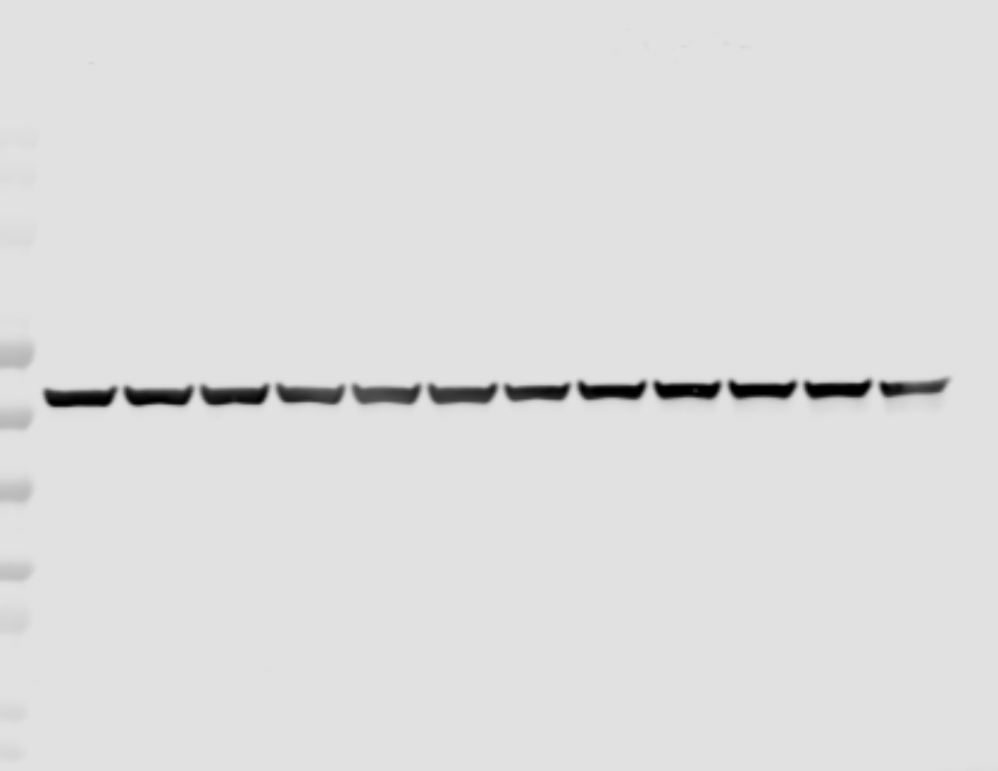

Supplement: Figure 3—figure supplement 2—source data 1. [file elife-102852-fig3-figsupp2-data1.zip › Figure 3-source data 3/Fig3Supplement2F_Actin_original.tif]

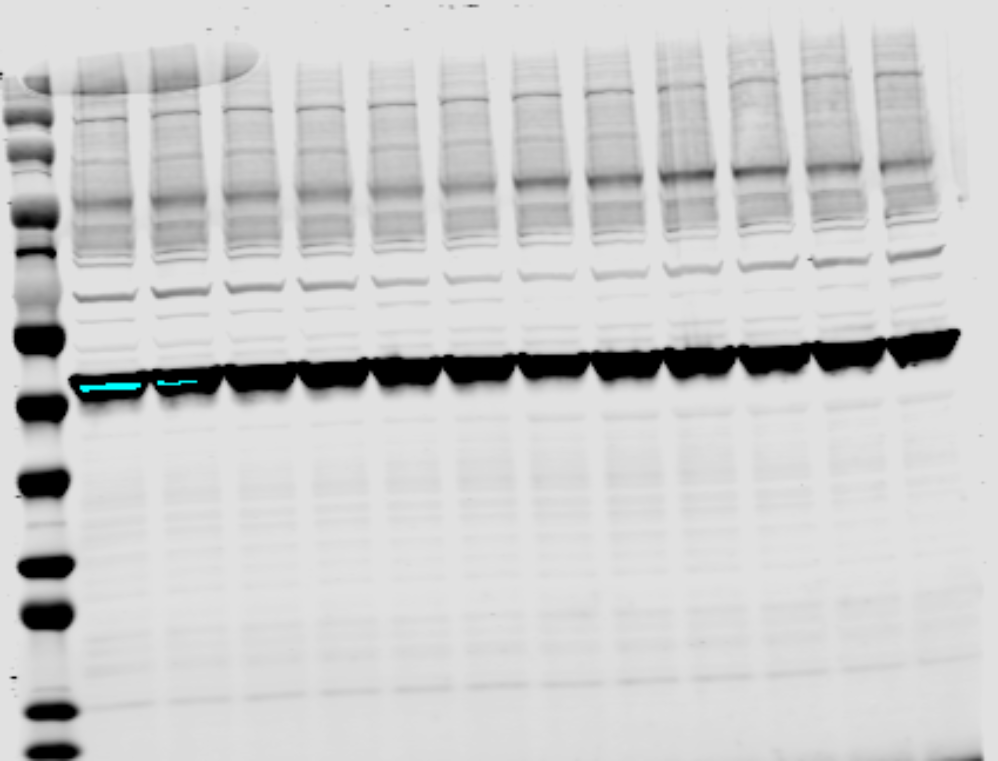

Supplement: Figure 3—figure supplement 2—source data 1. [file elife-102852-fig3-figsupp2-data1.zip › Figure 3-source data 3/Fig3Supplement2E_CREP_original.tif]

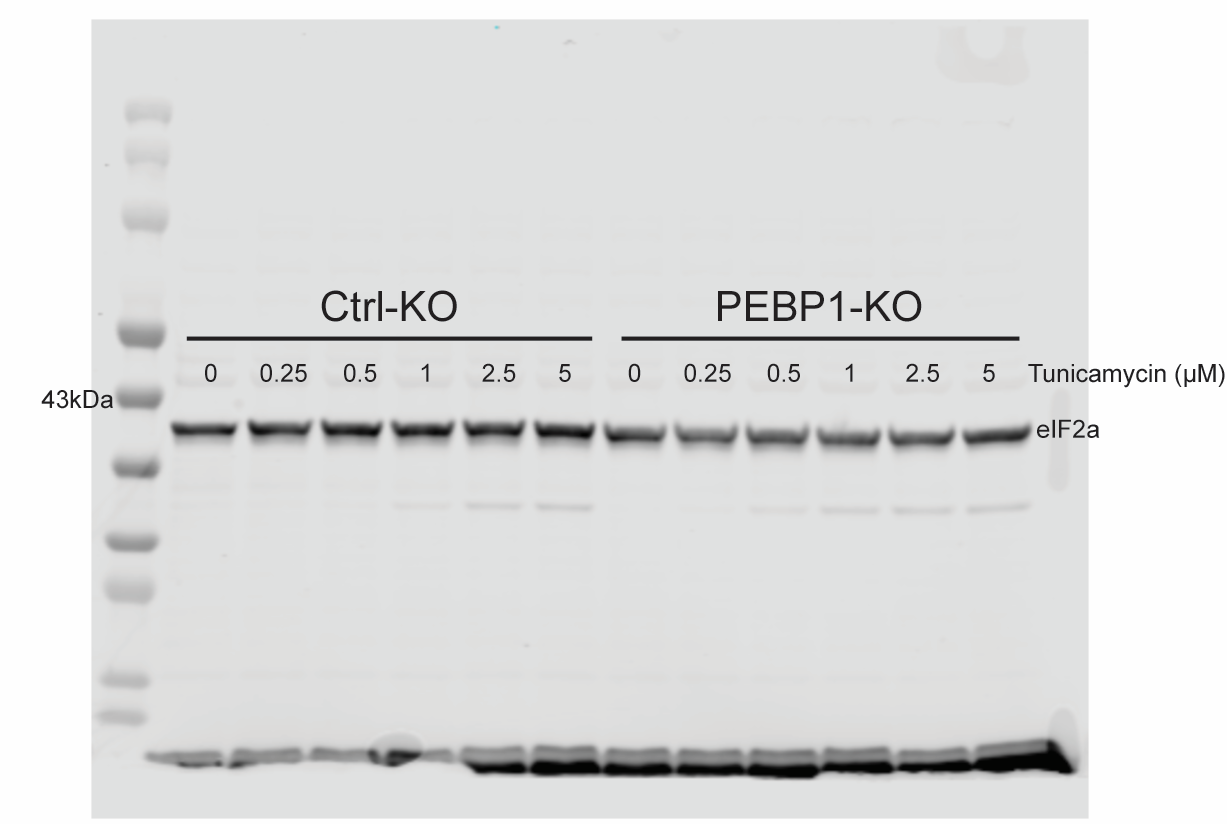

Supplement: Figure 3—figure supplement 2—source data 1. [file elife-102852-fig3-figsupp2-data1.zip › Figure 3-source data 3/Fig3Supplement2G_eIF2a_band_indicated.tif]

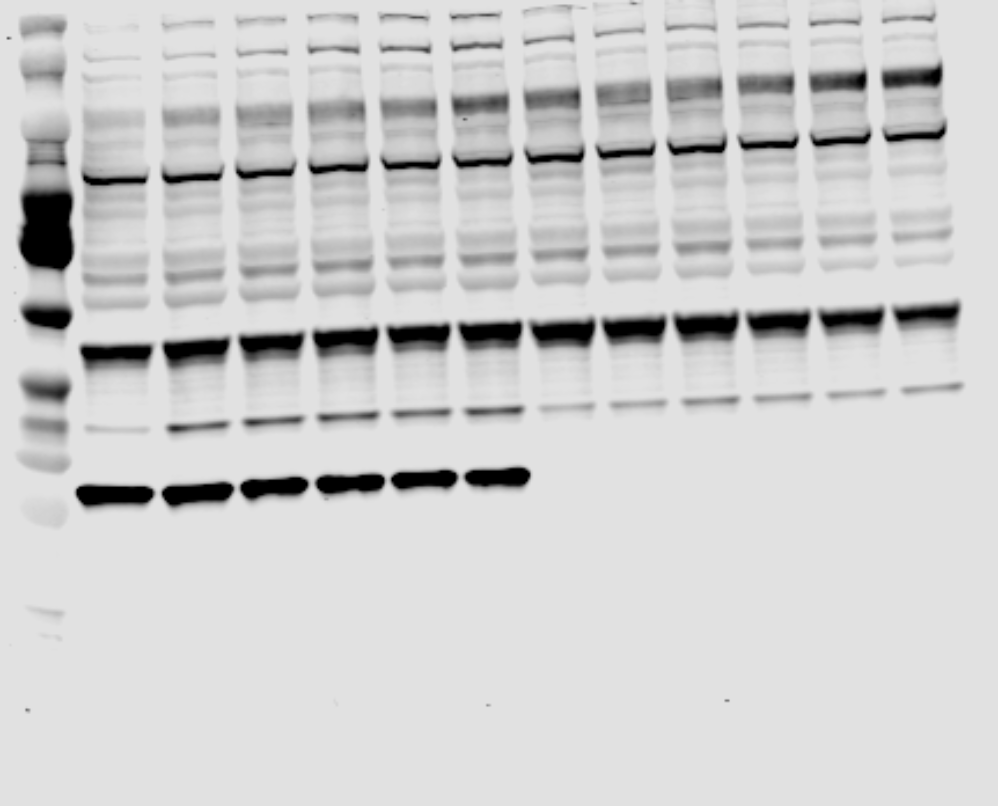

Supplement: Figure 3—figure supplement 2—source data 1. [file elife-102852-fig3-figsupp2-data1.zip › Figure 3-source data 3/Fig3Supplement2C_GADD34_original.tif]

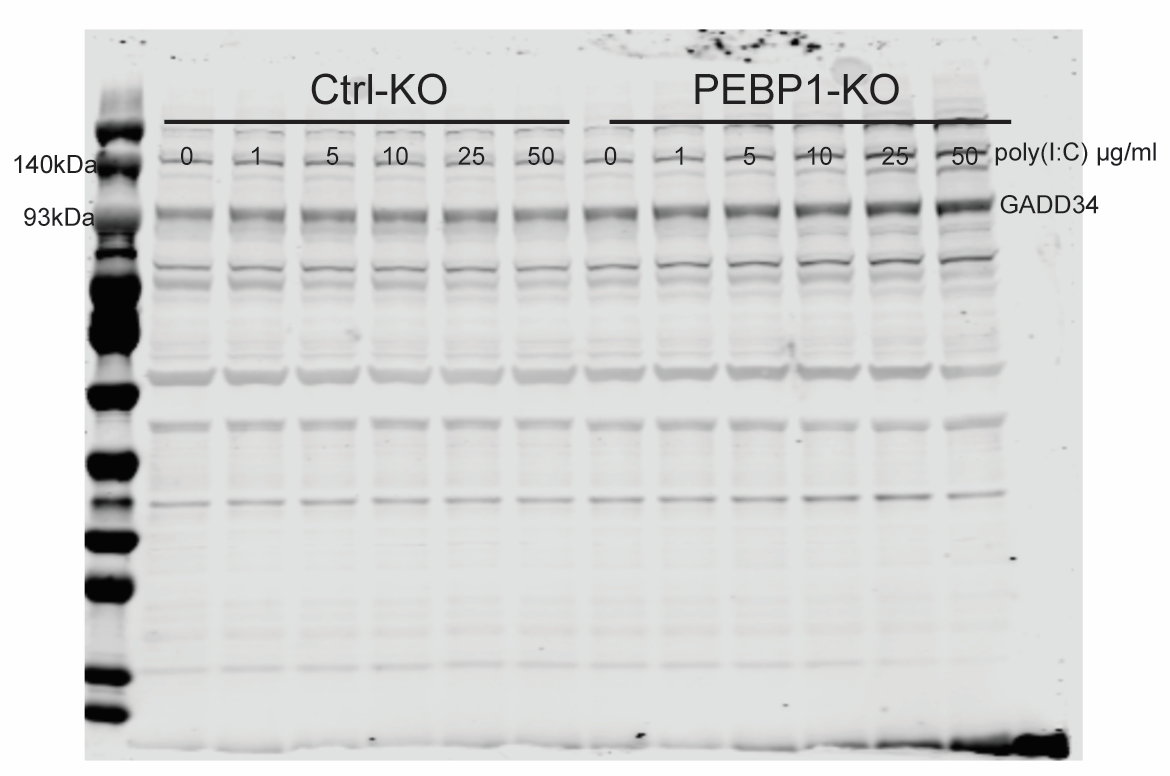

Supplement: Figure 3—figure supplement 2—source data 1. [file elife-102852-fig3-figsupp2-data1.zip › Figure 3-source data 3/Fig3Supplement2F_GADD34_band_indicated.tif]

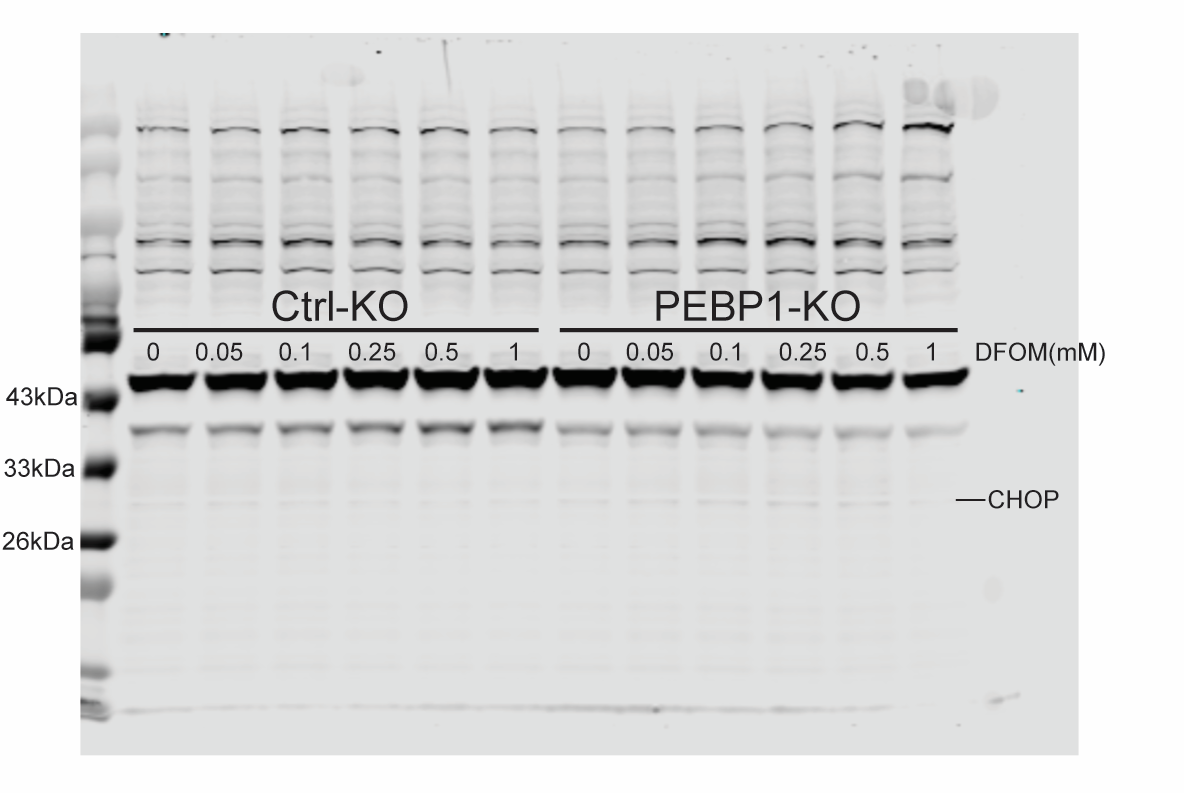

Supplement: Figure 3—figure supplement 2—source data 1. [file elife-102852-fig3-figsupp2-data1.zip › Figure 3-source data 3/Fig3Supplement2D_CHOP_band_indicated.tif]

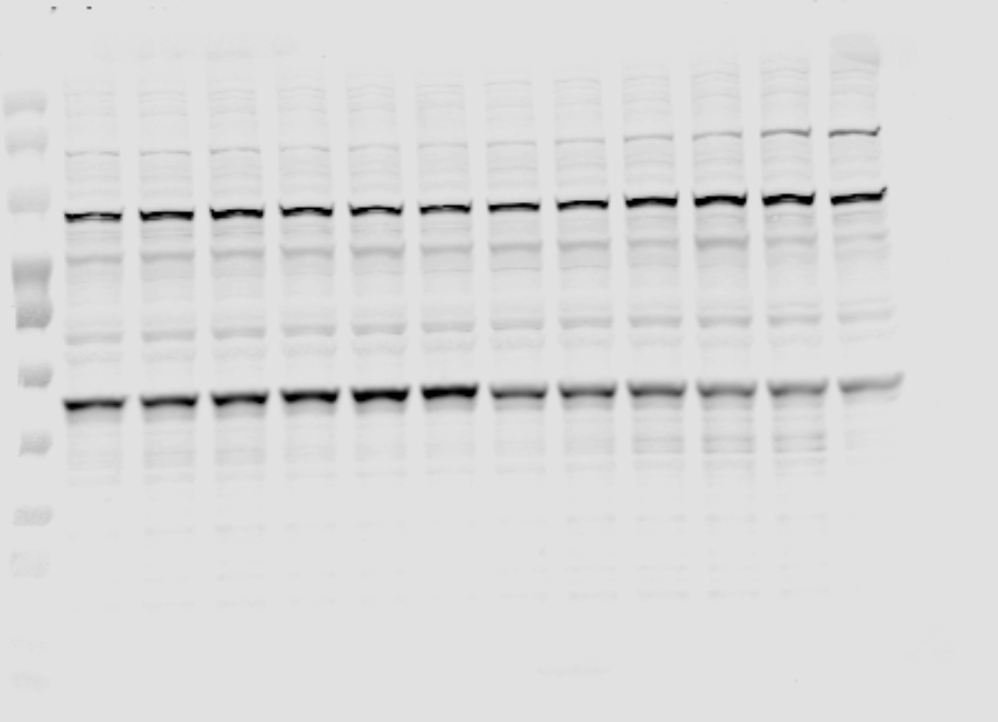

Supplement: Figure 3—figure supplement 2—source data 1. [file elife-102852-fig3-figsupp2-data1.zip › Figure 3-source data 3/Fig3Supplement2D_P-eIF2a_original.tif]

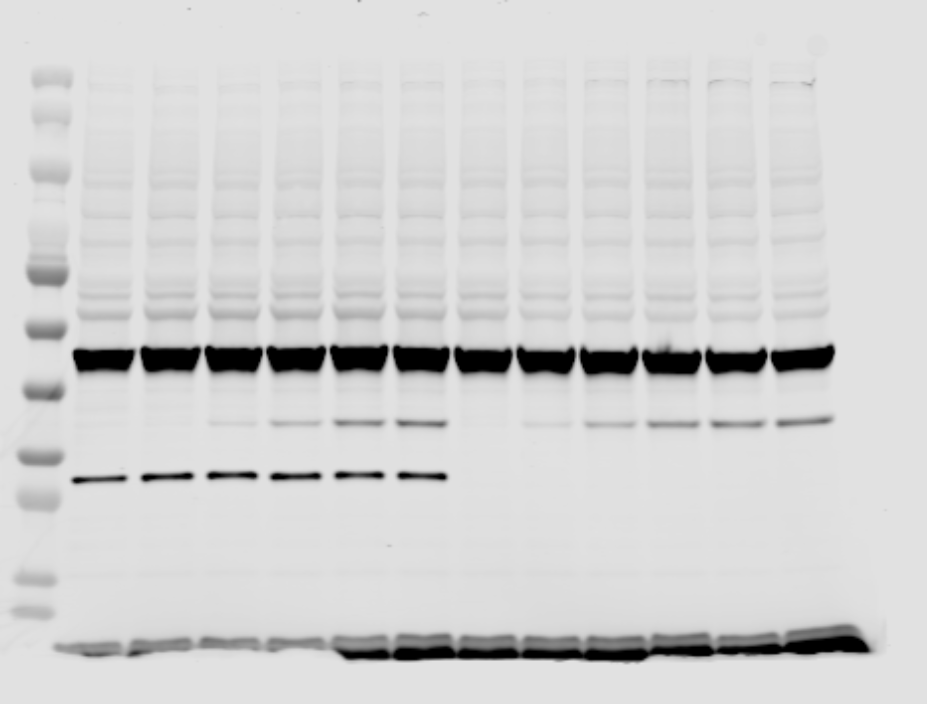

Supplement: Figure 3—figure supplement 2—source data 1. [file elife-102852-fig3-figsupp2-data1.zip › Figure 3-source data 3/Fig3Supplement2G_PEBP1_original.tif]
