# Supplementary figures and images for: PEBP1 amplifies mitochondrial dysfunction-induced integrated stress response (part 3 of 3)
Source: eLife. 2025 Jan 29;13:RP102852. doi: 10.7554/eLife.102852 (PMC11778924; doi:10.7554/eLife.102852)

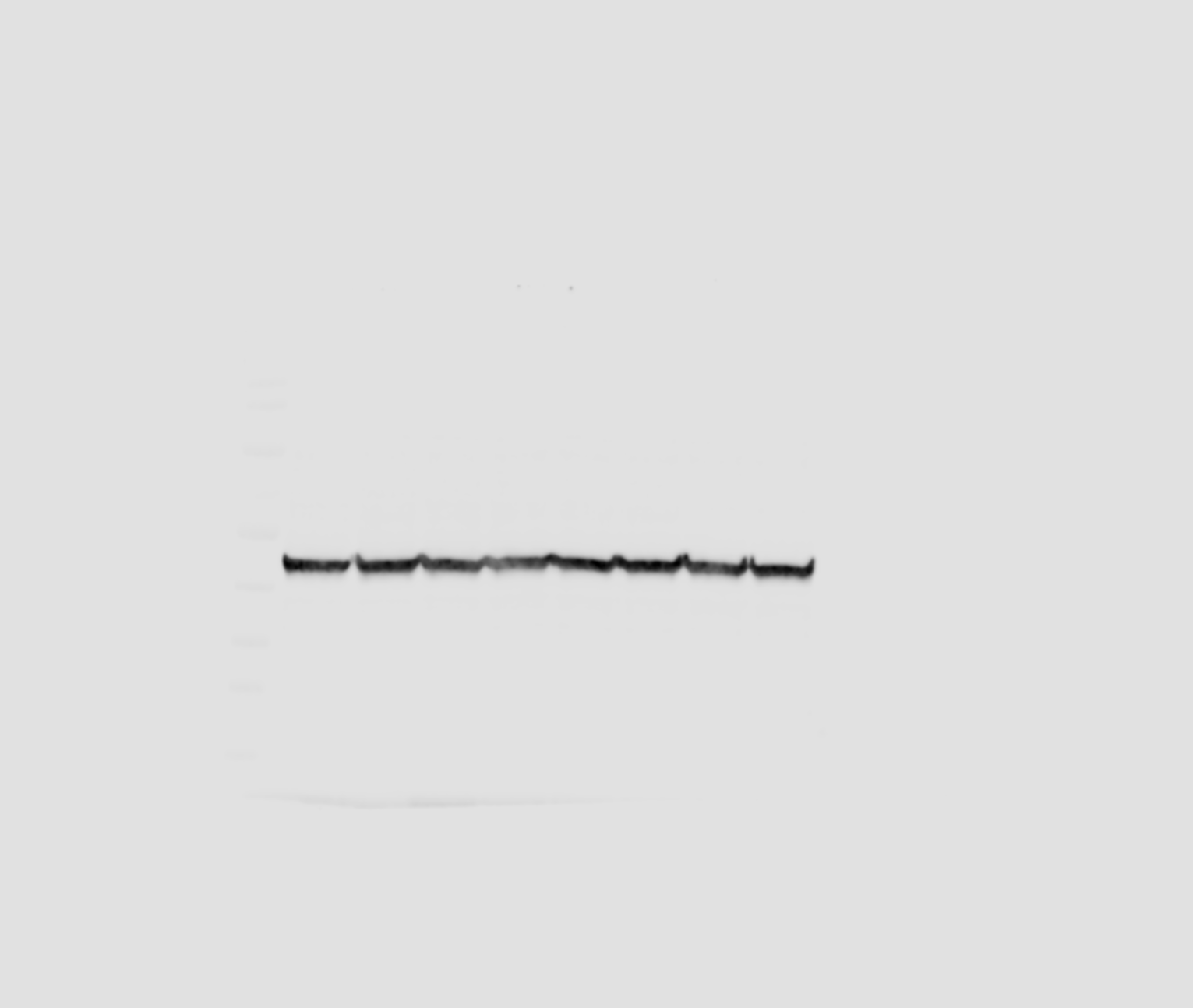

Supplement: Figure 4—source data 1. [file elife-102852-fig4-data1.zip › Figure 4-source data 1/Fig4F_Actin_original.tif]

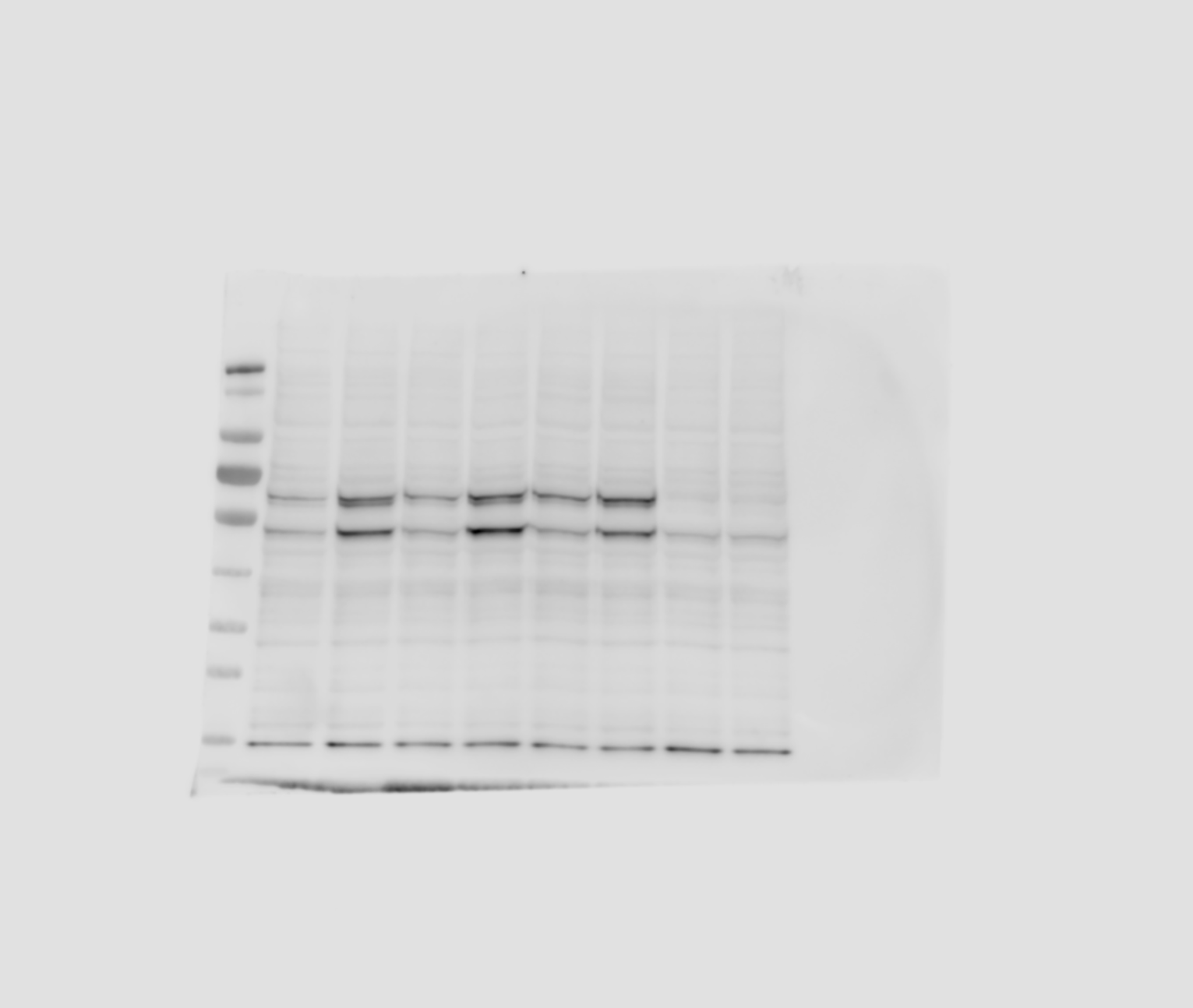

Supplement: Figure 4—source data 1. [file elife-102852-fig4-data1.zip › Figure 4-source data 1/Fig4F_DELE1 HA_original.tif]

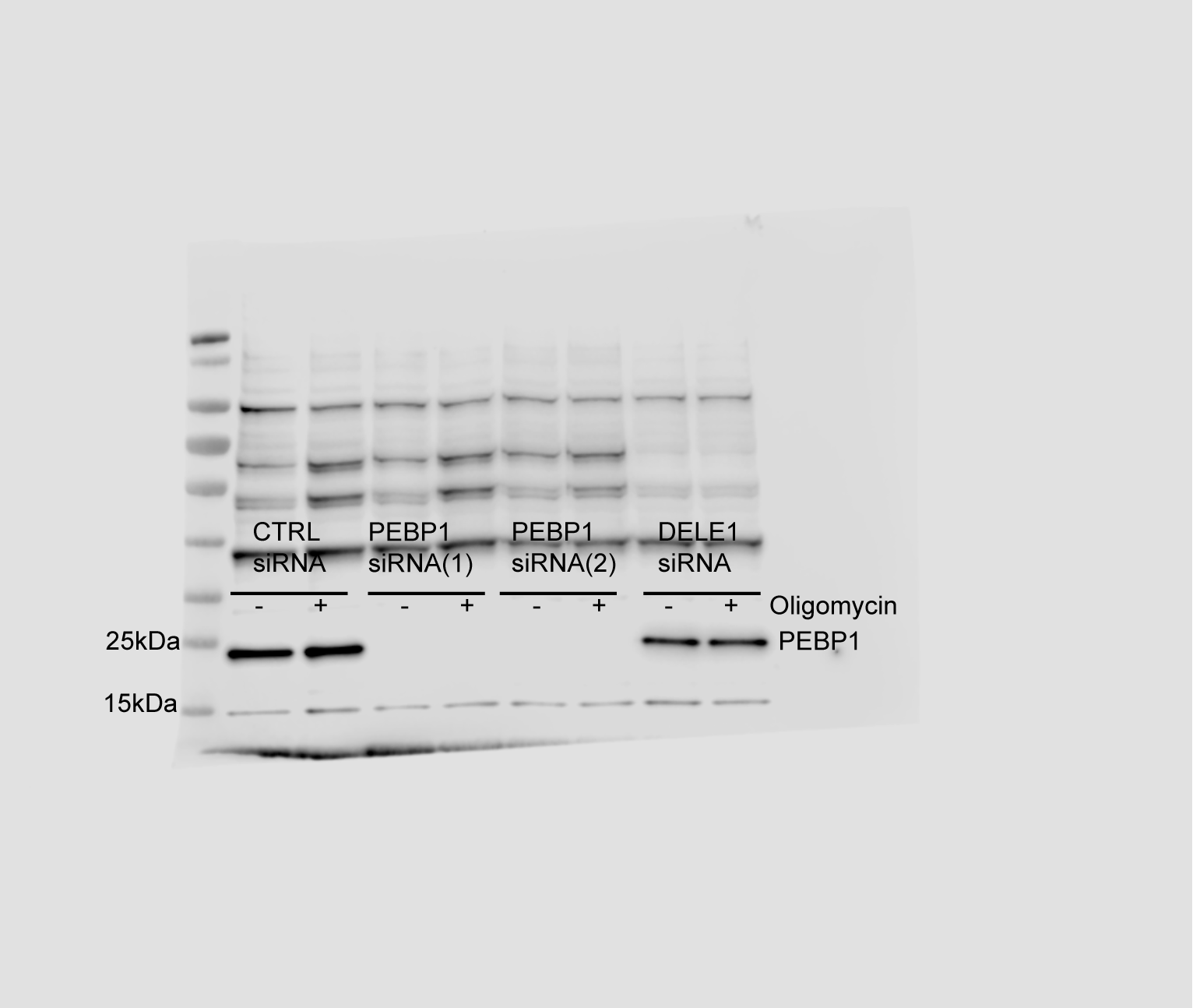

Supplement: Figure 4—source data 1. [file elife-102852-fig4-data1.zip › Figure 4-source data 1/Fig4F_PEBP1_band_indicated.tif]

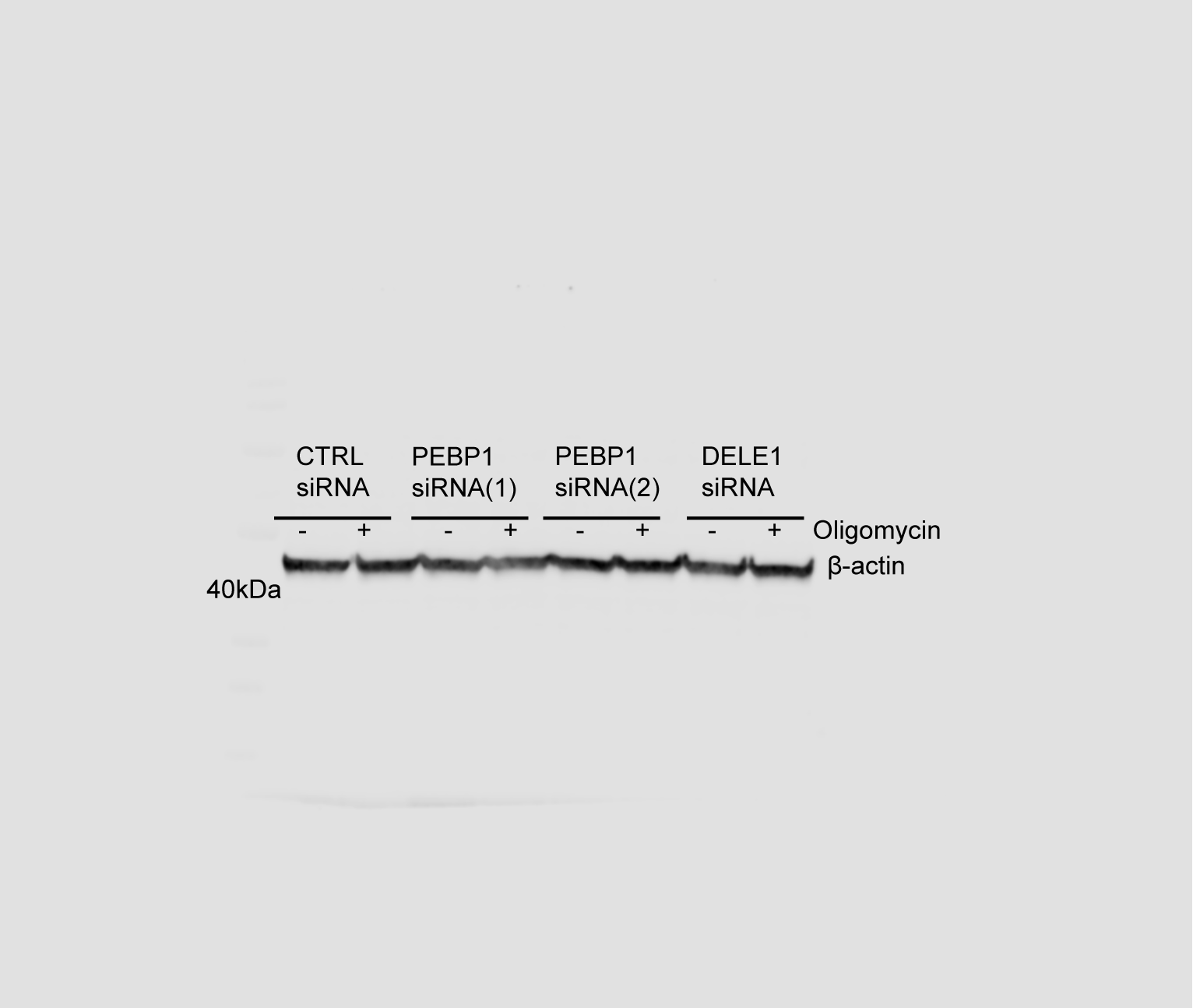

Supplement: Figure 4—source data 1. [file elife-102852-fig4-data1.zip › Figure 4-source data 1/Fig4F_Actin_band_indicated.tif]

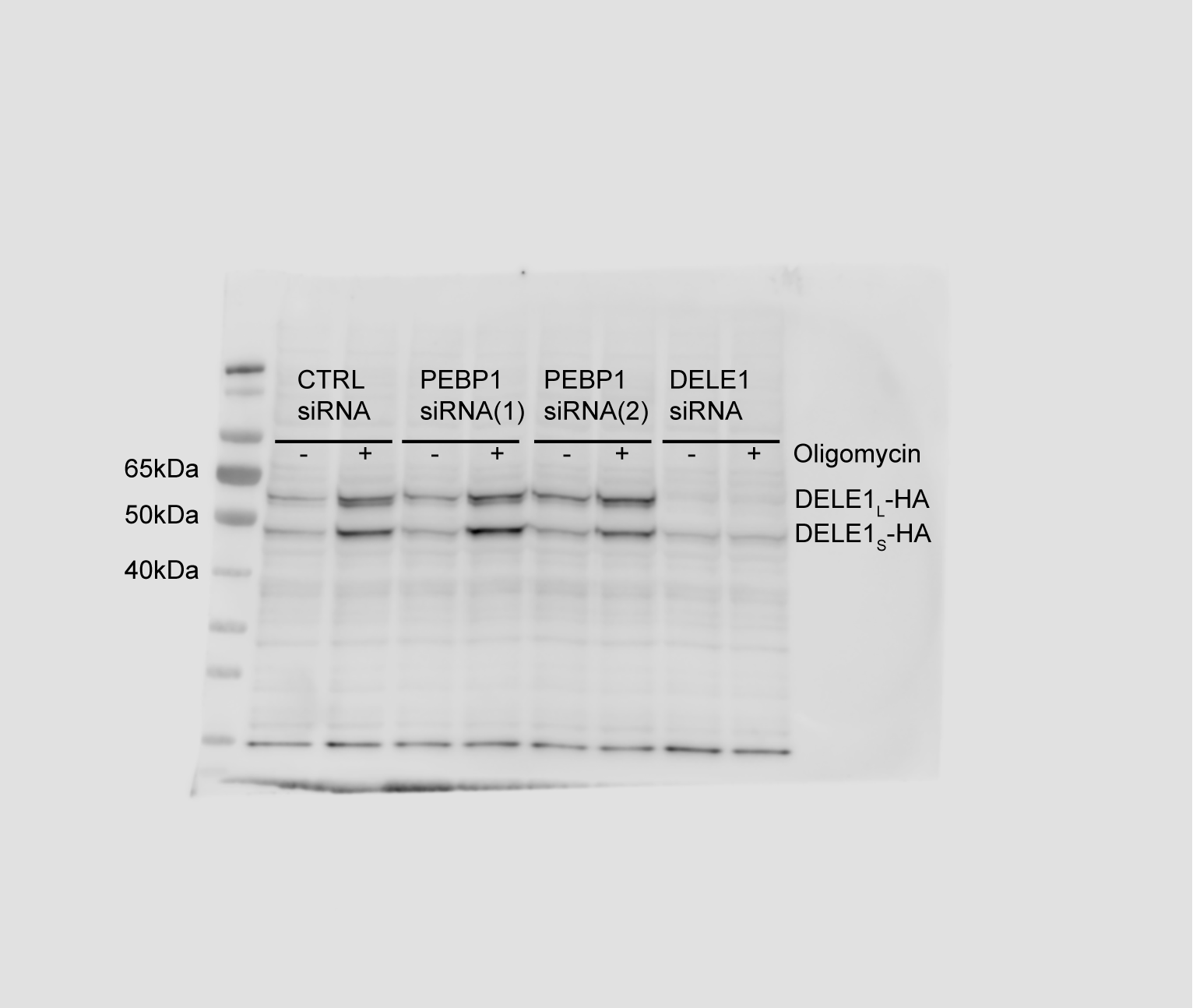

Supplement: Figure 4—source data 1. [file elife-102852-fig4-data1.zip › Figure 4-source data 1/Fig4F_DELE1 HA_band_indicated.tif]

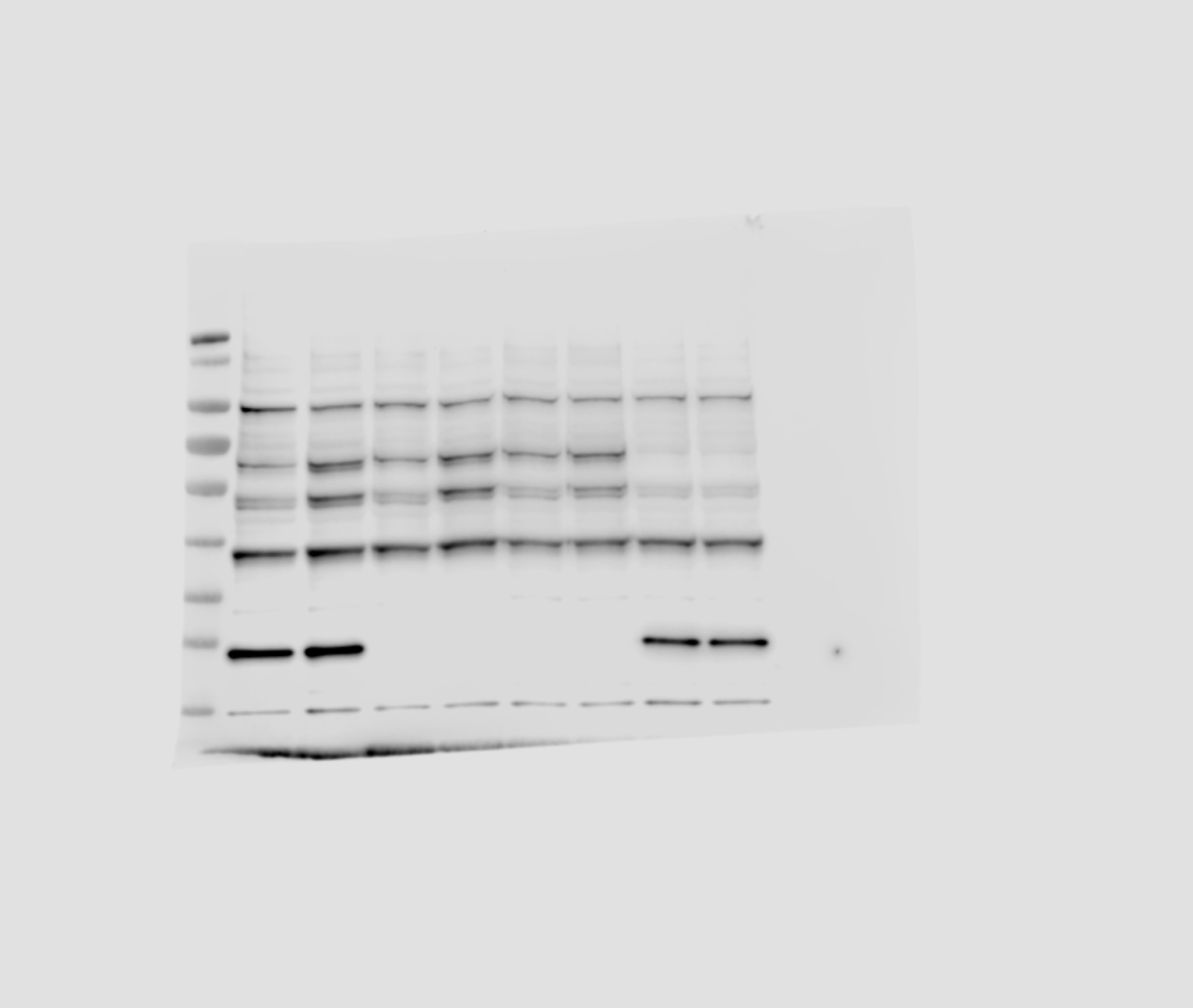

Supplement: Figure 4—source data 1. [file elife-102852-fig4-data1.zip › Figure 4-source data 1/Fig4F_PEBP1_original.tif]

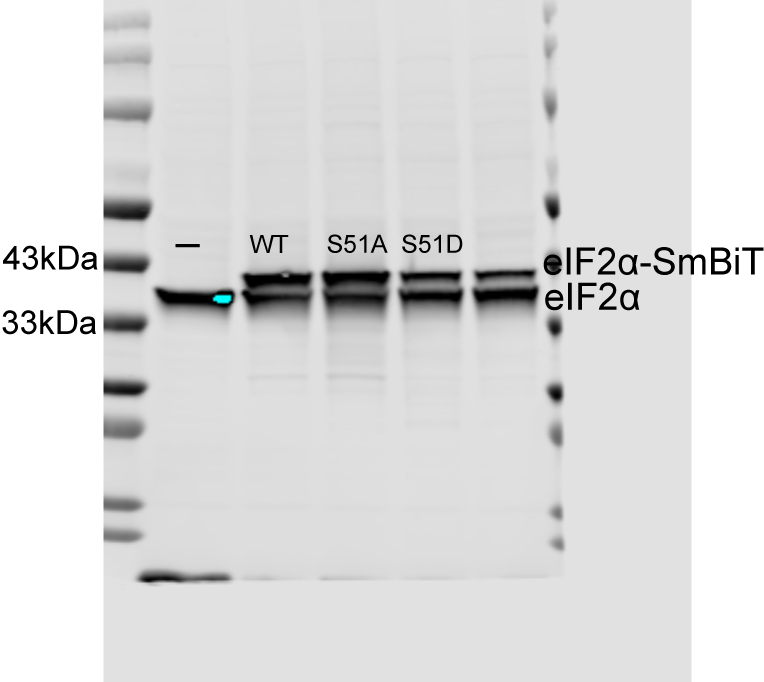

Supplement: Figure 5—source data 1. [file elife-102852-fig5-data1.zip › Figure 5-source data 1/Fig5E_eIF2a_band_indicated.tif]

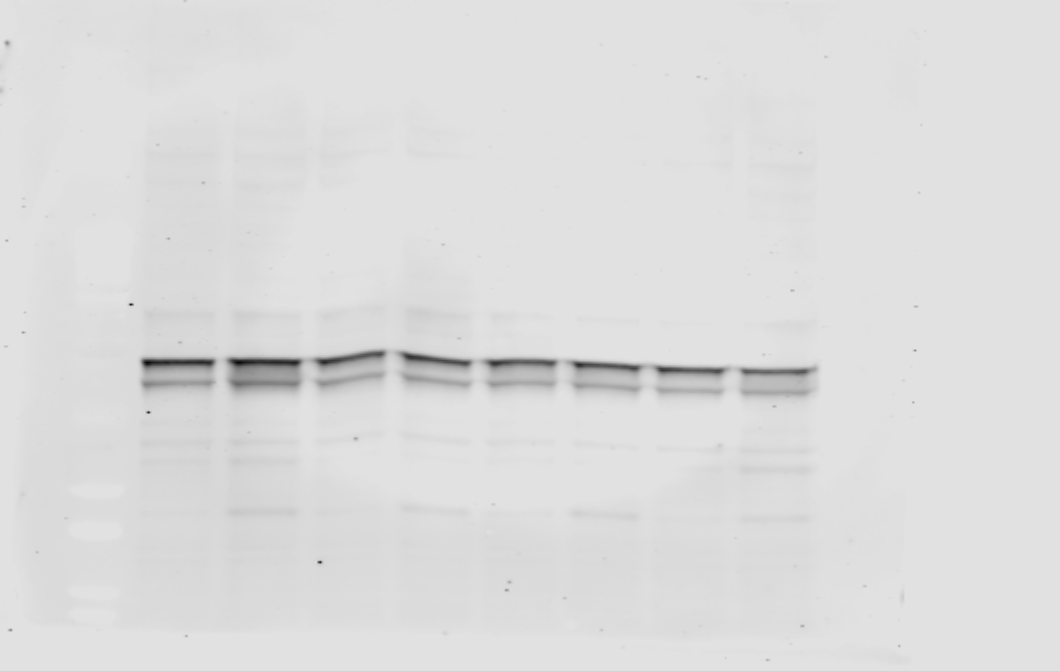

Supplement: Figure 5—source data 1. [file elife-102852-fig5-data1.zip › Figure 5-source data 1/Fig5B_eIF2a_original.tif]

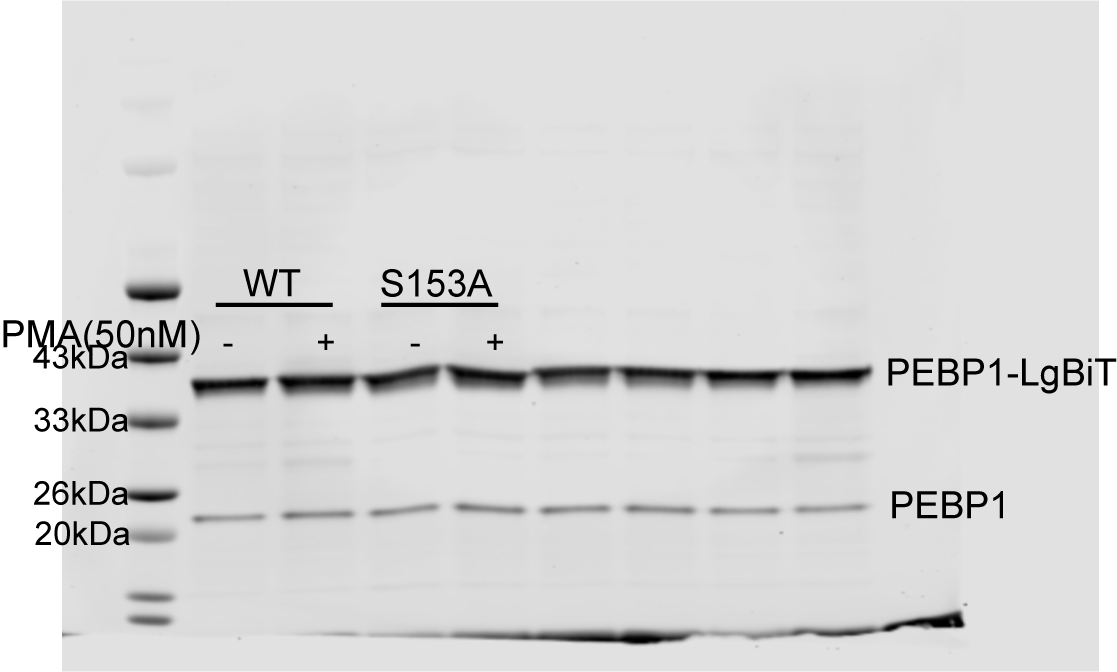

Supplement: Figure 5—source data 1. [file elife-102852-fig5-data1.zip › Figure 5-source data 1/Fig5B_PEBP1_band_indicated.tif]

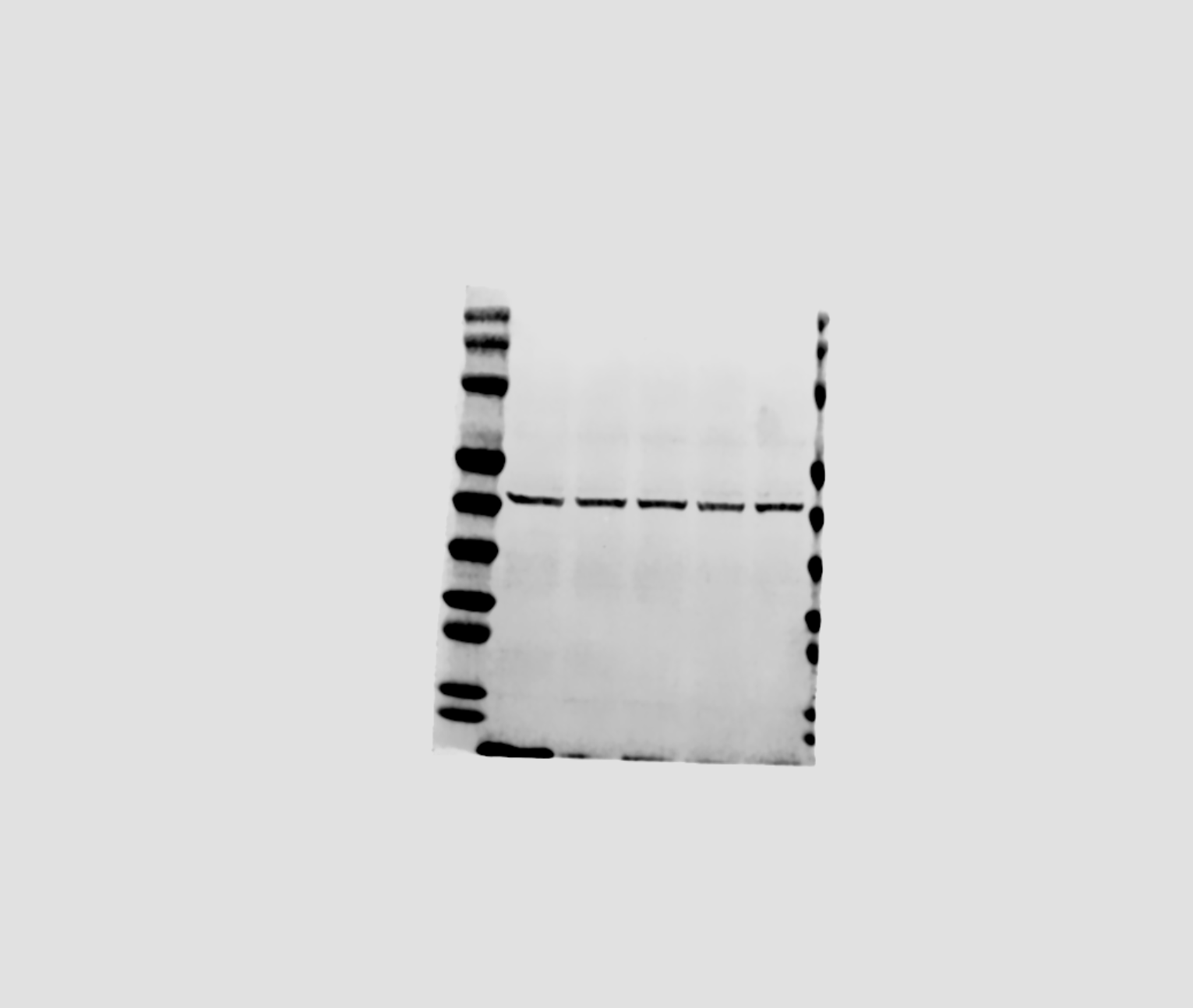

Supplement: Figure 5—source data 1. [file elife-102852-fig5-data1.zip › Figure 5-source data 1/Fig5E_Actin_original.tif]

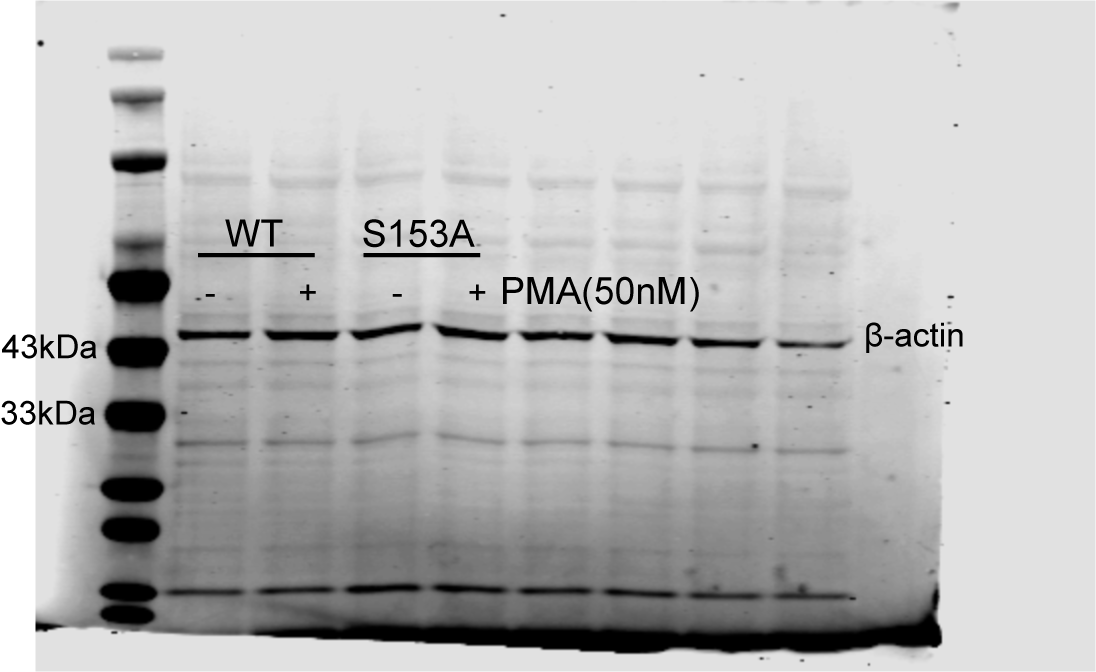

Supplement: Figure 5—source data 1. [file elife-102852-fig5-data1.zip › Figure 5-source data 1/Fig5B_Actin_band_indicated.tif]

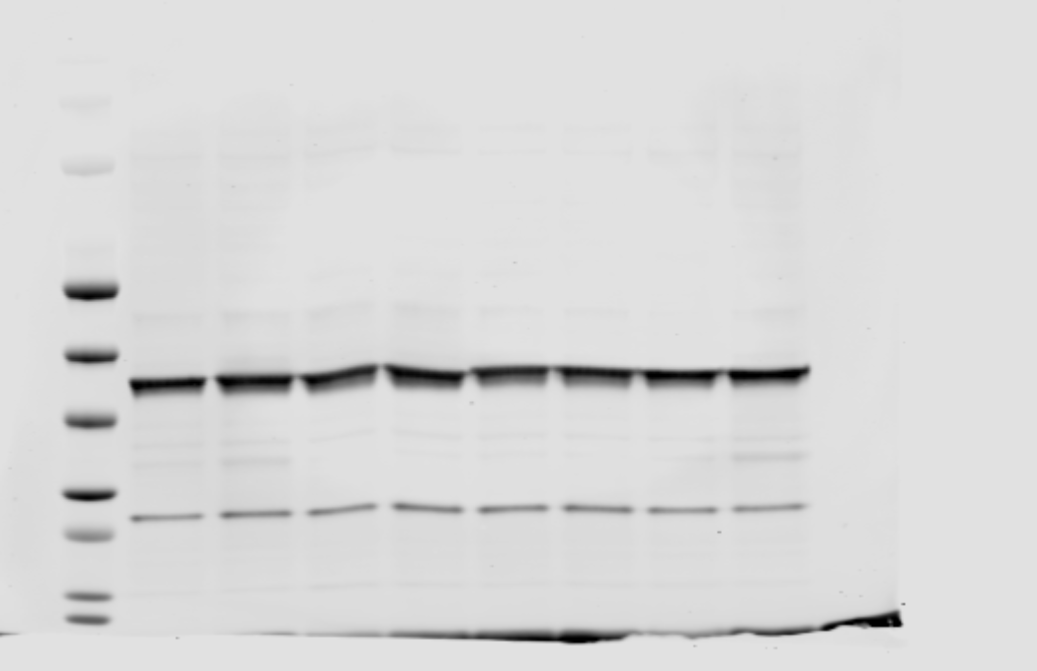

Supplement: Figure 5—source data 1. [file elife-102852-fig5-data1.zip › Figure 5-source data 1/Fig5B_PEBP1_original.tif]

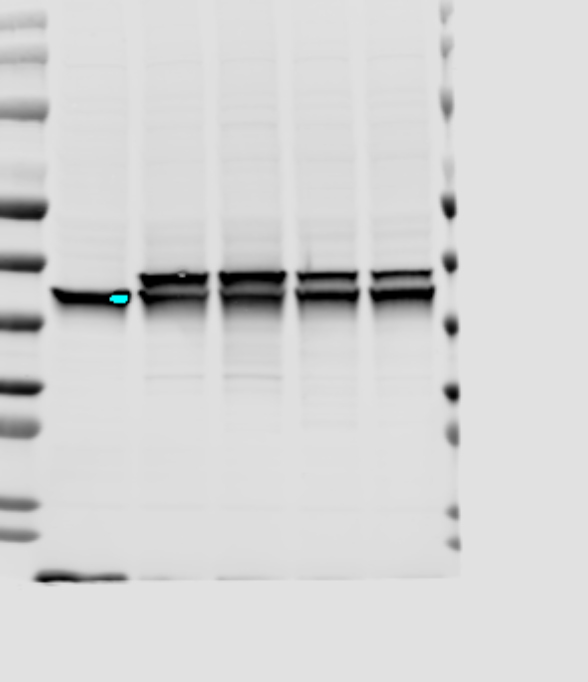

Supplement: Figure 5—source data 1. [file elife-102852-fig5-data1.zip › Figure 5-source data 1/Fig5E_eIF2a_original.tif]

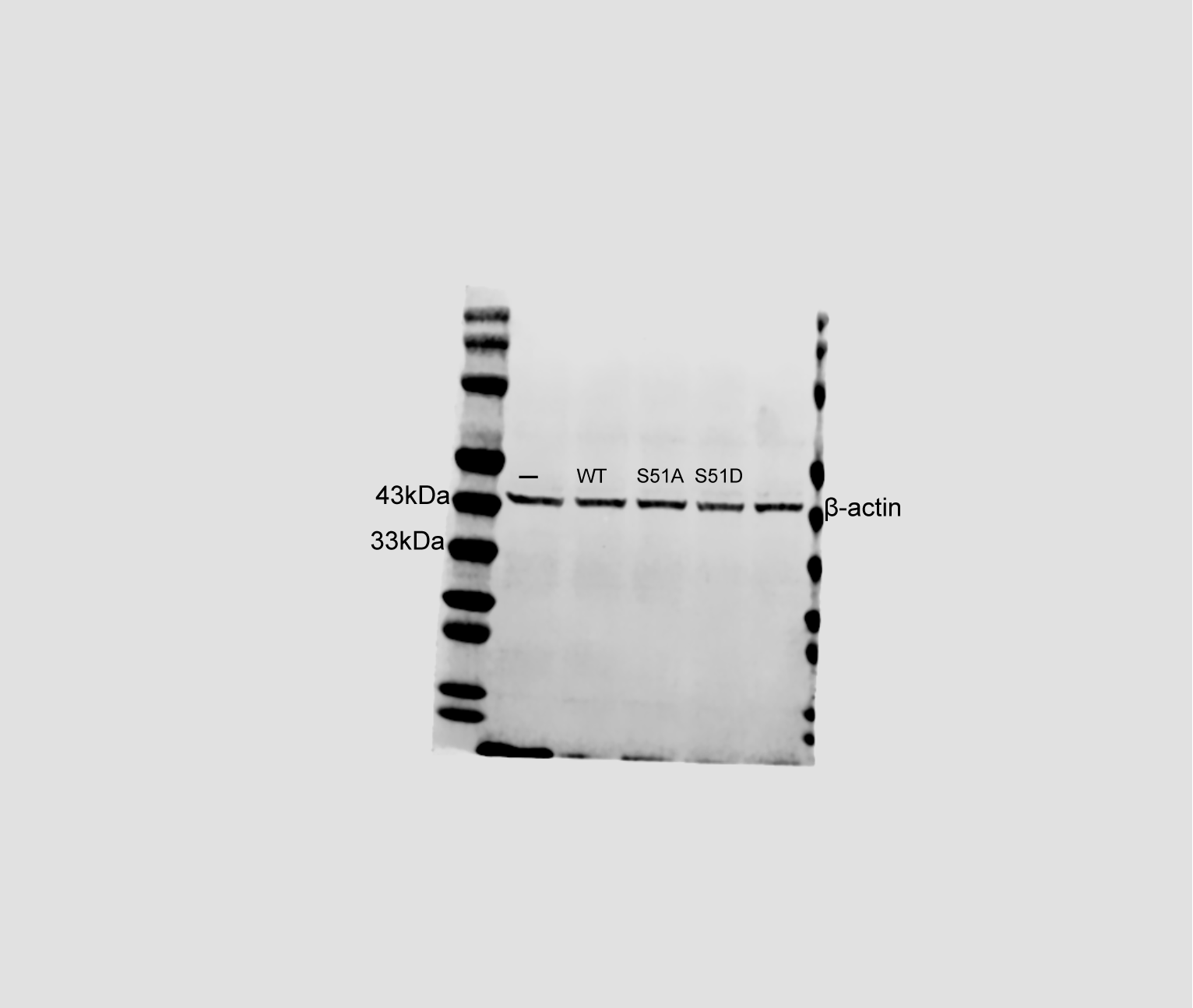

Supplement: Figure 5—source data 1. [file elife-102852-fig5-data1.zip › Figure 5-source data 1/Fig5E_Actin_band_indicated.tif]

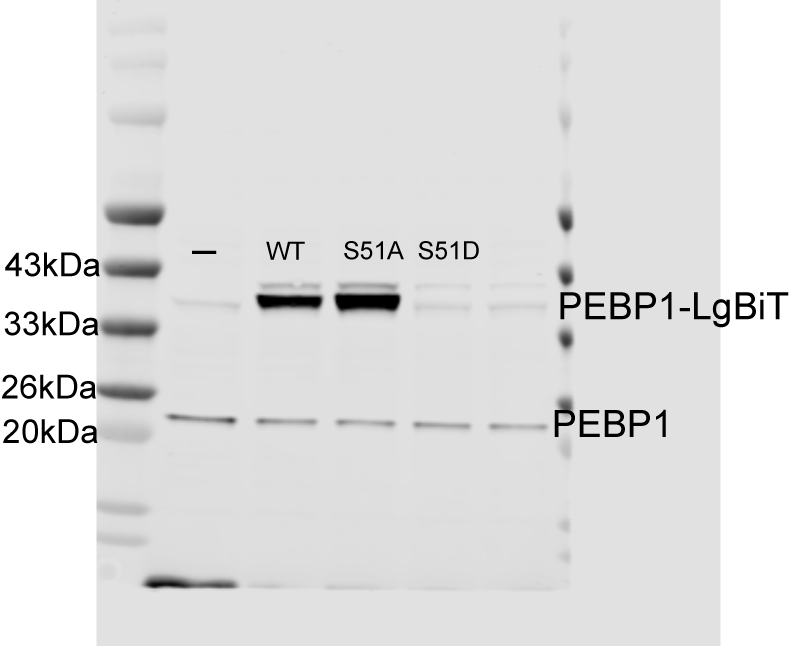

Supplement: Figure 5—source data 1. [file elife-102852-fig5-data1.zip › Figure 5-source data 1/Fig5E_PEBP1_band_indicated.tif]

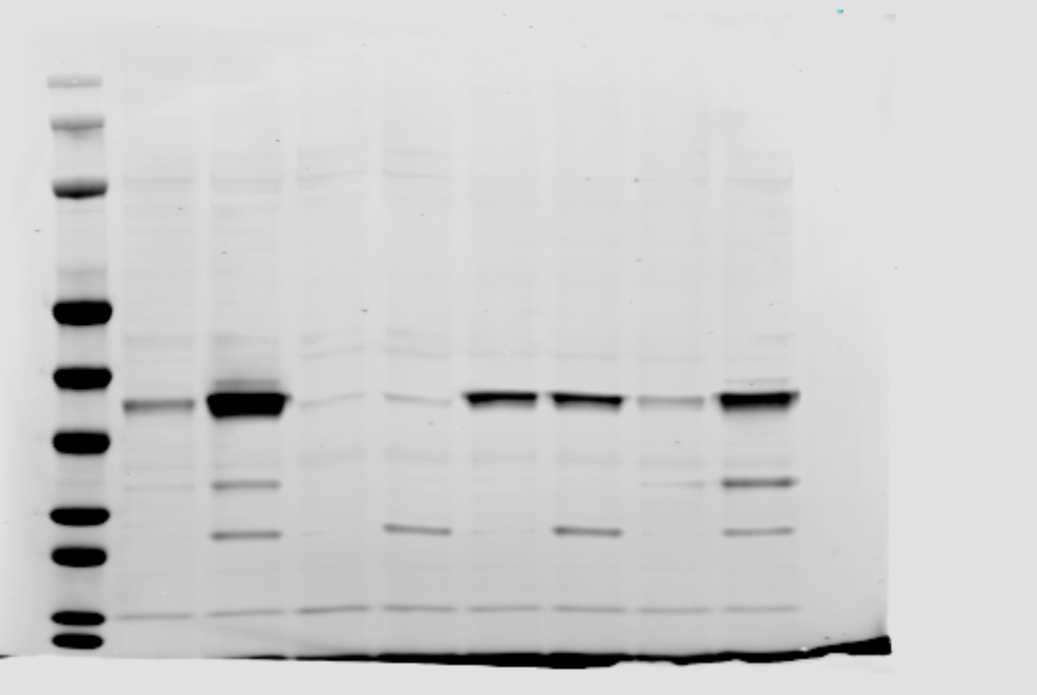

Supplement: Figure 5—source data 1. [file elife-102852-fig5-data1.zip › Figure 5-source data 1/Fig5B_P-PEBP1_original.tif]

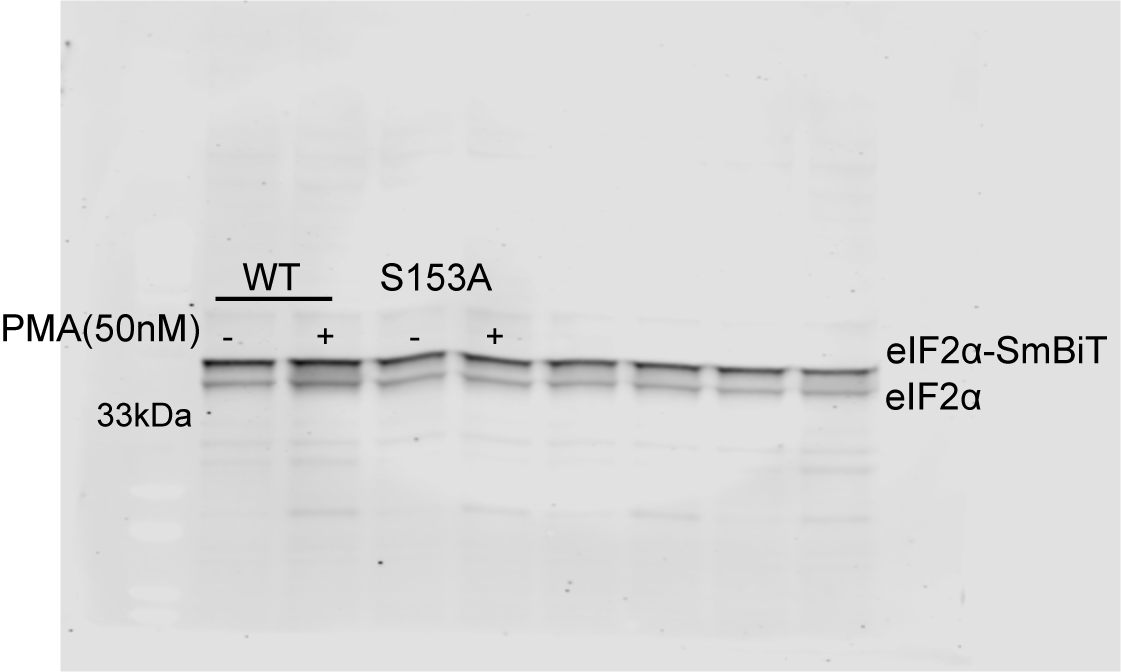

Supplement: Figure 5—source data 1. [file elife-102852-fig5-data1.zip › Figure 5-source data 1/Fig5B_eIF2a_band_indicated.tif]

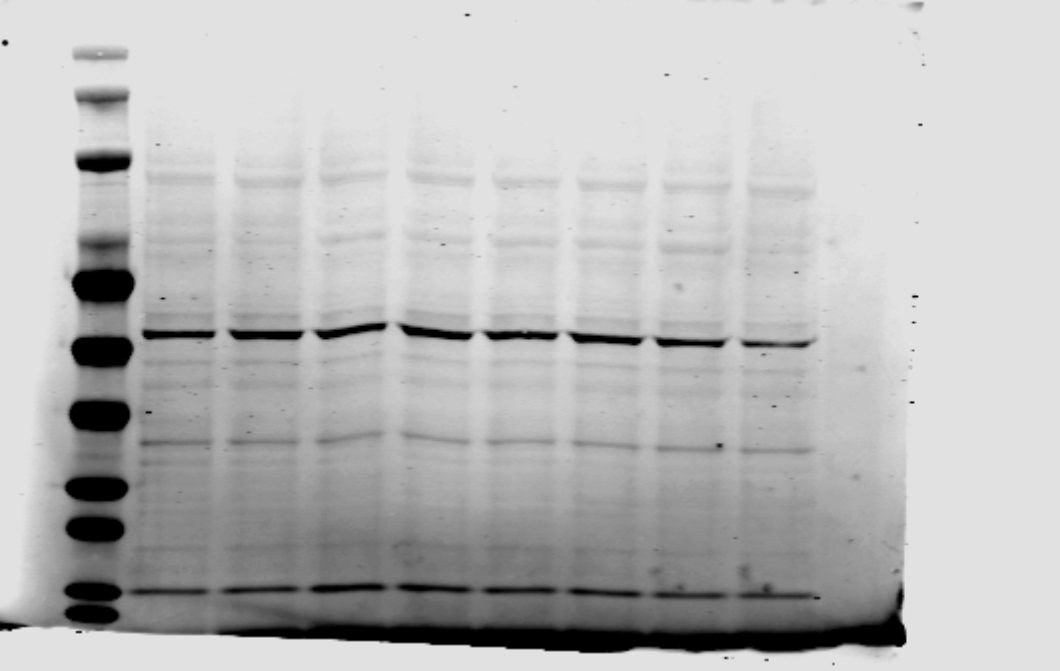

Supplement: Figure 5—source data 1. [file elife-102852-fig5-data1.zip › Figure 5-source data 1/Fig5B_Actin_original.tif]

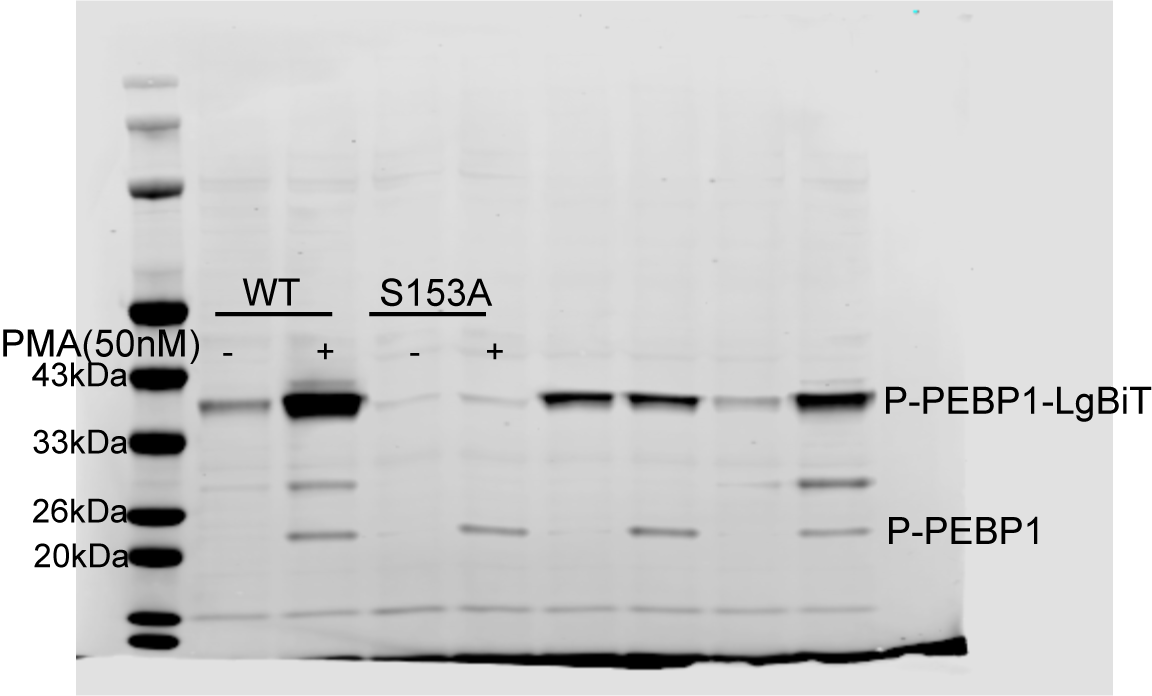

Supplement: Figure 5—source data 1. [file elife-102852-fig5-data1.zip › Figure 5-source data 1/Fig5B_P-PEBP1_band_indicated.tif]

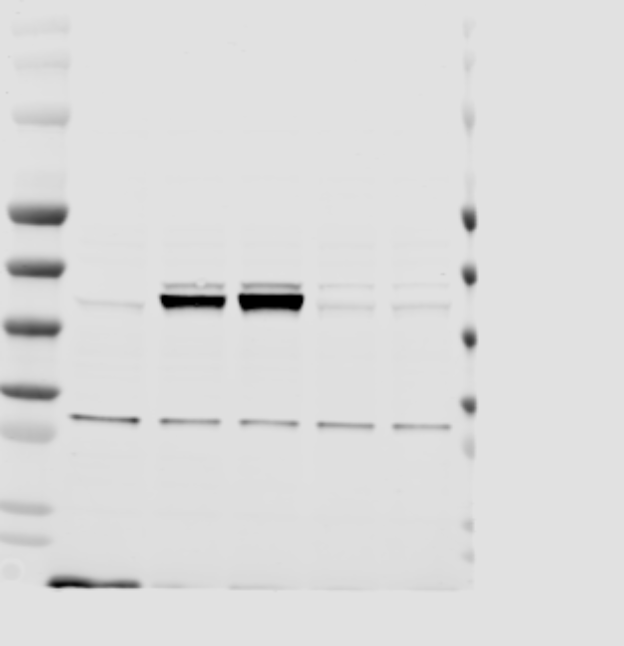

Supplement: Figure 5—source data 1. [file elife-102852-fig5-data1.zip › Figure 5-source data 1/Fig5E_PEBP1_original.tif]

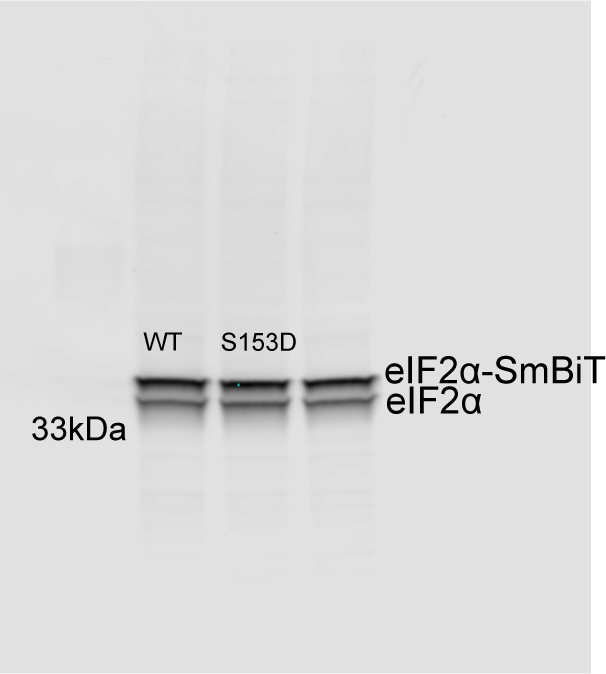

Supplement: Figure 5—figure supplement 1—source data 1. [file elife-102852-fig5-figsupp1-data1.zip › Figure 5-source data 2/Fig5Supplement1B_eIF2a_band_indicated.tif]

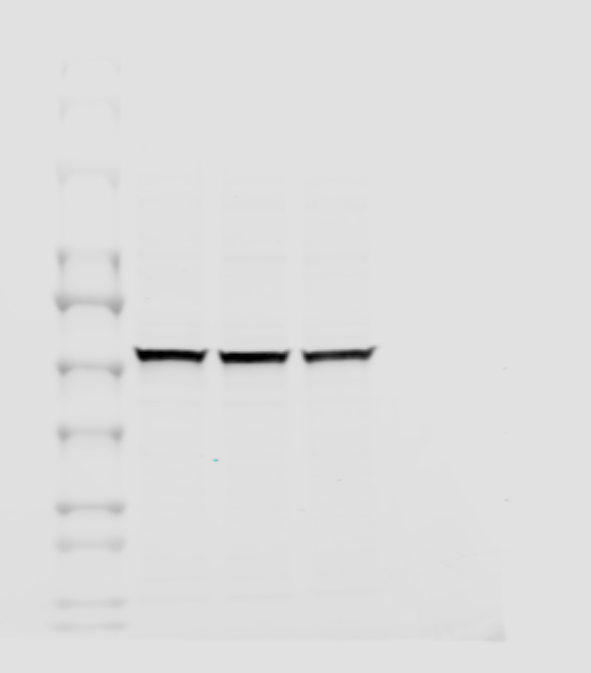

Supplement: Figure 5—figure supplement 1—source data 1. [file elife-102852-fig5-figsupp1-data1.zip › Figure 5-source data 2/Fig5Supplement1B_Actin_original.tif]

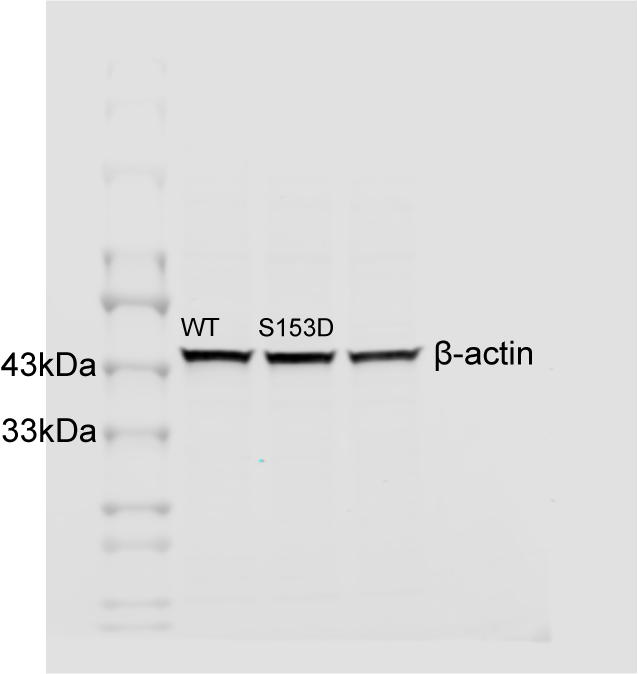

Supplement: Figure 5—figure supplement 1—source data 1. [file elife-102852-fig5-figsupp1-data1.zip › Figure 5-source data 2/Fig5Supplement1B_Actin_band_indicated.tif]

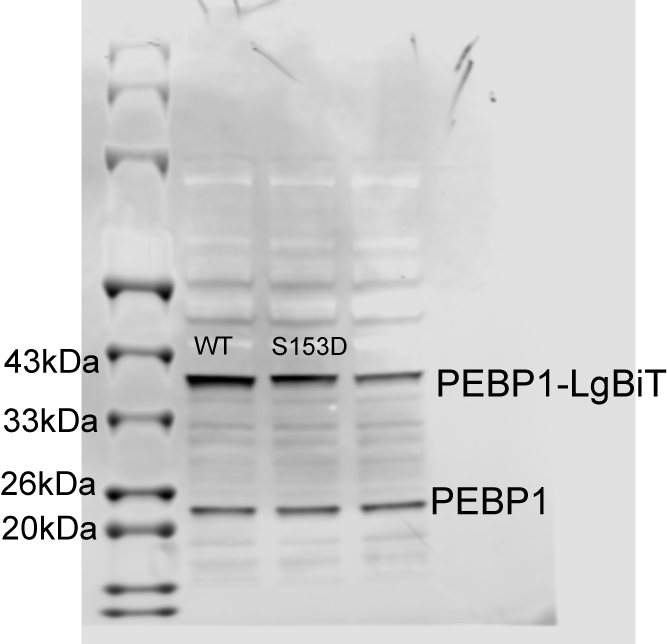

Supplement: Figure 5—figure supplement 1—source data 1. [file elife-102852-fig5-figsupp1-data1.zip › Figure 5-source data 2/Fig5Supplement1B_PEBP1_band_indicated.tif]

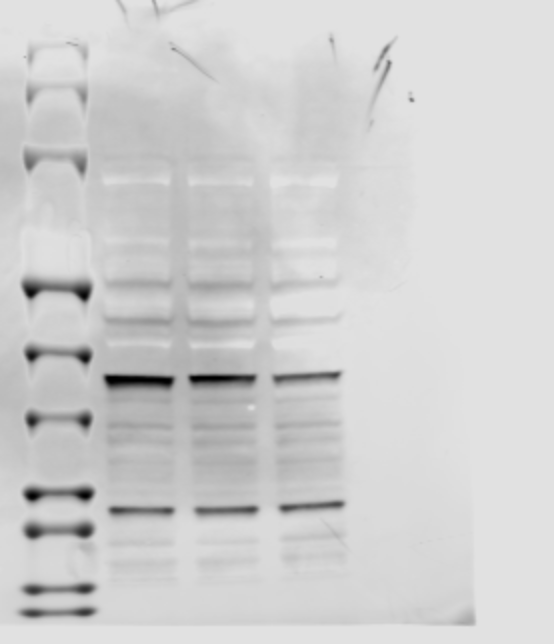

Supplement: Figure 5—figure supplement 1—source data 1. [file elife-102852-fig5-figsupp1-data1.zip › Figure 5-source data 2/Fig5Supplement1B_PEBP1_original.tif]

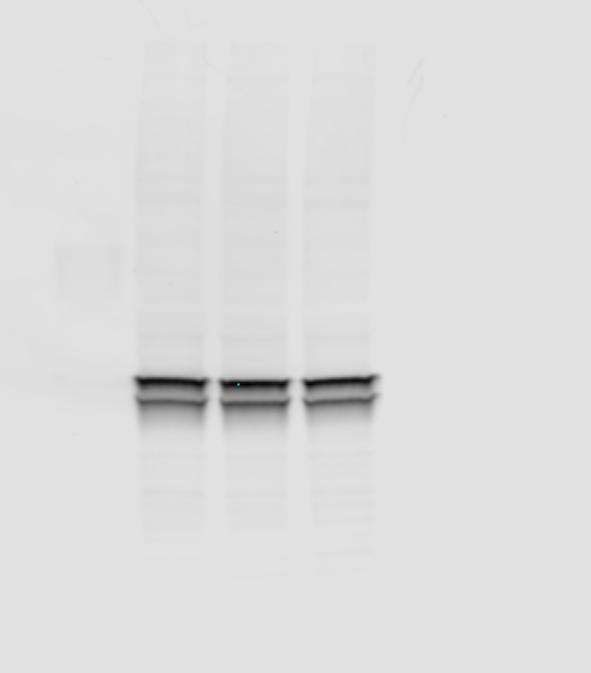

Supplement: Figure 5—figure supplement 1—source data 1. [file elife-102852-fig5-figsupp1-data1.zip › Figure 5-source data 2/Fig5Supplement1B_eIF2a_original.tif]
